# Genomic deletion of GIT2 induces a premature age-related thymic dysfunction and systemic immune system disruption

**DOI:** 10.18632/aging.101185

**Published:** 2017-03-04

**Authors:** Sana Siddiqui, Ana Lustig, Arnell Carter, Mathavi Sankar, Caitlin M. Daimon, Richard T. Premont, Harmonie Etienne, Jaana van Gastel, Abdelkrim Azmi, Jonathan Janssens, Kevin G. Becker, Yongqing Zhang, William Wood, Elin Lehrmann, James G. Martin, Bronwen Martin, Dennis D. Taub, Stuart Maudsley

**Affiliations:** ^1^ Receptor Pharmacology Unit, Laboratory of Neurosciences, National Institute on Aging (NIA), National Institutes of Health (NIH), Baltimore, MD 21224, USA; ^2^ Laboratory of Molecular Biology and Immunology, NIA, NIH, Baltimore, MD 21224, USA; ^3^ Metabolism Unit, Laboratory of Clinical Investigation, NIA, NIH, Baltimore, MD 21224, USA; ^4^ Duke University Medical Center, Durham, NC 27705, USA; ^5^ Translational Neurobiology Group, VIB Department of Molecular Genetics, University of Antwerp, Belgium; ^6^ Gene Expression and Genomics Unit, Research Resources Branch, NIA, NIH, Baltimore, MD 21224, USA; ^7^ Research Institute of the MUHC, Centre for Translational Biology (CTB), Meakins-Christie Laboratories, McGill University, Montreal, QC, H4A 3J1, Canada

**Keywords:** GIT2, thymic involution, aging, T cell differentiation, bioinformatics, CXCR4, metabolism, clock

## Abstract

Recent research has proposed that GIT2 (G protein-coupled receptor kinase interacting protein 2) acts as an integrator of the aging process through regulation of ‘neurometabolic’ integrity. One of the commonly accepted hallmarks of the aging process is thymic involution. At a relatively young age, 12 months old, GIT2^−/−^ mice present a prematurely distorted thymic structure and dysfunction compared to age-matched 12 month-old wild-type control (C57BL/6) mice. Disruption of thymic structure in GIT2^−/−^ (GIT2KO) mice was associated with a significant reduction in the expression of the cortical thymic marker, *Troma-I* (cytokeratin 8). Double positive (CD4^+^CD8^+^) and single positive CD4^+^ T cells were also markedly reduced in 12 month-old GIT2KO mice compared to age-matched control wild-type mice. Coincident with this premature thymic disruption in GIT2KO mice was the unique generation of a novel cervical ‘organ’, *i.e*. ‘parathymic lobes’. These novel organs did not exhibit classical peripheral lymph node-like characteristics but expressed high levels of T cell progenitors that were reflexively reduced in GIT2KO thymi. Using signaling pathway analysis of GIT2KO thymus and parathymic lobe transcriptomic data we found that the molecular signaling functions lost in the dysfunctional GIT2KO thymus were selectively reinstated in the novel parathymic lobe – suggestive of a compensatory effect for the premature thymic disruption. Broader inspection of high-dimensionality transcriptomic data from GIT2KO lymph nodes, spleen, thymus and parathymic lobes revealed a systemic alteration of multiple proteins (Dbp, Tef, Per1, Per2, Fbxl3, Ddit4, Sin3a) involved in the multidimensional control of cell cycle clock regulation, cell senescence, cellular metabolism and DNA damage. Altered cell clock regulation across both immune and non-immune tissues therefore may be responsible for the premature ‘aging’ phenotype of GIT2KO mice.

## INTRODUCTION

The thymus gland, responsible for T-cell maturation is most active during neonatal and pre-adolescent periods. Hematopoietic precursors originating from bone marrow translocate to the thymus and eventually these cells undergo a process of expansion, maturation and TcR (T-cell receptor) repertoire selection, and finally migrate to the periphery as mature T cells. Diminution in thymic size and function is a hallmark of normal aging and immunosenescence [1-4], in which the thymus begins to atrophy with gradual stromal adipose infiltration. This natural process of aging-related degeneration of the immune system exerts a significant impact upon quality of life in aged populations and increases the propensity for autoimmune diseases and cancers [5]. It is estimated that approximately 80% of aged individuals are afflicted with at least one chronic disease as a result of a declination of immune function [6]. In this respect the structural and functional degradation of the thymus is considered as one of the hallmarks of the natural aging process [6].

The thymus comprises a peripheral cortex and the central medulla that control separate maturational steps in the differentiation process from precursor cells to mature T cells. Loss of medullary and cortical definition and a disorganization of the corticomedullary junction are characteristic of the aged thymus [7, 8]. Age-related thymic involution results in the reduction of thymopoiesis which precedes T-cell related immuno-incompetence in an advanced age. Age-related thymic involution is classically associated with loss of gross structural integrity as well as disruption of multiple pathways involved in stress responses [6]. From several decades of concerted research it has become ever more clear that the ‘rate’ of somatic aging is a complex sum of multiple interconnected and synergistic molecular pathologies including mitochondrial dysfunction, alterations in nutrient sensation and metabolism, dysfunctions in tissue and DNA repair, chronic inflammation, attenuated stress responsivity and accumulated oxidative damage [9]. The aging process appears to be effectively coordinated across the whole body and the impact of these diverse molecular disruptions appears to effectively underpin virtually every form of disease-related process [10, 11]. Systemic and single cellular metabolic disruption, linked to either mitochondrial insufficiency or dysfunctional glucose uptake/transport, represents one of core features of aging as this pathophysiology can further entrain oxidative damage to lipids, nucleic acids and proteins. Given this, it is unsurprising that age-related diseases including Metabolic Syndrome/Type II diabetes mellitus (T2DM) [12], nonalcoholic steatohepatitis (NASH: [13]), cardiovascular disease [14], chronic kidney disease [15] and central neurodegenerative disorders such as Alzheimer's and Huntington's disease [16, 17] are strongly influenced by aberrant glucose metabolism.

A recent aspect of the aging/cellular damage/disease nexus is the emerging evidence demonstrating cellular clock and circadian rhythm disruption in the aging process [18]. Hence, considerable evidence now demonstrates that not only can cellular clock mechanisms regulate cellular tissue chronological aging but these mechanisms also strongly regulate the rate/extent of metabolic disruption, telomere stability and DNA damage during the aging process [19-21]. Considering this it is not surprising therefore that cellular clock functionality has now been linked to multiple age-related disorders including neurodegeneration/dementia [22], metabolic disorders such as NASH and Metabolic Syndrome [23, 24] and premature pathophysiological aging linked to attenuated DNA damage repair [25, 26]. It is interesting to note that a strong evolutionary synergy between clock genes and also proteins involved in the DDR process has been proposed [27]. With specific regards to classical mechanisms of aging it has also been demonstrated that alterations in cellular reduction-oxidation (redox) status (strongly linked to energy metabolism) triggers the transduction of light-entrained signals that regulate circadian clock gene transcription, suggesting that cellular responses to photo-oxidative stress may have been the evolutionary origin of the circadian clock [27]. Multiple intracellular signaling proteins involved in stress-responsive cascades, *e.g*. ATM, p53, MRE11, BRCA1 and CDKN2A, play important roles in both cell cycle/DDR control as well as circadian clock regulation [24, 26, 28-30]. Recently we have also demonstrated that G protein-coupled receptor (GPCR)-related signaling proteins, linked to premature aging, can also effectively connect energy metabolism, oxidative stress responses and DDR activity [31-34]. Hence, G protein-coupled receptor kinase interacting protein 2, also known as ADP-ribosylation factor GTPase-activating protein 2, (GIT2) is a G protein-coupled receptor-associated protein associated with cytoskeletal activity, receptor internalization and bone resorption [35-Wang et al, 2012]). GIT2 appears to exert a strong trophic effect upon multiple aspects of the aging process [31, 32]. GIT2 expression is highly sensitive to neurometabolic stress and cellular injury associated with oxidative damage or DNA double strand breaks, both pivotal controllers of pathological and normal aging [31-33]. In this regards we have demonstrated that GIT2 is an ATM kinase substrate that assists in the assembly of DDR complexes containing MRE11, p53 and BRCA1 – proteins that also serve a role in clock regulation. With respect to the *in vivo* accumulation of DNA damage, GIT2 knockout mice (GIT2KO) demonstrate essentially an advanced aging phenotype, as measured by the presence of phosphorylated H_2_AX histone adducts [33] indicating with respect to DNA damage accumulation these mice present a premature aging phenotype. Peripheral T cells and thymocytes also express GIT2 where it has a key role in regulating chemokine-mediated motility of thymocytes. Hence, GIT2 expression has been shown to negatively regulate T cell motility [36]. While it is clear that GIT2 may affect immune cell functionality, via control of T cell motility [36] we also asked whether, in the context of the keystone role GIT2 plays in connecting multiple age-related pathologies, classical age-related thymic involution was also affected as this process is considered one of the canonical aspects of physiological aging.

## RESULTS

### GIT2 genomic deletion affects total lifespan and alters indices of thymic functionality

**Figure 1:**
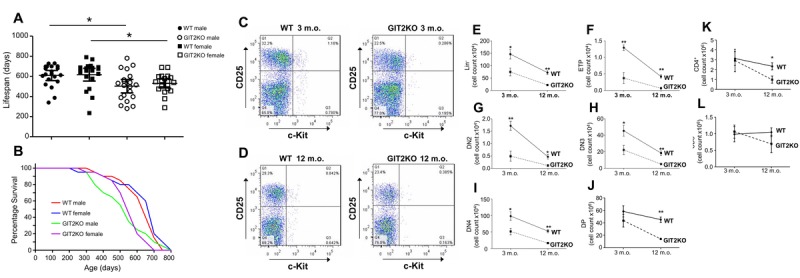
Genomic deletion of GIT2 attenuates overall murine lifespan and alters thymic T cell functionality Male and female GIT2KO overall lifespan was assessed through comparison to control wild type (WT) littermates (**A**). Survival curve analysis of GIT2KO and WT mouse cohorts across their lifespan (**B**). Representative FACS images of a male WT and GIT2KO thymus at (**C**) 3 and (**D**) 12 months of age. The x-axes show increasing c-Kit positive and the y-axes, increasing counts of CD25^+^ cells. Quadrant 3 (Q3) (bottom R) indicates ETPs (Lin^-^c-Kit^+^CD25^-^), Q2 (top R) indicates DN2 (Lin^-^c-Kit^+^CD25^+^), Q1 (top L) indicates DN3 (Lin^-^c-Kit^-^CD25^+^) and Q4 (bottom L) indicates DN4 cells (Lin^-^c-Kit^-^CD25^-^). Significant age- and GIT2KO-dependent changes (compared to WT) in Lin^-^ (**E**), ETP (**F**), DN2 (**G**), DN3 (**H**) and DN4 (**I**) cell counts. GIT2KO mice also demonstrate GIT2KO mice demonstrate significant decreases in DP (**J**) and CD4^+^ (**K**) cell counts at 12 months of age compared to WT. A non-significant trend for a similar reduction in CD8^+^ cell counts (**L**) in GIT2KO thymus compared to WT at 12 months of age was observed. Values indicated are mean ± SEM (standard error of mean). WT data are indicated in this and analogous figures with solid lines, GIT2KO data with dashed lines. Months of age is abbreviated to m.o. *p<0.05, **p<0.01.

Assessing age-related survival of male and female homozygous GIT2 knockout (GIT2KO) mice we found that GIT2KO males and females possessed a significantly shorter total lifespan compared to wildtype (WT) controls (males, p=0.0118; females, p=0.0225) (Figure 1A). The longitudinal mortality rate was accelerated in GIT2KO (male and female) compared to WT controls: in this context the males demonstrated a faster longitudinal rate of expiration compared to the females (Figure 1B). With this strong distinction in mortality rate in male GIT2KO mice we then chose to assess whether there was an alteration in the rate of thymic degradation in these males compared to WT male controls. We analyzed the presence of thymic progenitor cells, a proxy of thymic function, using FACS analysis at early time points linked to good health (*i.e*. 3 months of age, zero mortality) and also at a timepoint in which male GIT2KO mice first demonstrate a profound divergence in mortality rate compared to WT controls (*i.e*. 12 months of age, Figure 1B). A representative FACS output image of thymic progenitors (CD25/c-Kit) is shown for 3 (Figure 1C) and 12 months of age (Figure 1D). At 3 and 12 months of ages, Lin^-^ cells were significantly reduced (Figure 1E), as were ETPs (early thymic progenitors: Figure 1F), DN2 (Figure 1G), DN3 (Figure 1H) and DN4 (Figure 1I) in GIT2KO mice compared to similarly-aged-matched WT controls. We also assessed DP and CD4^+^, CD8^+^ cell counts in the GIT2KO mice. DP and CD4^+^ cell counts were significantly reduced at 12 months in GIT2KO mice (Figure 1J-K) compared to WT (Figure 1J-K). A non-significant trend of reduced CD8^+^ cells at 12 months of age in GIT2KO mice was noted (Figure 1L). To assess whether reductions in GIT2KO thymic DP and CD4^+^ cells were linked to apoptosis-related activity we measured thymic transcript levels of Bcl-xL, Bim, Bax, Bid and Caspase 3 (Figure S1). Pro-apoptotic GIT2KO Bid expression was not significantly altered in GIT2KO mice at either 3 or 12 months of age (Figure S1A). Caspase-3 transcript levels were consistently, albeit in a non-significant manner, lower at both time points in the GIT2KO mice compared to WT mice (Figure S1B). Bax expression was significantly reduced in 12 month-old GIT2KO mice compared to controls (Figure S1C). Anti-apoptotic Bcl-xL and Bim transcript levels were significantly reduced in 12 month-old GIT2KO thymocytes (Figure S1D, E). Therefore GIT2 deletion appears to attenuate cell support in the thymus, without the excessive induction of pro-apoptotic activity.

We determined the status of recent thymic emigrant cell numbers from CD31^+^/CD31^-^ ratios in the spleen (Figure S2). Compared to WT, splenic GIT2KO CD8^+^CD31^+^ and CD4^+^CD31^+^ cell counts were significantly reduced at both 3 months of age (Figure S2A, B): a distinction missing at the 12 month timepoint. GIT2KO CD8^+^CD31^-^ and CD4^+^CD31^-^ cell counts were both similar to WT at 3 months of age but only CD4^+^CD31^-^ counts were significantly elevated in GIT2^−/−^ compared to WT at 12 months (Figure S2C, D). In contrast the ratios of GIT2KO CD8^+^: CD31^+^/CD31^-^ or CD4^+^: CD31^+^/CD31^-^ were both significantly lower than those found in WT mice at the 12 month timepoint (Figure S2E, F). With respect to the status of circulatory white blood cells (WBCs) no significant differences between GIT2KO or WT mice at either time-point (Figure S2G, H) were observed: a trend of reduced levels in GIT2KO mice at both 3 and 12 months of age was evident. Circulating lymphocyte percentages and total cell counts were consistently reduced in GIT2KO mice, at 3 (significant) and 12 (non-significant) months of age, compared to WT (Figure S2I-L). To further investigate the age-dependent alterations in GIT2KO immune cellular status, we investigated bone marrow (BM) cell lineages. We assessed BM cell counts of Lin^-^CD127^+^, Lin^-^CD127^-^, LSK (Lin^-^Sca1^+^c-Kit^+^), LK (Lin^-^Sca1^-^c-Kit^+^) and CLPs (common lymphoid progenitors) (Figure S3). Levels of Lin^-^CD127^+^, Lin^-^CD127^-^ and CLPs were similar between WT and GIT2KO mice between 3 and 12 months of age (Figure S3A-C). In contrast we found an aging-specific elevation of BM LSK (Figure S3D) and LK cells (Figure S3E) in GIT2KO mice compared to WT (FACS insets for 12 month WT: Figure S3F and GIT2^−/−^: Figure S3G, LSK/LK counts). This age-dependent accumulation of LSK/LK cells in the GIT2KO BM may be associated with reduced cell motility [36] as GIT2 functions as a regulator of cytoskeletal remodeling [37]. Chemotactic targeting is also crucial for BM progenitor thymic transition. We found an age-induced decline of transcript expression in the bone marrow of GIT2KO mice for chemokine receptors involved in thymic seeding (CCR7, CCR9, CXCR4: Figure S3H-J).

**Figure 2:**
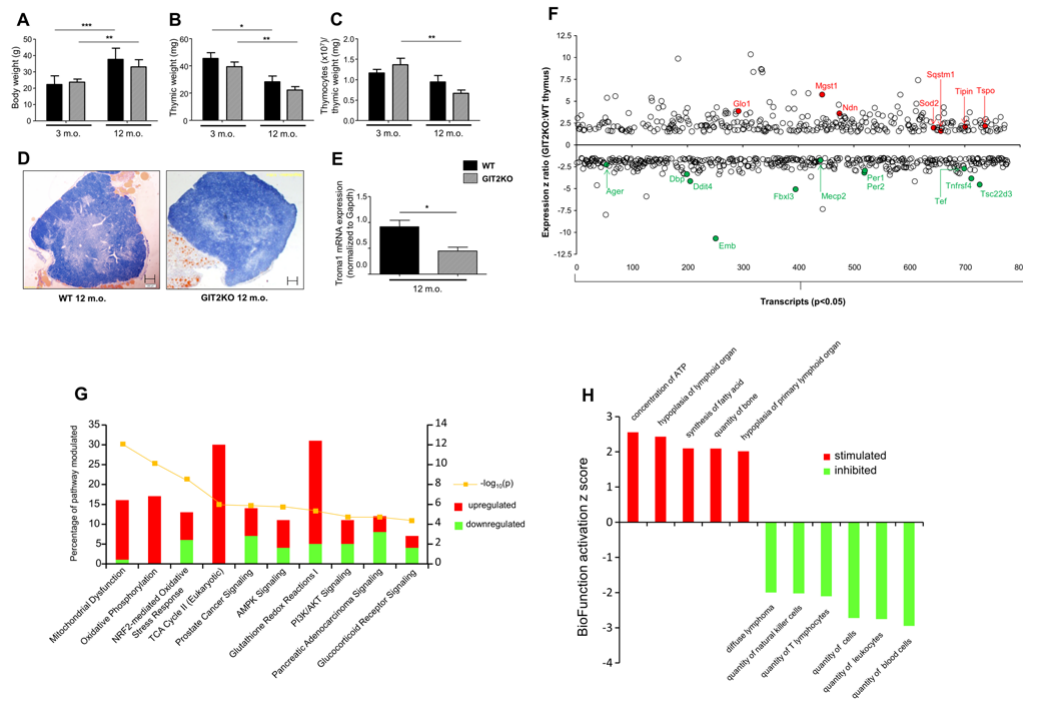
Thymic structural dysregulation in GIT2KO mice Significant age-dependent bodyweight variations observed between WT and GIT2KO mice (**A**). Thymic weight was reduced for all mice with increasing age (**B**) while only GIT2KO thymocytes, normalized to thymic weight, were reduced with age (**C**). Compared to age-matched 12 month old WT mice (left image panel) gross cortico-medullary thymic structure was disrupted in GIT2KO (right panel) mice (**D**: original magnification: 4x. Scale bar: 200 μm). Troma-I transcript expression is significantly reduced in GIT2KO thymus at 12 months of age compared to WT controls (**E**). WT and GIT2KO histogram data is indicated by black and lined bars respectively: mean ± SEM is indicated on each histogram. Months of age is abbreviated to m.o. *p<0.05, **p<0.01, ***p<0.001. Significantly-regulated (p<0.05) transcripts differentially expressed in GIT2KO versus WT thymus are indicated – specifically highlighted up- (red) or down-regulated transcripts are denoted by their official Gene Symbol (**F**). Ingenuity Pathway Analysis (IPA) Canonical Signaling Pathway analysis of transcripts differentially and significantly regulated (% of transcripts in pathway - upregulated in red, down-regulated in green are shown) between 12 month old GIT2KO and WT thymus (Top 10 enrichment probability pathways indicated: yellow line indicates pathway enrichment probability) (**G**). IPA BioFunction Z score activation analysis was performed on significantly-regulated differential transcripts from GIT2KO thymus compared to WT (Top 10 activation Z-score BioFunctions indicated) (**H**). Pathways/BioFunctions were only considered significantly populated with >2 transcripts at a *p* value of <0.05. Transcript arrays were performed in triplicate.

**Figure 3:**
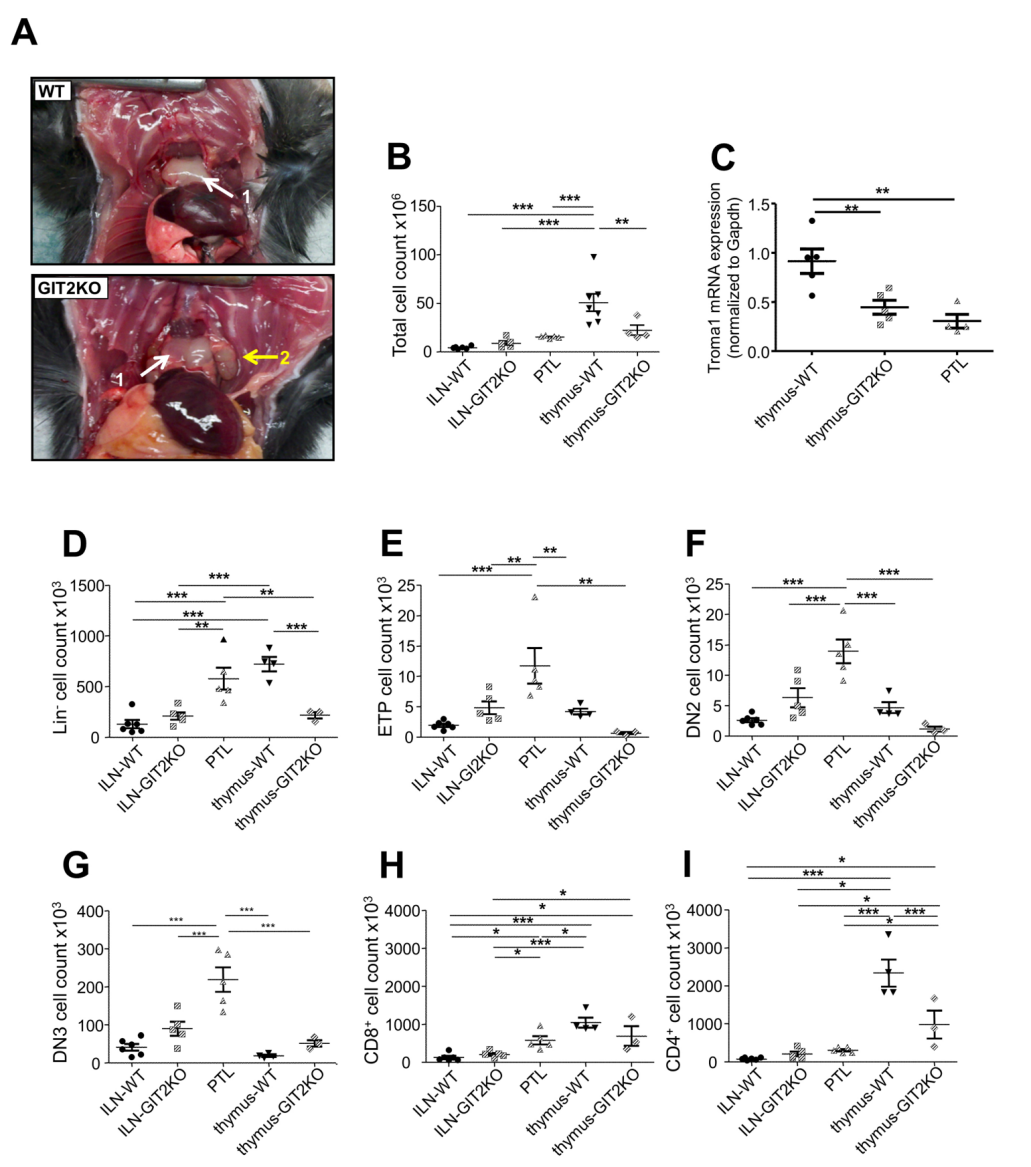
Development of idiosyncratic parathymic lobes in GIT2KO mice Large parathymic lobes (PTLs) were consistently observed in 12 month old GIT2KO mice only (**A**: 1-thymus, 2-PTL). Total cell count data measured in WT and GIT2KO inguinal and mesenteric lymph nodes (ILN and MLN respectively), thymus and PTLs (GIT2KO only) (**B**). (C) PTLs express significantly lower Troma-I expression compared to WT or GIT2KO thymus (**C**). PTLs also demonstrate significantly-distinct patterns (compared to WT and GIT2KO thymus, ILN and MLN) of total counts for Lin^-^ (**D**), ETP (**E**), DN2 (**F**) and DN3 (**G**) T cell precursors, as well as for CD8^+^ (**H**) and CD4^+^ (**I**) cells. All values indicated are mean ± SEM. For histograms WT data are represented by solid black objects, with GIT2KO data represented by lined objects. Months of age is abbreviated to m.o. *p<0.05, **p<0.01, ***p<0.001.

### GIT2KO mice exhibit early-onset physical thymic disruption

In line with the attenuated support of thymocyte function (Figure 1, S1), we next investigated the structural integrity of the thymus itself. WT and GIT2KO mice possessed similar body weights at 3 and 12 months (Figure 2A). As expected with advancing age total thymic weights were reduced in WT and GIT2KO mice from 3 to 12 months (Figure 2B) – at both time points however GIT2KO thymi were smaller than WT. An age-dependent significant decrease in thymocyte cell counts (normalized to thymic weight, *i.e*. thymocyte ‘density’) was only evident for GIT2KO mice (Figure 2C). Histologically we found that at the one year time point GIT2KO thymi failed to demonstrate a strongly-delineated cortex compared to WT mice (Figure 2D). Indicative of this cortical failure in GIT2KO thymi we found that thymic Troma-I/cytokeratin 8 (thymic cortical marker) gene expression (Figure 2E) was significantly lower compared to WT controls. To assess global GIT2KO molecular signaling alterations in the thymus we performed unbiased transcriptomic analysis of 12 month-old GIT2KO mice compared to WT controls. Commensurate with the profound structural effects of GIT2 deletion on gross thymic integrity we found that 777 transcripts from the GIT2KO mice were differentially regulated compared to WT (331 up-, 446 down-regulated, p<0.05; Figure 2F; Table S1). The most down-regulated transcripts in GIT2KO thymi included genes regulating: age-related stress responses (Ager [38]); T cell survival/regulation (Tsc22d3 [39]); structural integrity of tissues (Emb [40]); T cell autophagy (Ddit4 [41]); T cell expansion and differentiation (Tnfrsf4 [42]); susceptibility to thymic DNA damage (Mecp2 [43]) and cell cycle clock regulation/circadian rhythms (Fbxl3 [44], Dbp [45], Tef [46], Per1 and Per2 [47, 48]). Many of the thymic transcripts up-regulated in GIT2KO thymi are associated with: advanced aging (Glo1 [49]; Sod2 [50]; Mgst1 [51]; Ndn [52]; mitochondrial pathophysiology (Tspo [53]); DNA damage responses (Tipin [54]); autophagy suppression linked to thymic disruption (Sqstm1 [55]) (Figure 2F). We assessed the actual protein expression in GIT2KO thymus (compared to WT control) for multiple significantly altered transcripts (Glo1, Sod2, Per1, Ager: Figure S4A). For each of these factors our microarray data was reproduced at the level of protein expression. In addition, through *in vitro* siRNA-mediated attenuation of GIT2 expression in cultured Jurkat cells resulted in the modulation of multiple factors (Glo1, Cav1, Vdac3 – upregulated; Per1, Mgst2, Tef – downregulated: Figure S4B) in a similar manner indicated by our transcriptomic array data (see Supplementary Data).

**Figure 4:**
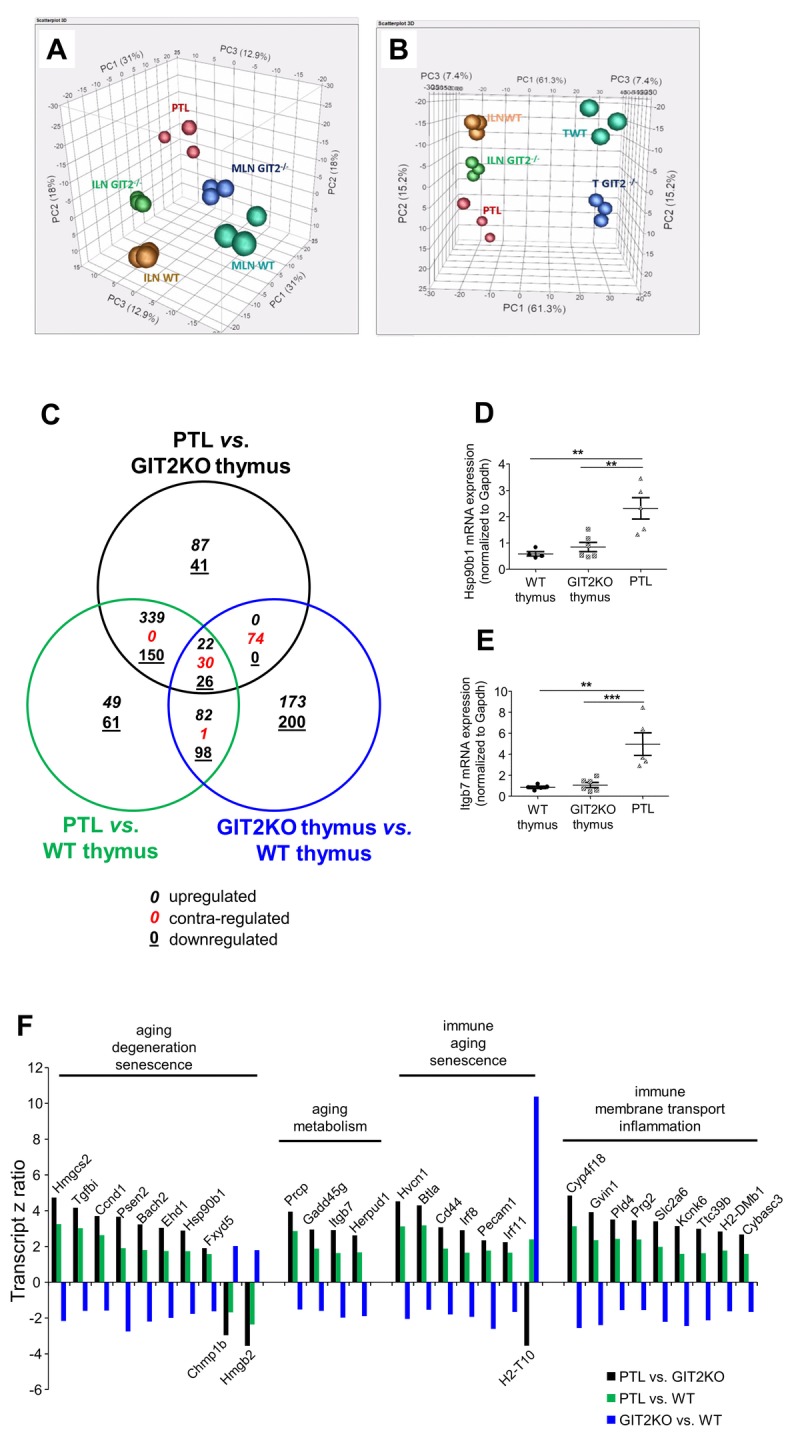
Transcriptomic analysis of GIT2KO parathymic lobes Principal component analyses were performed upon transcriptomic data from thymus (WT thymus = TWT; GIT2KO thymus = T GIT2^−/−^), WT/GIT2KO ILN and MLN, as well as PTLs from GIT2KO mice. PTL transcriptome data separates with PCA from ILN and MLN from both WT and GIT2KO mice (**A**). PTLs share component 2 with GIT2KO thymi and component 1 with WT and GIT2KO ILN (**B**). Venn diagram analysis of significantly-regulated transcripts generated by the following tissue transcriptome comparisons: GIT2KO PTLs *vs*. GIT2KO thymus (black circle); GIT2KO PTL *vs*. WT thymus (green circle); GIT2KO thymus *vs*. WT thymus (blue circle). For the Venn diagram numbers in italics represent upregulated transcripts, underlined numbers represent downregulated transcripts, red numbers represent transcripts possessing diverse expression polarities (**C**). RT-PCR validations of selected PTL transcripts Hsp90β1 (**D**) and Itgb7 (**E**: **p<0.01, ***p<0.001). Transcriptomic z ratios of 30 common and coherently regulated (same expression polarity across three Venn sectors) transcripts generated from Venn separation of PTL-associated transcripts (**F**).

At the functional signaling pathway level (Figure 2G, Table S2) GIT2KO thymic transcriptomes possessed significant alterations in (i) energy metabolism (‘*mitochondrial dysfunction*’, ‘*oxidative phosphorylation*’, ‘*TCA cycle’, ‘AMPK signaling’)* and (ii) oxidative stress resistance pathways (‘*NRF2-mediated oxidative stress response*’, ‘*Glutathione redox reactions*’, ‘*PI3K/AKT signaling’*, ‘*Glucocorticoid Receptor Signaling’*); both processes strongly implicated in the advanced aging process [31, 56, 57]. Reinforcing our pathway analysis we also assessed IPA BioFunction predictions (Table S3) generated using the significantly-affected, compared to WT controls, GIT2KO thymus transcriptomic data. Upon inspection of the predicted stimulation or inhibition of these BioFunctions (generated using the input GIT2KO vs. WT thymus data) a strong pattern of immunological depression was evident, *i.e*. stimulation of ‘*hypoplasia of lymphoid organ’* and *‘hypoplasia of primary lymphoid organ’* with a simultaneous inhibition of ‘*quantity of lymphocytes*’, ‘*quantity of mononuclear leukocytes*’, ‘*quantity of leukocytes*’ and ‘*quantity of blood cells*’ (Figure 2H, Table S3).

### Early-onset thymic disruption engenders cervical parathymicand DN3 cell count lobe development in GIT2KO mice

In addition to alterations in gross thymic structure/functionality (Figure 2) in GIT2KO mice at 12 months these mice specifically at this timepoint presented novel large ‘parathymic lobes’ (PTLs: Figure 3A). We hypothesized these novel organs could represent modified lymphoid tissues, induced in-part as a compensatory mechanism for T cell development by the early-onset deterioration of the GIT2KO thymus. Cellular analysis of the extracted GIT2KO PTL tissues revealed similar total cell counts compared to inguinal lymph nodes (ILNs) from WT or GIT2KO mice, as well as GIT2KO thymic tissue (Figure 3B). PTLs however demonstrated significantly-reduced cell counts compared to WT thymic tissue (Figure 3B). Similar to the disrupted GIT2KO thymus, PTLs exhibited significantly reduced Troma-I/cytokeratin 8 transcript expression levels (Figure 3C). Assessing the immune cell lineages in these GIT2KO-specific PTLs, we found that PTLs possessed similar levels of Lin^-^ cells as WT thymi (Figure 3D). This PTL Lin^-^ level was significantly greater than that of GIT2KO thyme or WT/GIT2KO ILNs. GIT2KO PTL tissues demonstrated ETP, DN2 and DN3 cell counts significantly greater than any ILNs or thymi of WT or GIT2KO origin (Figure 3E-G). With respect to the levels of CD8^+^ (Figure 3H) or CD4^+^ (Figure 3I) cells PTLs possessed counts similar to those seen in WT or GIT2KO ILNs as opposed to the CD8^+^/CD4^+^ count levels seen in WT or GIT2KO thymi. Therefore it appears that the GIT2KO PTLs represent, at a functional level, a ‘*pseudo-thymus’* (*e.g*. Lin^-^ cell counts) potentially from a lymph node origin (*e.g*. CD8^+^/CD4^+^) that still possesses some degree of unique functional nature (*e.g*. ETP/DN2/DN3 counts).

### Transcriptomic characterization of GIT2KO cervical PTLs

To gain an unbiased and comprehensive appreciation of the functional nature of the novel GIT2KO PTL organs we analysed the relative transcriptomic expression patterns present in PTLs and compared these to *i*) inguinal and mesenteric lymph nodes (ILNs and MLNs respectively) and *ii*) thymi of WT and GIT2KO mice. Using Principal Component Analysis (PCA), we found that global PTL transcriptomic data clustered independently of peripheral lymph nodes (ILN/MLN) from either WT or GIT2KO mice (Figure 4A). With a more targeted PCA, comparing thymus and ILNs from WT/GIT2KO with the PTLs, we found that PTLs shared one component (PC2) with ILN data, regardless of genotype, while sharing one component with GIT2KO thymic data (PC1) (Figure 4B). To analyze the differences in significantly-altered transcripts between PTLs and WT or GIT2KO tissues we performed multiple pair-wise transcriptomic comparisons. We compared significantly-regulated PTL transcriptomic expression patterns to those in both GIT2KO thymus (Table S4) and WT thymus (Table S5). 3-way Venn analysis was employed to identify any potential relationships between PTL-specific transcriptomic patterns (PTL transcriptomes compared to WT or GIT2KO thymus as ‘controls’) and those specific to the GIT2KO thymus (from Table S1, *i.e*. GIT2KO thymus compared to control WT thymus) (Table S6). Venn analysis revealed there were 22 upregulated, 30 contra-regulated (*i.e*. divergent expression polarities in at least two datasets), and 26 down-regulated transcripts shared across all the comparisons (Figure 4C). It is likely that the 30 contra-regulated common transcripts (all displaying expression polarity reversals between the PTL vs. GIT2KO thymus comparison and GIT2KO *vs*. WT thymus comparison) represents an indication of the core functional activity of the PTL compared to the dysfunctional GIT2KO thymus as well as a WT thymus. To further validate this specific data subset from the 30 contra-regulated common transcripts we independently measured Hsp90β1 and Itgb7 transcript levels in WT thymus, GIT2KO thymus and PTLs in 12 month old animals. These factors were chosen using GeneIndexer-based prioritization [58, 59] using interrogator terms related to T cell function. In accordance with our transcriptomic array data, both Hsp90β1 and Itgb7 were upregulated in PTLs compared to WT and GIT2KO thymus (Figure 4D, E: Tables S4, S5). A large proportion of this PTL-based dataset is strongly linked with metabolic aging and clock regulation [60, 61], cell senescence [62, 63] and age-related immune dysfunction [64] (Figure 4F). For each of the significantly-regulated PTL transcripts in these general categories (Figure 4F) it was evident that their regulation extent (z ratio magnitude) was consistently greater when compare to the dysfunctional GIT2KO thymus as opposed to the WT thymus. This supports our suggestion that PTL ‘functionality’ may be a reflexive compensatory process for the accelerated thymic disruption seen in the GIT2KO mice. Supportive of this posit is our identification of PTL activation of transcripts linked to T cell selection and development (Irf8 [65]; Irf1 [66]), T cell differentiation (Bach2 [67]; Btla [68]), immune cell ion channel regulation (Hvcn1 [69]), cellular chemotaxis (H2dmb1 [70]), interferon regulation of immune function (Gvin [71]), T cell activity regulation (Hmgb2 [72]) and protein sorting/endocytosis (Chmp1b [73]; Ehd1 [74]).

**Figure 5:**
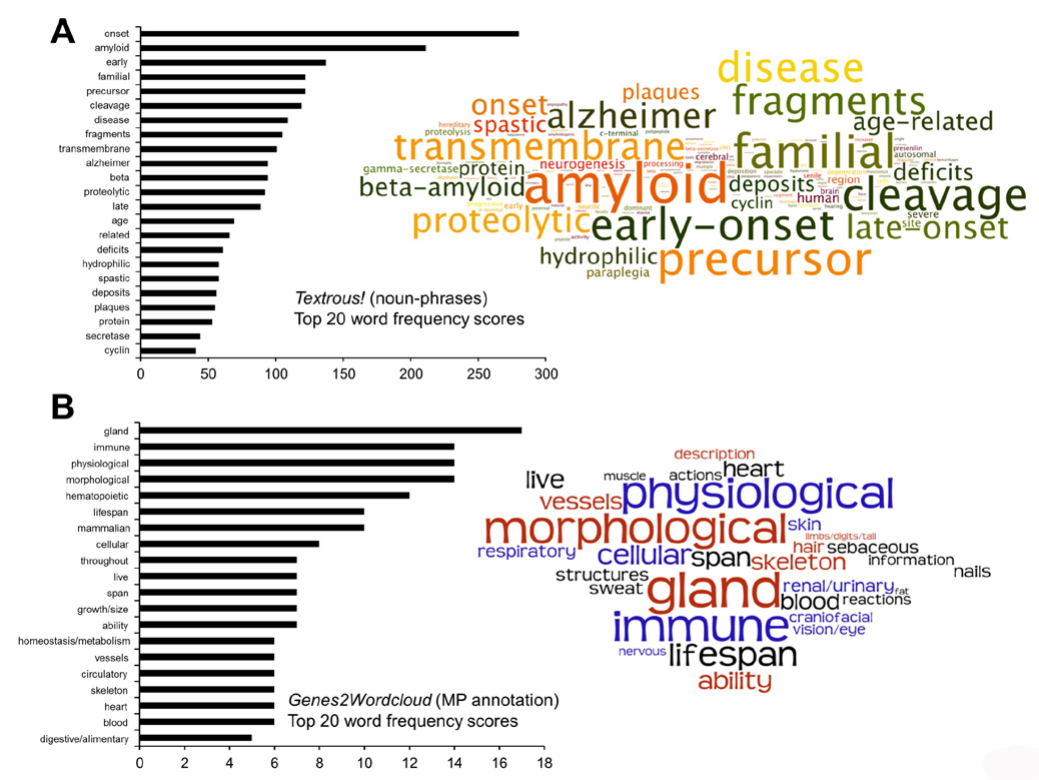
LSI-based natural language investigation of the core 30 GIT2KO PTL-associated transcripts The noun-phrase wordcloud for the 30 core PTL-associated coherently regulated transcripts was generated using the collective processing module of *Textrous!* coupled to proportional text representation output with Wordle. Noun-phrase frequency score analysis (histogram on left of panel **A**) of the resultant wordcloud was performed using WriteWords text analysis (**A**). In addition to LSI-based interpretation of the core 30 PTL-associated coherently regulated transcripts and orthogonal analysis was performed using Genes2wordcloud (Mammalian Phenotype annotation database: **B**). The wordcloud output in panel B is from Genes2wordcloud as is the associated histogram displaying the top 20 word frequencies from the cloud. For both wordclouds in panels **A** and **B**, the text size is directly proportional to the word/phrase frequency generated from either Textrous! or Genes2wordcloud.

To further investigate the functionality of this unique 30 transcript PTL dataset we employed two orthogonal latent semantic indexing (LSI) informatic platforms, *Textrous!* [75] and Genes2WordCloud [76]. *Textrous!* and Genes2Wordcloud facilitate the creation of natural language-based scientific interpretations of small datasets. Using the collective processing mode of *Textrous!* a hierarchical wordcloud was generated that indicated a strong functional bias towards age-dependent, presenilin-focused and pro-degenerative activities such as amyloid processing (Figure 5A: Table S7 - for Cosine Similarity Scores, Probability Values and Z-scores associated with the wordcloud). Among the top 20 highest frequency words semantically associated with the input 30 transcript dataset were: *early*, *onset*, *age*, *Alzheimer* and *amyloid*. The strongest risk factor for most neurodegenerative conditions, including Alzheimer's disease, is aging and therefore in this GIT2KO model it is not surprising that molecular signatures of advanced aging are present across multiple tissues in the body. It is interesting to note that presenilin-dependent gamma-secretase activity (processing Notch) has been demonstrated to modulate thymocyte development [77] and that significant immune function disruption is found in mutant presenilin-1 transgenic mice [78]. Using the mammalian phenotype analytical data module of Genes2Wordcloud to annotate the core 30 PTL transcripts we found that wordcloud interpretation reinforced the demonstration of a GIT2-specific immunological aging phenotype (Figure 5B). Among the top 20 highest frequency mammalian phenotype-based words semantically associated with the input 30 transcript dataset were: *immune*, *lifespan*, *hematopoietic* and *homeostasis /metabolism*.

### Complementary signaling pathway analysis of GIT2KO PTLs and thymus reveals effective compensatory functional ‘*transposition*’

**Figure 6:**
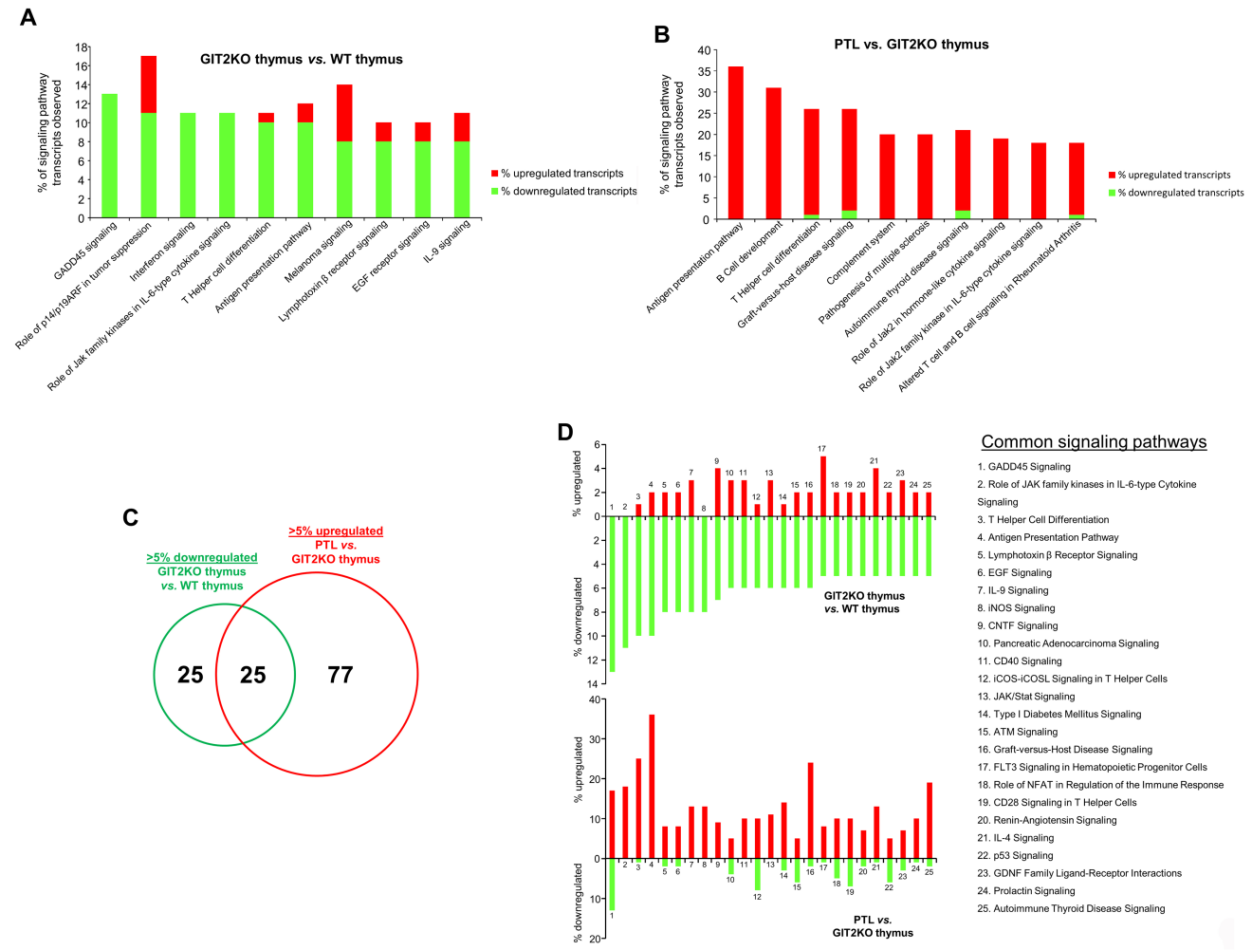
Functional signaling transposition between the GIT2KO thymus and PTLs Signaling analysis of transcripts differentially and significantly regulated between GIT2KO and WT thymus (**A**). The top 10 pathways containing the most downregulated transcripts are indicated. Upregulated (red) or downregulated (green) transcripts populating these specific pathways are indicated in the histogram. Signaling analysis of transcripts differentially and significantly regulated between the PTL and the GIT2KO thymus (**B**). Venn diagram comparison of the functional cross-over between GIT2KO thymus pathways containing the greatest number of downregulated transcripts (>5%) with the GIT2 PTL pathways containing the greatest number of upregulated transcripts (>5%) (**C**). Histograms indicate the functional transcript expression nature of the 25 shared signaling pathways (from **C**) common between the GIT2KO thymus and the PTLs (**D**).

To complement our natural language processing-based interpretation of the core 30 PTL-specific transcripts we performed classical signaling pathway analysis to investigate the potential compensatory functionality of the *de novo* PTL in the GIT2KO mice. IPA-based canonical signaling pathway analysis was performed with significant transcriptomic data comparing GIT2KO thymus *vs*. WT thymus (Table S8) and PTL *vs*. GIT2KO thymus (Table S9). We directly compared the most-downregulated (assessed by % of specific pathway transcripts downregulated – indicative of the ‘loss’ of thymic functionality in the GIT2KO mice) signaling pathways in the GIT2KO thymus (Table S8) with the most upregulated (assessed by % of specific pathway transcripts upregulated) – indicative of potential compensatory signaling pathways in the GIT2KO PTL (Table S9). Differential signaling activity in the dysfunctional GIT2KO thymus (Table S8) revealed a large number of significantly downregulated transcripts enriching pathways involved in cell growth and development *(‘GADD45 signaling’, ‘EGF receptor signaling’, ‘Role of p14/p19ARF in tumor suppression’*), T cell functionality (‘*T helper cell differentiation’, ‘Antigen presentation pathway’*) and cytokine signaling *(‘Role of Jak family kinases in IL-6 type cytokine signaling’, ‘IL-9 signaling’, ‘interferon signaling’*) (Figure 6A – top 10 downregulated – Table S8). Similar analysis of pathways modulated differentially between the PTL and GIT2KO thymus (Figure 6B – top 10 upregulated; Table S9). Many pathways that contained the greatest number of transcripts up-regulated in the PTL were functionally similar (although oppositely-regulated) to those representing this loss of function (downregulated transcripts) in GIT2KO thymus, *e.g*. ‘*T helper cell differentiation’, ‘Antigen presentation pathway’* and ‘*Role of Jak2 family kinase in IL-6-type cytokine signaling*’ (Figure 6B). To more comprehensively assess our hypothesized functional ‘*transposition*’ from the dysfunctional GIT2KO thymus to the PTLs, we assessed how much signaling pathway complementarity crossover existed between GIT2KO thymus and PTLs. Using signaling pathways containing the most down-regulated transcripts (pathways with >5% of transcripts down-regulated: Table S8) in GIT2KO thymus, and the corresponding group of signaling pathways containing the most up-regulated transcripts in the PTLs (pathways with >5% of transcripts up-regulated: Table S9), we found a considerable (25 signaling pathways; 50% of GIT2KO thymus and 25% of PTL pathways) functional cross-over between these two sets (Figure 6C). We analyzed these common functional groups and found an almost universal polarity reversal of regulation (with respect to percentage up- or downregulation of pathway-populating transcripts) between those in GIT2KO thymus data and the same pathways found using PTL data, indicating perhaps that multiple signaling functions lost in disrupted GIT2KO thymus were being *reflexively* stimulated in the PTLs (Figure 6D).

### GIT2 genomic deletion generates a systemic alteration of age-related clock gene functionality in the immune system

**Figure 7:**
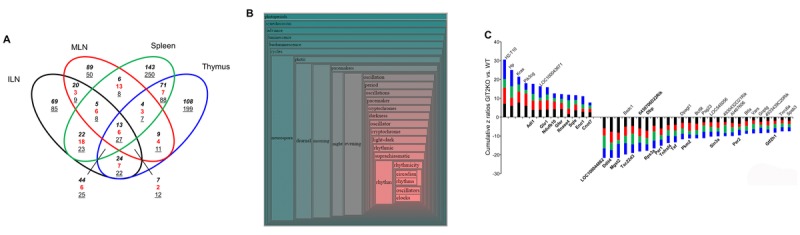
GIT2 genomic deletion engenders a consistent transcriptomic signature across multiple immune tissues Venn diagram analysis of the significant transcriptomic effects of GIT2 deletion in the ILN, MLN, spleen and thymus of GIT2KO mice compared to age-matched WT controls. In the Venn diagram numbers in italics represent upregulated transcripts, underlined numbers represent downregulated transcripts, red numbers represent transcripts possessing diverse expression polarities (**A**). Hierarchical wordcloud generated using the collective processing mode of Textrous! to investigate the functional nature of the 40 coherently-regulated transcripts common across GIT2KO ILN, MLN, spleen and thymus (**B**). Physical proximity of semantically-associated scientific words in public biomedical database curated documents indicates their strength of relationship. The most strongly associated words (with the entire input 40 transcript dataset) occur in the more intense red-hued regions of the cloud. A cumulative z ratio representation of the 40 coherently-regulated cross-tissue (ILN – black bars; MLN – red bars; spleen – green bars; thymus – blue bars) GIT2KO-spepcific factors indicates the strong presence of pro-aging/stress phenotype that is closely linked with clock gene dysfunction (stress/clock gene related transcripts possess Gene Symbols in bold typeface) (**C**).

**Figure 8:**
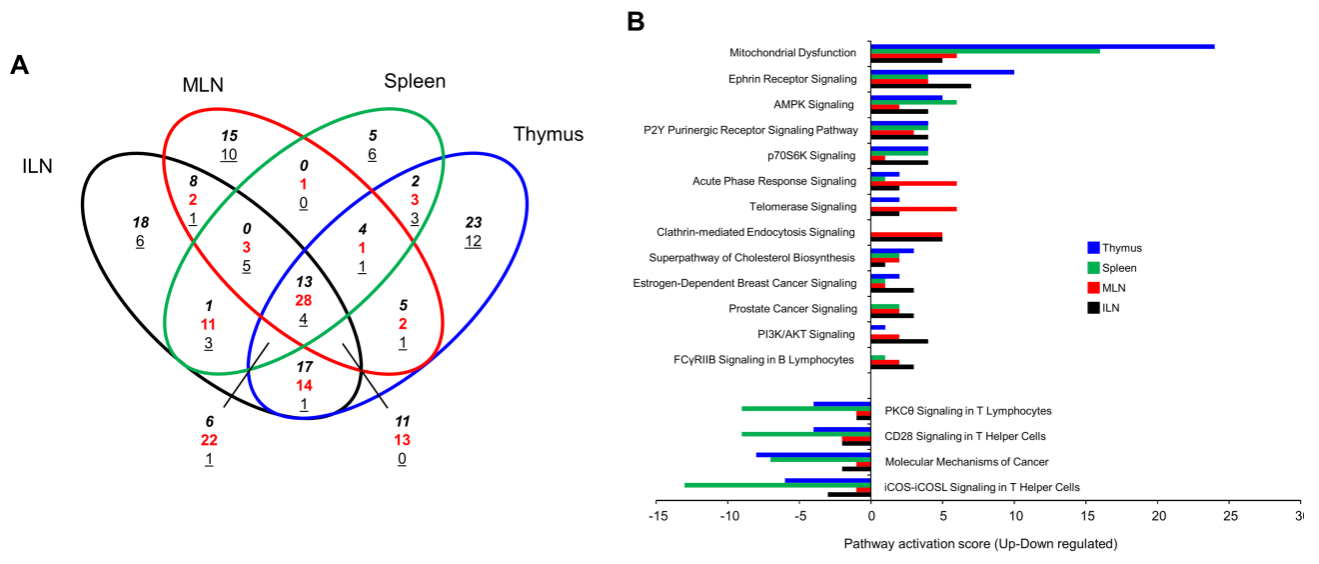
GIT2 genomic deletion engenders a consistent signaling pathway signature across multiple immune tissues Venn diagram analysis of the significant signaling pathway modulatory effects of GIT2 deletion (assessed using IPA-based canonical signaling pathway annotation of GIT2KO-specific significantly-regulated transcripts in ILN, MLN, spleen and thymus tissues) in the ILN, MLN, spleen and thymus of GIT2KO mice compared to age-matched WT controls. For each significantly-enriched signaling pathway derived from the respective transcriptomic datasets a pathway activation score was derived by subtraction of the number of downregulated transcripts from the number of upregulated transcripts that mediated the enrichment of the specific signaling pathway. In the Venn diagram numbers in italics represent upregulated transcripts, underlined numbers represent downregulated transcripts, red numbers represent transcripts possessing diverse expression polarities (**A**). The Venn diagram in panel **A** indicates that there are 17 coherently-regulated signaling pathways common to all tissues studied – 13 upregulated and 4 downregulated. The respective pathway scores of these pathways and their functional identities are indicated in the histogram in panel **B**. Pathways with a 0 pathway activation score were considered positive – a zero score indicated an even number of up and downregulated enriching transcripts.

From ours and previous research it is clear that GIT2 plays a profound role in immune system regulation: here we demonstrate that the immune system can also compensate for GIT2-associated disruption. To investigate the systemic actions of GIT2 deletion in multiple immune-related tissues we assessed the presence of consistent significantly-regulated transcriptomic expression patterns between the thymus (Table S1), ILNs (Table S10), MLNs (Table S11) and Spleen (Table S12) and of GIT2KO mice compared to WT controls. As previously demonstrated for GIT2KO thymus tissue (Figure S4) we also assessed the correlation between transcriptomic data and protein expression levels (for Ndufb10, Rnase4, Per1, Sin3a) in for example the spleen (Figure S5). For each factor we found that protein levels of these factors closely mirrored our transcriptomic data. Venn analysis of the significantly-regulated transcripts found in all of the four tissues studied (Figure 7A) revealed a core of 40 coherently-regulated transcripts common to all of the tissues (13 commonly upregulated, 27 commonly downregulated) (Table S13). We have previously shown that the Collective Processing module of *Textrous!* can efficiently generate meaningful biomedical semantic output from small data corpi [75]. Using the 40 commonly-regulated transcripts from GIT2KO ILN, MLN, spleen and thymus we found a remarkably strong and focused hierarchical wordcloud was created (Figure 7B: Table S14). The collective processing module of *Textrous!* employs latent semantic indexing (LSI) to assess the strength of correlation between all the members of the input dataset (40 coherently-regulated GIT2KO ILN/MLN/spleen/thymus transcripts) and semantically-associated nouns and noun-phrases from multiple curated biomedical databases. Biomedical textmining Cosine Similarity scores, ranging from 0 to a theoretical perfect textual correlation score of 1 [59], are employed to indicate the strength of the semantic correlation between datasets and specific words – here we found five output nouns possessing a near perfect Cosine Similarity score of over 0.9, *i.e*. ‘*oscillators*’, ‘*clocks*’, ‘*circadian*’, ‘*rhythms*’ and ‘*rhythmicity*’ (Table S14). Therefore it appears that the disruption of circadian clock-related transcript expression observed previously in the GIT2KO thymus is reproduced across multiple tissues involved in immune regulation across the body. Collective *Textrous!* processing attempts to find commonalities of textual output from a complete corpus of input data. Alternatively, the Individual Processing mode of *Textrous!* reveals the strongest individual transcript-word associations. With the same 40 transcript used for Collective Processing we found that the dataset possessed multiple input transcripts strongly linked to ‘*clock regulation’*, ‘*transcription*’, ‘*metabolism/stress’* and ‘*immune*’ functionalities (Figure S6A). Creating a summated Z-score (addition of individual transcript z ratio scores (GIT2KO *vs*. WT expression)) for each of these functional categories of activity it was evident that a profound inhibitory activity towards ‘clock regulation’ was present in the input dataset (Figure S6B). Inspection of the individual transcripts coherently regulated across ILN/MLN/ spleen/thymus in GIT2KO mice demonstrated that, aside from the expected changes in immune-related factors (Btla [79], Tnfrsf4 [80], AI467606 [81]), there were significant reductions in multiple clock-related transcripts that are also associated with premature aging and DNA damage repair actions (DDR) (Per1 [22], Per2 [48], Tef [82], Dbp [83], LOC100044862-Fbxl3-like [84] as well as transcripts related to age-related stress/metabolism alterations and cell senescence (Glo1 [85], Ndufb10 [86], Ddit4 [87], Sin3a [88], Rnase4 [89] (Figure 7C). In addition to our LSI-based interpretation of the systemic effects of GIT2 genomic deletion we also individually annotated each significant GIT2KO dataset (ILN, MLN, spleen, thymus) using Ingenuity Pathway Analysis canonical signaling pathways (ILN Table S15; MLN Table S16; spleen Table S17, thymus Table S1). As with our analysis at the transcript level we aimed to identify the qualitative and quantitative nature of the coherently-regulated signaling pathways common to each of the experimental tissues (Figure 8A). Venn analysis revealed the presence of 13 coherently upregulated (calculated by creating a Z score of Up/Down regulated transcripts that significantly populate that specific pathway) and 4 coherently downregulated signaling pathways common to all the immune tissues (Table S18). The pathway Z scores, indicating the relative degree of activation (positive Z score) or inhibition (negative Z score) of the total of 17 coherently-regulated pathways are represented in Figure 8B. Corroborating our multiple observations of an aberrant aging process in the GIT2KO mice we found consistent and significant GIT2KO-specific alterations in aging-related pathways linked to clock gene disruption: ‘*Mitochondrial dysfunction’* [90]; ‘*AMPK Signaling’* [91]; ‘*P2Y Purinergic Receptor Signaling pathway’* [92]; ‘*P70S6K Signaling’* [93]; ‘*Telomerase Signaling pathway’* [94]; ‘*Superpathway of Cholesterol Biosynthesis’* [95]; ‘*PI3K/AKT Signaling pathway’* [96]. In the context of these multiple interconnected pathways there are also a group of inter-related pathways linking immune cell function (‘*PKCθ Signaling in T Lymphocytes’*, ‘*CD28 Signaling in T Helper Cells’*, ‘*iCOS-iCOSL Signaling in T Helper Cells*’) with Ephrin receptor signaling and clathrin-mediated endocytic subcellular trafficking [97]. Taken together from both LSI-based and classical signaling pathway analysis it is evident that GIT2 genomic deletion engenders a premature state of cellular senescence/aging across multiple immune-related tissues.

## DISCUSSION

We have previously demonstrated that the GPCR-interacting protein GIT2 is strongly implicated in somatic regulation of the aging process, via the management of stress response systems linked to pathological aging, oxidative stress, metabolic disruption and eventual DNA damage [5, 31-34]. Here we investigated the potential effects of GIT2 genomic deletion upon the structure and functionality of a key immune tissue, the thymus, whose deterioration is considered an important hallmark of aging [1-4]. Compared to aged-matched WT controls, GIT2KO mice demonstrated an attenuated overall lifespan (Figure 1) as well as an accelerated idiosyncratic form of thymic involution/dysfunction (Figure 2). GIT2KO mice demonstrated premature decreases in T cell precursor levels (similar at 3 months of age to 12 month old WT mice) (Figure 1), an advanced-age reduction in DP/CD4^+^/CD8^+^ total cell counts (Figure 1), significant reductions in thymocyte density (Figure 2) and significant deficits in thymic structure and key functional regulators (Figure 2). Unbiased transcriptomic analysis coupled to signaling pathway analysis of GIT2KO thymi revealed the significant population of pathways linked to thymic aging, *e.g*. ‘*Mitochondrial Dysfunction’* [98], ‘*AMPK Signaling’* [99] and ‘*Glucocorticoid Receptor Signaling*’ [100] (Figure 2F). With IPA BioFunction analysis we found a strong indication that GIT2 genomic ablation negatively affects cellular development and functionality (elevation of ‘*hypoplasia of lymphoid organ’* – reduction of ‘*quantity of T lymphocytes’*) of the thymus at the relatively young age of 12 months (Figure 2G).

Our observed accelerated decline in GIT2KO thymocytes DP (CD4^+^CD8^+^) and CD4^+^ T cells (Figure S2) was not fully accounted for by apoptosis as we found no significant increases in pro-apoptotic mediators in GIT2KO thymocytes. Instead we observed, at 12 months of age in GIT2KOs, a decrease in transcript expression of the pro-survival mediator Bcl-xL and two of the pro-apoptotic mediators (Bim and Bax) (Figure S1). GIT2 deletion thus seems to, in-part, engender thymic dysfunction via mechanism(s) linked to reductions in cellular survival support, as suggested by the loss of an appropriate T cell maturation environment due to the Troma-I reductions (Figure 2). Our data are in accord with reports demonstrating age-related changes to thymic size and structure, including that of reduced Troma-I, which would result in decreased thymic output of mature naive T cells [3, 101]. Most significant age-related thymic changes often occur in the cortex, resulting in a negative correlation between thymic cortex, volume, and age [3]. As the thymic cortex hosts the earlier stages of thymic T cell development, its disruption in GIT2KO mice may contribute to disturbed T cell development. Cortical integrity is strongly linked to cortical thymic epithelial cell interaction with thymocytes. Genomic loss of Troma-I (keratin 8) induces mitochondrial dysfunction and somatic metabolism in an aging murine context [102, 103]. In addition to regulating mitochondrial functionality – a process strongly linked to metabolic aging [104], Troma-I is involved (like GIT2), in maintaining normal pancreatic beta cell functionality and circulatory glycemic control [34, 105]. Pancreatic regulation of the insulin/IGF-1 signaling system represents one of the prime drivers of the aging process in most species [106-109]. Our link of GIT2 to thymic involution and the creation of a premature aging phenotype, demonstrates the systemic connectivity of metabolism and immune functionality in the aging process.

Assessing the subtle role(s) of GIT2 in controlling thymic function we assessed recent thymic emigrants and observed marked reductions in newly-emigrated CD8^+^ cells as early as 3 months in GIT2KO mice (Figure S2). For newly-emigrated CD4^+^ cells from the thymus, reduced numbers in GIT2KO mice were observed at 12 months (Figure S2). These data reflect a disruption in thymic T cell differentiation with increasing age in GIT2KO mice. We next investigated the status of hematopoietic precursors in GIT2KO BM at 12 months: only LSK (Lin^-^CD127^-^Sca-1^+^c-Kit^+^) and LK (Lin^-^CD127^-^Sca-1^-^c-Kit^+^) were upregulated in GIT2KO mice. If migration of hematopoietic precursors from the BM to the thymus was negatively affected over time, an accumulation of precursors within the BM can be accounted for. Migration is vital for hematopoietic progenitor movement from BM to the thymus. Chemokine receptors CCR7, CCR9 [110, 111] and CXCR4 [112] are important for thymic seeding by hematopoietic progenitors. CCR7 and CCR9 expression were reduced in GIT2KO mice at 3 months, aligning our data with reports implicating chemokine receptors as key mediators of thymic targeting [112]. As GIT2KO mice possess reduced thymic progenitors as early as 3 months, but only observable differences in DP and CD4^+^ cell counts by 12 months of age, these results agree with earlier studies, *i.e*. despite the absence of a key thymic settling agent as CCR9, CCR9^−/−^ mice still retain some thymic settling demonstrated simply by their ability to generate thymocytes [113]. While chemotactic mechanisms have been proposed to play an important role in GIT2KO mice, our data and others (Phee et al. [36]), suggest additional factors are also important. One novel ameliorative mechanism by which GIT2KO mice attempt to provide sufficient T cells may reside in their age-dependent generation of PTLs. A second cervical ‘*thymus*’ in mice has been reported previously [114, 115]. Cervical thymus-like structure have also been observed in humans [116]. We found that the generation of cervical parathymic lobes (PTLs) was coincident with the disruption of GIT2KO thymic function (Figure 3). Despite their potential resemblance to cervical lymph nodes, these idiosyncratic PTLs did not exhibit classical peripheral lymph node-like characteristics, but expressed relatively high levels of T cell progenitors which we found to be contemporaneously reduced in GIT2KO thymus. From our PTL cell count data (Figure 3) PTLs exhibited characteristics of a hybrid thymus/lymph node organ, *e.g*. PTLs expressed higher numbers of T cell progenitors (ETP, DN2, DN3), compared to inguinal lymph nodes or even WT or GIT2KO thymus. Unbiased transcriptomic PCA analysis confirms this *gestalt* distinction from either lymph node or thymic tissue (Figure 4). These data suggest that PTLs serve as a site of extrathymic T cell development in GIT2KOs.

As we observed differences in chemokine receptor gene expression in bone marrow cells, we also investigated the expression of the cognate ligands (CCL19, CCL25, and CXCL12) for these receptors in PTLs. CXCL12 expression was significantly increased in PTLs compared to both WT and GIT2KO thymus (Figure S7). GIT2 has been implicated in the CXCL12/CCL25-mediated regulation of the *in vitro* motility of DP thymocytes [36]. Earlier studies suggested that CXCL12 and CXCR4 are involved in T cell precursor expansion in both fetal and adult thymi *in vivo* and any defect in T cell development caused by a CXCR4 mutation is not caused by reduced expression of the anti-apoptotic mediator Bcl-2 [117]. Our data suggest that CXCR4/CXCL12 are also implicated in the GIT2-dependent model of dysfunctional thymic function as T cell development is disrupted and the anti-apoptotic mediator Bcl-xL is significantly reduced at 12 months of age. There has been much debate on the relevance of extrathymic T cell development in a variety of organs including the gut [118, 119], skin, and liver and their physiological relevance to thymic T cell development [120]. We contrasted the disrupted GIT2KO thymus to the PTL at a cellular and functional signaling level to assess whether functions that were lost in the GIT2KO thymus were effectively recapitulated in the transcriptomic profile of the PTLs. Comparing the GIT2KO thymus signaling pathways populated by the greatest percentage of downregulated transcripts with the pathways populated by the greatest percentage of upregulated transcripts we found that at a signaling pathway level, these two tissues were almost completely inverted mirror images of each other (Figure 6D). For example, multiple pathways associated with T cell development were among the most downregulated in the GIT2KO thymus (Figure 6A), while these pathways were amongst the most upregulated in the GIT2KO PTLs (Figure 6B). Our data suggest that the PTL is a functional hybrid tissue between the thymus and lymph nodes (Figure 3D, Figure 4A-B) that can be generated in mice and may serve as an ameliorative mechanism to counter the abnormal immunological aging profile (Figure 5) present in GIT2KO mice.

Our analyses of PTL-unique transcript datasets (Table S6 – indicated by asterisk) uncovered a core of 30 GIT2KO PTL-specific transcripts that were functionally linked to biomedical text terms associated with age-related neurodegeneration, cell senescence, lifespan regulation, metabolic disruption and immunomodulation (Figures 4, 5). Hsp90β1 and Itgb7 were specifically and markedly upregulated in PTLs compared to WT/GIT2KO thymi (Figure 6D, E). Hsp90β1 is a molecular chaperone for integrins such as Itgb7 [121]. In tamoxifen-inducible Hsp90β1^−/−^ mice, T cell development is severely compromised with the inability of thymocytes to develop beyond the DN stage. This was strongly correlated with thymic atrophy [121]. At seven months of age, DP and SP (single positive) cells were almost absent in Hsp90β1^−/−^ mice. It has been reported that without β-7 integrins, lymphopoiesis may proceed [122], our current data indicate that Itgb7 may be important in the functioning of the PTLs which express higher numbers of T cell progenitors than thymi, along with higher Itgb7 gene expression. With respect to the strong pro-aging/senescence signature induced in thymic tissues by GIT2 deletion it is interesting to note that multiple High Mobility Group (HMG) proteins (HMGCS2, HMGB2) are affected. HMG proteins are stress-sensitive DNA-modulatory factors involved in transcription/translation and DNA repair activities. We have previously demonstrated that HMG proteins are functionally linked with GIT2 in neuronal cells [33] where they likely coordinate the actions of GIT2 in the DDR response and PARP activity modulation [123]. HMGCS2 is one of the controlling enzymes in the mitochondrial mechanism of ketogenesis, an energy derivation process often entrained in the context of disrupted glucose metabolism and altered aging [49, 102]. HMGCS2 functionality is not only associated with ketogenesis but also with fatty acid β-oxidation, a compensatory process which is commonly observed in the context of disrupted aging mechanisms, age-related neurodegenerative conditions and age-related metabolic disruption [60, 78, 124]. Confirming our observations of disrupted Troma-I expression in the GIT2KO model it has also been demonstrated that reductions in Troma-I can also modulate HMGCS2 expression and in doing so modulate energy metabolism and ketogenic activity [103]. HMGB2 is also critically involved in regulatory mechanisms that, control DNA damage [125], cell senescence [126], innate immune responses and neuroinflammation [127, 128] as well as stem cell proliferation and neurogenesis [129]. In addition to the specific GIT2-dependent alteration of HMG proteins multiple additional proteins that regulate age-related energy metabolism (Prcp [130], age-related DNA damage (Bach2 [131], age-related hematopoietic network regulation (Irf8 [132], immunosenescence (Pecam1 [133] and ER stress/amyloid related regulation of insulin secretion (Herpud1 [134-136]) were altered in GIT2KOs. From our work it was evident that GIT2 deletion in our models was potentially associated with cellular senescence. To further investigate this, we sought to identify whether any GIT2-associated factors were also associated with the well-characterized senescence-associated secretory phenotype (SASP). Using a canonical list of 80 SASP-associated proteins extracted from REACTOME (www.reactome.org) we cross matched this with GIT2KO data from thymus, PTL, spleen and lymph nodes. Across these multiple tissues we found that GIT2 deletion significantly altered the expression of over 20% (17 from 80) of the REACTOME SASP pathway list (Figure S8) that revealed a highly interconnected group of factors strongly linked to senescent/DNA stability activity (Enrichr-based Reactome 2015 analysis - http://amp.pharm.mssm.edu/Enrichr/enrich: Table S19).

As genomic GIT2 deletion appeared to modify physiological immune system aspects in the thymus and the PTL that were functionally related to additional immune-relates tissues, *e.g*. ILN/MLN (Figures 3D, 4A-B), we next investigated the functional effects of GIT2 deletion upon splenic and ILN/MLN tissues to assess whether a core ‘GIT2-functional signature’ was present in multiple immune-related sites. Using the significant transcriptomic patterns of gene expression in thymus, spleen, ILN and MLN in GIT2KO mice we discovered a functional core of forty GIT2-dependent gene transcripts coherently regulated (expression polarity versus WT control) in all four tissues (Figure 7A). Using *Textrous!* to investigate this core dataset a strong clock gene-associated phenotype was evident (Figures 7B, S6A). Multiple transcripts possessing well-characterized roles in circadian rhythm control, *e.g*. Dbp, Tef and Per2, were coherently regulated in all the GIT2KO immune tissues (Figure 7C). GIT2 genomic deletion also generated a coherent pathway signature (Figure 8A) across the multiple tissues, *i.e*. 13 pathways commonly upregulated and four pathways commonly downregulated (Figure 8B). Nearly all of the coherent and commonly-controlled signaling pathways were strongly linked to cellular clock control mechanisms, e.g. ‘*Mitochondrial Dysfunction’* [90], ‘*AMPK Signaling’* [91], ‘*P70S6K Signaling’* [93], *‘Telomerase Signaling*’ [94], ‘*Superpathway of Cholesterol Biosynthesis’* [95] and ‘*PI3K/AKT Signaling’* [96]. Additional GIT2KO multi-immune tissue signaling pathways also demonstrated multiple functional data corpi links as ‘*P2Y Purinergic Receptor Signaling’* has been shown to strongly control the AMPK signaling pathway [92]. In addition the quality and nature of ‘*Ephrin Receptor Signaling’* is strongly dependent on ‘*Clathrin mediated endocytosis signaling’* mechanisms [97].

Our data demonstrates that loss of GIT2 protein causes an attenuated lifespan and induction of a premature aging/senescent state in the thymus as well as other immune-related tissues. This premature GIT2KO aging phenotype is associated with the disruption of cellular clock regulation and DDR activity. In this respect, our data are synergistic with previous research linking GIT2 with multiple physiological/pathophysiological activities associated with the aging process, *e.g*. oxidative damage, dysglycemia and DNA damage [32-34]. Hence GIT2 appears to likely act as a powerful mechanistic factor in the aging process through its control of cellular aging via clock gene regulation. In addition to an evident common evolutionary mechanism for clock and DDR genes [27] it has been shown that a strong functional interaction occurs between clock-DDR functions [137-139] that involves several GIT2 interaction partners such as MRE11, ATM and p53 [33]. GIT2 therefore may serve as a functional bridge between cellular senescence, clock regulation and DNA damage, and thus possess the capacity to potently control the accumulation of age-related cellular damage. As age-related accumulation of DNA damage and metabolic dysfunction appear to synergize to accelerate the onset of aging-related disorders it is interesting to note that already therapies targeting clock-regulation mechanisms are currently showing promise [140-142].

## MATERIALS AND METHODS

### Animal handling

All animal studies performed were approved according to the guidelines of the NIA Animal Care and Use Committee. Mice were maintained in a 12h light/dark cycle on an *ad libitum* regular diet. Male GIT2^−/−^ (GIT2KO) (n=3-16) and C57BL/6 WT mice (n=3-8), at 1, 3 and 12 months of age, were bred in the NIA animal facility. Lifespan measurements at this facility for the wild-type (WT) C57/BL6 indicated that for both male and female GIT2KO mice presented a significantly-reduced total lifespan (n≥20 per group: male WT: 609.3±24.2 days; female WT: 617.25±30.15; male GIT2^−/−^: 503.15±32; female GIT2^−/−^: 529.35±21.4). They were fasted 12h before experimentation, sacrificed by carbon dioxide asphyxiation and whole body and organ weights were recorded. GIT2KO gene-trap animals [143], based on a standard C57BL/6 background, were transported from Duke University (Richard Premont, Durham, NC) and bred at the National Institute on Aging under NIH protocol numbers, 432-LCI-2015 and 433-LCI-2015, according to approval of the Institutional Review Board. Prior to experimentation, the animals were labelled with an ID number and thus, during experimentation, the investigator(s) were blinded to the animal genotype(s). Equal numbers of animals belonging to the control and/or experimental groups were experimented on the same day. Overall, the order of animal experimentation was performed in a random manner.

### Blood cell counts

Prior to sacrifice, mice were bled retro-orbitally and blood cells were counted using a Horiba ABX Micros 60 (Horiba Medical).

### Flow cytometry

Bone marrow cells were collected from femurs and disrupted into a single cell suspension. All other immune organs were dissociated in RPMI media (Life Technologies, Carlsbad CA). Cell suspensions were then passed through 70 μm nylon mesh strainers (BD Falcon). RBCs (red blood cells) were lysed with ammonium chloride buffer (Quality Biological) and washed twice with HBSS/1%BSA/0.1% sodium azide. Antibodies (BD BioSciences, eBioScience or BioLegend) were subsequently used. The antibodies used in the study are outlined as follows: For the bone marrow: CD3 PE (phycoerythrin) (clone 17A2), CD8a PE (53-6.7), CD4 PE (L3T4), CD19 PE (1D3), B220 PE (RA3-6B2), Ly6G PE (RB6-8C5), CD127 PE-Cy5 (A7R34), Sca-1 FITC (D7), C-Kit APC (2B9), viability e506. For the assessment of early thymic progenitors (ETPs), the following antibodies were used: c-Kit FITC (2B8), CD11b PE (MAC-1), CD11C PE (N418), B220 (RA3-6B2), CD3 (17A2), CD8a (53-6.7), CD19 (MB19-1), CD 127 (A7R34), Ter 119 (Ter 119), TCRB (H57-597), TCRGD (GL3), NK 1.1 (PK 136), GR1 (RB6-8C5), PanNK PE (DX5), CD25 PerCP-Cy5.5 (PC61). For other assessments in the thymus, the following antibodies were used: CD4 e450, CD8 PerCP, IgM APC, B220 FITC, fixable viability e506. For the peripheral lymph nodes and novel parathymic lobes the following antibodies were used: (i) CD31 FITC (390), CD62L PE (MEL-14), CD8 PerCP, CD44 APC (IM-7), CD4 e450, viab e506; (ii) B220 FITC, CD23 PE (B3-B4), CD21 e710 (4E3), IgM APC, CD1d e450 (1B1), viab e506, (iii) B220 FITC, CD19 PE, IgM APC, GL7 e450, viab e506. Cells were examined on a FACSCanto II (Becton Dickinson, Franklin Lakes NJ) and the data were analyzed using FlowJo (Ashland, OR). In all analyses, the primary gate was set on total lymphocytes, using forward and side scatter. Dead cells stained with Fixable Viability Dye eFluor® 506 (eBioscience) were excluded. The following were assessed in bone marrow: LSK and LK cells were identified by first gating out all Lin^-^ cells that were negative for the following lymphoid markers: CD3 (clone 2C11), CD4 (RM4-5), CD8a (53-6.7), CD19 (6D5), CD45R (RA3-6B2), Ly6G (8C5, MAC1) and Ter119). From Lin^-^ cells, those that were CD127^+^c-Kit^+^Sca-1^+^ were identified as common lymphoid progenitors (CLPs). Cells that were CD127^-^c-Kit^+^Sca-1^+^ or CD127^-^c-Kit^+^Sca-1^-^ were designated as LSK and LK progenitor cells, respectively. In the thymi: ETPs were identified by first gating out all Lin^-^ cells and then further subdividing based on CD25 and c-Kit expression. ETPs were CD25^-^c-Kit^+^ or DN, DN2 (CD25^+^c-Kit^+^), DN3 (CD25^+^c-Kit^-^) and DN4 (CD25^-^c-Kit^-^). Lin^+^ cells were divided based on CD4 and CD8 expression. In the spleen, after gating on lymphocytes and live cells, cells were analyzed based on their expression of the various epitopes examined. CD31^+^/CD31^-^ are indicative of recent thymic emigrants in the spleen.

### RT-PCR

The RNeasy Mini kit (Qiagen) was used for thymic cell mRNA extraction. Reverse transcription was performed using proprietary kits (Life Technologies, Carlsbad CA). Genes were normalized to GAPDH. RT-PCR was performed using the ABI Prism 7300 Sequence Detector (Applied Biosystems, Carlsbad CA). Thymic cells were subsequently assessed for pro-apoptotic mediator caspase-3 and pro-apoptotic mediators of the Bcl-2 family: Bid, Bim, Bax and the pro-survival mediator Bcl-xL. Bone marrow cells were assessed for chemokine receptors CCR7 and 9, and CXCR4. Whole thymic organs and novel parathymic lobes (PTLs) were assessed for the cortical marker Troma-I (keratin 8), Hsp90β1, Itgb7, CCL19 and 25, and CXCL12.

The primer sequences; GAPDH-F: 5′-ACCACAGTCCATGCCATCAC-3′; GAPDH-R: 5′-TCCACCACCCTGTTGCTGTA-3′; Bax-F: 5′-GTGAGCGGCTGCTTGTCT′3′; Bax-R: 5′-GGTCCCGAAGTAGGAGAGGA-3′; Bcl-xL-F: 5′-TGACCACCTAGAGCCTTGGA-3′; Bcl-xL-R: 5′-GCTGCATTGTTCCCGTAGA-3′; Bid-F: 5′-GTGAGGAACTTGGTTAGAAACGA-3′; Bid-R: 5′-CAGGCCAAGGTCTTTCCAT-3′; Bim-F: 5′-AGGGCGGGTACATTCTGA-3′; Bim-R: 5′-GGCGTGTTTACCCTAGTGTCTT-3′; Caspase-3-F: 5′-GAGGCTGACTTCCTGTATGCTT-3′; Caspase-3-R: 5′-AACCACGACCCGTCCTTT-3′; CCL19-F: 5′-TGTGGCCTGCCTCAGATTAT-3′; CCL19-R: 5′-AGTCTTCCGCATCATTAGCAC-3′; CCL25-F: 5′-GAGTGCCACCCTAGGTCATC-3′; CCL25-R: 5′-CCAGCTGGTGCTTACTCTGA-3′; CCR7-F: 5′-ATTTCTACAGCCCCCAGAGC-3′; CCR7-R: 5′-AGCACACCTGGAAAATGACA-3′; CCR9-F: 5′-GGCTGGTCTGCATTATCTTGA-3′; CCR9-R: 5′-CATGCCAGGAATAAGGCTTG-3′; CXCL12-F: 5′-CCAAACTGTGCCCTTCAGAT-3′; CXCL12-R: 5′-ATTTCGGGTCAATGCACACT-3′; CXCR-4-F: 5′-TGGAACCGATCAGTGTGAGT-3′; CXCR-4-R: 5′-GGGCAGGAAGATCCTATTGA-3′; HSP90β1-F: 5′-AGGGTCCTGTGGGTGTTG-3′; HSP90β1-R:5′-CATCATCAGCTCTGACGAACC-3′; ITGB7-F: 5′-TGTGCATGGTGCAAACAAC-3′; ITGB7-R: 5′-GCGAGCCAGTAGCTCCTCT-3′; Troma I-F: 5′-GGGGGTTGGGAAATGAGTAT-3′; Troma I-R: 5′-CAGAGATACAGGGCATGCAA-3′. Most of the primer sequences were designed to be intron-spanning, if applicable (https://www.roche-appliedscience.com/sis/rtpcr/upl/index.jsp?id=uplct_030000).

### Histological analysis

Thymi were snap-frozen for histological assessment. Alcohol-free Oil red O staining was performed on frozen OCT (optimal cutting temperature)-fixed, 5 μm thymic sections. Sections were dipped in Oil Red O for 10 minutes, followed by water, hematoxylin (10 dips), tap water, clarifier (10 dips), tap water, bluing agent (10 dips), tap water and aqueous mounting media was used to coverslip the sections.

### Transcriptomic microarray analysis

RNA isolation of 12-month old thymi and PTLs, subsequent cDNA generation, labeling and hybridization to Illumina Sentrix Mouse Ref-8 Expression BeadChips (Illumina) were performed as previously described [16]. We have deposited the raw transcriptomic data at GEO/ArrayExpress under accession number GSExxx. All details are MIAME compliant. Gene array data were analyzed using DIANE 6.0 as described previously [16].

### Bioinformatic analyses

Ingenuity Pathway Analysis (Redwood City, CA), was used for BioFunction and Canonical Signaling Pathway analyses for the high-dimensionality transcriptomic data. The latent semantic indexing-based natural language processor, *Textrous!* was employed for further novel informatics analyses [58, 75].

### Statistical analysis

Data are presented as mean ± SEM. Where applicable, data are represented in dot plots (Figs 5, 6, S3) to indicate the variation between data points within a study group, providing further insight as to the distribution of the data. Statistical analysis was performed using GraphPad Prism, v5. Two-way ANOVA followed by a Bonferroni post-hoc test was performed for all panels in Figs 2 and 3 and panels A-C for Fig 4. A one-way ANOVA was performed followed by a Newman Keuls test for graphs with comparisons that involved at least 3 study groups for all other figures. For comparisons with only 2 study groups, a two-tailed Student's t-test was performed. *p* ≤ 0.05 was considered significant.

## SUPPLEMENTARY MATERIALS TABLES AND FIGURES








































