# Supplementary material for: Genomic deletion of GIT2 induces a premature age-related thymic dysfunction and systemic immune system disruption
Source: Aging (Albany NY). 2017 Mar 4;9(3):706–30. doi: 10.18632/aging.101185 (PMC5391227; doi:10.18632/aging.101185)
Supplement: Supplementary file 1 [file aging-09-706-s001.pdf]

SUPPLEMENTARY MATERIALS

Please browse the links in Full Text version of this manuscript to see Supplemental Tables.

Supplemental Tables

Table S1. **Transcripts significantly regulated differentially between the GIT2KO thymus and the WT thymus.** Supplemental File 1

Table S2. Full metabolic and signaling pathway analysis of transcripts significantly regulated in GIT2KO thymus compared to WT thymus. Supplemental File 2.

Table S3. IPA-based BioFunction enrichment annotation of transcripts significantly regulated in a differential manner in GIT2KO thymus compared to WT control thymus. Supplemental File 3.

Table S4. Transcripts significantly and differentially regulated in GIT2KO PTLs versus GIT2KO thymus. Supplemental File 4.

Table S5. Transcripts significantly and differentially regulated in GIT2KO PTLs versus WT thymus. Supplemental File 5.

Table S6. Venn diagram analysis of GIT2KO parathymic lobe (PTL) transcriptomic profiles. Supplemental File 6.

Table S7. Textrous!-based natural language collective processing output from the core of 30 GIT2KO PTL-regulated transcripts. Supplemental File 7.

Table S8. Canonical signaling pathway analysis of transcripts significantly regulated in GIT2KO thymus compared to WT thymus. Supplemental File 8.

Table S9. Canonical signaling pathway analysis of transcripts significantly regulated in GIT2KO PTLs compared to GIT2KO thymus. Supplemental File 9.

Table S10. Transcripts significantly regulated differentially between the GIT2KO inguinal lymph node (ILN) and WT ILN. Supplemental File 10.

Table S11. Transcripts significantly regulated differentially between the GIT2KO mesenteric lymph node (MLN) and WT MLN. Supplemental File 11.

Table S12. Transcripts significantly regulated differentially between the GIT2KO spleen and WT spleen. Supplemental File 12.

Table S13. Venn diagram separation of transcripts significantly regulated differentially between the GIT2KO inguinal lymph node (ILN), mesenteric lymph node (MLN), Spleen and Thymus compared to WT tissue counterparts. Supplemental File 13.

Table S14. Collective Processing Textrous! analysis of coherently-regulated transcripts common across GIT2KO ILN, MLN, spleen and thymus tissues. Supplemental File 14.

Table S15. Ingenuity Pathway Analysis-based canonical signaling pathway analysis of significantly regulated transcripts differential between GIT2KO ILN compared to WT controls. Supplemental File 15.

Table S16. IPA-based canonical signaling pathway analysis of significantly regulated transcripts differential between GIT2KO MLN compared to WT controls. Supplemental File 16.

Table S17. IPA-based canonical signaling pathway analysis of significantly regulated transcripts differential between GIT2KO spleen compared to WT controls. Supplemental File 17.

Table S18. Venn diagram analysis of significantly populated Ingenuity Pathway Analysis canonical signaling pathways from GIT2KO versus WT control ILN, MLN, spleen and thymus transcriptomic data. Supplemental File 18.

Table S19. Enrichr-based Reactome-2015 analysis of SASP (senescence-associated secretory phenotype) factors affected by GIT2 deletion. Supplemental File 19.

Supplemental Figures.

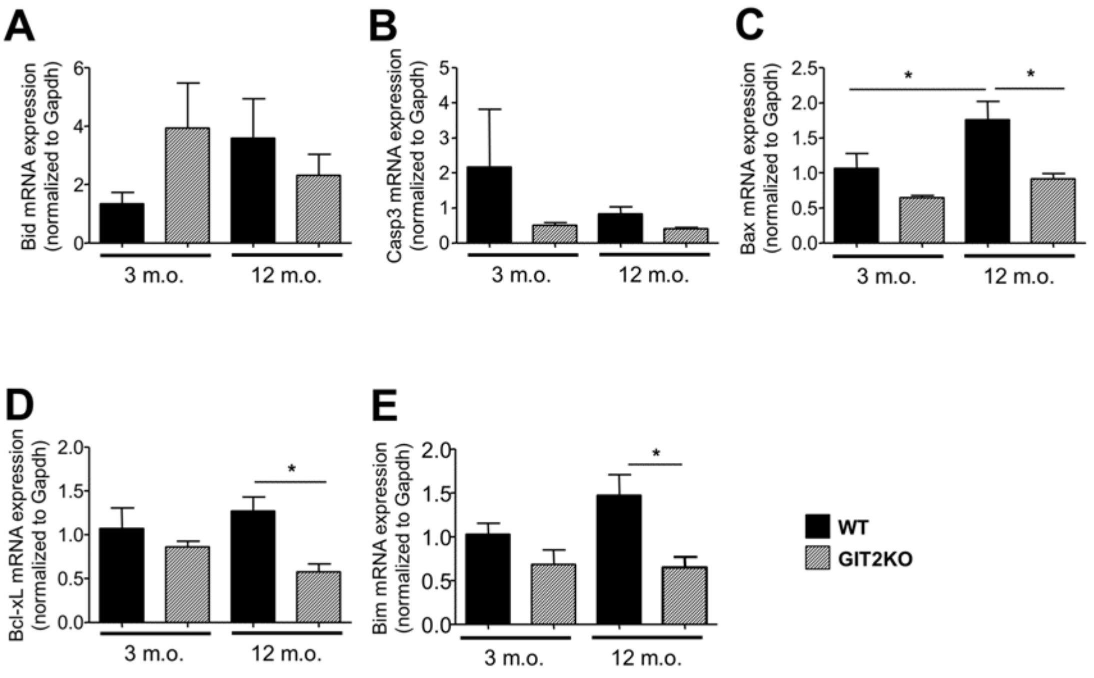

Figure S1. Altered apoptotic marker expression in GIT2KO thymocytes. Thymocyte transcript expression of apoptotic regulators Bid (**A**), caspase-3 (**B**), Bax (**C**), Bcl-xL (**D**) and Bim (**E**). All values indicated are mean ± SEM. For line charts (A-C) WT data is indicated with solid lines, GIT2KO data with dashed lines. For histograms, WT data are represented by black bars with GIT2KO data represented by lined bars. Months of age is abbreviated to m.o. *p<0.05, **p<0.01.

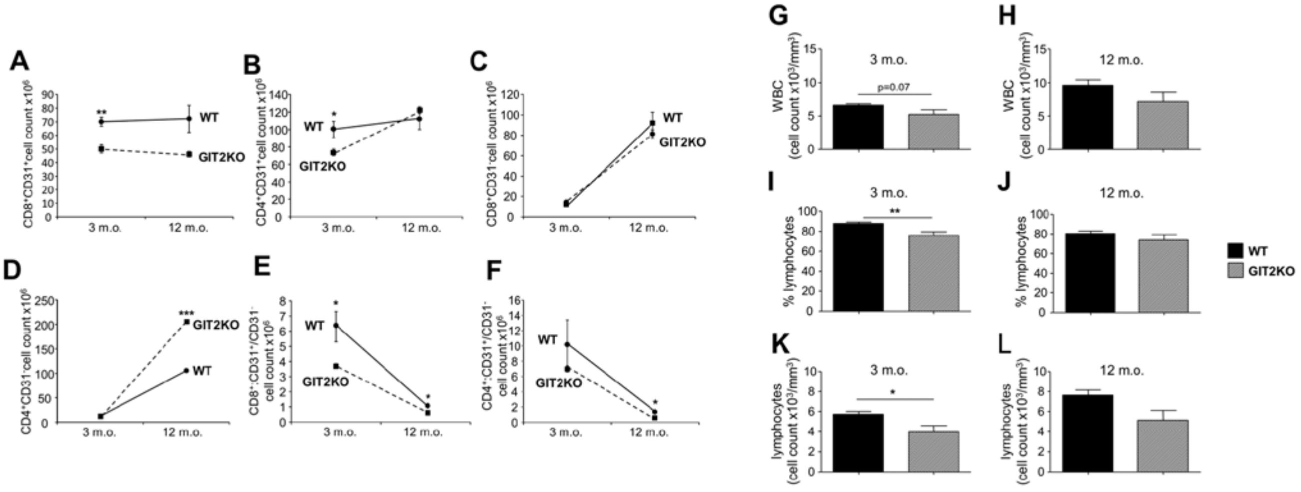

Fig S2

Figure. S2 GIT2 genomic depletion affects functional T cell development and circulating white cell status. WT and GIT2KO total cell counts for splenic $CD8^+CD31^+$ cells (**A**) and $CD4^+CD31^+$ cells (**B**). WT and GIT2KO total cell counts for splenic $CD8^+CD31^-$ cells (**C**) and $CD4^+CD31^-$ cells (**D**). $CD8^+$:$CD31^+/CD31^-$ cell count ratios in WT and GIT2KO spleen (**E**). $CD4^+$:$CD31^+/CD31^-$ cell count ratios in WT and $GIT2^{KO}$ spleen (**F**). Total white blood cell (WBC) counts for 3 month old WT and GIT2KO mice (**G**). (B) Total WBC counts for 12 month old WT and GIT2KO mice (**H**). (C) Total percentage lymphocyte measurements for 3 month old WT and GIT2KO mice (**I**). Total percentage lymphocyte measurements for 12 month old WT and GIT2KO mice (**J**). Total lymphocyte cell counts for 3 month old WT and GIT2KO mice (**K**). Total lymphocyte cell counts for 12 month old WT and GIT2KO mice (**L**). All values indicated are mean ± SEM. WT data are represented by black bars with GIT2KO data represented by lined bars. Months of age is abbreviated to m.o. *p<0.05, **p<0.01, ***p<0.001.

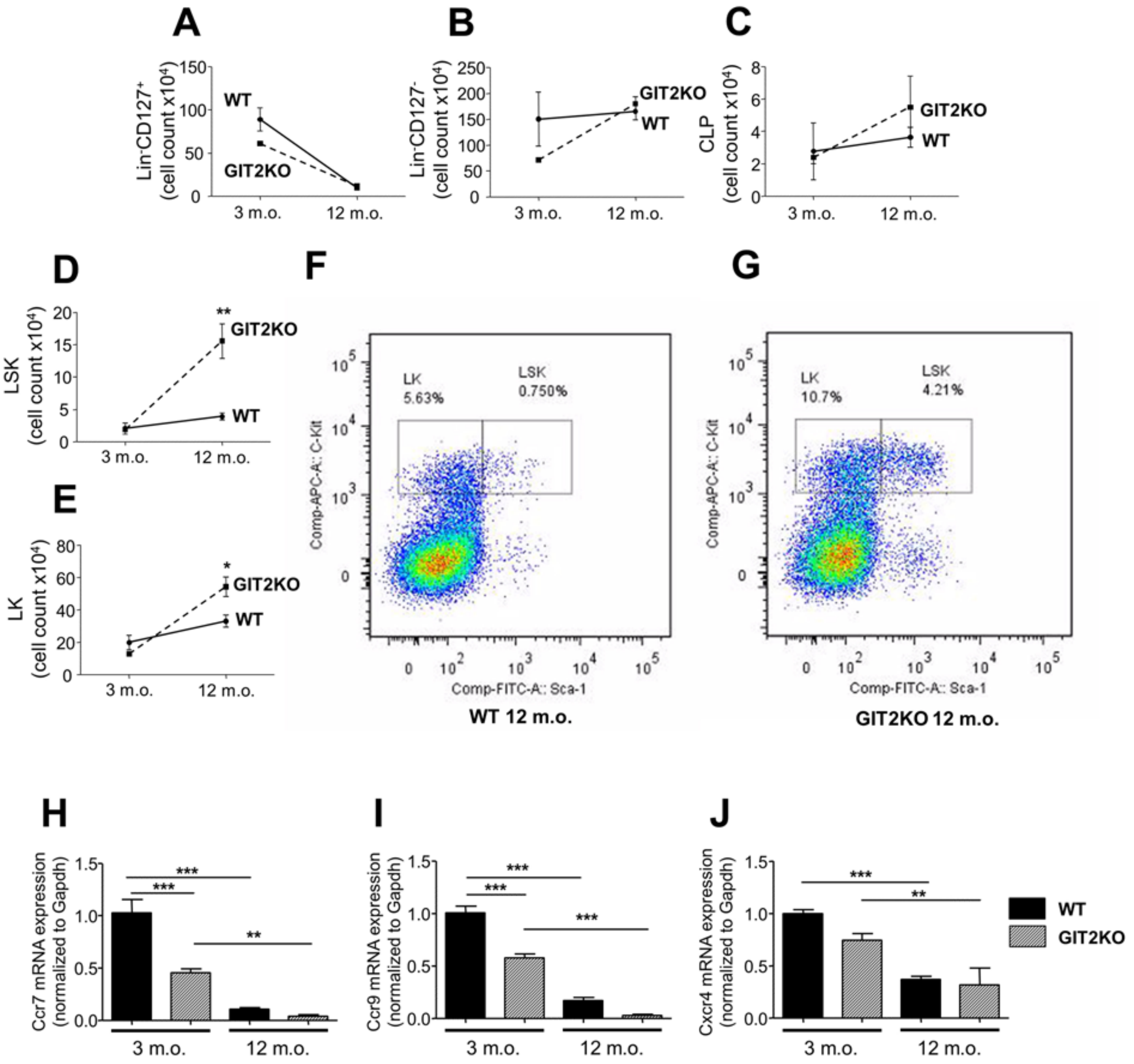

Figure S3 Hematopoietic precursors for T cell development in WT and GIT2KO mouse bone marrow. Cell counts for Lin$^-$CD127$^+$ (**A**), Lin$^-$CD127$^-$ (**B**) and CLP (common lymphoid progenitors: **C**) were not significantly different between WT and GIT2KO mice. LSK (Lin$^-$CD127$^-$Sca-1$^+$c-Kit$^+$: (**D**)) and LK (Lin$^-$CD127$^-$Sca-1$^-$c-Kit$^+$: (**E**)) were up-regulated in GIT2KO mice in an age-dependent manner. Representative FACS images are represented for WT (**F**) and GIT2KO (**G**) bone marrow extracts. In each panel (**F**-**G**) x-axes represents increasing Sca-1 and y-axes represent increasing c-Kit. Bone marrow transcript expression for CCR7 (**H**), CCR9 (**I**) or CXCR4 (**J**) in WT and GIT2KO mice. All values indicated are mean ± SEM. For line charts WT data is indicated with solid lines, GIT2KO data with dashed lines. For histograms, WT data are represented by black bars with GIT2KO data represented by lined bars. Months of age is abbreviated to m.o. *p<0.05, **p<0.01, ***p<0.001.

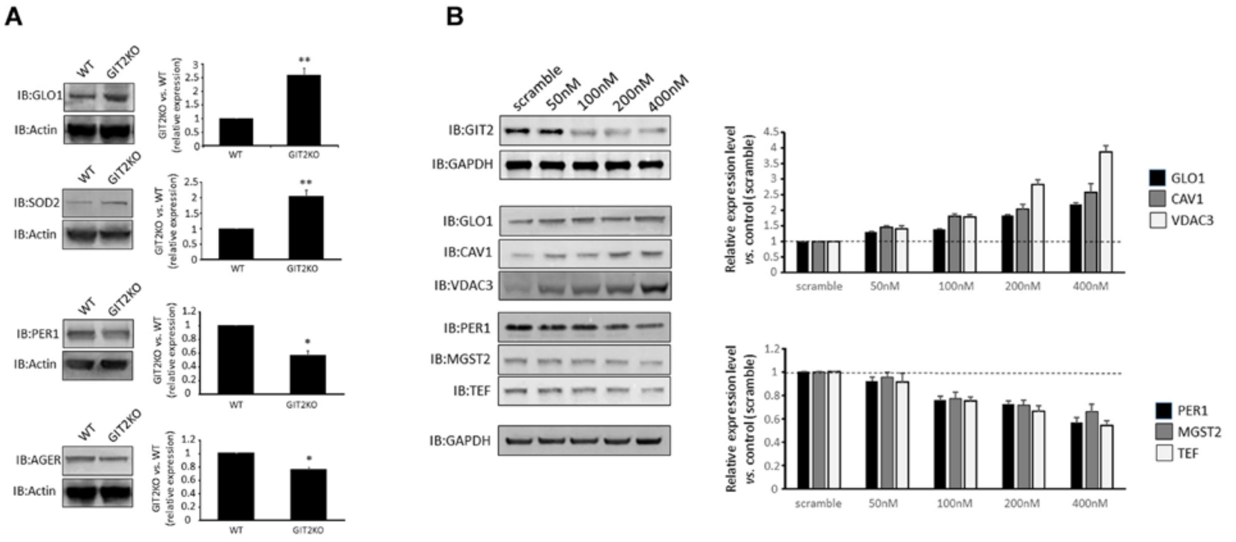

Figure S4. GIT2-dependent protein expression analysis reinforces transcriptomic microarray data. Protein expression data for both GIT2-dependent upregulation and downregulation (IB – immunoblot) in thymi from GIT2KO mice (**A**). For each representative blot quantification a quantitative histogram is depicted. Data represented is mean ± SEM (n=3). *p<0.05, **p<0.01. Endogenous GIT2 expression was attenuated in cultured Jurkat cells using a combination of GIT2 siRNA (50-400nM final concentration). Immunoblots for proteins suggested by transcriptomic microarray analysis were performed, for both upregulated and downregulated factors (**B**).

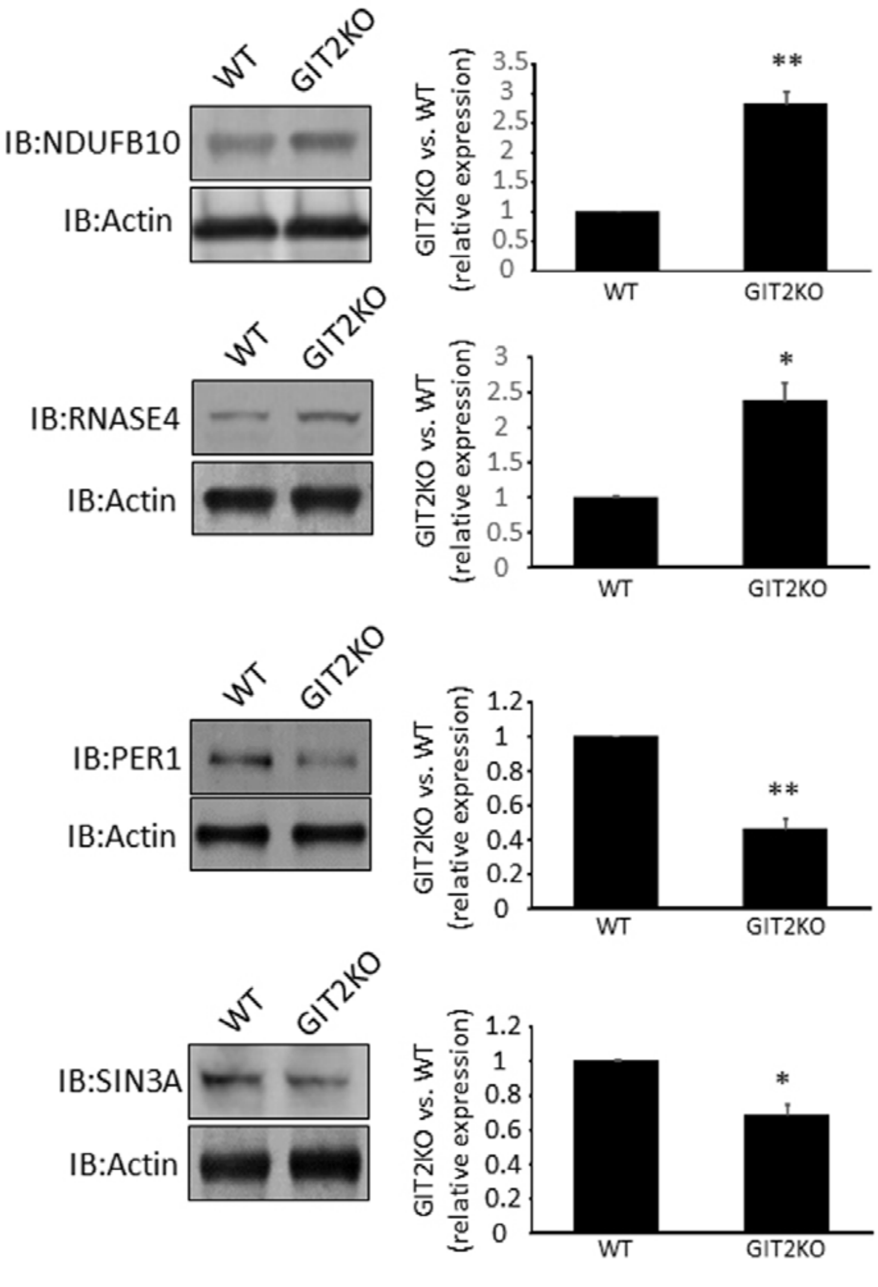

Figure S5. GIT2-dependent protein expression analysis reinforces transcriptomic microarray data in spleen. Protein expression data for both GIT2-dependent upregulation and downregulation (IB – immunoblot) in spleen from GIT2KO mice (**A**). For each representative blot quantification a quantitative histogram is depicted. Data represented is mean ± SEM (n=3). *p<0.05, **p<0.01.

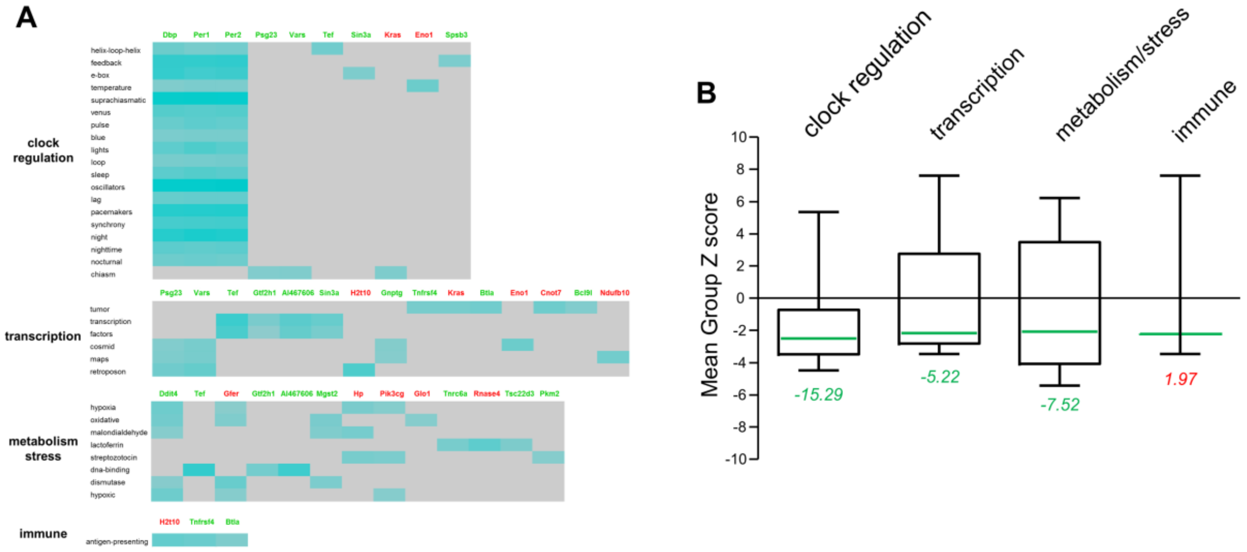

Figure S6. LSI-based analysis of GIT2KO-specific, cross-tissue immune regulatory factors. Individual semantic processing heatmaps of the strongest individual transcript-word associations generated using the input of the 40 transcripts coherently regulated in GIT2KO mice across ILN, MLN, spleen and thymus tissues. The strength of the latent semantic associations between input transcript identities (arranged in columns: upregulated in GIT2KO vs. WT are depicted in red; downregulated in GIT2KO vs. WT in green) and the extracted scientific words (arranged in rows) are indicated in a scale from grey (no correlation) to increasing densities of teal (strongest correlations) (**A**). The semantically-associated words were then clustered into functional groups: '*clock regulation*'; '*transcription*'; '*metabolism/stress*'; '*immune*'. For the four functional categories indicated in panel A – the mean geometric group Z score is indicated (mean ± 95% confidence limit range) in panel (**B**). In the histogram the mean Z score value is indicated by the solid green line and the number in italics associated with each column represents the cumulative group Z score value – calculated by the summation of the individual z ratios of the significantly-regulated transcripts linked with that specific group (green indicates a negative cumulative Z score, red indicates a positive cumulative Z score).

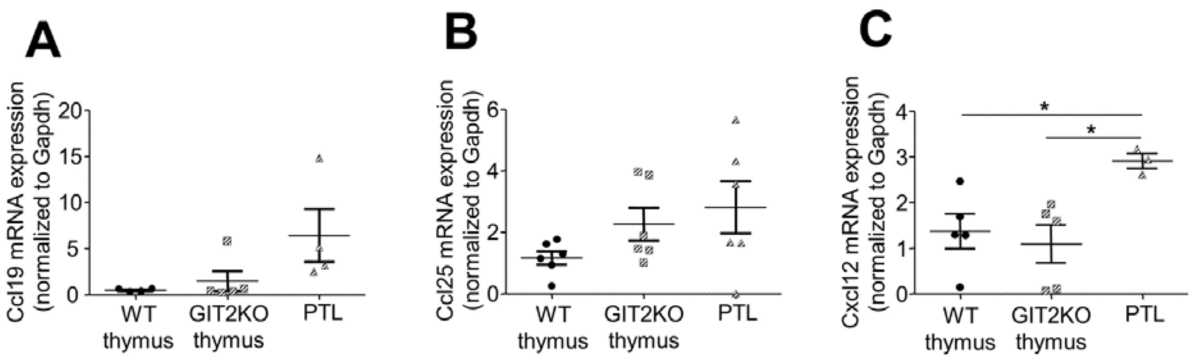

Figure S7 Chemokine ligand transcript expression patterns in GIT2KO parathymic lobes. Transcript expression for CCL19 (**A**), CCL25 (**B**) and CXCL12 (**C**) was measured in WT thymus, GIT2KO thymus and in GIT2KO PTLs. All values indicated are mean ± SEM. WT data are represented by solid black objects and GIT2KO data are represented by lined objects. *p<0.05, **p<0.01.

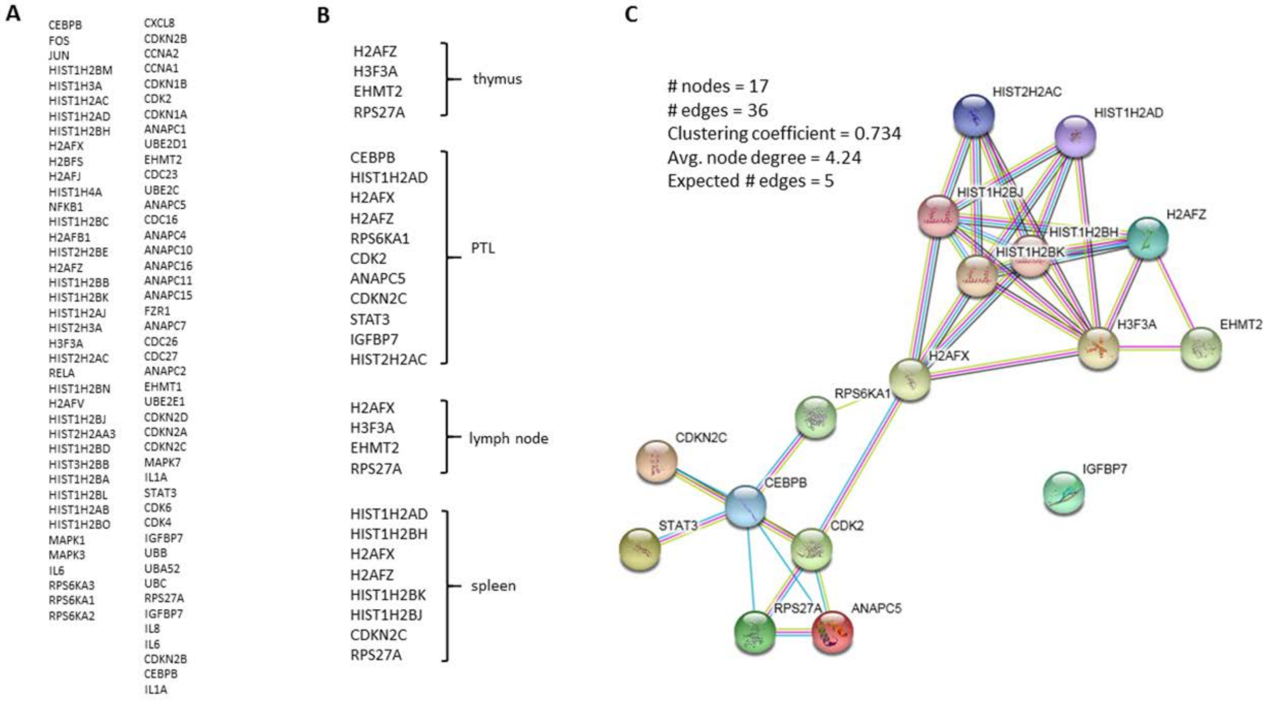

Figure S8. Functional overlap between the canonical senescence-associated secretory pathway (SASP) and the GIT2KO molecular phenotype. An unbiased SASP pathway data list was obtained from REACTOME (www.reactome.org) (**A**). Transcripts significantly altered in the respective tissues (thymus, PTL – parathymic lobe, lymph nodes, spleen) that are found in the canonical SASP list. Functional interaction network analysis (STRING – www. http://string-db.org/: high network confidence applied) using the 17 GIT2KO-altered SASP-overlapping transcripts demonstrates a strong interconnectivity between the GIT2KO and SASP phenotypes.

SUPPLEMENTARY MATERIALS AND METHODS

Western Blotting

Cell and tissue lysates were prepared using a standard RIPA buffer containing 0.1% SDS (Janssens et al., 2015). All protein extracts were quantified using BCA reagent (ThermoScientific, Rockford IL) and then normalized for each specific experiment before resolution with SDS-PAGE and semi-dry electrotransfer (Bio-Rad, Hercules CA) to PVDF membranes (PerkinElmer Life Sciences; Waltham MA). Membranes were blocked using a 4% bovine serum albumin for Western blot and primary antibody immune-reactive complexes were identified using alkaline phosphatase-conjugated secondary antisera (Sigma-Aldrich, St. Louis MO) with enzyme-linked chemifluorescence (GE Healthcare) as described previously (Martin et al., 2016). Primary antibodies specific for glyoxalase 1 (GLO1: 6F10: ab81461), superoxide dismutase 2 (SOD2: ab13533), period 1 (PER1: ab3443), receptor for advanced glycation endproducts (AGER: ab37647), RNase 4 (RNASE4: ab200717), microsomal glutathione S-transferase 2 (MGST2: ab208802) and thyrotrophic embryonic factor (TEF: ab48836) were obtained from Abcam (Cambridge MA). Primary antibodies specific for glyceraldehyde-3-phosphate dehydrogenase (GAPDH: I-19), caveolin 1 (CAV1: N-20) and voltage-dependent anion-selective channel protein 3 (VDAC3: H-40) were obtained from Santa Cruz Biotechnology (Santa Cruz CA). A primary antibody specific for Paired amphipathic helix protein Sin3a (SIN3A: NB600-1263) was obtained from Novus Biologicals (Littleton CO). A primary antibody specific for NADH dehydrogenase (ubiquinone) 1 beta subcomplex, 10 (NDUFB1010: 15589-1-AP) was obtained from, ProteinTech (Chicago IL). A primary antibodies specific for G protein-coupled receptor kinase-interactor 2 (GIT2: A302-102A) was obtained from Bethyl Laboratories Inc. (Montgomery TX).

Cellular RNA interference

Human Jurkat cells (obtained from ATCC: clone E6-1) were maintained at 37°C in a 5% CO_2 atmosphere in RPMI media (Sigma-Aldrich, St. Louis MO) supplemented with 10% fetal bovine serum (Sigma-Aldrich, St. Louis MOI) as recommended by ATCC (https://www.lgcstandards-atcc.org/en.aspx). Human GIT2 siRNA (Santa Cruz) was a pool of 3 target-specific 19-25 nt siRNAs. Control siRNA-A consists of a scrambled sequence that will not lead to the specific degradation of any cellular mRNA. Sequences of siRNAs: GIT2-A: Sense: CCAAUAAAGCGG AAUUCAU; Antisense: AUGAAUUCCGCUUUAUUGG. GIT2-B: Sense: GUACUCAUCAA CACG AAAU; Antisense: AUUUCGUGUUGAUGAGUAC. GIT2-C: Sense: GCGUUGAGAG UCAAGACAA; Antisense: UUGUCUUGACUCUCAACGC. Control siRNA-A: Sense: UUCU CCGAACGUGUCACGU; Antisense: CGUGACACGUUCGGAGAA. Jurkat cells were transfected with siRNA oligos using Lipofectamine RNAi MAX (Life Technologies, Carlsbad CA) according to the manufacturer's protocol.
