# Supplementary material for: Genomic deletion of GIT2 induces a premature age-related thymic dysfunction and systemic immune system disruption
Source: Aging (Albany NY). 2017 Mar 4;9(3):706–30. doi: 10.18632/aging.101185 (PMC5391227; doi:10.18632/aging.101185)
Supplement: Supplementary file 2 [file aging-09-706-s002.docx]

**Table S1. Transcripts significantly regulated differentially between the GIT2KO thymus and the WT thymus.** The official Gene Symbol, transcript description and associated Z ratios for the comparison of the GIT2KO thymus *vs.* WT thymus at the 12 month time point are indicated. Each transcript was significantly regulated at p<0.05, with a Z ratio >± 1.5.

| **Symbol** | **Description** | **Z ratio** |
| --- | --- | --- |
| H2-T10 | histocompatibility 2, T region locus 10 (H2-T10) | 10.36 |
| Ctse | cathepsin E (Ctse) | 9.86 |
| Hp | haptoglobin (Hp) | 8.67 |
| Hp | haptoglobin (Hp) | 8.61 |
| Hba-a1 | hemoglobin alpha, adult chain 1 (Hba-a1) | 8.33 |
| Hp | haptoglobin (Hp) | 8.29 |
| Scd1 | stearoyl-Coenzyme A desaturase 1 (Scd1) | 7.41 |
| LOC100043671 | hypothetical protein LOC100043671 (LOC100043671) | 5.98 |
| Lpl | lipoprotein lipase (Lpl) | 5.91 |
| Mgst1 | microsomal glutathione S-transferase 1 (Mgst1) | 5.76 |
| Rasl12 | RAS-like, family 12 (Rasl12) | 5.74 |
| Aoc3 | amine oxidase, copper containing 3 (Aoc3) | 5.57 |
| Adi1 | acireductone dioxygenase 1 (Adi1) | 5.38 |
| Cav1 | caveolin, caveolae protein 1 (Cav1) | 5.37 |
| Cbr3 | carbonyl reductase 3 (Cbr3) | 5.04 |
| Cdo1 | cysteine dioxygenase 1, cytosolic (Cdo1) | 5.02 |
| Hspa8 | heat shock protein 8 (Hspa8) | 4.89 |
| S100a8 | S100 calcium binding protein A8 (calgranulin A) (S100a8) | 4.7 |
| Ndg2 | Nur77 downstream gene 2 (Ndg2) | 4.59 |
| Pygl | liver glycogen phosphorylase (Pygl) | 4.39 |
| Ces3 | carboxylesterase 3 (Ces3) | 4.38 |
| Idh3g | isocitrate dehydrogenase 3 (NAD+), gamma (Idh3g), nuclear gene encoding mitochondrial protein | 4.32 |
| Eno1 | enolase 1, alpha non-neuron (Eno1) | 4.29 |
| Sparc | secreted acidic cysteine rich glycoprotein (Sparc) | 4.29 |
| Pnpla2 | patatin-like phospholipase domain containing 2 (Pnpla2) | 4.24 |
| Cd59a | CD59a antigen (Cd59a) | 4.23 |
| Dci | dodecenoyl-Coenzyme A delta isomerase (3,2 trans-enoyl-Coenyme A isomerase) (Dci), nuclear gene encoding mitochondrial protein | 4.21 |
| Kras | v-Ki-ras2 Kirsten rat sarcoma viral oncogene homolog (Kras) | 4.16 |
| LOC100044190 | hypothetical protein LOC100044190 (LOC100044190) | 4.13 |
| Fhl1 | four and a half LIM domains 1 (Fhl1), transcript variant 2 | 3.88 |
| Gng10 | guanine nucleotide binding protein (G protein), gamma 10 (Gng10) | 3.87 |
| S100a1 | S100 calcium binding protein A1 (S100a1) | 3.86 |
| Glo1 | glyoxalase 1 (Glo1) | 3.83 |
| Tmem66 | transmembrane protein 66 (Tmem66) | 3.73 |
| Dbi | diazepam binding inhibitor (Dbi), transcript variant 2 | 3.7 |
| H2-K1 | histocompatibility 2, K1, K region (H2-K1) | 3.68 |
| Fhl1 | four and a half LIM domains 1 (Fhl1), transcript variant 1 | 3.68 |
| Pdhb | pyruvate dehydrogenase (lipoamide) beta (Pdhb) | 3.67 |
| Sdpr | serum deprivation response (Sdpr) | 3.64 |
| Atp5f1 | ATP synthase, H+ transporting, mitochondrial F0 complex, subunit b, isoform 1 (Atp5f1) | 3.64 |
| Rarres2 | retinoic acid receptor responder (tazarotene induced) 2 (Rarres2) | 3.64 |
| Sparc | secreted acidic cysteine rich glycoprotein (Sparc) | 3.63 |
| Fmo1 | flavin containing monooxygenase 1 (Fmo1) | 3.62 |
| Ndn | necdin (Ndn) | 3.6 |
| Eif4ebp1 | eukaryotic translation initiation factor 4E binding protein 1 (Eif4ebp1) | 3.56 |
| Fh1 | fumarate hydratase 1 (Fh1) | 3.55 |
| Cpt2 | carnitine palmitoyltransferase 2 (Cpt2) | 3.55 |
| Gja1 | gap junction membrane channel protein alpha 1 (Gja1) | 3.53 |
| Ppp1ca | protein phosphatase 1, catalytic subunit, alpha isoform (Ppp1ca) | 3.51 |
| Pik3cg | phosphoinositide-3-kinase, catalytic, gamma polypeptide (Pik3cg) | 3.48 |
| Gstm1 | glutathione S-transferase, mu 1 (Gstm1) | 3.47 |
| EG433923 | predicted gene, EG433923 (EG433923) | 3.44 |
| Sdhb | succinate dehydrogenase complex, subunit B, iron sulfur (Ip) (Sdhb) | 3.42 |
| 1700037H04Rik | RIKEN cDNA 1700037H04 gene (1700037H04Rik) | 3.39 |
| Prelp | proline arginine-rich end leucine-rich repeat (Prelp) | 3.33 |
| Idh1 | isocitrate dehydrogenase 1 (NADP+), soluble (Idh1) | 3.33 |
| Psmb7 | proteasome (prosome, macropain) subunit, beta type 7 (Psmb7) | 3.32 |
| Loxl1 | lysyl oxidase-like 1 (Loxl1) | 3.28 |
| Ndufa12 | NADH dehydrogenase (ubiquinone) 1 alpha subcomplex, 12 (Ndufa12) | 3.26 |
| Ndufb6 | NADH dehydrogenase (ubiquinone) 1 beta subcomplex, 6 (Ndufb6), nuclear gene encoding mitochondrial protein | 3.24 |
| Sdcbp | syndecan binding protein (Sdcbp), transcript variant 1 | 3.22 |
| Caprin1 | cell cycle associated protein 1 (Caprin1) | 3.2 |
| Cxcl12 | chemokine (C-X-C motif) ligand 12 (Cxcl12), transcript variant 3 | 3.19 |
| LOC100047934 | hypothetical protein LOC100047934 (LOC100047934) | 3.19 |
| Ywhag | tyrosine 3-monooxygenase/tryptophan 5-monooxygenase activation protein, gamma polypeptide (Ywhag) | 3.18 |
| Smu1 | smu-1 suppressor of mec-8 and unc-52 homolog (C. elegans) (Smu1) | 3.15 |
| Sdhb | succinate dehydrogenase complex, subunit B, iron sulfur (Ip) (Sdhb) | 3.12 |
| Ndufc1 | NADH dehydrogenase (ubiquinone) 1, subcomplex unknown, 1 (Ndufc1) | 3.1 |
| Emp1 | epithelial membrane protein 1 (Emp1) | 3.09 |
| Pfkl | phosphofructokinase, liver, B-type (Pfkl) | 3.08 |
| Mgst3 | microsomal glutathione S-transferase 3 (Mgst3) | 3.07 |
| Sept15 | selenoprotein (Sep15) | 3.07 |
| Ptp4a2 | protein tyrosine phosphatase 4a2 (Ptp4a2) | 3.07 |
| Dbi | diazepam binding inhibitor (Dbi), transcript variant 2 | 3.07 |
| Ndufc1 | NADH dehydrogenase (ubiquinone) 1, subcomplex unknown, 1 (Ndufc1) | 3.04 |
| EG434858 | predicted gene, EG434858 (EG434858) on chromosome X. | 3.01 |
| Tmem33 | transmembrane protein 33 (Tmem33), transcript variant 2 | 2.99 |
| Txnl4a | thioredoxin-like 4A (Txnl4a), transcript variant 2 | 2.94 |
| Mrpl3 | mitochondrial ribosomal protein L3 (Mrpl3), nuclear gene encoding mitochondrial protein | 2.93 |
| Cxcl12 | chemokine (C-X-C motif) ligand 12 (Cxcl12), transcript variant 1 | 2.93 |
| Eif5a | eukaryotic translation initiation factor 5A (Eif5a) | 2.92 |
| Scp2 | sterol carrier protein 2, liver (Scp2) | 2.9 |
| Rnase4 | ribonuclease, RNase A family 4 (Rnase4), transcript variant 1 | 2.89 |
| Nola3 | nucleolar protein family A, member 3 (Nola3) | 2.89 |
| Scp2 | sterol carrier protein 2, liver (Scp2) | 2.88 |
| Hnrpk | heterogeneous nuclear ribonucleoprotein K (Hnrpk) | 2.87 |
| Cyc1 | cytochrome c-1 (Cyc1) | 2.85 |
| Sfrs5 | splicing factor, arginine/serine-rich 5 (SRp40, HRS) (Sfrs5), transcript variant 2 | 2.8 |
| Tspan3 | tetraspanin 3 (Tspan3) | 2.8 |
| Ndufa8 | NADH dehydrogenase (ubiquinone) 1 alpha subcomplex, 8 (Ndufa8), nuclear gene encoding mitochondrial protein | 2.78 |
| Cox6a1 | cytochrome c oxidase subunit VIa polypeptide 1 (Cox6a1) | 2.77 |
| Vdac3 | voltage-dependent anion channel 3 (Vdac3) | 2.74 |
| Dcn | decorin (Dcn) | 2.73 |
| Hnrnpa2b1 | heterogeneous nuclear ribonucleoprotein A2/B1 (Hnrnpa2b1), transcript variant 2 | 2.7 |
| Etfa | electron transferring flavoprotein, alpha polypeptide (Etfa), nuclear gene encoding mitochondrial protein | 2.7 |
| Supt4h2 | suppressor of Ty 4 homolog 2 (S. cerevisiae) (Supt4h2) | 2.69 |
| Adk | adenosine kinase (Adk) | 2.68 |
| Cd151 | CD151 antigen (Cd151) | 2.68 |
| Pno1 | partner of NOB1 homolog (S. cerevisiae) (Pno1) | 2.67 |
| Pdha1 | pyruvate dehydrogenase E1 alpha 1 (Pdha1), nuclear gene encoding mitochondrial protein | 2.66 |
| Cdr2 | cerebellar degeneration-related 2 (Cdr2) | 2.65 |
| Nola2 | nucleolar protein family A, member 2 (Nola2) | 2.65 |
| Msn | moesin (Msn) | 2.64 |
| Ube2g1 | ubiquitin-conjugating enzyme E2G 1 (UBC7 homolog, C. elegans) (Ube2g1) | 2.63 |
| Slc25a1 | solute carrier family 25 (mitochondrial carrier, citrate transporter), member 1 (Slc25a1), nuclear gene encoding mitochondrial protein | 2.63 |
| Gnas | GNAS (guanine nucleotide binding protein, alpha stimulating) complex locus (Gnas), transcript variant 3 | 2.63 |
| Tram1 | translocating chain-associating membrane protein 1 (Tram1) | 2.61 |
| Aebp1 | AE binding protein 1 (Aebp1) | 2.6 |
| Mcm6 | minichromosome maintenance deficient 6 (MIS5 homolog, S. pombe) (S. cerevisiae) (Mcm6) | 2.6 |
| Psmb7 | proteasome (prosome, macropain) subunit, beta type 7 (Psmb7) | 2.59 |
| Pfn1 | profilin 1 (Pfn1) | 2.59 |
| Acly | ATP citrate lyase (Acly) | 2.59 |
| Gstk1 | glutathione S-transferase kappa 1 (Gstk1) | 2.59 |
| Hadhb | hydroxyacyl-Coenzyme A dehydrogenase/3-ketoacyl-Coenzyme A thiolase/enoyl-Coenzyme A hydratase (trifunctional protein), beta subunit (Hadhb), nuclear gene encoding mitochondrial protein | 2.58 |
| Naca | nascent polypeptide-associated complex alpha polypeptide (Naca) | 2.57 |
| Sypl | synaptophysin-like protein (Sypl), transcript variant 1 | 2.56 |
| Gfer | growth factor, erv1 (S. cerevisiae)-like (augmenter of liver regeneration) (Gfer) | 2.55 |
| Cox6b1 | cytochrome c oxidase, subunit VIb polypeptide 1 (Cox6b1) | 2.54 |
| Gpd2 | glycerol phosphate dehydrogenase 2, mitochondrial (Gpd2), nuclear gene encoding mitochondrial protein | 2.53 |
| Spcs1 | signal peptidase complex subunit 1 homolog (S. cerevisiae) (Spcs1) | 2.53 |
| Suclg1 | succinate-CoA ligase, GDP-forming, alpha subunit (Suclg1) | 2.51 |
| Sqle | squalene epoxidase (Sqle) | 2.51 |
| Nme2 | non-metastatic cells 2, protein (NM23B) expressed in (Nme2), transcript variant 1 | 2.5 |
| Acss2 | acyl-CoA synthetase short-chain family member 2 (Acss2) | 2.5 |
| Isca1 | iron-sulfur cluster assembly 1 homolog (S. cerevisiae) (Isca1) | 2.48 |
| Tmem33 | transmembrane protein 33 (Tmem33), transcript variant 1 | 2.47 |
| Tspo | translocator protein (Tspo) | 2.46 |
| Naca | nascent polypeptide-associated complex alpha polypeptide (Naca) | 2.46 |
| Nudt4 | nudix (nucleoside diphosphate linked moiety X)-type motif 4 (Nudt4) | 2.46 |
| Psmd7 | proteasome (prosome, macropain) 26S subunit, non-ATPase, 7 (Psmd7) | 2.45 |
| Etfa | electron transferring flavoprotein, alpha polypeptide (Etfa), nuclear gene encoding mitochondrial protein | 2.45 |
| Acadm | acyl-Coenzyme A dehydrogenase, medium chain (Acadm) | 2.44 |
| Ndufb10 | NADH dehydrogenase (ubiquinone) 1 beta subcomplex, 10 (Ndufb10) | 2.44 |
| H3f3a | H3 histone, family 3A (H3f3a) | 2.42 |
| Bola3 | bolA-like 3 (E. coli) (Bola3) | 2.42 |
| Sod2 | superoxide dismutase 2 (Sod2) | 2.41 |
| LOC100047353 | PREDICTED: similar to myocardial vascular inhibition factor (LOC100047353) | 2.41 |
| Lamp2 | lysosomal-associated membrane protein 2 (Lamp2), transcript variant 1 | 2.41 |
| Hsd17b12 | hydroxysteroid (17-beta) dehydrogenase 12 (Hsd17b12) | 2.4 |
| Mmd | monocyte to macrophage differentiation-associated (Mmd) | 2.39 |
| Dcps | decapping enzyme, scavenger (Dcps) | 2.38 |
| Pnrc2 | proline-rich nuclear receptor coactivator 2 (Pnrc2) | 2.37 |
| Mgll | monoglyceride lipase (Mgll) | 2.36 |
| Cox5a | cytochrome c oxidase, subunit Va (Cox5a), nuclear gene encoding mitochondrial protein | 2.34 |
| Usp39 | ubiquitin specific peptidase 39 (Usp39) | 2.33 |
| Rnf11 | ring finger protein 11 (Rnf11) | 2.32 |
| Mylc2b | myosin light chain, regulatory B (Mylc2b) | 2.31 |
| Ndufa4 | NADH dehydrogenase (ubiquinone) 1 alpha subcomplex, 4 (Ndufa4) | 2.31 |
| 1810035L17Rik | RIKEN cDNA 1810035L17 gene (1810035L17Rik) | 2.29 |
| Bxdc2 | brix domain containing 2 (Bxdc2) | 2.28 |
| 1110001J03Rik | RIKEN cDNA 1110001J03 gene (1110001J03Rik) | 2.28 |
| Aacs | acetoacetyl-CoA synthetase (Aacs) | 2.28 |
| Zfp207 | zinc finger protein 207 (Zfp207) | 2.28 |
| LOC654426 | ATP synthase, H+ transporting, mitochondrial F0 complex, subunit F pseudogene (LOC654426) on chromosome 14. | 2.28 |
| Ednrb | endothelin receptor type B (Ednrb) | 2.27 |
| Gpx4 | glutathione peroxidase 4 (Gpx4), transcript variant 1 | 2.26 |
| Eef2 | eukaryotic translation elongation factor 2 (Eef2) | 2.26 |
| Eef2 | eukaryotic translation elongation factor 2 (Eef2) | 2.25 |
| Tuba1a | tubulin, alpha 1A (Tuba1a) | 2.24 |
| Drg1 | developmentally regulated GTP binding protein 1 (Drg1) | 2.23 |
| Echs1 | enoyl Coenzyme A hydratase, short chain, 1, mitochondrial (Echs1) | 2.23 |
| Cox6a1 | cytochrome c oxidase, subunit VI a, polypeptide 1 (Cox6a1), nuclear gene encoding mitochondrial protein | 2.22 |
| Sdhc | succinate dehydrogenase complex, subunit C, integral membrane protein (Sdhc), nuclear gene encoding mitochondrial protein | 2.22 |
| Ppa1 | pyrophosphatase (inorganic) 1 (Ppa1) | 2.21 |
| Coro1a | coronin, actin binding protein 1A (Coro1a) | 2.21 |
| Rnaset2 | ribonuclease T2 (Rnaset2), transcript variant 2 | 2.21 |
| Stx8 | syntaxin 8 (Stx8) | 2.21 |
| Eif3s10 | eukaryotic translation initiation factor 3, subunit 10 (theta) (Eif3s10) | 2.21 |
| Oxct1 | 3-oxoacid CoA transferase 1 (Oxct1) | 2.2 |
| Fkbp2 | FK506 binding protein 2 (Fkbp2) | 2.2 |
| Rnf11 | ring finger protein 11 (Rnf11) | 2.2 |
| Dph3 | DPH3 homolog (KTI11, S. cerevisiae) (Dph3), transcript variant 1 | 2.2 |
| Mid1ip1 | Mid1 interacting protein 1 (gastrulation specific G12-like (zebrafish)) (Mid1ip1) | 2.18 |
| Timm8b | translocase of inner mitochondrial membrane 8 homolog b (yeast) (Timm8b) | 2.17 |
| LOC100048480 | similar to ubiquinol-cytochrome c reductase binding protein (LOC100048480) | 2.17 |
| Prdx3 | peroxiredoxin 3 (Prdx3) | 2.16 |
| Ndufa9 | NADH dehydrogenase (ubiquinone) 1 alpha subcomplex, 9 (Ndufa9) | 2.16 |
| Gstp1 | glutathione S-transferase, pi 1 (Gstp1) | 2.15 |
| Txnl4 | thioredoxin-like 4A (Txnl4) | 2.15 |
| Rps27a | ribosomal protein S27a (Rps27a), transcript variant 2 | 2.15 |
| Etfa | electron transferring flavoprotein, alpha polypeptide (Etfa), nuclear gene encoding mitochondrial protein | 2.15 |
| Hnrpa1 | heterogeneous nuclear ribonucleoprotein A1 (Hnrpa1), transcript variant 2 | 2.15 |
| LOC100047155 | similar to Small nuclear ribonucleoprotein polypeptide A (LOC100047155) | 2.14 |
| Arhgap29 | Rho GTPase activating protein 29 (Arhgap29) | 2.12 |
| Nudt5 | nudix (nucleoside diphosphate linked moiety X)-type motif 5 (Nudt5) | 2.12 |
| Brp17 | paroxysmal nonkinesiogenic dyskinesia (Brp17) | 2.11 |
| Mylc2b | myosin light chain, regulatory B (Mylc2b) | 2.1 |
| Eef2 | eukaryotic translation elongation factor 2 (Eef2) | 2.1 |
| Serpinf1 | serine (or cysteine) peptidase inhibitor, clade F, member 1 (Serpinf1) | 2.1 |
| Dhx15 | DEAH (Asp-Glu-Ala-His) box polypeptide 15 (Dhx15), transcript variant 1 | 2.1 |
| Aldoa | aldolase 1, A isoform (Aldoa) | 2.1 |
| Tmem38b | transmembrane protein 38B (Tmem38b) | 2.08 |
| Nap1l1 | nucleosome assembly protein 1-like 1 (Nap1l1) | 2.08 |
| Tmem14c | transmembrane protein 14C (Tmem14c) | 2.07 |
| Pmp22 | peripheral myelin protein (Pmp22) | 2.07 |
| Eif5 | eukaryotic translation initiation factor 5 (Eif5), transcript variant 2 | 2.06 |
| Igfbp4 | insulin-like growth factor binding protein 4 (Igfbp4) | 2.04 |
| 1500032D16Rik | RIKEN cDNA 1500032D16 gene (1500032D16Rik) | 2.04 |
| Gabarap | gamma-aminobutyric acid receptor associated protein (Gabarap) | 2.04 |
| Fdps | farnesyl diphosphate synthetase (Fdps) | 2.04 |
| Pcyox1 | prenylcysteine oxidase 1 (Pcyox1) | 2.03 |
| Hsp90ab1 | heat shock protein 90kDa alpha (cytosolic), class B member 1 (Hsp90ab1) | 2.03 |
| MGC18837 | transmembrane protein 205 (Tmem205) | 2.02 |
| 2610029G23Rik | RIKEN cDNA 2610029G23 gene (2610029G23Rik) | 2.02 |
| Chmp1b | chromatin modifying protein 1B (Chmp1b) | 2.01 |
| Tspo | translocator protein (Tspo) | 2.01 |
| Eif5 | eukaryotic translation initiation factor 5 (Eif5), transcript variant 1 | 2.01 |
| 9430029K10Rik | Yip1 interacting factor homolog B (S. cerevisiae) (Yif1b) | 2 |
| Ifitm2 | interferon induced transmembrane protein 2 (Ifitm2) | 2 |
| Smarce1 | SWI/SNF related, matrix associated, actin dependent regulator of chromatin, subfamily e, member 1 (Smarce1) | 2 |
| Fundc1 | FUN14 domain containing 1 (Fundc1) | 2 |
| Snap23 | synaptosomal-associated protein 23 (Snap23) | 1.99 |
| Ndufa5 | NADH dehydrogenase (ubiquinone) 1 alpha subcomplex, 5 (Ndufa5), nuclear gene encoding mitochondrial protein | 1.99 |
| B230219D22Rik | RIKEN cDNA B230219D22 gene (B230219D22Rik) | 1.99 |
| Ndufs4 | NADH dehydrogenase (ubiquinone) Fe-S protein 4 (Ndufs4), nuclear gene encoding mitochondrial protein | 1.99 |
| Slc25a5 | solute carrier family 25 (mitochondrial carrier, adenine nucleotide translocator), member 5 (Slc25a5), nuclear gene encoding mitochondrial protein | 1.96 |
| Rpa3 | replication protein A3 (Rpa3) | 1.96 |
| Cs | citrate synthase (Cs), nuclear gene encoding mitochondrial protein | 1.95 |
| Tgfbr2 | transforming growth factor, beta receptor II (Tgfbr2), transcript variant 1 | 1.94 |
| Vim | vimentin (Vim) | 1.94 |
| Hsp90ab1 | heat shock protein 90kDa alpha (cytosolic), class B member 1 (Hsp90ab1) | 1.94 |
| Tipin | timeless interacting protein (Tipin) | 1.93 |
| Commd1 | COMM domain containing 1 (Commd1) | 1.93 |
| Snrpd1 | small nuclear ribonucleoprotein D1 (Snrpd1) | 1.93 |
| H2afz | H2A histone family, member Z (H2afz) | 1.92 |
| Rb1 | retinoblastoma 1 (Rb1) | 1.92 |
| Mdh2 | malate dehydrogenase 2, NAD (mitochondrial) (Mdh2), nuclear gene encoding mitochondrial protein | 1.92 |
| D10Ertd322e | DNA segment, Chr 10, ERATO Doi 322, expressed (D10Ertd322e) | 1.92 |
| Zeb1 | zinc finger E-box binding homeobox 1 (Zeb1) | 1.91 |
| Capns1 | calpain, small subunit 1 (Capns1) | 1.9 |
| Hspa9 | heat shock protein 9 (Hspa9) | 1.9 |
| Rps6 | ribosomal protein S6 (Rps6) | 1.89 |
| Coq5 | coenzyme Q5 homolog, methyltransferase (yeast) (Coq5) | 1.88 |
| Prkaca | protein kinase, cAMP dependent, catalytic, alpha (Prkaca) | 1.88 |
| Pcbp1 | poly(rC) binding protein 1 (Pcbp1) | 1.85 |
| Snrpd1 | small nuclear ribonucleoprotein D1 (Snrpd1) | 1.85 |
| Mrps33 | mitochondrial ribosomal protein S33 (Mrps33), nuclear gene encoding mitochondrial protein, transcript variant 1 | 1.85 |
| Arl6ip5 | ADP-ribosylation factor-like 6 interacting protein 5 (Arl6ip5) | 1.84 |
| 1110008P14Rik | RIKEN cDNA 1110008P14 gene (1110008P14Rik) | 1.84 |
| Gpsn2 | glycoprotein, synaptic 2 (Gpsn2) | 1.83 |
| 1200003C05Rik | RIKEN cDNA 1200003C05 gene (1200003C05Rik) | 1.82 |
| Atp5l | ATP synthase, H+ transporting, mitochondrial F0 complex, subunit g (Atp5l), nuclear gene encoding mitochondrial protein | 1.82 |
| Mthfd1 | methylenetetrahydrofolate dehydrogenase (NADP+ dependent), methenyltetrahydrofolate cyclohydrolase, formyltetrahydrofolate synthase (Mthfd1) | 1.82 |
| 5730437N04Rik | RIKEN cDNA 5730437N04 gene (5730437N04Rik) | 1.82 |
| Rnf167 | ring finger protein 167 (Rnf167) | 1.81 |
| Ppm1a | protein phosphatase 1A, magnesium dependent, alpha isoform (Ppm1a) | 1.8 |
| Col4a1 | procollagen, type IV, alpha 1 (Col4a1) | 1.8 |
| Cd151 | CD151 antigen (Cd151) | 1.79 |
| Hsd11b1 | hydroxysteroid 11-beta dehydrogenase 1 (Hsd11b1), transcript variant 1 | 1.79 |
| 2610204L23Rik | coiled-coil domain containing 47 (Ccdc47) | 1.78 |
| Hmgb2 | high mobility group box 2 (Hmgb2) | 1.78 |
| Hmox2 | heme oxygenase (decycling) 2 (Hmox2) | 1.78 |
| Lsm2 | LSM2 homolog, U6 small nuclear RNA associated (S. cerevisiae) (Lsm2) | 1.78 |
| Timm8b | translocase of inner mitochondrial membrane 8 homolog b (yeast) (Timm8b) | 1.77 |
| Gpx1 | glutathione peroxidase 1 (Gpx1) | 1.77 |
| Yif1a | Yip1 interacting factor homolog A (S. cerevisiae) (Yif1a) | 1.77 |
| Tcp1 | t-complex protein 1 (Tcp1) | 1.76 |
| Nme2 | non-metastatic cells 2, protein (NM23B) expressed in (Nme2), transcript variant 1 | 1.76 |
| Ndufs2 | NADH dehydrogenase (ubiquinone) Fe-S protein 2 (Ndufs2) | 1.75 |
| LOC100048613 | similar to cytochrome c oxidase, subunit VIIc, transcript variant 1 (LOC100048613) | 1.74 |
| Mrps18b | mitochondrial ribosomal protein S18B (Mrps18b), nuclear gene encoding mitochondrial protein | 1.74 |
| Hbp1 | high mobility group box transcription factor 1 (Hbp1), transcript variant 1 | 1.74 |
| Smarce1 | SWI/SNF related, matrix associated, actin dependent regulator of chromatin, subfamily e, member 1 (Smarce1) | 1.73 |
| Arpc1a | actin related protein 2/3 complex, subunit 1A (Arpc1a) | 1.73 |
| Sec61b | Sec61 beta subunit (Sec61b) | 1.73 |
| Vps29 | vacuolar protein sorting 29 (S. pombe) (Vps29) | 1.73 |
| 2310016E02Rik | RIKEN cDNA 2310016E02 gene (2310016E02Rik) | 1.72 |
| Prkaca | protein kinase, cAMP dependent, catalytic, alpha (Prkaca) | 1.72 |
| Csrp2 | cysteine and glycine-rich protein 2 (Csrp2) | 1.72 |
| Tomm7 | translocase of outer mitochondrial membrane 7 homolog (yeast) (Tomm7) | 1.71 |
| Ghitm | growth hormone inducible transmembrane protein (Ghitm) | 1.71 |
| Arpc1a | actin related protein 2/3 complex, subunit 1A (Arpc1a) | 1.71 |
| Atp5c1 | ATP synthase, H+ transporting, mitochondrial F1 complex, gamma polypeptide 1 (Atp5c1) | 1.71 |
| LOC100046393 | similar to Protein phosphatase 2, regulatory subunit B (B56), alpha (LOC100046393) | 1.7 |
| Txnl4a | thioredoxin-like 4A (Txnl4a), transcript variant 1 | 1.7 |
| Arpc5 | actin related protein 2/3 complex, subunit 5 (Arpc5) | 1.69 |
| Tank | TRAF family member-associated Nf-kappa B activator (Tank) | 1.69 |
| Ppp2r1a | protein phosphatase 2 (formerly 2A), regulatory subunit A (PR 65), alpha isoform (Ppp2r1a) | 1.69 |
| LOC100046855 | similar to BKLF (LOC100046855) | 1.69 |
| Mcm5 | minichromosome maintenance deficient 5, cell division cycle 46 (S. cerevisiae) (Mcm5) | 1.69 |
| Gnb1 | guanine nucleotide binding protein (G protein), beta 1 (Gnb1) | 1.68 |
| EG622339 | predicted gene, EG622339 (EG622339) | 1.68 |
| l7Rn6 | lethal, Chr 7, Rinchik 6 (l7Rn6) | 1.68 |
| Tmem50b | transmembrane protein 50B (Tmem50b) | 1.67 |
| Tmem59 | transmembrane protein 59 (Tmem59) | 1.66 |
| Slc9a3r2 | solute carrier family 9 (sodium/hydrogen exchanger), member 3 regulator 2 (Slc9a3r2), transcript variant A | 1.66 |
| 2010316F05Rik | RIKEN cDNA 2010316F05 gene (2010316F05Rik) | 1.66 |
| Hsd11b1 | hydroxysteroid 11-beta dehydrogenase 1 (Hsd11b1), transcript variant 1 | 1.65 |
| Lin54 | lin-54 homolog (C. elegans) (Lin54) | 1.65 |
| Mrpl30 | mitochondrial ribosomal protein L30 (Mrpl30), nuclear gene encoding mitochondrial protein | 1.65 |
| Adk | adenosine kinase (Adk) | 1.65 |
| Spcs1 | signal peptidase complex subunit 1 homolog (S. cerevisiae) (Spcs1) | 1.64 |
| Magoh | mago-nashi homolog, proliferation-associated (Drosophila) (Magoh) | 1.64 |
| Dynll1 | dynein light chain LC8-type 1 (Dynll1) | 1.64 |
| Ube2k | ubiquitin-conjugating enzyme E2K (UBC1 homolog, yeast) (Ube2k) | 1.64 |
| Igfbp4 | insulin-like growth factor binding protein 4 (Igfbp4) | 1.63 |
| Phlda1 | pleckstrin homology-like domain, family A, member 1 (Phlda1) | 1.63 |
| Lgals1 | lectin, galactose binding, soluble 1 (Lgals1) | 1.63 |
| LOC668492 | similar to zinc finger CCHC-type and RNA binding motif 1 (LOC668492), misc RNA. | 1.62 |
| Atp5j | ATP synthase, H+ transporting, mitochondrial F0 complex, subunit F (Atp5j), nuclear gene encoding mitochondrial protein | 1.61 |
| Hsd17b4 | hydroxysteroid (17-beta) dehydrogenase 4 (Hsd17b4) | 1.61 |
| Hprt1 | hypoxanthine guanine phosphoribosyl transferase 1 (Hprt1) | 1.6 |
| Ahsa1 | AHA1, activator of heat shock protein ATPase homolog 1 (yeast) (Ahsa1) | 1.6 |
| Ndufa8 | NADH dehydrogenase (ubiquinone) 1 alpha subcomplex, 8 (Ndufa8) | 1.6 |
| Csde1 | cold shock domain containing E1, RNA binding (Csde1) | 1.59 |
| Snrpb | small nuclear ribonucleoprotein B (Snrpb) | 1.59 |
| Ormdl3 | ORM1-like 3 (S. cerevisiae) (Ormdl3) | 1.59 |
| Vkorc1 | vitamin K epoxide reductase complex, subunit 1 (Vkorc1) | 1.58 |
| Ak3 | adenylate kinase 3 (Ak3) | 1.58 |
| Rab35 | RAB35, member RAS oncogene family (Rab35) | 1.58 |
| Scoc | short coiled-coil protein (Scoc), transcript variant 2 | 1.58 |
| Cyb5r4 | cytochrome b5 reductase 4 (Cyb5r4) | 1.57 |
| Tmem126a | transmembrane protein 126A (Tmem126a) | 1.57 |
| Ccdc47 | coiled-coil domain containing 47 (Ccdc47) | 1.56 |
| Use1 | unconventional SNARE in the ER 1 homolog (S. cerevisiae) (Use1), transcript variant 1 | 1.55 |
| Nme3 | non-metastatic cells 3, protein expressed in (Nme3) | 1.54 |
| LOC100045617 | similar to Eukaryotic translation initiation factor 4A2 (LOC100045617) | 1.54 |
| Cops6 | COP9 (constitutive photomorphogenic) homolog, subunit 6 (Arabidopsis thaliana) (Cops6) | 1.54 |
| Rnps1 | ribonucleic acid binding protein S1 (Rnps1), transcript variant 2 | 1.54 |
| Ddx47 | DEAD (Asp-Glu-Ala-Asp) box polypeptide 47 (Ddx47) | 1.54 |
| Rps2 | ribosomal protein S2 (Rps2) | 1.54 |
| Plekhf2 | pleckstrin homology domain containing, family F (with FYVE domain) member 2 (Plekhf2) | 1.54 |
| Sqstm1 | sequestosome 1 (Sqstm1) | 1.53 |
| Cnot7 | CCR4-NOT transcription complex, subunit 7 (Cnot7) | 1.53 |
| Prkcdbp | protein kinase C, delta binding protein (Prkcdbp) | 1.53 |
| Atf4 | activating transcription factor 4 (Atf4) | 1.53 |
| Atp6v0e | ATPase, H+ transporting, lysosomal V0 subunit E (Atp6v0e) | 1.53 |
| Fkbp4 | FK506 binding protein 4 (Fkbp4) | 1.52 |
| Orc6l | origin recognition complex, subunit 6-like (S. cerevisiae) (Orc6l) | 1.52 |
| Inppl1 | inositol polyphosphate phosphatase-like 1 (Inppl1) | 1.51 |
| Hsd11b1 | hydroxysteroid 11-beta dehydrogenase 1 (Hsd11b1), transcript variant 1 | 1.51 |
| Tufm | Tu translation elongation factor, mitochondrial (Tufm), nuclear gene encoding mitochondrial protein | 1.51 |
| Ebp | phenylalkylamine Ca2+ antagonist (emopamil) binding protein (Ebp) | 1.51 |
| Prf1 | perforin 1 (pore forming protein) (Prf1) | -1.5 |
| Nfatc3 | nuclear factor of activated T-cells, cytoplasmic, calcineurin-dependent 3 (Nfatc3) | -1.51 |
| Mecp2 | methyl CpG binding protein 2 (Mecp2), transcript variant 1 | -1.51 |
| 5133401N09Rik | RIKEN cDNA 5133401N09 gene (5133401N09Rik), transcript variant 1 | -1.51 |
| Sertad2 | SERTA domain containing 2 (Sertad2), transcript variant 1 | -1.51 |
| Pigo | phosphatidylinositol glycan anchor biosynthesis, class O (Pigo) | -1.52 |
| Pstpip1 | proline-serine-threonine phosphatase-interacting protein 1 (Pstpip1) | -1.52 |
| Lias | lipoic acid synthetase (Lias) | -1.52 |
| Unc84b | unc-84 homolog B (C. elegans) (Unc84b) | -1.52 |
| Akap8l | A kinase (PRKA) anchor protein 8-like (Akap8l) | -1.52 |
| Hdgfrp2 | hepatoma-derived growth factor, related protein 2 (Hdgfrp2) | -1.52 |
| Atg16l1 | autophagy-related 16-like 1 (yeast) (Atg16l1), transcript variant b | -1.52 |
| Dctn5 | dynactin 5 (Dctn5) | -1.53 |
| 1810008A18Rik | RIKEN cDNA 1810008A18 gene (1810008A18Rik) | -1.53 |
| Igtp | interferon gamma induced GTPase (Igtp) | -1.53 |
| Prcp | prolylcarboxypeptidase (angiotensinase C) (Prcp) | -1.53 |
| Tnrc6c | trinucleotide repeat containing 6C (Tnrc6c) | -1.53 |
| Zbtb17 | zinc finger and BTB domain containing 17 (Zbtb17) | -1.53 |
| Mif4gd | MIF4G domain containing (Mif4gd) | -1.53 |
| Luzp1 | leucine zipper protein 1 (Luzp1) | -1.53 |
| Whsc2 | Wolf-Hirschhorn syndrome candidate 2 (human) (Whsc2) | -1.53 |
| Trp53bp1 | transformation related protein 53 binding protein 1 (Trp53bp1) | -1.53 |
| Napg | N-ethylmaleimide sensitive fusion protein attachment protein gamma (Napg) | -1.53 |
| Ier3 | immediate early response 3 (Ier3) | -1.53 |
| Il6st | interleukin 6 signal transducer (Il6st) | -1.54 |
| Usp20 | ubiquitin specific peptidase 20 (Usp20) | -1.54 |
| Atp2a3 | ATPase, Ca++ transporting, ubiquitous (Atp2a3) | -1.54 |
| Clk2 | CDC-like kinase 2 (Clk2) | -1.54 |
| Rcan3 | regulator of calcineurin 3 (Rcan3) | -1.54 |
| Gapvd1 | GTPase activating protein and VPS9 domains 1 (Gapvd1) | -1.55 |
| Ndrg1 | N-myc downstream regulated gene 1 (Ndrg1) | -1.55 |
| Rnf8 | ring finger protein 8 (Rnf8) | -1.55 |
| Btla | B and T lymphocyte associated (Btla), transcript variant 2 | -1.55 |
| Klhl6 | kelch-like 6 (Drosophila) (Klhl6) | -1.55 |
| Prep | prolyl endopeptidase (Prep) | -1.55 |
| Statip1 | signal transducer and activator of transcription interacting protein 1 (Statip1) | -1.55 |
| Tmem63a | transmembrane protein 63a (Tmem63a) | -1.56 |
| ORF61 | open reading frame 61 (ORF61) | -1.56 |
| Utx | ubiquitously transcribed tetratricopeptide repeat gene, X chromosome (Utx) | -1.56 |
| D11Wsu47e | DNA segment, Chr 11, Wayne State University 47, expressed (D11Wsu47e) | -1.56 |
| Zc3h18 | zinc finger CCCH-type containing 18 (Zc3h18), transcript variant 1 | -1.56 |
| Pick1 | protein interacting with C kinase 1 (Pick1), transcript variant 1 | -1.56 |
| Krtcap3 | keratinocyte associated protein 3 (Krtcap3) | -1.56 |
| Nagk | N-acetylglucosamine kinase (Nagk) | -1.57 |
| Ubp1 | upstream binding protein 1 (Ubp1) | -1.57 |
| 3300001P08Rik | RIKEN cDNA 3300001P08 gene (3300001P08Rik) | -1.57 |
| 4833420G17Rik | RIKEN cDNA 4833420G17 gene (4833420G17Rik) | -1.57 |
| Pld4 | phospholipase D family, member 4 (Pld4) | -1.57 |
| Prg2 | proteoglycan 2, bone marrow (Prg2) | -1.57 |
| Coro1a | coronin, actin binding protein 1A (Coro1a) | -1.57 |
| Prpf19 | PRP19/PSO4 pre-mRNA processing factor 19 homolog (S. cerevisiae) (Prpf19) | -1.57 |
| Ldb1 | LIM domain binding 1 (Ldb1), transcript variant 3 | -1.58 |
| Arhgef1 | Rho guanine nucleotide exchange factor (GEF) 1 (Arhgef1) | -1.58 |
| Slc24a6 | solute carrier family 24 (sodium/potassium/calcium exchanger), member 6 (Slc24a6) | -1.58 |
| Trit1 | tRNA isopentenyltransferase 1 (Trit1) | -1.58 |
| Phc2 | polyhomeotic-like 2 (Drosophila) (Phc2) | -1.58 |
| LOC100044298 | hypothetical protein LOC100044298 (LOC100044298) | -1.58 |
| Atp6v0a2 | ATPase, H+ transporting, lysosomal V0 subunit A2 (Atp6v0a2) | -1.58 |
| 1810027O10Rik | RIKEN cDNA 1810027O10 gene (1810027O10Rik) | -1.58 |
| Lysmd1 | LysM, putative peptidoglycan-binding, domain containing 1 (Lysmd1) | -1.59 |
| Golga2 | golgi autoantigen, golgin subfamily a, 2 (Golga2), transcript variant 1 | -1.59 |
| Ccnd1 | cyclin D1 (Ccnd1) | -1.59 |
| Dcp1b | DCP1 decapping enzyme homolog b (S. cerevisiae) (Dcp1b) | -1.59 |
| Cant1 | calcium activated nucleotidase 1 (Cant1), transcript variant 2 | -1.59 |
| 4930432O21Rik | RIKEN cDNA 4930432O21 gene (4930432O21Rik) | -1.59 |
| Peci | peroxisomal delta3, delta2-enoyl-Coenzyme A isomerase (Peci) | -1.59 |
| Hgs | HGF-regulated tyrosine kinase substrate (Hgs) | -1.6 |
| LOC100048445 | PREDICTED: similar to fau (LOC100048445) | -1.6 |
| Wsb2 | WD repeat and SOCS box-containing 2 (Wsb2) | -1.6 |
| Tgfbi | transforming growth factor, beta induced (Tgfbi) | -1.6 |
| Lyrm2 | LYR motif containing 2 (Lyrm2) | -1.61 |
| Trim56 | tripartite motif-containing 56 (Trim56) | -1.61 |
| Cnp | 2',3'-cyclic nucleotide 3' phosphodiesterase (Cnp) | -1.61 |
| Degs1 | degenerative spermatocyte homolog 1 (Drosophila) (Degs1) | -1.61 |
| Gadd45g | growth arrest and DNA-damage-inducible 45 gamma (Gadd45g) | -1.61 |
| Gpc1 | glypican 1 (Gpc1) | -1.61 |
| Helb | helicase (DNA) B (Helb) | -1.62 |
| Ep400 | E1A binding protein p400 (Ep400) | -1.62 |
| Rbm5 | RNA binding motif protein 5 (Rbm5) | -1.62 |
| Snrp70 | U1 small nuclear ribonucleoprotein polypeptide A (Snrp70) | -1.62 |
| Ift172 | intraflagellar transport 172 homolog (Chlamydomonas) (Ift172) | -1.62 |
| Daxx | Fas death domain-associated protein (Daxx) | -1.63 |
| Tmem23 | transmembrane protein 23 (Tmem23) | -1.63 |
| Fxyd5 | FXYD domain-containing ion transport regulator 5 (Fxyd5) | -1.63 |
| H2-DMb1 | histocompatibility 2, class II, locus Mb1 (H2-DMb1) | -1.63 |
| Stat1 | signal transducer and activator of transcription 1 (Stat1) | -1.63 |
| Agrn | agrin (Agrn) | -1.63 |
| Pip4k2b | phosphatidylinositol-5-phosphate 4-kinase, type II, beta (Pip4k2b) | -1.63 |
| Ehmt2 | euchromatic histone lysine N-methyltransferase 2 (Ehmt2), transcript variant short | -1.63 |
| Txlna | taxilin alpha (Txlna) | -1.64 |
| Hp1bp3 | heterochromatin protein 1, binding protein 3 (Hp1bp3) | -1.64 |
| BC025076 | membrane magnesium transporter 2 (Mmgt2) | -1.64 |
| Nfrkb | nuclear factor related to kappa B binding protein (Nfrkb) | -1.64 |
| Phkg2 | phosphorylase kinase, gamma 2 (testis) (Phkg2) | -1.64 |
| Gas6 | growth arrest specific 6 (Gas6) | -1.65 |
| Cdipt | CDP-diacylglycerol--inositol 3-phosphatidyltransferase (phosphatidylinositol synthase) (Cdipt) | -1.66 |
| Pacs1 | phosphofurin acidic cluster sorting protein 1 (Pacs1) | -1.66 |
| Irf1 | interferon regulatory factor 1 (Irf1) | -1.66 |
| Rbak | RB-associated KRAB repressor (Rbak), transcript variant 1 | -1.66 |
| Rnf145 | ring finger protein 145 (Rnf145) | -1.66 |
| Eps15l1 | epidermal growth factor receptor pathway substrate 15-like 1 (Eps15l1) | -1.66 |
| Cdkn2aipnl | CDKN2A interacting protein N-terminal like (Cdkn2aipnl) | -1.67 |
| D4Wsu132e | DNA segment, Chr 4, Wayne State University 132, expressed (D4Wsu132e) | -1.67 |
| LOC100045882 | hypothetical protein LOC100045882 (LOC100045882) | -1.67 |
| Tagap | T-cell activation Rho GTPase-activating protein (Tagap) | -1.67 |
| Cybasc3 | cytochrome b, ascorbate dependent 3 (Cybasc3) | -1.67 |
| Ccnd2 | cyclin D2 (Ccnd2) | -1.67 |
| Slc4a2 | solute carrier family 4 (anion exchanger), member 2 (Slc4a2) | -1.68 |
| Zcchc8 | zinc finger, CCHC domain containing 8 (Zcchc8) | -1.68 |
| Tomm22 | translocase of outer mitochondrial membrane 22 homolog (yeast) (Tomm22), nuclear gene encoding mitochondrial protein | -1.68 |
| Psap | prosaposin (Psap) | -1.68 |
| 6430527G18Rik | RIKEN cDNA 6430527G18 gene (6430527G18Rik) | -1.68 |
| LOC100045005 | similar to Deltex3 (LOC100045005), misc RNA. | -1.68 |
| Atp6ap1 | ATPase, H+ transporting, lysosomal accessory protein 1 (Atp6ap1) | -1.69 |
| EG434197 | predicted gene, EG434197 (EG434197) | -1.7 |
| Pitpnm2 | phosphatidylinositol transfer protein, membrane-associated 2 (Pitpnm2) | -1.7 |
| Slc35b3 | solute carrier family 35, member B3 (Slc35b3) | -1.71 |
| Ptpre | protein tyrosine phosphatase, receptor type, E (Ptpre) | -1.71 |
| Dhx38 | DEAH (Asp-Glu-Ala-His) box polypeptide 38 (Dhx38) | -1.71 |
| LOC100045780 | similar to metalloprotease-disintegrin meltrin beta (LOC100045780) | -1.71 |
| Hcst | hematopoietic cell signal transducer (Hcst) | -1.72 |
| Vars2 | valyl-tRNA synthetase 2, mitochondrial (putative) (Vars2) | -1.72 |
| 2400003C14Rik | RIKEN cDNA 2400003C14 gene (2400003C14Rik) | -1.72 |
| Preb | prolactin regulatory element binding (Preb) | -1.72 |
| Kctd2 | potassium channel tetramerisation domain containing 2 (Kctd2) | -1.72 |
| AI450540 | expressed sequence AI450540 (AI450540) | -1.72 |
| Ppox | protoporphyrinogen oxidase (Ppox), nuclear gene encoding mitochondrial protein | -1.72 |
| Ppp4c | protein phosphatase 4, catalytic subunit (Ppp4c) | -1.73 |
| Med15 | mediator complex subunit 15 (Med15), transcript variant 2 | -1.73 |
| Zfp263 | zinc finger protein 263 (Zfp263) | -1.73 |
| Ccnd1 | cyclin D1 (Ccnd1) | -1.73 |
| Ccdc130 | coiled-coil domain containing 130 (Ccdc130) | -1.74 |
| LOC100047369 | similar to 0610007P22Rik protein (LOC100047369), misc RNA. | -1.74 |
| Ars2 | arsenate resistance protein 2 (Ars2) | -1.74 |
| Abcc5 | ATP-binding cassette, sub-family C (CFTR/MRP), member 5 (Abcc5), transcript variant 2 | -1.75 |
| Notch1 | Notch gene homolog 1 (Drosophila) (Notch1) | -1.75 |
| Gmfg | glia maturation factor, gamma (Gmfg), transcript variant 1 | -1.75 |
| Kns2 | kinesin 2 (Kns2), transcript variant d | -1.75 |
| Dok2 | docking protein 2 (Dok2) | -1.76 |
| Skiv2l | superkiller viralicidic activity 2-like (S. cerevisiae) (Skiv2l) | -1.76 |
| Bcl11b | B-cell leukemia/lymphoma 11B (Bcl11b), transcript variant 2 | -1.76 |
| Arhgap17 | Rho GTPase activating protein 17 (Arhgap17) | -1.76 |
| Edem2 | ER degradation enhancer, mannosidase alpha-like 2 (Edem2) | -1.76 |
| Yeats4 | YEATS domain containing 4 (Yeats4) | -1.76 |
| Mycbp2 | MYC binding protein 2 (Mycbp2) | -1.76 |
| Specc1l | SPECC1-like (Specc1l) | -1.76 |
| Med23 | mediator complex subunit 23 (Med23) | -1.76 |
| Lias | lipoic acid synthetase (Lias), nuclear gene encoding mitochondrial protein | -1.76 |
| 2700087H15Rik | mesoderm induction early response 1, family member 2 (Mier2) | -1.77 |
| Pqlc1 | PQ loop repeat containing 1 (Pqlc1) | -1.77 |
| Pacs2 | phosphofurin acidic cluster sorting protein 2 (Pacs2) | -1.77 |
| Hsdl1 | hydroxysteroid dehydrogenase like 1 (Hsdl1) | -1.77 |
| Ap3m2 | adaptor-related protein complex 3, mu 2 subunit (Ap3m2) | -1.77 |
| Napg | N-ethylmaleimide sensitive fusion protein attachment protein gamma (Napg) | -1.77 |
| Akna | AT-hook transcription factor (Akna) | -1.77 |
| Hsp90b1 | heat shock protein 90, beta (Grp94), member 1 (Hsp90b1) | -1.77 |
| Vps16 | vacuolar protein sorting 16 (yeast) (Vps16) | -1.77 |
| Rag1ap1 | recombination activating gene 1 activating protein 1 (Rag1ap1) | -1.78 |
| ENSMUSG00000053178 | predicted gene, ENSMUSG00000053178 (ENSMUSG00000053178), nuclear gene encoding mitochondrial protein | -1.78 |
| Cdc2l1 | cell division cycle 2-like 1 (Cdc2l1) | -1.78 |
| 2410001C21Rik | RIKEN cDNA 2410001C21 gene (2410001C21Rik) | -1.78 |
| Scrib | scribbled homolog (Drosophila) (Scrib) | -1.78 |
| Slc25a45 | solute carrier family 25, member 45 (Slc25a45) | -1.78 |
| Junb | Jun-B oncogene (Junb) | -1.78 |
| Arrdc3 | arrestin domain containing 3 (Arrdc3) | -1.78 |
| Erp29 | endoplasmic reticulum protein 29 (Erp29) | -1.79 |
| Keap1 | kelch-like ECH-associated protein 1 (Keap1) | -1.79 |
| Slc25a28 | solute carrier family 25, member 28 (Slc25a28) | -1.79 |
| Sppl3 | signal peptide peptidase 3 (Sppl3) | -1.79 |
| A430107D22Rik | RIKEN cDNA A430107D22 gene (A430107D22Rik) | -1.79 |
| Zkscan6 | zinc finger with KRAB and SCAN domains 6 (Zkscan6) | -1.79 |
| Oma1 | OMA1 homolog, zinc metallopeptidase (S. cerevisiae) (Oma1) | -1.8 |
| Ddx6 | DEAD (Asp-Glu-Ala-Asp) box polypeptide 6 (Ddx6) | -1.8 |
| Snx17 | sorting nexin 17 (Snx17) | -1.8 |
| 3110056O03Rik | RIKEN cDNA 3110056O03 gene (3110056O03Rik) | -1.8 |
| Clk1 | CDC-like kinase 1 (Clk1) | -1.81 |
| Slc25a38 | solute carrier family 25, member 38 (Slc25a38) | -1.81 |
| Btbd12 | BTB (POZ) domain containing 12 (Btbd12) | -1.81 |
| Irf8 | interferon regulatory factor 8 (Irf8) | -1.81 |
| Vat1 | vesicle amine transport protein 1 homolog (T californica) (Vat1) | -1.81 |
| Relb | avian reticuloendotheliosis viral (v-rel) oncogene related B (Relb) | -1.82 |
| Inmt | indolethylamine N-methyltransferase (Inmt) | -1.82 |
| Gripap1 | GRIP1 associated protein 1 (Gripap1) | -1.82 |
| Arhgap9 | Rho GTPase activating protein 9 (Arhgap9) | -1.83 |
| Usp52 | ubiquitin specific peptidase 52 (Usp52) | -1.83 |
| 4933439C20Rik | RIKEN cDNA 4933439C20 gene (4933439C20Rik) | -1.84 |
| Bat1a | HLA-B-associated transcript 1A (Bat1a) | -1.84 |
| BC067047 | cDNA sequence BC067047 (BC067047) | -1.84 |
| Gba | glucosidase, beta, acid (Gba) | -1.84 |
| Bcl9l | B cell CLL/lymphoma 9-like (Bcl9l) | -1.85 |
| Ppp1r9b | protein phosphatase 1, regulatory subunit 9B (Ppp1r9b) | -1.85 |
| C230093N12Rik | RIKEN cDNA C230093N12 gene (C230093N12Rik) | -1.85 |
| Clk3 | CDC-like kinase 3 (Clk3) | -1.86 |
| LOC100047369 | similar to 0610007P22Rik protein (LOC100047369), misc RNA. | -1.86 |
| Arhgef1 | Rho guanine nucleotide exchange factor (GEF) 1 (Arhgef1) | -1.86 |
| Gimap6 | GTPase, IMAP family member 6 (Gimap6) | -1.86 |
| Sfrs14 | splicing factor, arginine/serine-rich 14 (Sfrs14) | -1.86 |
| Mdm2 | transformed mouse 3T3 cell double minute 2 (Mdm2) | -1.86 |
| Aasdh | aminoadipate-semialdehyde dehydrogenase (Aasdh) | -1.87 |
| Map4k2 | mitogen-activated protein kinase kinase kinase kinase 2 (Map4k2) | -1.87 |
| Dscr1l2 | Down syndrome critical region gene 1-like 2 (Dscr1l2) | -1.87 |
| 6330569M22Rik | RIKEN cDNA 6330569M22 gene (6330569M22Rik) | -1.87 |
| Camta2 | calmodulin binding transcription activator 2 (Camta2) | -1.88 |
| Ddx24 | DEAD (Asp-Glu-Ala-Asp) box polypeptide 24 (Ddx24) | -1.89 |
| 2410001C21Rik | RIKEN cDNA 2410001C21 gene (2410001C21Rik) | -1.89 |
| Trabd | TraB domain containing (Trabd) | -1.89 |
| Ars2 | arsenate resistance protein 2 (Ars2) | -1.89 |
| Cbx7 | chromobox homolog 7 (Cbx7) | -1.9 |
| Dgka | diacylglycerol kinase, alpha (Dgka) | -1.9 |
| Faah | fatty acid amide hydrolase (Faah) | -1.9 |
| Tmem68 | transmembrane protein 68 (Tmem68) | -1.9 |
| Sidt2 | SID1 transmembrane family, member 2 (Sidt2) | -1.9 |
| Herpud1 | homocysteine-inducible, endoplasmic reticulum stress-inducible, ubiquitin-like domain member 1 (Herpud1) | -1.9 |
| Scarf2 | scavenger receptor class F, member 2 (Scarf2) | -1.92 |
| Med12 | mediator of RNA polymerase II transcription, subunit 12 homolog (yeast) (Med12) | -1.92 |
| Gsdmdc1 | gasdermin domain containing 1 (Gsdmdc1) | -1.93 |
| Mapk1ip1 | mitogen-activated protein kinase 1 interacting protein 1 (Mapk1ip1), transcript variant 1 | -1.93 |
| LOC676420 | similar to ceramide kinases (LOC676420), misc RNA. | -1.94 |
| Rbm5 | RNA binding motif protein 5 (Rbm5) | -1.94 |
| Tubb2b | tubulin, beta 2b (Tubb2b) | -1.94 |
| Birc2 | baculoviral IAP repeat-containing 2 (Birc2) | -1.94 |
| Psmb9 | proteasome (prosome, macropain) subunit, beta type 9 (large multifunctional peptidase 2) (Psmb9) | -1.94 |
| Psmd13 | proteasome (prosome, macropain) 26S subunit, non-ATPase, 13 (Psmd13) | -1.95 |
| Cd44 | CD44 antigen (Cd44), transcript variant 2 | -1.95 |
| Dgkz | diacylglycerol kinase zeta (Dgkz) | -1.96 |
| 1110038F14Rik | RIKEN cDNA 1110038F14 gene (1110038F14Rik) | -1.96 |
| Foxj2 | forkhead box J2 (Foxj2) | -1.96 |
| Dhrsx | dehydrogenase/reductase (SDR family) X chromosome (Dhrsx) | -1.96 |
| 2410002F23Rik | RIKEN cDNA 2410002F23 gene (2410002F23Rik) | -1.96 |
| Mterf | mitochondrial transcription termination factor (Mterf), nuclear gene encoding mitochondrial protein, transcript variant 1 | -1.96 |
| AI467606 | expressed sequence AI467606 (AI467606) | -1.96 |
| Ankrd54 | ankyrin repeat domain 54 (Ankrd54) | -1.97 |
| Acox3 | acyl-Coenzyme A oxidase 3, pristanoyl (Acox3) | -1.97 |
| Ttc14 | tetratricopeptide repeat domain 14 (Ttc14) | -1.97 |
| Brd9 | bromodomain containing 9 (Brd9) | -1.97 |
| Il11ra1 | interleukin 11 receptor, alpha chain 1 (Il11ra1) | -1.98 |
| Itgb7 | integrin beta 7 (Itgb7) | -1.98 |
| Mark3 | MAP/microtubule affinity-regulating kinase 3 (Mark3) | -1.98 |
| Prpf38b | PRP38 pre-mRNA processing factor 38 (yeast) domain containing B (Prpf38b) | -1.98 |
| Zmym3 | zinc finger, MYM-type 3 (Zmym3) | -1.99 |
| Slc35c2 | solute carrier family 35, member C2 (Slc35c2) | -1.99 |
| Dgkz | diacylglycerol kinase zeta (Dgkz) | -2 |
| Ehd1 | EH-domain containing 1 (Ehd1) | -2 |
| Ascc2 | activating signal cointegrator 1 complex subunit 2 (Ascc2) | -2 |
| Lrrfip1 | leucine rich repeat (in FLII) interacting protein 1 (Lrrfip1) | -2 |
| Ubtf | upstream binding transcription factor, RNA polymerase I (Ubtf), transcript variant 2 | -2.01 |
| Dgka | diacylglycerol kinase, alpha (Dgka) | -2.01 |
| Ars2 | arsenate resistance protein 2 (Ars2) | -2.01 |
| Chkb | choline kinase beta (Chkb) | -2.02 |
| Ihpk1 | inositol hexaphosphate kinase 1 (Ihpk1) | -2.02 |
| Vav1 | vav 1 oncogene (Vav1) | -2.02 |
| Map3k7ip2 | mitogen-activated protein kinase kinase kinase 7 interacting protein 2 (Map3k7ip2) | -2.02 |
| Syvn1 | synovial apoptosis inhibitor 1, synoviolin (Syvn1) | -2.02 |
| Mterf | mitochondrial transcription termination factor (Mterf), nuclear gene encoding mitochondrial protein, transcript variant 2 | -2.03 |
| Clk4 | CDC like kinase 4 (Clk4) | -2.04 |
| Utx | ubiquitously transcribed tetratricopeptide repeat gene, X chromosome (Utx) | -2.04 |
| Trib2 | tribbles homolog 2 (Drosophila) (Trib2) | -2.04 |
| Elp2 | elongation protein 2 homolog (S. cerevisiae) (Elp2) | -2.04 |
| Nxf1 | nuclear RNA export factor 1 homolog (S. cerevisiae) (Nxf1) | -2.04 |
| Atp9b | ATPase, class II, type 9B (Atp9b) | -2.05 |
| EG665378 | predicted gene, EG665378 (EG665378) | -2.05 |
| Brf1 | BRF1 homolog, subunit of RNA polymerase III transcription initiation factor IIIB (S. cerevisiae) (Brf1) | -2.05 |
| Mapk11 | mitogen-activated protein kinase 11 (Mapk11) | -2.05 |
| Sgpp1 | sphingosine-1-phosphate phosphatase 1 (Sgpp1) | -2.05 |
| Sept9 | septin 9 (Sept9) | -2.05 |
| Csnk1g2 | casein kinase 1, gamma 2 (Csnk1g2) | -2.06 |
| Hvcn1 | hydrogen voltage-gated channel 1 (Hvcn1), transcript variant 1 | -2.06 |
| Sidt2 | SID1 transmembrane family, member 2 (Sidt2) | -2.08 |
| Acss1 | acyl-CoA synthetase short-chain family member 1 (Acss1), nuclear gene encoding mitochondrial protein | -2.08 |
| Trpv2 | transient receptor potential cation channel, subfamily V, member 2 (Trpv2) | -2.08 |
| Ssbp3 | single-stranded DNA binding protein 3 (Ssbp3), transcript variant 1 | -2.08 |
| 8430432M10Rik | phosphopantothenoylcysteine decarboxylase (Ppcdc) | -2.08 |
| Saps3 | SAPS domain family, member 3 (Saps3) | -2.09 |
| Riok1 | RIO kinase 1 (yeast) (Riok1) | -2.09 |
| Acot1 | acyl-CoA thioesterase 1 (Acot1) | -2.1 |
| Cd86 | CD86 antigen (Cd86) | -2.1 |
| Pik3r1 | phosphatidylinositol 3-kinase, regulatory subunit, polypeptide 1 (p85 alpha) (Pik3r1), transcript variant 1 | -2.1 |
| 2310061F22Rik | RIKEN cDNA 2310061F22 gene (2310061F22Rik) | -2.11 |
| Spsb3 | splA/ryanodine receptor domain and SOCS box containing 3 (Spsb3) | -2.12 |
| Pkm2 | pyruvate kinase, muscle (Pkm2) | -2.12 |
| Zc3h7a | zinc finger CCCH type containing 7 A (Zc3h7a) | -2.12 |
| BC037034 | cDNA sequence BC037034 (BC037034) | -2.13 |
| Suv420h2 | suppressor of variegation 4-20 homolog 2 (Drosophila) (Suv420h2) | -2.13 |
| Nrbp2 | nuclear receptor binding protein 2 (Nrbp2) | -2.14 |
| 9130422G05Rik | tetratricopeptide repeat domain 39B (Ttc39b) | -2.14 |
| 2410025L10Rik | RIKEN cDNA 2410025L10 gene (2410025L10Rik) | -2.14 |
| Trim39 | tripartite motif-containing 39 (Trim39) | -2.14 |
| Nfyb | nuclear transcription factor-Y beta (Nfyb) | -2.14 |
| Brd2 | bromodomain containing 2 (Brd2), transcript variant 2 | -2.15 |
| Vars | valyl-tRNA synthetase (Vars) | -2.15 |
| Snapc3 | small nuclear RNA activating complex, polypeptide 3 (Snapc3) | -2.15 |
| Hsd3b2 | hydroxy-delta-5-steroid dehydrogenase, 3 beta- and steroid delta-isomerase 2 (Hsd3b2) | -2.15 |
| Ifngr2 | interferon gamma receptor 2 (Ifngr2) | -2.16 |
| Brd9 | bromodomain containing 9 (Brd9) | -2.16 |
| Hmgcs2 | 3-hydroxy-3-methylglutaryl-Coenzyme A synthase 2 (Hmgcs2), nuclear gene encoding mitochondrial protein | -2.17 |
| 2310021P13Rik | zinc finger SWIM-type containing 8 (Zswim8) | -2.17 |
| Rgl2 | ral guanine nucleotide dissociation stimulator-like 2 (Rgl2) | -2.17 |
| Iap | magnesium transporter 1 (Iap) | -2.18 |
| Tnrc6a | trinucleotide repeat containing 6a (Tnrc6a) | -2.2 |
| Zfp523 | zinc finger protein 523 (Zfp523) | -2.2 |
| Tsc2 | tuberous sclerosis 2 (Tsc2), transcript variant 2 | -2.2 |
| Tap1 | transporter 1, ATP-binding cassette, sub-family B (MDR/TAP) (Tap1) | -2.2 |
| Bach2 | BTB and CNC homology 2 (Bach2) | -2.2 |
| Slc2a6 | solute carrier family 2 (facilitated glucose transporter), member 6 (Slc2a6) | -2.22 |
| Ube1l | ubiquitin-activating enzyme E1-like (Ube1l) | -2.23 |
| Mum1 | melanoma associated antigen (mutated) 1 (Mum1) | -2.23 |
| Gpatch2 | G patch domain containing 2 (Gpatch2) | -2.23 |
| Neu1 | neuraminidase 1 (Neu1) | -2.23 |
| Zfp579 | zinc finger protein 579 (Zfp579) | -2.24 |
| Gdi1 | guanosine diphosphate (GDP) dissociation inhibitor 1 (Gdi1) | -2.25 |
| 2410002F23Rik | RIKEN cDNA 2410002F23 gene (2410002F23Rik) | -2.26 |
| Adcy6 | adenylate cyclase 6 (Adcy6) | -2.26 |
| Iqgap1 | IQ motif containing GTPase activating protein 1 (Iqgap1) | -2.26 |
| Slc44a2 | solute carrier family 44, member 2 (Slc44a2) | -2.27 |
| LOC622404 | hypothetical protein LOC622404 (LOC622404) | -2.27 |
| Atp9b | ATPase, class II, type 9B (Atp9b) | -2.27 |
| Ccdc88b | coiled-coil domain containing 88B (Ccdc88b) | -2.28 |
| Slc25a37 | solute carrier family 25, member 37 (Slc25a37), nuclear gene encoding mitochondrial protein | -2.28 |
| Fcgr4 | Fc receptor, IgG, low affinity IV (Fcgr4) | -2.29 |
| Hvcn1 | hydrogen voltage-gated channel 1 (Hvcn1), transcript variant 1 | -2.29 |
| Pde1b | phosphodiesterase 1B, Ca2+-calmodulin dependent (Pde1b) | -2.3 |
| Ly6d | lymphocyte antigen 6 complex, locus D (Ly6d) | -2.31 |
| Pik3cd | phosphatidylinositol 3-kinase catalytic delta polypeptide (Pik3cd), transcript variant 1 | -2.31 |
| LOC100048020 | similar to RIKEN cDNA 0610009K11 gene (LOC100048020) | -2.31 |
| Bat2 | HLA-B associated transcript 2 (Bat2) | -2.31 |
| Map3k7ip1 | mitogen-activated protein kinase kinase kinase 7 interacting protein 1 (Map3k7ip1) | -2.33 |
| Nisch | nischarin (Nisch) | -2.33 |
| Mta3 | metastasis associated 3 (Mta3) | -2.33 |
| Ehmt2 | euchromatic histone lysine N-methyltransferase 2 (Ehmt2), transcript variant short | -2.34 |
| Tmc6 | transmembrane channel-like gene family 6 (Tmc6), transcript variant 1 | -2.34 |
| Il18r1 | interleukin 18 receptor 1 (Il18r1) | -2.35 |
| Myst4 | MYST histone acetyltransferase monocytic leukemia 4 (Myst4) | -2.35 |
| 4930504E06Rik | RIKEN cDNA 4930504E06 gene (4930504E06Rik) | -2.37 |
| Hap1 | huntingtin-associated protein 1 (Hap1) | -2.37 |
| Pold3 | polymerase (DNA-directed), delta 3, accessory subunit (Pold3) | -2.37 |
| Tcof1 | Treacher Collins Franceschetti syndrome 1, homolog (Tcof1) | -2.37 |
| Csnk1g2 | casein kinase 1, gamma 2 (Csnk1g2) | -2.38 |
| Drctnnb1a | family with sequence similarity 126, member A (Fam126a) | -2.38 |
| Fbxw17 | F-box and WD-40 domain protein 17 (Fbxw17) | -2.39 |
| Gtf2h1 | general transcription factor II H, polypeptide 1 (Gtf2h1) | -2.39 |
| Atpbd1b | ATP binding domain 1 family, member B (Atpbd1b) | -2.4 |
| Ager | advanced glycosylation end product-specific receptor (Ager) | -2.4 |
| Cc2d1b | coiled-coil and C2 domain containing 1B (Cc2d1b) | -2.4 |
| 2610207I05Rik | RIKEN cDNA 2610207I05 gene (2610207I05Rik) | -2.41 |
| Tmc6 | transmembrane channel-like gene family 6 (Tmc6), transcript variant 1 | -2.41 |
| Psmd4 | proteasome (prosome, macropain) 26S subunit, non-ATPase, 4 (Psmd4) | -2.41 |
| Gvin1 | GTPase, very large interferon inducible 1 (Gvin1), transcript variant B | -2.41 |
| Mib2 | mindbomb homolog 2 (Drosophila) (Mib2) | -2.43 |
| Birc2 | baculoviral IAP repeat-containing 2 (Birc2) | -2.43 |
| Tcf25 | transcription factor 25 (basic helix-loop-helix) (Tcf25), transcript variant 3 | -2.44 |
| Kcnk6 | potassium inwardly-rectifying channel, subfamily K, member 6 (Kcnk6) | -2.45 |
| Ddx26b | DEAD/H (Asp-Glu-Ala-Asp/His) box polypeptide 26B (Ddx26b) | -2.45 |
| Prkcbp1 | protein kinase C binding protein 1 (Prkcbp1) | -2.45 |
| Mt1 | metallothionein 1 (Mt1) | -2.45 |
| Txnip | thioredoxin interacting protein (Txnip), transcript variant 1 | -2.47 |
| Gtf2h4 | general transcription factor II H, polypeptide 4 (Gtf2h4) | -2.47 |
| Rpl29 | ribosomal protein L29 (Rpl29) | -2.47 |
| Icos | inducible T-cell co-stimulator (Icos) | -2.47 |
| Taf15 | TAF15 RNA polymerase II, TATA box binding protein (TBP)-associated factor (Taf15) | -2.48 |
| Ercc5 | excision repair cross-complementing rodent repair deficiency, complementation group 5 (Ercc5) | -2.49 |
| Bach1 | BTB and CNC homology 1 (Bach1) | -2.52 |
| Klk8 | kallikrein related-peptidase 8 (Klk8) | -2.54 |
| Rgs1 | regulator of G-protein signaling 1 (Rgs1) | -2.54 |
| Ctgf | connective tissue growth factor (Ctgf) | -2.54 |
| Zc3h7a | zinc finger CCCH type containing 7 A (Zc3h7a) | -2.54 |
| Abca7 | ATP-binding cassette, sub-family A (ABC1), member 7 (Abca7) | -2.55 |
| Sin3a | transcriptional regulator, SIN3A (yeast) (Sin3a) | -2.55 |
| Arhgap4 | Rho GTPase activating protein 4 (Arhgap4) | -2.55 |
| Atp2a3 | ATPase, Ca++ transporting, ubiquitous (Atp2a3) | -2.55 |
| Rcsd1 | RCSD domain containing 1 (Rcsd1), transcript variant 1 | -2.56 |
| BC057552 | cDNA sequence BC057552 (BC057552) | -2.56 |
| Cyp4f18 | cytochrome P450, family 4, subfamily f, polypeptide 18 (Cyp4f18) | -2.57 |
| Itpr2 | inositol 1,4,5-triphosphate receptor 2 (Itpr2), transcript variant 1 | -2.58 |
| Rell1 | RELT-like 1 (Rell1) | -2.58 |
| Dmn | desmuslin (Dmn), transcript variant 3 | -2.59 |
| LOC100047214 | similar to PTEN induced putative kinase 1 (LOC100047214) | -2.59 |
| Rgl2 | ral guanine nucleotide dissociation stimulator-like 2 (Rgl2) | -2.61 |
| Pecam1 | platelet/endothelial cell adhesion molecule 1 (Pecam1), transcript variant 2 | -2.61 |
| Haghl | hydroxyacylglutathione hydrolase-like (Haghl) | -2.63 |
| Myo9b | myosin IXb (Myo9b) | -2.63 |
| LOC545056 | ubiquitin-conjugating enzyme E2, J2 homolog pseudogene (LOC545056) on chromosome 14. | -2.63 |
| Rnf40 | ring finger protein 40 (Rnf40) | -2.63 |
| Rab27a | RAB27A, member RAS oncogene family (Rab27a) | -2.65 |
| Stk11 | serine/threonine kinase 11 (Stk11) | -2.65 |
| Dusp7 | dual specificity phosphatase 7 (Dusp7) | -2.67 |
| Ckb | creatine kinase, brain (Ckb) | -2.67 |
| BC030476 | cDNA sequence BC030476 (BC030476) | -2.69 |
| Arid3b | AT rich interactive domain 3B (Bright like) (Arid3b) | -2.69 |
| Tmc6 | transmembrane channel-like gene family 6 (Tmc6), transcript variant 1 | -2.7 |
| Purb | purine rich element binding protein B (Purb) | -2.7 |
| BC017643 | cDNA sequence BC017643 (BC017643) | -2.72 |
| Tcirg1 | T-cell, immune regulator 1, ATPase, H+ transporting, lysosomal V0 protein A3 (Tcirg1) | -2.72 |
| 1810073G14Rik | RIKEN cDNA 1810073G14 gene (1810073G14Rik) | -2.73 |
| Araf | v-raf murine sarcoma 3611 viral oncogene homolog (Araf) | -2.75 |
| Prkcbp1 | protein kinase C binding protein 1 (Prkcbp1) | -2.75 |
| Psen2 | presenilin 2 (Psen2) | -2.76 |
| Myo9b | myosin IXb (Myo9b) | -2.76 |
| Ptpn1 | protein tyrosine phosphatase, non-receptor type 1 (Ptpn1) | -2.78 |
| Ptprs | protein tyrosine phosphatase, receptor type, S (Ptprs) | -2.78 |
| Lgals4 | lectin, galactose binding, soluble 4 (Lgals4) | -2.82 |
| Tmem66 | transmembrane protein 66 (Tmem66) | -2.83 |
| Gnptg | N-acetylglucosamine-1-phosphotransferase, gamma subunit (Gnptg) | -2.84 |
| Dennd1c | DENN/MADD domain containing 1C (Dennd1c) | -2.86 |
| Nup210 | nucleoporin 210 (Nup210) | -2.89 |
| Traf1 | Tnf receptor-associated factor 1 (Traf1) | -2.89 |
| Cyp4f13 | cytochrome P450, family 4, subfamily f, polypeptide 13 (Cyp4f13) | -2.92 |
| Hmha1 | histocompatibility (minor) HA-1 (Hmha1) | -2.92 |
| Dpagt1 | dolichyl-phosphate (UDP-N-acetylglucosamine) acetylglucosaminephosphotransferase 1 (GlcNAc-1-P transferase) (Dpagt1) | -2.93 |
| Per2 | period homolog 2 (Drosophila) (Per2) | -2.94 |
| Sfrs16 | CLK4-associating serine/arginine rich protein (Sfrs16) | -2.95 |
| Oasl2 | 2'-5' oligoadenylate synthetase-like 2 (Oasl2) | -2.95 |
| Dnajc7 | DnaJ (Hsp40) homolog, subfamily C, member 7 (Dnajc7) | -2.96 |
| Ercc5 | excision repair cross-complementing rodent repair deficiency, complementation group 5 (Ercc5) | -2.99 |
| Sirpa | signal-regulatory protein alpha (Sirpa) | -2.99 |
| BC021381 | cDNA sequence BC021381 (BC021381) | -3 |
| Tap2 | transporter 2, ATP-binding cassette, sub-family B (MDR/TAP) (Tap2) | -3.02 |
| Psmb1 | proteasome (prosome, macropain) subunit, beta type 1 (Psmb1) | -3.06 |
| Gnptg | N-acetylglucosamine-1-phosphotransferase, gamma subunit (Gnptg) | -3.06 |
| Galnt10 | UDP-N-acetyl-alpha-D-galactosamine:polypeptide N-acetylgalactosaminyltransferase 10 (Galnt10) | -3.06 |
| Axud1 | AXIN1 up-regulated 1 (Axud1) | -3.07 |
| Amica1 | adhesion molecule, interacts with CXADR antigen 1 (Amica1) | -3.15 |
| Per1 | period homolog 1 (Drosophila) (Per1) | -3.19 |
| Tcirg1 | T-cell, immune regulator 1, ATPase, H+ transporting, lysosomal V0 protein A3 (Tcirg1) | -3.19 |
| Gpr114 | G protein-coupled receptor 114 (Gpr114) | -3.21 |
| Dkk3 | dickkopf homolog 3 (Xenopus laevis) (Dkk3) | -3.21 |
| D17H6S56E-5 | DNA segment, Chr 17, human D6S56E 5 (D17H6S56E-5) | -3.22 |
| Tef | thyrotroph embryonic factor (Tef), transcript variant 1 | -3.27 |
| Cecr5 | cat eye syndrome chromosome region, candidate 5 homolog (human) (Cecr5) | -3.3 |
| D230007K08Rik | RIKEN cDNA D230007K08 gene, transcript variant 5 (D230007K08Rik) | -3.31 |
| Dbp | D site albumin promoter binding protein (Dbp) | -3.35 |
| Epsti1 | epithelial stromal interaction 1 (breast) (Epsti1), transcript variant a | -3.37 |
| Psg23 | pregnancy-specific glycoprotein 23 (Psg23) | -3.43 |
| Ly6d | lymphocyte antigen 6 complex, locus D (Ly6d) | -3.63 |
| Tnfrsf4 | tumor necrosis factor receptor superfamily, member 4 (Tnfrsf4) | -3.83 |
| Ddit4 | DNA-damage-inducible transcript 4 (Ddit4) | -4.14 |
| Rps3a | ribosomal protein S3a (Rps3a) | -4.16 |
| Tsc22d3 | TSC22 domain family 3 (Tsc22d3), transcript variant 1 | -4.54 |
| 6430706D22Rik | RIKEN cDNA 6430706D22 gene (6430706D22Rik) | -4.61 |
| LOC100044862 | similar to Fbxl3 protein (LOC100044862) | -5.08 |
| Bsdc1 | BSD domain containing 1 (Bsdc1) | -5.89 |
| Mgst2 | microsomal glutathione S-transferase 2 (Mgst2) | -7.34 |
| Actb | actin, beta, cytoplasmic (Actb) | -7.99 |
| Emb | embigin (Emb) | -10.7 |
