# Supplementary material for: Genomic deletion of GIT2 induces a premature age-related thymic dysfunction and systemic immune system disruption
Source: Aging (Albany NY). 2017 Mar 4;9(3):706–30. doi: 10.18632/aging.101185 (PMC5391227; doi:10.18632/aging.101185)
Supplement: Supplementary file 4 [file aging-09-706-s004.docx]

**Table S3. IPA-based BioFunction enrichment annotation of transcripts significantly regulated in a differential manner in GIT2KO thymus compared to WT control thymus.** For each significantly-enriched BioFunction the resultant probability of enrichment (p-Value) and the predicted BioFunction stimulation (positive activation z score) or inhibition (negative activation z score) are indicated.

| **IPA BioFunction** | **p-Value** | **Activation z-score** |
| --- | --- | --- |
| concentration of ATP | 1.53E-04 | 2.55 |
| hypoplasia of lymphoid organ | 3.23E-04 | 2.427 |
| synthesis of fatty acid | 2.76E-04 | 2.093 |
| quantity of bone | 1.86E-05 | 2.088 |
| advanced malignant tumor | 1.79E-06 | 2.084 |
| hypoplasia of primary lymphoid organ | 2.90E-04 | 2.008 |
| diffuse lymphoma | 1.11E-04 | -2 |
| cell death of muscle cells | 1.01E-05 | -2.009 |
| phagocytosis of cells | 2.13E-05 | -2.02 |
| quantity of natural killer cells | 3.91E-05 | -2.024 |
| quantity of T lymphocytes | 2.54E-06 | -2.109 |
| proteinuria | 7.84E-04 | -2.115 |
| transcription | 9.68E-10 | -2.185 |
| generation of reactive oxygen species | 8.26E-06 | -2.253 |
| activation of cells | 1.74E-05 | -2.26 |
| interstitial fibrosis | 7.11E-04 | -2.333 |
| quantity of lymphocytes | 4.20E-08 | -2.447 |
| proliferation of fibroblasts | 1.73E-09 | -2.553 |
| expression of RNA | 1.11E-09 | -2.718 |
| quantity of cells | 6.01E-09 | -2.725 |
| quantity of leukocytes | 7.46E-08 | -2.753 |
| quantity of blood cells | 2.19E-09 | -2.951 |
