# Supplementary material for: Genomic deletion of GIT2 induces a premature age-related thymic dysfunction and systemic immune system disruption
Source: Aging (Albany NY). 2017 Mar 4;9(3):706–30. doi: 10.18632/aging.101185 (PMC5391227; doi:10.18632/aging.101185)
Supplement: Supplementary file 6 [file aging-09-706-s006.docx]

**Table S5. Transcripts significantly and differentially regulated in GIT2KO PTLs versus WT thymus.** For each significantly-regulated (p<0.05; Z ratio >± 1.5) transcript the Official Gene Symbol, Transcript description and expression z ratio (GIT2KO PTL versus WT thymus) is indicated.

| **Gene Symbol** | **Description** | **Z ratio** |
| --- | --- | --- |
| Cd209b | CD209b antigen (Cd209b), transcript variant 1 | 9.09 |
| Cxcl13 | chemokine (C-X-C motif) ligand 13 (Cxcl13) | 7.98 |
| Ccl19 | chemokine (C-C motif) ligand 19 (Ccl19) | 6.66 |
| LOC100046120 | similar to clusterin (LOC100046120) | 6.39 |
| Hp | haptoglobin (Hp) | 6.37 |
| Hp | haptoglobin (Hp) | 6.3 |
| Slpi | secretory leukocyte peptidase inhibitor (Slpi) | 6.26 |
| Hp | haptoglobin (Hp) | 6.22 |
| Lyz2 | lysozyme 2 (Lyz2) | 5.72 |
| Cd55 | CD55 antigen (Cd55) | 5.69 |
| Plvap | plasmalemma vesicle associated protein (Plvap) | 5.65 |
| Cd79b | CD79B antigen (Cd79b) | 5.59 |
| Ctse | cathepsin E (Ctse) | 5.57 |
| Serping1 | serine (or cysteine) peptidase inhibitor, clade G, member 1 (Serping1) | 5.49 |
| LOC100047815 | similar to CD79A antigen (immunoglobulin-associated alpha) (LOC100047815) | 5.26 |
| Tnfsf13b | tumor necrosis factor (ligand) superfamily, member 13b (Tnfsf13b) | 5.23 |
| Faim3 | Fas apoptotic inhibitory molecule 3 (Faim3) | 5.18 |
| C4b | complement component 4B (Childo blood group) (C4b) | 5.18 |
| Serpina3f | serine (or cysteine) peptidase inhibitor, clade A, member 3F (Serpina3f) | 5.16 |
| Lrg1 | leucine-rich alpha-2-glycoprotein 1 (Lrg1) | 5.14 |
| Cpxm1 | carboxypeptidase X 1 (M14 family) (Cpxm1) | 5.12 |
| Mfge8 | milk fat globule-EGF factor 8 protein (Mfge8), transcript variant 2 | 5.11 |
| Cd79b | CD79B antigen (Cd79b) | 5.05 |
| Chst3 | carbohydrate (chondroitin 6/keratan) sulfotransferase 3 (Chst3) | 5.02 |
| Tnfsf13b | tumor necrosis factor (ligand) superfamily, member 13b (Tnfsf13b) | 4.97 |
| Pou2af1 | POU domain, class 2, associating factor 1 (Pou2af1) | 4.9 |
| Lmo2 | LIM domain only 2 (Lmo2) | 4.73 |
| Tnfrsf13c | tumor necrosis factor receptor superfamily, member 13c (Tnfrsf13c) | 4.73 |
| Lyzs | lysozyme (Lyzs) | 4.68 |
| Serpina3n | serine (or cysteine) peptidase inhibitor, clade A, member 3N (Serpina3n) | 4.61 |
| Srpk3 | serine/arginine-rich protein specific kinase 3 (Srpk3) | 4.59 |
| Cd22 | CD22 antigen (Cd22), transcript variant 2 | 4.55 |
| Ccl21c | chemokine (C-C motif) ligand 21c (leucine) (Ccl21c) | 4.51 |
| Igfbp7 | insulin-like growth factor binding protein 7 (Igfbp7) | 4.48 |
| Cldn5 | claudin 5 (Cldn5) | 4.47 |
| Cp | ceruloplasmin (Cp), transcript variant 2 | 4.44 |
| Txndc5 | thioredoxin domain containing 5 (Txndc5) | 4.42 |
| Fgd2 | FYVE, RhoGEF and PH domain containing 2 (Fgd2) | 4.39 |
| Chst3 | carbohydrate (chondroitin 6/keratan) sulfotransferase 3 (Chst3) | 4.37 |
| Cxcr5 | chemochine (C-X-C motif) receptor 5 (Cxcr5) | 4.34 |
| Sparc | secreted acidic cysteine rich glycoprotein (Sparc) | 4.32 |
| Mef2c | myocyte enhancer factor 2C (Mef2c) | 4.32 |
| Mfge8 | milk fat globule-EGF factor 8 protein (Mfge8), transcript variant 2 | 4.3 |
| Napsa | napsin A aspartic peptidase (Napsa) | 4.29 |
| Sparc | secreted acidic cysteine rich glycoprotein (Sparc) | 4.28 |
| C2 | complement component 2 (within H-2S) (C2) | 4.26 |
| Ndg2 | Nur77 downstream gene 2 (Ndg2) | 4.26 |
| Reln | reelin (Reln) | 4.23 |
| Rarres2 | retinoic acid receptor responder (tazarotene induced) 2 (Rarres2) | 4.19 |
| 2010001M09Rik | RIKEN cDNA 2010001M09 gene (2010001M09Rik) | 4.18 |
| Flrt3 | fibronectin leucine rich transmembrane protein 3 (Flrt3) | 4.14 |
| Fcrla | Fc receptor-like A (Fcrla) | 4.12 |
| Bgn | biglycan (Bgn) | 4.09 |
| Napsa | napsin A aspartic peptidase (Napsa) | 4.08 |
| Mgst1 | microsomal glutathione S-transferase 1 (Mgst1) | 4.04 |
| Ramp2 | receptor (calcitonin) activity modifying protein 2 (Ramp2) | 4.02 |
| Tspan7 | tetraspanin 7 (Tspan7) | 4.02 |
| S100a8 | S100 calcium binding protein A8 (calgranulin A) (S100a8) | 4.02 |
| Napsa | napsin A aspartic peptidase (Napsa) | 4.02 |
| Dok3 | docking protein 3 (Dok3) | 4.01 |
| H2-Ob | histocompatibility 2, O region beta locus (H2-Ob) | 4.01 |
| LOC100041504 | similar to beta chemokine Exodus-2 (LOC100041504) | 3.99 |
| Fcrla | Fc receptor-like A (Fcrla) | 3.99 |
| Cxcl9 | chemokine (C-X-C motif) ligand 9 (Cxcl9) | 3.97 |
| Il33 | interleukin 33 (Il33) | 3.97 |
| LOC100048721 | similar to fibronectin leucine rich transmembrane protein 3, transcript variant 1 (LOC100048721) | 3.96 |
| Egfl7 | EGF-like domain 7 (Egfl7), transcript variant c | 3.95 |
| Cygb | cytoglobin (Cygb) | 3.92 |
| Mef2c | myocyte enhancer factor 2C (Mef2c) | 3.92 |
| Sox18 | SRY-box containing gene 18 (Sox18) | 3.9 |
| Hhex | hematopoietically expressed homeobox (Hhex) | 3.88 |
| Chst1 | carbohydrate (keratan sulfate Gal-6) sulfotransferase 1 (Chst1) | 3.84 |
| Hba-a1 | hemoglobin alpha, adult chain 1 (Hba-a1) | 3.84 |
| Gpr18 | G protein-coupled receptor 18 (Gpr18) | 3.8 |
| Il4i1 | interleukin 4 induced 1 (Il4i1) | 3.79 |
| S100a9 | S100 calcium binding protein A9 (calgranulin B) (S100a9) | 3.79 |
| Fcrla | Fc receptor-like A (Fcrla) | 3.78 |
| Sorl1 | sortilin-related receptor, LDLR class A repeats-containing (Sorl1) | 3.77 |
| Rasl12 | RAS-like, family 12 (Rasl12) | 3.74 |
| Ccl21b | chemokine (C-C motif) ligand 21b (Ccl21b) | 3.73 |
| Des | desmin (Des) | 3.73 |
| Dnase1l3 | deoxyribonuclease 1-like 3 (Dnas1l3) | 3.73 |
| Igfbp4 | insulin-like growth factor binding protein 4 (Igfbp4) | 3.71 |
| Cyp1b1 | cytochrome P450, family 1, subfamily b, polypeptide 1 (Cyp1b1) | 3.7 |
| Btk | Bruton agammaglobulinemia tyrosine kinase (Btk) | 3.69 |
| Lpl | lipoprotein lipase (Lpl) | 3.69 |
| Tgm2 | transglutaminase 2, C polypeptide (Tgm2) | 3.69 |
| Ppap2a | phosphatidic acid phosphatase 2a (Ppap2a), transcript variant 1 | 3.68 |
| Mylk | myosin, light polypeptide kinase (Mylk) | 3.67 |
| Egfl7 | EGF-like domain 7 (Egfl7), transcript variant b | 3.67 |
| Plcg2 | phospholipase C, gamma 2 (Plcg2) | 3.65 |
| Cav1 | caveolin, caveolae protein 1 (Cav1) | 3.65 |
| H2-DMb2 | histocompatibility 2, class II, locus Mb2 (H2-DMb2) | 3.64 |
| Zcchc18 | zinc finger, CCHC domain containing 18 (Zcchc18), transcript variant 3 | 3.63 |
| Aebp1 | AE binding protein 1 (Aebp1) | 3.63 |
| LOC100044439 | similar to cytochrome P450 CYP4F18 (LOC100044439) | 3.59 |
| Loxl1 | lysyl oxidase-like 1 (Loxl1) | 3.59 |
| Mmrn2 | multimerin 2 (Mmrn2) | 3.59 |
| Arhgap29 | Rho GTPase activating protein 29 (Arhgap29) | 3.58 |
| Ly6c1 | lymphocyte antigen 6 complex, locus C1 (Ly6c1) | 3.56 |
| Cnr2 | cannabinoid receptor 2 (macrophage) (Cnr2) | 3.55 |
| Mef2c | myocyte enhancer factor 2C (Mef2c) | 3.55 |
| Ctsh | cathepsin H (Ctsh) | 3.54 |
| Ccl5 | chemokine (C-C motif) ligand 5 (Ccl5) | 3.52 |
| Enpp2 | ectonucleotide pyrophosphatase/phosphodiesterase 2 (Enpp2) | 3.51 |
| Fxyd6 | FXYD domain-containing ion transport regulator 6 (Fxyd6) | 3.48 |
| Mylk | myosin, light polypeptide kinase (Mylk) | 3.48 |
| Lyz1 | lysozyme 1 (Lyz1) | 3.45 |
| Lyz | lysozyme (Lyz) | 3.44 |
| Rasgrp3 | RAS, guanyl releasing protein 3 (Rasgrp3) | 3.43 |
| Csf1r | colony stimulating factor 1 receptor (Csf1r) | 3.42 |
| Serpina3g | serine (or cysteine) peptidase inhibitor, clade A, member 3G (Serpina3g) | 3.41 |
| Apol7c | apolipoprotein L 7c (Apol7c) | 3.41 |
| Ly86 | lymphocyte antigen 86 (Ly86) | 3.4 |
| Ifitm2 | interferon induced transmembrane protein 2 (Ifitm2) | 3.38 |
| Adcy4 | adenylate cyclase 4 (Adcy4) | 3.37 |
| Sdpr | serum deprivation response (Sdpr) | 3.37 |
| C3 | complement component 3 (C3) | 3.36 |
| Gimap7 | GTPase, IMAP family member 7 (Gimap7) | 3.35 |
| Klf2 | Kruppel-like factor 2 (lung) (Klf2) | 3.35 |
| Ednrb | endothelin receptor type B (Ednrb) | 3.34 |
| Igfbp4 | insulin-like growth factor binding protein 4 (Igfbp4) | 3.32 |
| Dcn | decorin (Dcn) | 3.32 |
| Fcna | ficolin A (Fcna) | 3.3 |
| Eif4ebp1 | eukaryotic translation initiation factor 4E binding protein 1 (Eif4ebp1) | 3.29 |
| Aoc3 | amine oxidase, copper containing 3 (Aoc3) | 3.28 |
| Prdx4 | peroxiredoxin 4 (Prdx4) | 3.28 |
| Nkg7 | natural killer cell group 7 sequence (Nkg7) | 3.27 |
| Ndn | necdin (Ndn) | 3.27 |
| Dennd3 | DENN/MADD domain containing 3 (Dennd3) | 3.25 |
| Hmgcs2 | 3-hydroxy-3-methylglutaryl-Coenzyme A synthase 2 (Hmgcs2), nuclear gene encoding mitochondrial protein | 3.24 |
| Emcn | endomucin (Emcn) | 3.24 |
| Ccr6 | chemokine (C-C motif) receptor 6 (Ccr6) | 3.23 |
| Ncf4 | neutrophil cytosolic factor 4 (Ncf4) | 3.22 |
| Tmem2 | transmembrane protein 2 (Tmem2), transcript variant 2 | 3.21 |
| Sidt1 | SID1 transmembrane family, member 1 (Sidt1) | 3.2 |
| Eng | endoglin (Eng) | 3.2 |
| Pdia4 | protein disulfide isomerase associated 4 (Pdia4) | 3.2 |
| Ifi30 | interferon gamma inducible protein 30 (Ifi30) | 3.19 |
| Ccl9 | chemokine (C-C motif) ligand 9 (Ccl9) | 3.18 |
| Srgn | serglycin (Srgn) | 3.17 |
| Anxa3 | annexin A3 (Anxa3) | 3.17 |
| Robo4 | roundabout homolog 4 (Drosophila) (Robo4) | 3.17 |
| Btla | B and T lymphocyte associated (Btla), transcript variant 2 | 3.17 |
| Vim | vimentin (Vim) | 3.17 |
| Dci | dodecenoyl-Coenzyme A delta isomerase (3,2 trans-enoyl-Coenyme A isomerase) (Dci), nuclear gene encoding mitochondrial protein | 3.16 |
| Tmem204 | transmembrane protein 204 (Tmem204) | 3.15 |
| Slamf9 | SLAM family member 9 (Slamf9) | 3.15 |
| Brp17 | paroxysmal nonkinesiogenic dyskinesia (Brp17) | 3.13 |
| Fcgr2b | Fc receptor, IgG, low affinity IIb (Fcgr2b), transcript variant 2 | 3.13 |
| Cyp4f18 | cytochrome P450, family 4, subfamily f, polypeptide 18 (Cyp4f18) | 3.12 |
| Angptl4 | angiopoietin-like 4 (Angptl4) | 3.11 |
| Hvcn1 | hydrogen voltage-gated channel 1 (Hvcn1), transcript variant 1 | 3.11 |
| E330036I19Rik | RIKEN cDNA E330036I19 gene (E330036I19Rik) | 3.1 |
| Col4a1 | procollagen, type IV, alpha 1 (Col4a1) | 3.09 |
| Cd59a | CD59a antigen (Cd59a) | 3.08 |
| Evi2a | ecotropic viral integration site 2a (Evi2a), transcript variant 2 | 3.08 |
| P2ry13 | purinergic receptor P2Y, G-protein coupled 13 (P2ry13) | 3.05 |
| Unc93b1 | unc-93 homolog B1 (C. elegans) (Unc93b1) | 3.05 |
| Myl4 | myosin, light polypeptide 4 (Myl4) | 3.04 |
| B3gnt5 | UDP-GlcNAc:betaGal beta-1,3-N-acetylglucosaminyltransferase 5 (B3gnt5) | 3.04 |
| Nuak2 | NUAK family, SNF1-like kinase, 2 (Nuak2) | 3.03 |
| Irak3 | interleukin-1 receptor-associated kinase 3 (Irak3) | 3.02 |
| Anxa3 | annexin A3 (Anxa3) | 3.01 |
| Tgfbi | transforming growth factor, beta induced (Tgfbi) | 3.01 |
| Sepp1 | selenoprotein P, plasma, 1 (Sepp1), transcript variant 1 | 2.98 |
| BC028528 | cDNA sequence BC028528 (BC028528) | 2.98 |
| Trf | transferrin (Trf) | 2.98 |
| Tpst1 | protein-tyrosine sulfotransferase 1 (Tpst1) | 2.97 |
| Col4a2 | collagen, type IV, alpha 2 (Col4a2) | 2.97 |
| Swap70 | SWA-70 protein (Swap70) | 2.97 |
| Ldb2 | LIM domain binding 2 (Ldb2) | 2.97 |
| Hvcn1 | hydrogen voltage-gated channel 1 (Hvcn1), transcript variant 1 | 2.96 |
| Glipr2 | GLI pathogenesis-related 2 (Glipr2) | 2.96 |
| Slc11a1 | solute carrier family 11 (proton-coupled divalent metal ion transporters), member 1 (Slc11a1) | 2.95 |
| Creld2 | cysteine-rich with EGF-like domains 2 (Creld2) | 2.95 |
| 1200002N14Rik | RIKEN cDNA 1200002N14 gene (1200002N14Rik) | 2.94 |
| Nme2 | non-metastatic cells 2, protein (NM23B) expressed in (Nme2), transcript variant 1 | 2.94 |
| Ccr6 | chemokine (C-C motif) receptor 6 (Ccr6) | 2.93 |
| Blk | B lymphoid kinase (Blk) | 2.93 |
| Cd74 | CD74 antigen (invariant polypeptide of major histocompatibility complex, class II antigen-associated) (Cd74), transcript variant 1 | 2.92 |
| Vegfa | vascular endothelial growth factor A (Vegfa), transcript variant 2 | 2.92 |
| Fscn1 | fascin homolog 1, actin bundling protein (Strongylocentrotus purpuratus) (Fscn1) | 2.91 |
| LOC100044538 | similar to immunity-associated nucleotide 4 (LOC100044538), misc RNA. | 2.91 |
| Ebi2 | G protein-coupled receptor 183 (Gpr183) | 2.9 |
| Socs2 | suppressor of cytokine signaling 2 (Socs2) | 2.88 |
| EG630499 | predicted gene, EG630499 (EG630499) | 2.86 |
| Prcp | prolylcarboxypeptidase (angiotensinase C) (Prcp) | 2.85 |
| Mgst1 | microsomal glutathione S-transferase 1 (Mgst1) | 2.85 |
| St6galnac2 | ST6 (alpha-N-acetyl-neuraminyl-2,3-beta-galactosyl-1, 3)-N-acetylgalactosaminide alpha-2,6-sialyltransferase 2 (St6galnac2) | 2.84 |
| Rnase6 | ribonuclease, RNase A family, 6 (Rnase6) | 2.84 |
| Socs3 | suppressor of cytokine signaling 3 (Socs3) | 2.83 |
| Gbp3 | guanylate nucleotide binding protein 3 (Gbp3) | 2.83 |
| Blk | B lymphoid kinase (Blk) | 2.83 |
| Pnpla2 | patatin-like phospholipase domain containing 2 (Pnpla2) | 2.82 |
| Capg | capping protein (actin filament), gelsolin-like (Capg), transcript variant 1 | 2.81 |
| Unc93b1 | unc-93 homolog B1 (C. elegans) (Unc93b1) | 2.8 |
| X99384 | cDNA sequence X99384 (X99384) | 2.8 |
| Swap70 | SWA-70 protein (Swap70) | 2.78 |
| Golm1 | golgi membrane protein 1 (Golm1), transcript variant 1 | 2.77 |
| Pdgfra | platelet derived growth factor receptor, alpha polypeptide (Pdgfra), transcript variant 1 | 2.76 |
| Pygl | liver glycogen phosphorylase (Pygl) | 2.75 |
| H2-Q8 | histocompatibility 2, Q region locus 8 (H2-Q8) | 2.75 |
| Swap70 | SWA-70 protein (Swap70) | 2.74 |
| Lat2 | linker for activation of T cells family, member 2 (Lat2), transcript variant 2 | 2.73 |
| Pold4 | polymerase (DNA-directed), delta 4 (Pold4) | 2.72 |
| Slc9a3r2 | solute carrier family 9 (sodium/hydrogen exchanger), member 3 regulator 2 (Slc9a3r2), transcript variant A | 2.72 |
| Zc3h12d | zinc finger CCCH type containing 12D (Zc3h12d) | 2.71 |
| Cd37 | CD37 antigen (Cd37) | 2.71 |
| Ffar2 | free fatty acid receptor 2 (Ffar2) | 2.7 |
| Vasn | vasorin (Vasn) | 2.7 |
| Esam1 | endothelial cell-specific adhesion molecule (Esam1) | 2.7 |
| Gng10 | guanine nucleotide binding protein (G protein), gamma 10 (Gng10) | 2.69 |
| Eno1 | enolase 1, alpha non-neuron (Eno1) | 2.69 |
| Slc15a3 | solute carrier family 15, member 3 (Slc15a3) | 2.68 |
| Tgfbr2 | transforming growth factor, beta receptor II (Tgfbr2), transcript variant 1 | 2.68 |
| Gpx1 | glutathione peroxidase 1 (Gpx1) | 2.67 |
| Nod1 | nucleotide-binding oligomerization domain containing 1 (Nod1) | 2.66 |
| LOC547343 | similar to H-2 class I histocompatibility antigen, L-D alpha chain precursor (LOC547343) | 2.66 |
| Sel1l | sel-1 suppressor of lin-12-like (C. elegans) (Sel1l), transcript variant 1 | 2.65 |
| Fkbp2 | FK506 binding protein 2 (Fkbp2) | 2.64 |
| Nme2 | non-metastatic cells 2, protein (NM23B) expressed in (Nme2), transcript variant 1 | 2.64 |
| App | amyloid precursor protein (App) | 2.63 |
| Gucy1a3 | guanylate cyclase 1, soluble, alpha 3 (Gucy1a3) | 2.63 |
| Pglyrp1 | peptidoglycan recognition protein 1 (Pglyrp1) | 2.63 |
| Dbi | diazepam binding inhibitor (Dbi), transcript variant 2 | 2.62 |
| Gata2 | GATA binding protein 2 (Gata2) | 2.62 |
| Ccnd1 | cyclin D1 (Ccnd1) | 2.62 |
| LOC100047934 | hypothetical protein LOC100047934 (LOC100047934) | 2.61 |
| Rgl1 | ral guanine nucleotide dissociation stimulator,-like 1 (Rgl1) | 2.6 |
| H2-K1 | histocompatibility 2, K1, K region (H2-K1) | 2.6 |
| Lcp1 | lymphocyte cytosolic protein 1 (Lcp1) | 2.59 |
| Ccnd1 | cyclin D1 (Ccnd1) | 2.59 |
| Hspa8 | heat shock protein 8 (Hspa8) | 2.59 |
| Srgn | serglycin (Srgn) | 2.58 |
| Cd74 | CD74 antigen (invariant polypeptide of major histocompatibility complex, class II antigen-associated) (Cd74), transcript variant 2 | 2.57 |
| Tmem49 | transmembrane protein 49 (Tmem49) | 2.57 |
| Scotin | scotin gene (Scotin), transcript variant 1 | 2.55 |
| Prkcdbp | protein kinase C, delta binding protein (Prkcdbp) | 2.55 |
| H2-Oa | histocompatibility 2, O region alpha locus (H2-Oa) | 2.55 |
| Dcn | decorin (Dcn) | 2.55 |
| H2-Q7 | histocompatibility 2, Q region locus 7 (H2-Q7) | 2.55 |
| Aif1 | allograft inflammatory factor 1 (Aif1) | 2.54 |
| Snx8 | sorting nexin 8 (Snx8) | 2.53 |
| Samsn1 | SAM domain, SH3 domain and nuclear localization signals, 1 (Samsn1) | 2.53 |
| Eef1b2 | eukaryotic translation elongation factor 1 beta 2 (Eef1b2) | 2.53 |
| Rps6 | ribosomal protein S6 (Rps6) | 2.53 |
| Scotin | scotin gene (Scotin), transcript variant 2 | 2.53 |
| Lhfp | lipoma HMGIC fusion partner (Lhfp) | 2.53 |
| Asns | asparagine synthetase (Asns) | 2.52 |
| Fcgrt | Fc receptor, IgG, alpha chain transporter (Fcgrt) | 2.52 |
| Tmem86a | transmembrane protein 86A (Tmem86a) | 2.52 |
| Stard8 | START domain containing 8 (Stard8) | 2.51 |
| Gbp3 | guanylate nucleotide binding protein 3 (Gbp3) | 2.5 |
| Gm2a | GM2 ganglioside activator protein (Gm2a) | 2.5 |
| Tyrobp | TYRO protein tyrosine kinase binding protein (Tyrobp) | 2.5 |
| Hyal2 | hyaluronoglucosaminidase 2 (Hyal2) | 2.49 |
| Creld2 | cysteine-rich with EGF-like domains 2 (Creld2) | 2.49 |
| EG667977 | predicted gene, EG667977 (EG667977) | 2.49 |
| P2ry6 | pyrimidinergic receptor P2Y, G-protein coupled, 6 (P2ry6) | 2.48 |
| Xbp1 | X-box binding protein 1 (Xbp1) | 2.48 |
| Add3 | adducin 3 (gamma) (Add3) | 2.48 |
| Myadm | myeloid-associated differentiation marker (Myadm) | 2.47 |
| Tmem38b | transmembrane protein 38B (Tmem38b) | 2.46 |
| Cebpb | CCAAT/enhancer binding protein (C/EBP), beta (Cebpb) | 2.46 |
| Cdk2ap2 | CDK2-associated protein 2 (Cdk2ap2) | 2.45 |
| Sh3tc1 | SH3 domain and tetratricopeptide repeats 1 (Sh3tc1) | 2.45 |
| Litaf | LPS-induced TN factor (Litaf) | 2.44 |
| Samhd1 | SAM domain and HD domain, 1 (Samhd1) | 2.42 |
| Casp1 | caspase 1 (Casp1) | 2.42 |
| Pld4 | phospholipase D family, member 4 (Pld4) | 2.42 |
| Tmem176b | transmembrane protein 176B (Tmem176b) | 2.41 |
| D3Ucla1 | DNA segment, Chr 3, University of California at Los Angeles 1 (D3Ucla1) | 2.41 |
| Cd40 | CD40 antigen (Cd40), transcript variant 5 | 2.41 |
| Tmem2 | transmembrane protein 2 (Tmem2), transcript variant 1 | 2.4 |
| H2-T10 | histocompatibility 2, T region locus 10 (H2-T10) | 2.39 |
| LOC100045981 | similar to synaptotagmin XI (LOC100045981) | 2.39 |
| Prg2 | proteoglycan 2, bone marrow (Prg2) | 2.38 |
| H2-DMa | histocompatibility 2, class II, locus DMa (H2-DMa) | 2.38 |
| BC004728 | small cell adhesion glycoprotein (Smagp) | 2.37 |
| Atp10d | ATPase, Class V, type 10D (Atp10d) | 2.36 |
| Irak2 | interleukin-1 receptor-associated kinase 2 (Irak2) | 2.36 |
| Nr1h3 | nuclear receptor subfamily 1, group H, member 3 (Nr1h3) | 2.36 |
| Actb | actin, beta, cytoplasmic (Actb) | 2.34 |
| Gvin1 | GTPase, very large interferon inducible 1 (Gvin1), transcript variant B | 2.34 |
| Ciita | class II transactivator (Ciita) | 2.32 |
| Vkorc1 | vitamin K epoxide reductase complex, subunit 1 (Vkorc1) | 2.32 |
| Ostf1 | osteoclast stimulating factor 1 (Ostf1) | 2.31 |
| Rps2 | ribosomal protein S2 (Rps2) | 2.31 |
| Dnajb9 | DnaJ (Hsp40) homolog, subfamily B, member 9 (Dnajb9) | 2.31 |
| Ssr4 | signal sequence receptor, delta (Ssr4) | 2.31 |
| Uap1l1 | UDP-N-acteylglucosamine pyrophosphorylase 1-like 1 (Uap1l1) | 2.3 |
| Mylc2b | myosin light chain, regulatory B (Mylc2b) | 2.3 |
| Grn | granulin (Grn) | 2.29 |
| Mylc2b | myosin light chain, regulatory B (Mylc2b) | 2.28 |
| Cd93 | CD93 antigen (Cd93) | 2.28 |
| Sdc3 | syndecan 3 (Sdc3) | 2.28 |
| Slc46a3 | solute carrier family 46, member 3 (Slc46a3) | 2.28 |
| Pik3cg | phosphoinositide-3-kinase, catalytic, gamma polypeptide (Pik3cg) | 2.26 |
| Scara3 | scavenger receptor class A, member 3 (Scara3) | 2.26 |
| Lgmn | legumain (Lgmn) | 2.26 |
| H2-Ab1 | histocompatibility 2, class II antigen A, beta 1 (H2-Ab1) | 2.25 |
| Sec61b | Sec61 beta subunit (Sec61b) | 2.25 |
| Add3 | adducin 3 (gamma) (Add3) | 2.25 |
| Cd63 | Cd63 antigen (Cd63), transcript variant 2 | 2.24 |
| Smpdl3a | sphingomyelin phosphodiesterase, acid-like 3A (Smpdl3a) | 2.24 |
| Gstm2 | glutathione S-transferase, mu 2 (Gstm2) | 2.24 |
| Aqp1 | aquaporin 1 (Aqp1) | 2.23 |
| Rogdi | rogdi homolog (Drosophila) (Rogdi) | 2.23 |
| Dab2 | disabled 2, mitogen-responsive phosphoprotein (Dab2) | 2.22 |
| Ctsc | cathepsin C (Ctsc) | 2.22 |
| Etfb | electron transferring flavoprotein, beta polypeptide (Etfb) | 2.22 |
| Serpinf1 | serine (or cysteine) peptidase inhibitor, clade F, member 1 (Serpinf1) | 2.22 |
| Mid1ip1 | Mid1 interacting protein 1 (gastrulation specific G12-like (zebrafish)) (Mid1ip1) | 2.21 |
| Lst1 | leukocyte specific transcript 1 (Lst1) | 2.21 |
| Col16a1 | collagen, type XVI, alpha 1 (Col16a1) | 2.21 |
| Gstm1 | glutathione S-transferase, mu 1 (Gstm1) | 2.2 |
| Ms4a6d | membrane-spanning 4-domains, subfamily A, member 6D (Ms4a6d) | 2.2 |
| Cd53 | CD53 antigen (Cd53) | 2.19 |
| Fes | feline sarcoma oncogene (Fes) | 2.19 |
| Arsb | arylsulfatase B (Arsb) | 2.19 |
| C1qb | complement component 1, q subcomponent, beta polypeptide (C1qb) | 2.18 |
| Eef2 | eukaryotic translation elongation factor 2 (Eef2) | 2.18 |
| Ccnd1 | cyclin D1 (Ccnd1) | 2.18 |
| LOC100046953 | similar to Rab6 interacting protein 1 (LOC100046953) | 2.17 |
| Cpt2 | carnitine palmitoyltransferase 2 (Cpt2) | 2.17 |
| Ly6a | lymphocyte antigen 6 complex, locus A (Ly6a) | 2.16 |
| Apobec1 | apolipoprotein B editing complex 1 (Apobec1) | 2.16 |
| Aqp1 | aquaporin 1 (Aqp1) | 2.15 |
| Rhoj | ras homolog gene family, member J (Rhoj) | 2.15 |
| Sepp1 | selenoprotein P, plasma, 1 (Sepp1), transcript variant 2 | 2.15 |
| Stard8 | START domain containing 8 (Stard8) | 2.14 |
| LOC100046883 | similar to CKLF-like MARVEL transmembrane domain containing 3 (LOC100046883) | 2.14 |
| Rps5 | ribosomal protein S5 (Rps5) | 2.13 |
| Timp3 | tissue inhibitor of metalloproteinase 3 (Timp3) | 2.13 |
| Cyp4v3 | cytochrome P450, family 4, subfamily v, polypeptide 3 (Cyp4v3) | 2.13 |
| Aldoa | aldolase 1, A isoform (Aldoa) | 2.13 |
| Plac8 | placenta-specific 8 (Plac8) | 2.13 |
| Lrrk1 | leucine-rich repeat kinase 1 (Lrrk1) | 2.12 |
| Blvrb | biliverdin reductase B (flavin reductase (NADPH)) (Blvrb) | 2.11 |
| Cxcl12 | chemokine (C-X-C motif) ligand 12 (Cxcl12), transcript variant 3 | 2.11 |
| Aatk | apoptosis-associated tyrosine kinase (Aatk) | 2.1 |
| C1qa | complement component 1, q subcomponent, alpha polypeptide (C1qa) | 2.1 |
| Nucb1 | nucleobindin 1 (Nucb1) | 2.09 |
| Ehd4 | EH-domain containing 4 (Ehd4) | 2.09 |
| Rab6ip1 | Rab6 interacting protein 1 (Rab6ip1) | 2.09 |
| Cd63 | Cd63 antigen (Cd63), transcript variant 2 | 2.09 |
| Gstm2 | glutathione S-transferase, mu 2 (Gstm2) | 2.08 |
| Gstm2 | glutathione S-transferase, mu 2 (Gstm2) | 2.08 |
| Hs3st1 | heparan sulfate (glucosamine) 3-O-sulfotransferase 1 (Hs3st1) | 2.08 |
| Centd3 | centaurin, delta 3 (Centd3) | 2.08 |
| Irak2 | interleukin-1 receptor-associated kinase 2 (Irak2) | 2.07 |
| Rassf3 | Ras association (RalGDS/AF-6) domain family member 3 (Rassf3) | 2.06 |
| Idh1 | isocitrate dehydrogenase 1 (NADP+), soluble (Idh1) | 2.06 |
| Sepn1 | selenoprotein N, 1 (Sepn1) | 2.05 |
| Flt1 | FMS-like tyrosine kinase 1 (Flt1) | 2.04 |
| C1qc | complement component 1, q subcomponent, C chain (C1qc) | 2.04 |
| Cd9 | CD9 antigen (Cd9) | 2.02 |
| Cfp | complement factor properdin (Cfp) | 2.02 |
| Prrc1 | proline-rich coiled-coil 1 (Prrc1) | 2.01 |
| Inppl1 | inositol polyphosphate phosphatase-like 1 (Inppl1) | 2.01 |
| Ptprb | protein tyrosine phosphatase, receptor type, B (Ptprb) | 2.01 |
| Adssl1 | adenylosuccinate synthetase like 1 (Adssl1) | 2.01 |
| Grb10 | growth factor receptor bound protein 10 (Grb10) | 2.01 |
| Vkorc1 | vitamin K epoxide reductase complex, subunit 1 (Vkorc1) | 2 |
| B2m | beta-2 microglobulin (B2m) | 2 |
| Zfp318 | zinc finger protein 318 (Zfp318), transcript variant 1 | 2 |
| 2610103J23Rik | metadherin (Mtdh) | 1.99 |
| Idh3g | isocitrate dehydrogenase 3 (NAD+), gamma (Idh3g), nuclear gene encoding mitochondrial protein | 1.99 |
| Sod2 | superoxide dismutase 2 (Sod2) | 1.99 |
| LOC100043391 | similar to QM protein (LOC100043391) | 1.99 |
| Eef2 | eukaryotic translation elongation factor 2 (Eef2) | 1.98 |
| Slc2a6 | solute carrier family 2 (facilitated glucose transporter), member 6 (Slc2a6) | 1.97 |
| Il10ra | interleukin 10 receptor, alpha (Il10ra) | 1.97 |
| Nucb1 | nucleobindin 1 (Nucb1) | 1.97 |
| Dhrs7 | dehydrogenase/reductase (SDR family) member 7 (Dhrs7) | 1.97 |
| Rbp1 | retinol binding protein 1, cellular (Rbp1) | 1.97 |
| Rps3 | ribosomal protein S3 (Rps3) | 1.96 |
| Zeb2 | zinc finger E-box binding homeobox 2 (Zeb2), transcript variant 2 | 1.96 |
| Eef2 | eukaryotic translation elongation factor 2 (Eef2) | 1.96 |
| Laptm4a | lysosomal-associated protein transmembrane 4A (Laptm4a) | 1.96 |
| Cxcl12 | chemokine (C-X-C motif) ligand 12 (Cxcl12), transcript variant 1 | 1.95 |
| Aifm2 | apoptosis-inducing factor, mitochondrion-associated 2 (Aifm2), nuclear gene encoding mitochondrial protein, transcript variant 1 | 1.95 |
| LOC621823 | similar to Proteasome activator complex subunit 2 (Proteasome activator 28-beta subunit) (PA28beta) (PA28b) (Activator of multicatalytic protease subunit 2) (11S regulator complex beta subunit) (REG-beta) (LOC621823) | 1.95 |
| Arrdc4 | arrestin domain containing 4 (Arrdc4), transcript variant 1 | 1.95 |
| Ywhag | tyrosine 3-monooxygenase/tryptophan 5-monooxygenase activation protein, gamma polypeptide (Ywhag) | 1.94 |
| Gbp2 | guanylate nucleotide binding protein 2 (Gbp2) | 1.94 |
| Suclg1 | succinate-CoA ligase, GDP-forming, alpha subunit (Suclg1) | 1.93 |
| Fmo1 | flavin containing monooxygenase 1 (Fmo1) | 1.93 |
| Gja1 | gap junction membrane channel protein alpha 1 (Gja1) | 1.93 |
| Inppl1 | inositol polyphosphate phosphatase-like 1 (Inppl1) | 1.93 |
| Gadd45b | growth arrest and DNA-damage-inducible 45 beta (Gadd45b) | 1.93 |
| Emp3 | epithelial membrane protein 3 (Emp3) | 1.92 |
| Dbi | diazepam binding inhibitor (Dbi), transcript variant 2 | 1.92 |
| LOC100045343 | similar to CDNA sequence BC046404 (LOC100045343), misc RNA. | 1.91 |
| Sep15 | selenoprotein (Sep15) | 1.91 |
| Pfkl | phosphofructokinase, liver, B-type (Pfkl) | 1.91 |
| Mvp | major vault protein (Mvp) | 1.9 |
| Psen2 | presenilin 2 (Psen2) | 1.9 |
| Rrbp1 | ribosome binding protein 1 (Rrbp1), transcript variant 1 | 1.89 |
| Sas | N-acetylneuraminic acid synthase (sialic acid synthase) (Sas) | 1.89 |
| Oxct1 | 3-oxoacid CoA transferase 1 (Oxct1) | 1.89 |
| Sspn | sarcospan (Sspn) | 1.89 |
| Foxo1 | forkhead box O1 (Foxo1) | 1.89 |
| Ppp3ca | protein phosphatase 3, catalytic subunit, alpha isoform (Ppp3ca) | 1.88 |
| Rassf4 | Ras association (RalGDS/AF-6) domain family member 4 (Rassf4) | 1.88 |
| Adi1 | acireductone dioxygenase 1 (Adi1) | 1.88 |
| 9130213B05Rik | RIKEN cDNA 9130213B05 gene (9130213B05Rik) | 1.88 |
| Cd52 | CD52 antigen (Cd52) | 1.88 |
| B2m | beta-2 microglobulin (B2m) | 1.88 |
| Pqlc3 | PQ loop repeat containing (Pqlc3) | 1.88 |
| Plod3 | procollagen-lysine, 2-oxoglutarate 5-dioxygenase 3 (Plod3) | 1.87 |
| Plekho2 | pleckstrin homology domain containing, family O member 2 (Plekho2) | 1.87 |
| Gadd45g | growth arrest and DNA-damage-inducible 45 gamma (Gadd45g) | 1.87 |
| Irf8 | interferon regulatory factor 8 (Irf8) | 1.87 |
| Abca3 | ATP-binding cassette, sub-family A (ABC1), member 3 (Abca3), transcript variant 2 | 1.86 |
| Ppm1m | protein phosphatase 1M (Ppm1m), transcript variant 1 | 1.86 |
| Spcs1 | signal peptidase complex subunit 1 homolog (S. cerevisiae) (Spcs1) | 1.86 |
| Pea15 | phosphoprotein enriched in astrocytes 15 (Pea15) | 1.86 |
| Aldh3b1 | aldehyde dehydrogenase 3 family, member B1 (Aldh3b1) | 1.85 |
| Pros1 | protein S (alpha) (Pros1) | 1.85 |
| Psme1 | proteasome (prosome, macropain) 28 subunit, alpha (Psme1) | 1.85 |
| H2-Eb1 | histocompatibility 2, class II antigen E beta (H2-Eb1) | 1.85 |
| Pdlim7 | PDZ and LIM domain 7 (Pdlim7) | 1.84 |
| Ctsc | cathepsin C (Ctsc) | 1.83 |
| Mtdh | Metadherin (Mtdh) | 1.83 |
| Tpst2 | protein-tyrosine sulfotransferase 2 (Tpst2) | 1.83 |
| Tspan17 | tetraspanin 17 (Tspan17) | 1.83 |
| LOC100045567 | similar to purine nucleoside phosphorylase (LOC100045567) | 1.83 |
| Ube2e2 | ubiquitin-conjugating enzyme E2E 2 (UBC4/5 homolog, yeast) (Ube2e2) | 1.83 |
| Ctsc | cathepsin C (Ctsc) | 1.82 |
| Rpl18 | ribosomal protein L18 (Rpl18) | 1.82 |
| Lgals3bp | lectin, galactoside-binding, soluble, 3 binding protein (Lgals3bp) | 1.82 |
| Cyba | cytochrome b-245, alpha polypeptide (Cyba) | 1.82 |
| 4632417K18Rik | RIKEN cDNA 4632417K18 gene (4632417K18Rik) | 1.82 |
| Gfer | growth factor, erv1 (S. cerevisiae)-like (augmenter of liver regeneration) (Gfer) | 1.81 |
| Atp5l | ATP synthase, H+ transporting, mitochondrial F0 complex, subunit g (Atp5l), nuclear gene encoding mitochondrial protein | 1.8 |
| Bach2 | BTB and CNC homology 2 (Bach2) | 1.8 |
| Mapre2 | microtubule-associated protein, RP/EB family, member 2 (Mapre2) | 1.79 |
| Sgk1 | serum/glucocorticoid regulated kinase 1 (Sgk1) | 1.79 |
| Ltb | lymphotoxin B (Ltb) | 1.78 |
| 1700037H04Rik | RIKEN cDNA 1700037H04 gene (1700037H04Rik) | 1.78 |
| Ppp1ca | protein phosphatase 1, catalytic subunit, alpha isoform (Ppp1ca) | 1.77 |
| Tmsb10 | thymosin, beta 10 (Tmsb10) | 1.77 |
| Clec4n | C-type lectin domain family 4, member n (Clec4n) | 1.77 |
| Aqp1 | aquaporin 1 (Aqp1) | 1.77 |
| Id2 | inhibitor of DNA binding 2 (Id2) | 1.77 |
| Pecam1 | platelet/endothelial cell adhesion molecule 1 (Pecam1), transcript variant 1 | 1.76 |
| Prelp | proline arginine-rich end leucine-rich repeat (Prelp) | 1.76 |
| H2-DMb1 | histocompatibility 2, class II, locus Mb1 (H2-DMb1) | 1.76 |
| Tmem126a | transmembrane protein 126A (Tmem126a) | 1.76 |
| Tnfrsf21 | tumor necrosis factor receptor superfamily, member 21 (Tnfrsf21) | 1.76 |
| Tmem33 | transmembrane protein 33 (Tmem33), transcript variant 2 | 1.75 |
| Gns | glucosamine (N-acetyl)-6-sulfatase (Gns) | 1.75 |
| Hspg2 | perlecan (heparan sulfate proteoglycan 2) (Hspg2) | 1.75 |
| Ehd1 | EH-domain containing 1 (Ehd1) | 1.74 |
| Etfb | electron transferring flavoprotein, beta polypeptide (Etfb) | 1.74 |
| Adssl1 | adenylosuccinate synthetase like 1 (Adssl1) | 1.73 |
| Ctsz | cathepsin Z (Ctsz) | 1.73 |
| Pfdn5 | prefoldin 5 (Pfdn5) | 1.73 |
| Hsp90b1 | heat shock protein 90, beta (Grp94), member 1 (Hsp90b1) | 1.73 |
| Ptp4a2 | protein tyrosine phosphatase 4a2 (Ptp4a2) | 1.73 |
| Tpm4 | tropomyosin 4 (Tpm4) | 1.72 |
| Ifi47 | interferon gamma inducible protein 47 (Ifi47) | 1.72 |
| Rpl7a | ribosomal protein L7a (Rpl7a) | 1.72 |
| Prr13 | proline rich 13 (Prr13) | 1.72 |
| Nt5e | 5' nucleotidase, ecto (Nt5e) | 1.72 |
| Rnase4 | ribonuclease, RNase A family 4 (Rnase4), transcript variant 1 | 1.72 |
| Ndfip1 | Nedd4 family interacting protein 1 (Ndfip1) | 1.72 |
| Rps7 | ribosomal protein S7 (Rps7) | 1.72 |
| ENSMUSG00000068790 | predicted gene, ENSMUSG00000068790 (ENSMUSG00000068790) | 1.72 |
| Irf5 | interferon regulatory factor 5 (Irf5) | 1.72 |
| Slc25a20 | solute carrier family 25 (mitochondrial carnitine/acylcarnitine translocase), member 20 (Slc25a20) | 1.72 |
| Fchsd2 | FCH and double SH3 domains 2 (Fchsd2) | 1.71 |
| Tmem176a | transmembrane protein 176A (Tmem176a) | 1.71 |
| Fbxo4 | F-box protein 4 (Fbxo4) | 1.71 |
| Ndufc1 | NADH dehydrogenase (ubiquinone) 1, subcomplex unknown, 1 (Ndufc1) | 1.71 |
| Ctsz | cathepsin Z (Ctsz) | 1.7 |
| Sult1a1 | sulfotransferase family 1A, phenol-preferring, member 1 (Sult1a1) | 1.7 |
| Rassf4 | Ras association (RalGDS/AF-6) domain family member 4 (Rassf4) | 1.7 |
| Zfp608 | zinc finger protein 608 (Zfp608) | 1.7 |
| Cotl1 | coactosin-like 1 (Dictyostelium) (Cotl1) | 1.69 |
| Atf5 | activating transcription factor 5 (Atf5) | 1.69 |
| Rab31 | RAB31, member RAS oncogene family (Rab31) | 1.69 |
| Apoe | apolipoprotein E (Apoe) | 1.68 |
| Acadvl | acyl-Coenzyme A dehydrogenase, very long chain (Acadvl) | 1.68 |
| Cst3 | cystatin C (Cst3) | 1.67 |
| Cd63 | Cd63 antigen (Cd63), transcript variant 2 | 1.67 |
| Sema4a | sema domain, immunoglobulin domain (Ig), transmembrane domain (TM) and short cytoplasmic domain, (semaphorin) 4A (Sema4a) | 1.67 |
| Fermt2 | fermitin family homolog 2 (Drosophila) (Fermt2) | 1.67 |
| Rps15 | ribosomal protein S15 (Rps15) | 1.66 |
| Pfn1 | profilin 1 (Pfn1) | 1.66 |
| MGC18837 | transmembrane protein 205 (Tmem205) | 1.66 |
| Krtcap2 | keratinocyte associated protein 2 (Krtcap2) | 1.66 |
| Asah3l | N-acylsphingosine amidohydrolase 3-like (Asah3l) | 1.66 |
| Herpud1 | homocysteine-inducible, endoplasmic reticulum stress-inducible, ubiquitin-like domain member 1 (Herpud1) | 1.66 |
| 2310016E02Rik | RIKEN cDNA 2310016E02 gene (2310016E02Rik) | 1.65 |
| Asb2 | ankyrin repeat and SOCS box-containing protein 2 (Asb2) | 1.65 |
| Gstp1 | glutathione S-transferase, pi 1 (Gstp1) | 1.65 |
| Msn | moesin (Msn) | 1.65 |
| Tmed10 | transmembrane emp24-like trafficking protein 10 (yeast) (Tmed10) | 1.65 |
| Dph3 | DPH3 homolog (KTI11, S. cerevisiae) (Dph3), transcript variant 1 | 1.65 |
| LOC100047998 | similar to ribosomal protein L37a (LOC100047998) | 1.65 |
| Gusb | glucuronidase, beta (Gusb) | 1.65 |
| Fkbp9 | FK506 binding protein 9 (Fkbp9) | 1.64 |
| H2-Ab1 | histocompatibility 2, class II antigen A, beta 1 (H2-Ab1) | 1.64 |
| H47 | histocompatibility 47 (H47) | 1.64 |
| Rnf135 | ring finger protein 135 (Rnf135) | 1.64 |
| Cox6a1 | cytochrome c oxidase subunit VIa polypeptide 1 (Cox6a1) | 1.64 |
| LOC641240 | similar to MHC class II antigen beta chain (LOC641240) | 1.64 |
| Irf1 | interferon regulatory factor 1 (Irf1) | 1.64 |
| Arhgef3 | Rho guanine nucleotide exchange factor (GEF) 3 (Arhgef3) | 1.64 |
| Csrp2 | cysteine and glycine-rich protein 2 (Csrp2) | 1.64 |
| Snx10 | sorting nexin 10 (Snx10) | 1.64 |
| Cd44 | CD44 antigen (Cd44), transcript variant 2 | 1.64 |
| Itgb7 | integrin beta 7 (Itgb7) | 1.63 |
| Cd81 | CD 81 antigen (Cd81) | 1.63 |
| Obrgrp | leptin receptor (Lepr) | 1.63 |
| Cd151 | CD151 antigen (Cd151) | 1.63 |
| Rpl36al | ribosomal protein L36a-like (Rpl36al) | 1.63 |
| Ech1 | enoyl coenzyme A hydratase 1, peroxisomal (Ech1) | 1.62 |
| 9130422G05Rik | tetratricopeptide repeat domain 39B (Ttc39b) | 1.62 |
| Hist1h1c | histone cluster 1, H1c (Hist1h1c) | 1.62 |
| Ctsw | cathepsin W (Ctsw) | 1.62 |
| Rabac1 | Rab acceptor 1 (prenylated) (Rabac1) | 1.62 |
| Aadacl1 | arylacetamide deacetylase-like 1 (Aadacl1) | 1.61 |
| Rpl39 | ribosomal protein L39 (Rpl39) | 1.61 |
| Adk | adenosine kinase (Adk) | 1.61 |
| Mbc2 | membrane bound C2 domain containing protein (Mbc2) | 1.61 |
| Yif1a | Yip1 interacting factor homolog A (S. cerevisiae) (Yif1a) | 1.61 |
| LOC381629 | all-trans retinoic acid induced differentiation factor (Atraid) | 1.61 |
| H2-T23 | histocompatibility 2, T region locus 23 (H2-T23) | 1.61 |
| Gnas | GNAS (guanine nucleotide binding protein, alpha stimulating) complex locus (Gnas), transcript variant 3 | 1.6 |
| Kras | v-Ki-ras2 Kirsten rat sarcoma viral oncogene homolog (Kras) | 1.6 |
| Ndufb10 | NADH dehydrogenase (ubiquinone) 1 beta subcomplex, 10 (Ndufb10) | 1.6 |
| Tdrd7 | tudor domain containing 7 (Tdrd7) | 1.59 |
| Tspo | translocator protein (Tspo) | 1.58 |
| Cybasc3 | cytochrome b, ascorbate dependent 3 (Cybasc3) | 1.58 |
| Kcnk6 | potassium inwardly-rectifying channel, subfamily K, member 6 (Kcnk6) | 1.58 |
| Evl | Ena-vasodilator stimulated phosphoprotein (Evl) | 1.58 |
| Pml | promyelocytic leukemia (Pml), transcript variant 1 | 1.57 |
| Tmem205 | transmembrane protein 205 (Tmem205) | 1.57 |
| Fxyd5 | FXYD domain-containing ion transport regulator 5 (Fxyd5) | 1.57 |
| Sdhb | succinate dehydrogenase complex, subunit B, iron sulfur (Ip) (Sdhb) | 1.57 |
| Sec11c | SEC11 homolog C (S. cerevisiae) (Sec11c) | 1.57 |
| 2310001A20Rik | RIKEN cDNA 2310001A20 gene (2310001A20Rik) | 1.57 |
| 1110059E24Rik | RIKEN cDNA 1110059E24 gene (1110059E24Rik) | 1.57 |
| Cox6b1 | cytochrome c oxidase, subunit VIb polypeptide 1 (Cox6b1) | 1.57 |
| H2-Ab1 | histocompatibility 2, class II antigen A, beta 1 (H2-Ab1) | 1.57 |
| Ankrd47 | ankyrin repeat domain 47 (Ankrd47) | 1.56 |
| Tmem109 | transmembrane protein 109 (Tmem109) | 1.56 |
| Notch4 | Notch gene homolog 4 (Drosophila) (Notch4) | 1.56 |
| Arpc1b | actin related protein 2/3 complex, subunit 1B (Arpc1b) | 1.55 |
| 1700021K19Rik | RIKEN cDNA 1700021K19 gene (1700021K19Rik) | 1.55 |
| Dad1 | defender against cell death 1 (Dad1) | 1.55 |
| Slc25a1 | solute carrier family 25 (mitochondrial carrier, citrate transporter), member 1 (Slc25a1), nuclear gene encoding mitochondrial protein | 1.55 |
| Rhbdf1 | rhomboid family 1 (Drosophila) (Rhbdf1) | 1.55 |
| Mmp14 | matrix metallopeptidase 14 (membrane-inserted) (Mmp14) | 1.55 |
| Cnn2 | calponin 2 (Cnn2) | 1.55 |
| Bola2 | bolA-like 2 (E. coli) (Bola2) | 1.54 |
| Gpx4 | glutathione peroxidase 4 (Gpx4), transcript variant 1 | 1.54 |
| Arsa | arylsulfatase A (Arsa) | 1.54 |
| Bin1 | bridging integrator 1 (Bin1) | 1.54 |
| Cd151 | CD151 antigen (Cd151) | 1.54 |
| Rpl12 | ribosomal protein L12 (Rpl12) | 1.54 |
| Ndufc1 | NADH dehydrogenase (ubiquinone) 1, subcomplex unknown, 1 (Ndufc1) | 1.54 |
| Snx2 | sorting nexin 2 (Snx2) | 1.53 |
| Sypl | synaptophysin-like protein (Sypl), transcript variant 1 | 1.53 |
| Cox5a | cytochrome c oxidase, subunit Va (Cox5a), nuclear gene encoding mitochondrial protein | 1.53 |
| Hsd3b7 | hydroxy-delta-5-steroid dehydrogenase, 3 beta- and steroid delta-isomerase 7 (Hsd3b7), transcript variant 1 | 1.52 |
| Tmbim4 | transmembrane BAX inhibitor motif containing 4 (Tmbim4) | 1.52 |
| Galnt11 | UDP-N-acetyl-alpha-D-galactosamine:polypeptide N-acetylgalactosaminyltransferase 11 (Galnt11) | 1.52 |
| Tuba1a | tubulin, alpha 1A (Tuba1a) | 1.52 |
| Srm | spermidine synthase (Srm) | 1.52 |
| Rnaset2 | ribonuclease T2 (Rnaset2), transcript variant 2 | 1.52 |
| Ndufb6 | NADH dehydrogenase (ubiquinone) 1 beta subcomplex, 6 (Ndufb6), nuclear gene encoding mitochondrial protein | 1.52 |
| Tbc1d20 | TBC1 domain family, member 20 (Tbc1d20) | 1.51 |
| Rpl34 | ribosomal protein L34 (Rpl34), transcript variant 1 | 1.51 |
| Tspo | translocator protein (Tspo) | 1.51 |
| Rps9 | ribosomal protein S9 (Rps9) | 1.51 |
| Rras | Harvey rat sarcoma oncogene, subgroup R (Rras) | 1.51 |
| Atp5f1 | ATP synthase, H+ transporting, mitochondrial F0 complex, subunit b, isoform 1 (Atp5f1) | 1.51 |
| Col6a1 | procollagen, type VI, alpha 1 (Col6a1) | 1.51 |
| Irf2 | interferon regulatory factor 2 (Irf2) | 1.5 |
| Pum2 | pumilio 2 (Drosophila) (Pum2) | -1.5 |
| Hpgd | hydroxyprostaglandin dehydrogenase 15 (NAD) (Hpgd) | -1.5 |
| Ttc14 | tetratricopeptide repeat domain 14 (Ttc14) | -1.5 |
| C230071H18Rik | RIKEN cDNA C230071H18 gene (C230071H18Rik) | -1.51 |
| Scrib | scribbled homolog (Drosophila) (Scrib) | -1.51 |
| Atp9b | ATPase, class II, type 9B (Atp9b) | -1.51 |
| Sgpl1 | sphingosine phosphate lyase 1 (Sgpl1) | -1.51 |
| Rnf145 | ring finger protein 145 (Rnf145) | -1.51 |
| Tmc6 | transmembrane channel-like gene family 6 (Tmc6), transcript variant 1 | -1.52 |
| Zfp238 | zinc finger protein 238 (Zfp238), transcript variant 2 | -1.52 |
| Cstf2 | cleavage stimulation factor, 3' pre-RNA subunit 2 (Cstf2) | -1.52 |
| Ankrd10 | ankyrin repeat domain 10 (Ankrd10) | -1.52 |
| Ikzf1 | IKAROS family zinc finger 1 (Ikzf1), transcript variant 1 | -1.53 |
| Fntb | farnesyltransferase, CAAX box, beta (Fntb) | -1.53 |
| Tlk2 | tousled-like kinase 2 (Arabidopsis) (Tlk2) | -1.53 |
| St5 | suppression of tumorigenicity 5 (St5), transcript variant 1 | -1.53 |
| 2610020O08Rik | RIKEN cDNA 2610020O08 gene (2610020O08Rik) | -1.53 |
| H13 | histocompatibility 13 (H13) | -1.54 |
| Hbs1l | Hbs1-like (S. cerevisiae) (Hbs1l), transcript variant 1 | -1.54 |
| Zfp148 | zinc finger protein 148 (Zfp148) | -1.54 |
| Ints1 | integrator complex subunit 1 (Ints1) | -1.55 |
| BC017643 | cDNA sequence BC017643 (BC017643) | -1.55 |
| Utx | ubiquitously transcribed tetratricopeptide repeat gene, X chromosome (Utx) | -1.55 |
| Lyrm2 | LYR motif containing 2 (Lyrm2) | -1.55 |
| Suv420h1 | suppressor of variegation 4-20 homolog 1 (Drosophila) (Suv420h1) | -1.55 |
| Ppp1r9b | protein phosphatase 1, regulatory subunit 9B (Ppp1r9b) | -1.55 |
| Dscr1l2 | Down syndrome critical region gene 1-like 2 (Dscr1l2) | -1.55 |
| 2410127E18Rik | RIKEN cDNA 2410127E18 gene (2410127E18Rik) | -1.56 |
| Ubtf | upstream binding transcription factor, RNA polymerase I (Ubtf), transcript variant 2 | -1.56 |
| Rgl2 | ral guanine nucleotide dissociation stimulator-like 2 (Rgl2) | -1.56 |
| Usp52 | ubiquitin specific peptidase 52 (Usp52) | -1.56 |
| Irf2bp1 | interferon regulatory factor 2 binding protein 1 (Irf2bp1) | -1.57 |
| 4930455F23Rik | RIKEN cDNA 4930455F23 gene (4930455F23Rik) | -1.57 |
| 5730419I09Rik | RIKEN cDNA 5730419I09 gene (5730419I09Rik) | -1.57 |
| Zmym3 | zinc finger, MYM-type 3 (Zmym3) | -1.57 |
| Vav1 | vav 1 oncogene (Vav1) | -1.57 |
| Mark3 | MAP/microtubule affinity-regulating kinase 3 (Mark3) | -1.57 |
| Slc4a2 | solute carrier family 4 (anion exchanger), member 2 (Slc4a2) | -1.58 |
| Usp3 | ubiquitin specific peptidase 3 (Usp3) | -1.58 |
| Aldh4a1 | aldehyde dehydrogenase 4 family, member A1 (Aldh4a1), nuclear gene encoding mitochondrial protein | -1.58 |
| 1810073G14Rik | RIKEN cDNA 1810073G14 gene (1810073G14Rik) | -1.58 |
| Ttc3 | tetratricopeptide repeat domain 3 (Ttc3) | -1.58 |
| Cxcr4 | chemokine (C-X-C motif) receptor 4 (Cxcr4) | -1.58 |
| Cugbp2 | CUG triplet repeat, RNA binding protein 2 (Cugbp2), transcript variant 6 | -1.58 |
| 5730593F17Rik | RIKEN cDNA 5730593F17 gene (5730593F17Rik) | -1.58 |
| Sept9 | septin 9 (Sept9) | -1.58 |
| A730098D12Rik | YTH domain containing 1 (Ythdc1) | -1.59 |
| Wdr51b | WD repeat domain 51B (Wdr51b) | -1.59 |
| Arhgap4 | Rho GTPase activating protein 4 (Arhgap4) | -1.59 |
| Fcho1 | FCH domain only 1 (Fcho1) | -1.6 |
| Bcl7a | B-cell CLL/lymphoma 7A (Bcl7a) | -1.6 |
| Epc1 | enhancer of polycomb homolog 1 (Drosophila) (Epc1), transcript variant 1 | -1.61 |
| Dbp | D site albumin promoter binding protein (Dbp) | -1.61 |
| 2810046L04Rik | RIKEN cDNA 2810046L04 gene (2810046L04Rik) | -1.61 |
| LOC676420 | similar to ceramide kinases (LOC676420), misc RNA. | -1.62 |
| 1810020D17Rik | RIKEN cDNA 1810020D17 gene (1810020D17Rik) | -1.62 |
| Zfp212 | Zinc finger protein 212 (Zfp212) | -1.62 |
| Xlr4a | X-linked lymphocyte-regulated 4A (Xlr4a) | -1.62 |
| Casp2 | caspase 2 (Casp2) | -1.62 |
| Khdrbs1 | KH domain containing, RNA binding, signal transduction associated 1 (Khdrbs1) | -1.63 |
| Slc44a2 | solute carrier family 44, member 2 (Slc44a2) | -1.63 |
| Eif4e3 | eukaryotic translation initiation factor 4E member 3 (Eif4e3) | -1.63 |
| Hist2h2ac | histone cluster 2, H2ac (Hist2h2ac) | -1.63 |
| LOC100047369 | similar to 0610007P22Rik protein (LOC100047369), misc RNA. | -1.63 |
| A430107D22Rik | RIKEN cDNA A430107D22 gene (A430107D22Rik) | -1.63 |
| Dap3 | death associated protein 3 (Dap3), nuclear gene encoding mitochondrial protein | -1.63 |
| LOC545056 | ubiquitin-conjugating enzyme E2, J2 homolog pseudogene (LOC545056) on chromosome 14. | -1.64 |
| Rrm1 | ribonucleotide reductase M1 (Rrm1) | -1.64 |
| Nxf1 | nuclear RNA export factor 1 homolog (S. cerevisiae) (Nxf1) | -1.64 |
| LOC100045040 | similar to ING1 protein (LOC100045040), misc RNA. | -1.64 |
| Helb | helicase (DNA) B (Helb) | -1.65 |
| Ttc3 | tetratricopeptide repeat domain 3 (Ttc3) | -1.65 |
| Rbm38 | RNA binding motif protein 38 (Rbm38) | -1.65 |
| Akap8l | A kinase (PRKA) anchor protein 8-like (Akap8l) | -1.65 |
| Csnk1g2 | casein kinase 1, gamma 2 (Csnk1g2) | -1.65 |
| Atp9b | ATPase, class II, type 9B (Atp9b) | -1.66 |
| Ttc3 | tetratricopeptide repeat domain 3 (Ttc3) | -1.66 |
| Ldb1 | LIM domain binding 1 (Ldb1), transcript variant 3 | -1.66 |
| 2310016C08Rik | RIKEN cDNA 2310016C08 gene (2310016C08Rik) | -1.66 |
| Hes6 | hairy and enhancer of split 6 (Drosophila) (Hes6) | -1.66 |
| Epc1 | enhancer of polycomb homolog 1 (Drosophila) (Epc1), transcript variant 1 | -1.66 |
| Peci | peroxisomal delta3, delta2-enoyl-Coenzyme A isomerase (Peci) | -1.66 |
| Sbf1 | SET binding factor 1 (Sbf1) | -1.67 |
| Dgka | diacylglycerol kinase, alpha (Dgka) | -1.67 |
| Tollip | toll interacting protein (Tollip) | -1.67 |
| Tra2a | transformer 2 alpha homolog (Drosophila) (Tra2a) | -1.68 |
| Purb | purine rich element binding protein B (Purb) | -1.68 |
| Sin3a | transcriptional regulator, SIN3A (yeast) (Sin3a) | -1.68 |
| Mdm2 | transformed mouse 3T3 cell double minute 2 (Mdm2) | -1.68 |
| Mllt11 | myeloid/lymphoid or mixed-lineage leukemia (trithorax homolog, Drosophila); translocated to, 11 (Mllt11) | -1.68 |
| Brd9 | bromodomain containing 9 (Brd9) | -1.68 |
| Chmp1b | chromatin modifying protein 1B (Chmp1b) | -1.69 |
| Plekhg2 | pleckstrin homology domain containing, family G (with RhoGef domain) member 2 (Plekhg2) | -1.69 |
| Klf7 | Kruppel-like factor 7 (ubiquitous) (Klf7) | -1.69 |
| Sh2d2a | SH2 domain protein 2A (Sh2d2a), transcript variant 2 | -1.69 |
| Lysmd1 | LysM, putative peptidoglycan-binding, domain containing 1 (Lysmd1) | -1.69 |
| Bat2 | HLA-B associated transcript 2 (Bat2) | -1.69 |
| Ttc3 | tetratricopeptide repeat domain 3 (Ttc3) | -1.69 |
| Psat1 | phosphoserine aminotransferase 1 (Psat1) | -1.7 |
| 1500031L02Rik | RIKEN cDNA 1500031L02 gene (1500031L02Rik) | -1.7 |
| Zbtb17 | zinc finger and BTB domain containing 17 (Zbtb17) | -1.7 |
| Ars2 | arsenate resistance protein 2 (Ars2) | -1.7 |
| Rtn3 | reticulon 3 (Rtn3), transcript variant 3 | -1.7 |
| Fbxl14 | F-box and leucine-rich repeat protein 14 (Fbxl14) | -1.7 |
| Rab27a | RAB27A, member RAS oncogene family (Rab27a) | -1.71 |
| Trp53inp1 | transformation related protein 53 inducible nuclear protein 1 (Trp53inp1) | -1.71 |
| Tmem63b | transmembrane protein 63b (Tmem63b) | -1.71 |
| Slco3a1 | solute carrier organic anion transporter family, member 3a1 (Slco3a1), transcript variant 1 | -1.71 |
| Map4k2 | mitogen-activated protein kinase kinase kinase kinase 2 (Map4k2) | -1.72 |
| Pdpk1 | 3-phosphoinositide dependent protein kinase 1 (Pdpk1) | -1.72 |
| Dgka | diacylglycerol kinase, alpha (Dgka) | -1.72 |
| Brd9 | bromodomain containing 9 (Brd9) | -1.72 |
| AI450540 | expressed sequence AI450540 (AI450540) | -1.72 |
| Gtf2h1 | general transcription factor II H, polypeptide 1 (Gtf2h1) | -1.72 |
| Tbl1x | transducin (beta)-like 1 X-linked (Tbl1x) | -1.73 |
| Cdkn2aipnl | CDKN2A interacting protein N-terminal like (Cdkn2aipnl) | -1.73 |
| Smox | spermine oxidase (Smox) | -1.73 |
| Stk11 | serine/threonine kinase 11 (Stk11) | -1.73 |
| Usp3 | ubiquitin specific peptidase 3 (Usp3) | -1.73 |
| Traf6 | Tnf receptor-associated factor 6 (Traf6) | -1.73 |
| Llgl1 | lethal giant larvae homolog 1 (Drosophila) (Llgl1) | -1.74 |
| Scarf2 | scavenger receptor class F, member 2 (Scarf2) | -1.74 |
| Gadd45a | growth arrest and DNA-damage-inducible 45 alpha (Gadd45a) | -1.74 |
| Dcp1b | DCP1 decapping enzyme homolog b (S. cerevisiae) (Dcp1b) | -1.74 |
| Plekhg2 | pleckstrin homology domain containing, family G (with RhoGef domain) member 2 (Plekhg2), transcript variant 1 | -1.74 |
| Col18a1 | procollagen, type XVIII, alpha 1 (Col18a1) | -1.75 |
| LOC100045887 | similar to PTB-associated splicing factor (LOC100045887) | -1.75 |
| 4833420G17Rik | RIKEN cDNA 4833420G17 gene (4833420G17Rik) | -1.75 |
| Rasa1 | RAS p21 protein activator 1 (Rasa1) | -1.75 |
| Tmem66 | transmembrane protein 66 (Tmem66) | -1.75 |
| Jmjd3 | jumonji domain containing 3 (Jmjd3) | -1.76 |
| Notch1 | Notch gene homolog 1 (Drosophila) (Notch1) | -1.77 |
| AA536749 | myosin phosphatase Rho interacting protein (Mprip) | -1.77 |
| Rgl2 | ral guanine nucleotide dissociation stimulator-like 2 (Rgl2) | -1.78 |
| Zfp238 | zinc finger protein 238 (Zfp238), transcript variant 1 | -1.78 |
| Ckap5 | cytoskeleton associated protein 5 (Ckap5) | -1.78 |
| Mllt3 | myeloid/lymphoid or mixed-lineage leukemia (trithorax homolog, Drosophila); translocated to, 3 (Mllt3), transcript variant 1 | -1.8 |
| Tfrc | transferrin receptor (Tfrc) | -1.8 |
| Tcof1 | Treacher Collins Franceschetti syndrome 1, homolog (Tcof1) | -1.8 |
| Zfp292 | zinc finger protein 292, transcript variant 4 (Zfp292) | -1.8 |
| Sfrs14 | splicing factor, arginine/serine-rich 14 (Sfrs14) | -1.8 |
| LOC100045005 | similar to Deltex3 (LOC100045005), misc RNA. | -1.8 |
| Slc25a37 | solute carrier family 25, member 37 (Slc25a37), nuclear gene encoding mitochondrial protein | -1.8 |
| Dap3 | death associated protein 3 (Dap3), nuclear gene encoding mitochondrial protein | -1.82 |
| Ager | advanced glycosylation end product-specific receptor (Ager) | -1.83 |
| Med24 | mediator complex subunit 24 (Med24) | -1.84 |
| Nasp | nuclear autoantigenic sperm protein (histone-binding) (Nasp), transcript variant 2 | -1.85 |
| Ercc5 | excision repair cross-complementing rodent repair deficiency, complementation group 5 (Ercc5) | -1.85 |
| Tfrc | transferrin receptor (Tfrc) | -1.85 |
| Anapc5 | anaphase-promoting complex subunit 5 (Anapc5), transcript variant 1 | -1.86 |
| Iap | CD47 antigen (Rh-related antigen, integrin-associated signal transducer) (Iap) | -1.86 |
| Snrp70 | U1 small nuclear ribonucleoprotein polypeptide A (Snrp70) | -1.86 |
| Slc29a1 | solute carrier family 29 (nucleoside transporters), member 1 (Slc29a1) | -1.86 |
| Pold3 | polymerase (DNA-directed), delta 3, accessory subunit (Pold3) | -1.87 |
| Pik3r1 | phosphatidylinositol 3-kinase, regulatory subunit, polypeptide 1 (p85 alpha) (Pik3r1), transcript variant 2 | -1.87 |
| Taf15 | TAF15 RNA polymerase II, TATA box binding protein (TBP)-associated factor (Taf15) | -1.87 |
| Impdh1 | inosine 5'-phosphate dehydrogenase 1 (Impdh1) | -1.88 |
| Slc9a3r1 | solute carrier family 9 (sodium/hydrogen exchanger), member 3 regulator 1 (Slc9a3r1) | -1.88 |
| Ankrd54 | ankyrin repeat domain 54 (Ankrd54) | -1.89 |
| Hdac2 | histone deacetylase 2 (Hdac2) | -1.89 |
| Dmtf1 | cyclin D binding myb-like transcription factor 1 (Dmtf1) | -1.89 |
| Cdk2 | cyclin-dependent kinase 2 (Cdk2), transcript variant 1 | -1.89 |
| Nrf1 | nuclear respiratory factor 1 (Nrf1) | -1.9 |
| Nipbl | Nipped-B homolog (Drosophila) (Nipbl), transcript variant A | -1.9 |
| Rcor1 | REST corepressor 1 (Rcor1) | -1.9 |
| Yeats2 | YEATS domain containing 2 (Yeats2) | -1.91 |
| Trpv2 | transient receptor potential cation channel, subfamily V, member 2 (Trpv2) | -1.91 |
| Helz | helicase with zinc finger domain (Helz) | -1.91 |
| Trim39 | tripartite motif-containing 39 (Trim39) | -1.92 |
| Jmjd1a | jumonji domain containing 1A (Jmjd1a), transcript variant 1 | -1.92 |
| Ctcf | CCCTC-binding factor (Ctcf) | -1.92 |
| Cnot2 | CCR4-NOT transcription complex, subunit 2 (Cnot2), transcript variant 3 | -1.93 |
| 1190002H23Rik | RIKEN cDNA 1190002H23 gene (1190002H23Rik) | -1.93 |
| Fas | Fas (TNF receptor superfamily member 6) (Fas) | -1.93 |
| Ints7 | integrator complex subunit 7 (Ints7) | -1.94 |
| 5930416I19Rik | RIKEN cDNA 5930416I19 gene (5930416I19Rik) | -1.94 |
| Ncaph2 | non-SMC condensin II complex, subunit H2 (Ncaph2) | -1.94 |
| Anxa2 | annexin A2 (Anxa2) | -1.94 |
| Cnot2 | CCR4-NOT transcription complex, subunit 2 (Cnot2), transcript variant 1 | -1.95 |
| Als2 | amyotrophic lateral sclerosis 2 (juvenile) homolog (human) (Als2) | -1.96 |
| Kctd2 | potassium channel tetramerisation domain containing 2 (Kctd2) | -1.96 |
| Btbd12 | BTB (POZ) domain containing 12 (Btbd12) | -1.97 |
| Rnasen | ribonuclease III, nuclear (Rnasen) | -1.98 |
| Gprasp1 | G protein-coupled receptor associated sorting protein 1 (Gprasp1), transcript variant 3 | -1.98 |
| Pstpip1 | proline-serine-threonine phosphatase-interacting protein 1 (Pstpip1) | -1.99 |
| Zfp251 | zinc finger protein 251 (Zfp251) | -2 |
| Fyb | FYN binding protein (Fyb) | -2 |
| Nrbp2 | nuclear receptor binding protein 2 (Nrbp2) | -2.01 |
| Saps3 | SAPS domain family, member 3 (Saps3) | -2.01 |
| Hdc | histidine decarboxylase (Hdc) | -2.01 |
| Ncapd3 | non-SMC condensin II complex, subunit D3 (Ncapd3) | -2.01 |
| Rbak | RB-associated KRAB repressor (Rbak), transcript variant 1 | -2.01 |
| Cd6 | CD6 antigen (Cd6), transcript variant 2 | -2.02 |
| Pdk1 | pyruvate dehydrogenase kinase, isoenzyme 1 (Pdk1), nuclear gene encoding mitochondrial protein | -2.02 |
| Zcchc8 | zinc finger, CCHC domain containing 8 (Zcchc8) | -2.03 |
| LOC100046039 | similar to histone deacetylase HD1 (LOC100046039) | -2.03 |
| Cstb | cystatin B (Cstb) | -2.04 |
| Lrig1 | leucine-rich repeats and immunoglobulin-like domains 1 (Lrig1) | -2.05 |
| Myst4 | MYST histone acetyltransferase monocytic leukemia 4 (Myst4) | -2.05 |
| Smarcd2 | SWI/SNF related, matrix associated, actin dependent regulator of chromatin, subfamily d, member 2 (Smarcd2) | -2.06 |
| 2610207I05Rik | RIKEN cDNA 2610207I05 gene (2610207I05Rik) | -2.07 |
| Trim56 | tripartite motif-containing 56 (Trim56) | -2.08 |
| Cd6 | CD6 antigen (Cd6), transcript variant 2 | -2.08 |
| Zcchc8 | zinc finger, CCHC domain containing 8 (Zcchc8) | -2.09 |
| Sla | src-like adaptor (Sla), transcript variant 2 | -2.1 |
| Rbmx | RNA binding motif protein, X chromosome (Rbmx) | -2.11 |
| Cnot2 | CCR4-NOT transcription complex, subunit 2 (Cnot2), transcript variant 3 | -2.11 |
| Metrn | meteorin, glial cell differentiation regulator (Metrn) | -2.11 |
| Mum1 | melanoma associated antigen (mutated) 1 (Mum1) | -2.12 |
| Zfp512 | zinc finger protein 512 (Zfp512) | -2.13 |
| BC057552 | cDNA sequence BC057552 (BC057552) | -2.13 |
| 4921505C17Rik | RIKEN cDNA 4921505C17 gene (4921505C17Rik) | -2.13 |
| Cd6 | CD6 antigen (Cd6), transcript variant 2 | -2.14 |
| Chfr | checkpoint with forkhead and ring finger domains (Chfr) | -2.15 |
| Ptprs | protein tyrosine phosphatase, receptor type, S (Ptprs) | -2.15 |
| Sfrs16 | CLK4-associating serine/arginine rich protein (Sfrs16) | -2.16 |
| Nfrkb | nuclear factor related to kappa B binding protein (Nfrkb) | -2.16 |
| Gmfg | glia maturation factor, gamma (Gmfg), transcript variant 1 | -2.16 |
| Ncaph2 | non-SMC condensin II complex, subunit H2 (Ncaph2) | -2.16 |
| Brf1 | BRF1 homolog, subunit of RNA polymerase III transcription initiation factor IIIB (S. cerevisiae) (Brf1) | -2.17 |
| Pik3r1 | phosphatidylinositol 3-kinase, regulatory subunit, polypeptide 1 (p85 alpha) (Pik3r1), transcript variant 1 | -2.17 |
| Tnrc6c | trinucleotide repeat containing 6C (Tnrc6c) | -2.18 |
| Abtb1 | ankyrin repeat and BTB (POZ) domain containing 1 (Abtb1) | -2.18 |
| Galnt10 | UDP-N-acetyl-alpha-D-galactosamine:polypeptide N-acetylgalactosaminyltransferase 10 (Galnt10) | -2.19 |
| 1110049F12Rik | RIKEN cDNA 1110049F12 gene (1110049F12Rik) | -2.21 |
| Snapc3 | small nuclear RNA activating complex, polypeptide 3 (Snapc3) | -2.21 |
| Anp32e | acidic (leucine-rich) nuclear phosphoprotein 32 family, member E (Anp32e) | -2.22 |
| Lbr | lamin B receptor (Lbr) | -2.23 |
| Phc1 | polyhomeotic-like 1 (Drosophila) (Phc1) | -2.23 |
| Zdhhc14 | zinc finger, DHHC domain containing 14 (Zdhhc14) | -2.23 |
| AA536749 | expressed sequence AA536749 (AA536749), transcript variant 1 | -2.24 |
| Dyrk1b | dual-specificity tyrosine-(Y)-phosphorylation regulated kinase 1b (Dyrk1b), transcript variant 1 | -2.25 |
| Gpc1 | glypican 1 (Gpc1) | -2.25 |
| Cenpa | centromere protein A (Cenpa) | -2.28 |
| Zap70 | zeta-chain (TCR) associated protein kinase (Zap70) | -2.28 |
| Vars2 | valyl-tRNA synthetase 2, mitochondrial (putative) (Vars2) | -2.29 |
| Birc5 | baculoviral IAP repeat-containing 5 (Birc5), transcript variant 1 | -2.3 |
| Cdan1 | congenital dyserythropoietic anemia, type I (human) (Cdan1) | -2.31 |
| Tbcel | tubulin folding cofactor E-like (Tbcel) | -2.31 |
| Dnmt1 | DNA methyltransferase (cytosine-5) 1 (Dnmt1) | -2.33 |
| 5830405N20Rik | RIKEN cDNA 5830405N20 gene (5830405N20Rik) | -2.33 |
| Zap70 | zeta-chain (TCR) associated protein kinase (Zap70) | -2.33 |
| Aff1 | AF4/FMR2 family, member 1 (Aff1), transcript variant 2 | -2.36 |
| Aprin | androgen-induced proliferation inhibitor (Aprin) | -2.36 |
| D16Ertd472e | DNA segment, Chr 16, ERATO Doi 472, expressed (D16Ertd472e) | -2.36 |
| Hmgb2 | high mobility group box 2 (Hmgb2) | -2.37 |
| Gmfg | glia maturation factor, gamma (Gmfg), transcript variant 1 | -2.37 |
| 1500011H22Rik | RIKEN cDNA 1500011H22 gene (1500011H22Rik) | -2.38 |
| Pou6f1 | POU domain, class 6, transcription factor 1 (Pou6f1) | -2.39 |
| Egr1 | early growth response 1 (Egr1) | -2.39 |
| AI467606 | expressed sequence AI467606 (AI467606) | -2.39 |
| 2310061F22Rik | RIKEN cDNA 2310061F22 gene (2310061F22Rik) | -2.4 |
| Hnrpll | heterogeneous nuclear ribonucleoprotein L-like (Hnrpll) | -2.41 |
| Bcl6 | B-cell leukemia/lymphoma 6 (Bcl6) | -2.42 |
| Lig1 | ligase I, DNA, ATP-dependent (Lig1), transcript variant 2 | -2.43 |
| Uspl1 | ubiquitin specific peptidase like 1 (Uspl1) | -2.44 |
| Hmha1 | histocompatibility (minor) HA-1 (Hmha1) | -2.45 |
| Cd3d | CD3 antigen, delta polypeptide (Cd3d) | -2.48 |
| Nfyb | nuclear transcription factor-Y beta (Nfyb) | -2.48 |
| 4930572J05Rik | RIKEN cDNA 4930572J05 gene (4930572J05Rik) | -2.48 |
| Hist1h2af | histone cluster 1, H2af (Hist1h2af) | -2.48 |
| Spsb3 | splA/ryanodine receptor domain and SOCS box containing 3 (Spsb3) | -2.5 |
| Cdk2 | cyclin-dependent kinase 2 (Cdk2), transcript variant 1 | -2.53 |
| Ddx17 | DEAD (Asp-Glu-Ala-Asp) box polypeptide 17 (Ddx17), transcript variant 1 | -2.54 |
| Hist1h2ad | histone cluster 1, H2ad (Hist1h2ad) | -2.56 |
| Def6 | differentially expressed in FDCP 6 (Def6) | -2.56 |
| Csnk1e | casein kinase 1, epsilon (Csnk1e) | -2.58 |
| Akap12 | A kinase (PRKA) anchor protein (gravin) 12 (Akap12) | -2.58 |
| Cenpl | centromere protein L (Cenpl) | -2.58 |
| Cd3e | CD3 antigen, epsilon polypeptide (Cd3e) | -2.59 |
| Gse1 | genetic suppressor element 1 (Gse1) | -2.62 |
| Rcsd1 | RCSD domain containing 1 (Rcsd1), transcript variant 1 | -2.63 |
| Bhlhb9 | basic helix-loop-helix domain containing, class B9 (Bhlhb9) | -2.64 |
| Lip1 | lysosomal acid lipase A (Lip1) | -2.65 |
| Jakmip1 | janus kinase and microtubule interacting protein 1 (Jakmip1) | -2.66 |
| A130092J06Rik | RIKEN cDNA A130092J06 gene (A130092J06Rik) | -2.67 |
| Tpcn1 | two pore channel 1 (Tpcn1) | -2.67 |
| EG434197 | predicted gene, EG434197 (EG434197) | -2.68 |
| Csnk1e | casein kinase 1, epsilon (Csnk1e) | -2.68 |
| Hist1h2an | histone cluster 1, H2an (Hist1h2an) | -2.69 |
| Cdc20 | cell division cycle 20 homolog (S. cerevisiae) (Cdc20) | -2.7 |
| D230007K08Rik | RIKEN cDNA D230007K08 gene, transcript variant 5 (D230007K08Rik) | -2.7 |
| Prc1 | protein regulator of cytokinesis 1 (Prc1) | -2.72 |
| Gas6 | growth arrest specific 6 (Gas6) | -2.72 |
| Dgkz | diacylglycerol kinase zeta (Dgkz) | -2.73 |
| Arid2 | AT rich interactive domain 2 (Arid-rfx like) (Arid2) | -2.73 |
| Dgkz | diacylglycerol kinase zeta (Dgkz) | -2.74 |
| Prep | prolyl endopeptidase (Prep) | -2.75 |
| Lman2l | lectin, mannose-binding 2-like (Lman2l) | -2.77 |
| Cdca3 | cell division cycle associated 3 (Cdca3) | -2.77 |
| Acot1 | acyl-CoA thioesterase 1 (Acot1) | -2.79 |
| Ilvbl | ilvB (bacterial acetolactate synthase)-like (Ilvbl) | -2.8 |
| Cenpl | centromere protein L (Cenpl) | -2.82 |
| Dok2 | docking protein 2 (Dok2) | -2.85 |
| Tuft1 | tuftelin 1 (Tuft1) | -2.85 |
| Lman2l | lectin, mannose-binding 2-like (Lman2l) | -2.85 |
| Tubb2b | tubulin, beta 2b (Tubb2b) | -2.86 |
| Abcc5 | ATP-binding cassette, sub-family C (CFTR/MRP), member 5 (Abcc5), transcript variant 2 | -2.87 |
| Itpr2 | inositol 1,4,5-triphosphate receptor 2 (Itpr2), transcript variant 1 | -2.9 |
| Nfatc3 | nuclear factor of activated T-cells, cytoplasmic, calcineurin-dependent 3 (Nfatc3) | -2.9 |
| LOC100044475 | similar to SH2/SH3 adaptor protein (LOC100044475) | -2.9 |
| Agtrap | angiotensin II, type I receptor-associated protein (Agtrap) | -2.91 |
| Bsdc1 | BSD domain containing 1 (Bsdc1) | -2.92 |
| Hist1h2ak | histone cluster 1, H2ak (Hist1h2ak) | -2.92 |
| Cd3g | CD3 antigen, gamma polypeptide (Cd3g) | -2.94 |
| Hist1h2ah | histone cluster 1, H2ah (Hist1h2ah) | -2.95 |
| Hey1 | hairy/enhancer-of-split related with YRPW motif 1 (Hey1) | -2.95 |
| Atp1b1 | ATPase, Na+/K+ transporting, beta 1 polypeptide (Atp1b1) | -2.95 |
| Tbc1d10c | TBC1 domain family, member 10c (Tbc1d10c) | -2.97 |
| Lmnb1 | lamin B1 (Lmnb1) | -2.97 |
| Cecr5 | cat eye syndrome chromosome region, candidate 5 homolog (human) (Cecr5) | -2.98 |
| Psg23 | pregnancy-specific glycoprotein 23 (Psg23) | -2.99 |
| Prep | prolyl endopeptidase (Prep) | -3.02 |
| Oasl2 | 2'-5' oligoadenylate synthetase-like 2 (Oasl2) | -3.05 |
| Hsdl1 | hydroxysteroid dehydrogenase like 1 (Hsdl1) | -3.07 |
| P2ry5 | purinergic receptor P2Y, G-protein coupled, 5 (P2ry5) | -3.07 |
| Itk | IL2-inducible T-cell kinase (Itk) | -3.07 |
| Ilvbl | ilvB (bacterial acetolactate synthase)-like (Ilvbl) | -3.09 |
| Cdc7 | cell division cycle 7 (S. cerevisiae) (Cdc7) | -3.1 |
| BC021381 | cDNA sequence BC021381 (BC021381) | -3.12 |
| H2afx | H2A histone family, member X (H2afx) | -3.13 |
| Msh6 | mutS homolog 6 (E. coli) (Msh6) | -3.13 |
| Dmn | desmuslin (Dmn), transcript variant 3 | -3.14 |
| LOC100044862 | similar to Fbxl3 protein (LOC100044862) | -3.17 |
| Msh6 | mutS homolog 6 (E. coli) (Msh6) | -3.18 |
| Zfp579 | zinc finger protein 579 (Zfp579) | -3.2 |
| Pitpnm2 | phosphatidylinositol transfer protein, membrane-associated 2 (Pitpnm2) | -3.21 |
| Ssbp3 | single-stranded DNA binding protein 3 (Ssbp3), transcript variant 1 | -3.22 |
| Phf2 | PHD finger protein 2 (Phf2) | -3.26 |
| LOC100048845 | similar to CD28 antigen (LOC100048845) | -3.28 |
| Hdac7 | histone deacetylase 7 (Hdac7) | -3.3 |
| Map3k7ip1 | mitogen-activated protein kinase kinase kinase 7 interacting protein 1 (Map3k7ip1) | -3.33 |
| Hist1h2ag | histone cluster 1, H2ag (Hist1h2ag) | -3.33 |
| Uhrf1 | ubiquitin-like, containing PHD and RING finger domains, 1 (Uhrf1) | -3.35 |
| Tapt1 | transmembrane anterior posterior transformation 1 (Tapt1) | -3.37 |
| Nusap1 | nucleolar and spindle associated protein 1 (Nusap1), transcript variant 2 | -3.39 |
| Rasgrp1 | RAS guanyl releasing protein 1 (Rasgrp1) | -3.42 |
| Ezh2 | enhancer of zeste homolog 2 (Drosophila) (Ezh2) | -3.42 |
| Adcy6 | adenylate cyclase 6 (Adcy6) | -3.46 |
| Satb1 | special AT-rich sequence binding protein 1 (Satb1) | -3.48 |
| Fbxl12 | F-box and leucine-rich repeat protein 12 (Fbxl12), transcript variant 1 | -3.5 |
| Lck | lymphocyte protein tyrosine kinase (Lck) | -3.51 |
| D15Wsu75e | DNA segment, Chr 15, Wayne State University 75, expressed (D15Wsu75e) | -3.52 |
| Bach1 | BTB and CNC homology 1 (Bach1) | -3.53 |
| E2f2 | E2F transcription factor 2 (E2f2) | -3.56 |
| Ccnd3 | cyclin D3 (Ccnd3), transcript variant 1 | -3.6 |
| Cd8b1 | CD8 antigen, beta chain 1 (Cd8b1) | -3.61 |
| Kif22 | kinesin family member 22 (Kif22) | -3.62 |
| Cd27 | CD27 antigen (Cd27), transcript variant 1 | -3.7 |
| Cyp2f2 | cytochrome P450, family 2, subfamily f, polypeptide 2 (Cyp2f2) | -3.7 |
| Mier1 | mesoderm induction early response 1 homolog (Xenopus laevis (Mier1), transcript variant 1 | -3.71 |
| Clk3 | CDC-like kinase 3 (Clk3) | -3.72 |
| Thy1 | thymus cell antigen 1, theta (Thy1) | -3.83 |
| E2f2 | E2F transcription factor 2 (E2f2) | -3.86 |
| Hrbl | HIV-1 Rev binding protein-like (Hrbl), transcript variant 2 | -3.97 |
| Tubb2b | tubulin, beta 2b (Tubb2b) | -4.13 |
| Lat | linker for activation of T cells (Lat) | -4.16 |
| Pard6g | par-6 partitioning defective 6 homolog gamma (C. elegans) (Pard6g) | -4.28 |
| Ets2 | E26 avian leukemia oncogene 2, 3' domain (Ets2) | -4.29 |
| 6430706D22Rik | RIKEN cDNA 6430706D22 gene (6430706D22Rik) | -4.29 |
| Emb | embigin (Emb) | -4.42 |
| Rasl11b | RAS-like, family 11, member B (Rasl11b) | -4.54 |
| Xrcc6 | X-ray repair complementing defective repair in Chinese hamster cells 6 (Xrcc6) | -4.57 |
| Bcl11b | B-cell leukemia/lymphoma 11B (Bcl11b), transcript variant 2 | -4.77 |
| Rasl11b | RAS-like, family 11, member B (Rasl11b) | -4.79 |
| Mgst2 | microsomal glutathione S-transferase 2 (Mgst2) | -4.8 |
| Cd8b1 | CD8 antigen, beta chain 1 (Cd8b1) | -4.82 |
| Ramp1 | receptor (calcitonin) activity modifying protein 1 (Ramp1) | -4.94 |
| Cdca7 | cell division cycle associated 7 (Cdca7) | -5 |
| Gtf2h4 | general transcription factor II H, polypeptide 4 (Gtf2h4) | -5.13 |
| Mns1 | meiosis-specific nuclear structural protein 1 (Mns1) | -6.32 |
| Cbr2 | carbonyl reductase 2 (Cbr2) | -6.85 |
| Wfdc2 | WAP four-disulfide core domain 2 (Wfdc2) | -8.92 |
