# Supplementary material for: Genomic deletion of GIT2 induces a premature age-related thymic dysfunction and systemic immune system disruption
Source: Aging (Albany NY). 2017 Mar 4;9(3):706–30. doi: 10.18632/aging.101185 (PMC5391227; doi:10.18632/aging.101185)
Supplement: Supplementary file 7 [file aging-09-706-s007.docx]

**Table S6. Venn diagram analysis of GIT2KO parathymic lobe (PTL) transcriptomic profiles.** Table represents the expression patterns generated using a three-way quantitative Venn diagram approach. For each specific transcript, identified by its Official Gene Symbol, the normalized z ratios for the following comparisons are indicated: GIT2KO PTL vs. GIT2KO thymus; PTL vs. WT thymus and the GIT2KO thymus vs. WT thymus. Transcripts annotated with an asterisk represent the 30 common, contra-regulated transcripts associated with idiosyncratic PTL function.

| **Gene Symbol** | **PTL vs. GIT2KO thymus** | **PTL vs. WT thymus** | **GIT2KO thymus vs. WT thymus** |
| --- | --- | --- | --- |
| Cyp4f18* | 4.83 | 3.12 | -2.57 |
| Hmgcs2* | 4.72 | 3.24 | -2.17 |
| Hvcn1* | 4.52 | 3.11 | -2.06 |
| Btla* | 4.29 | 3.17 | -1.55 |
| Tgfbi* | 4.15 | 3.01 | -1.6 |
| Prcp* | 3.93 | 2.85 | -1.53 |
| Gvin1* | 3.91 | 2.34 | -2.41 |
| Ccnd1* | 3.69 | 2.62 | -1.59 |
| Psen2* | 3.65 | 1.9 | -2.76 |
| Pld4* | 3.5 | 2.42 | -1.57 |
| Prg2* | 3.45 | 2.38 | -1.57 |
| Slc2a6* | 3.4 | 1.97 | -2.22 |
| Bach2* | 3.22 | 1.8 | -2.2 |
| Kcnk6* | 3.13 | 1.58 | -2.45 |
| Irf8* | 3.06 | 1.87 | -1.81 |
| Ehd1* | 3.03 | 1.74 | -2 |
| 9130422G05Rik* | 2.98 | 1.62 | -2.14 |
| Gadd45g* | 2.94 | 1.87 | -1.61 |
| Itgb7* | 2.9 | 1.63 | -1.98 |
| Cd44* | 2.89 | 1.64 | -1.95 |
| Hsp90b1* | 2.88 | 1.73 | -1.77 |
| H2-DMb1* | 2.83 | 1.76 | -1.63 |
| Cybasc3* | 2.66 | 1.58 | -1.67 |
| Herpud1* | 2.61 | 1.66 | -1.9 |
| Pecam1* | 2.33 | 1.76 | -2.61 |
| Irf1* | 2.23 | 1.64 | -1.66 |
| Fxyd5* | 1.89 | 1.57 | -1.63 |
| Chmp1b* | -2.98 | -1.69 | 2.01 |
| H2-T10* | -3.56 | 2.39 | 10.36 |
| Hmgb2* | -3.57 | -2.37 | 1.78 |
|  |  |  |  |
| Igfbp4 | 2.75 | 3.71 | 2.04 |
| Arhgap29 | 2.56 | 3.58 | 2.12 |
| Sparc | 2.42 | 4.32 | 4.29 |
| Ifitm2 | 2.41 | 3.38 | 2 |
| Dcn | 2.39 | 3.32 | 2.73 |
| Aebp1 | 2.33 | 3.63 | 2.6 |
| Rarres2 | 2.32 | 4.19 | 3.64 |
| Vim | 2.24 | 3.17 | 1.94 |
| Col4a1 | 2.23 | 3.09 | 1.8 |
| Ednrb | 2.22 | 3.34 | 2.27 |
| Brp17 | 2.09 | 3.13 | 2.11 |
| Slc9a3r2 | 1.92 | 2.72 | 1.66 |
| Hp | 1.9 | 6.37 | 8.67 |
| Loxl1 | 1.89 | 3.59 | 3.28 |
| Prkcdbp | 1.81 | 2.55 | 1.53 |
| Gpx1 | 1.81 | 2.67 | 1.77 |
| Nme2 | 1.77 | 2.94 | 2.5 |
| Vkorc1 | 1.74 | 2.32 | 1.58 |
| Tgfbr2 | 1.71 | 2.68 | 1.94 |
| Rps6 | 1.58 | 2.53 | 1.89 |
| Rps2 | 1.55 | 2.31 | 1.54 |
| Fkbp2 | 1.52 | 2.64 | 2.2 |
|  |  |  |  |
| Oasl2 | -1.51 | -3.05 | -2.95 |
| Gmfg | -1.53 | -2.16 | -1.75 |
| BC021381 | -1.55 | -3.12 | -3 |
| Itpr2 | -1.57 | -2.9 | -2.58 |
| Zcchc8 | -1.68 | -2.03 | -1.68 |
| Dgkz | -1.74 | -2.73 | -1.96 |
| Acot1 | -1.74 | -2.79 | -2.1 |
| Dmn | -1.81 | -3.14 | -2.59 |
| 6430706D22Rik | -1.85 | -4.29 | -4.61 |
| EG434197 | -1.86 | -2.68 | -1.7 |
| Gas6 | -1.92 | -2.72 | -1.65 |
| Dok2 | -2 | -2.85 | -1.76 |
| Prep | -2.01 | -2.75 | -1.55 |
| Abcc5 | -2.02 | -2.87 | -1.75 |
| Zfp579 | -2.08 | -3.2 | -2.24 |
| Map3k7ip1 | -2.17 | -3.33 | -2.33 |
| Nfatc3 | -2.19 | -2.9 | -1.51 |
| Ssbp3 | -2.2 | -3.22 | -2.08 |
| Hsdl1 | -2.22 | -3.07 | -1.77 |
| Bach1 | -2.27 | -3.53 | -2.52 |
| Adcy6 | -2.36 | -3.46 | -2.26 |
| Pitpnm2 | -2.41 | -3.21 | -1.7 |
| Tubb2b | -2.61 | -2.86 | -1.94 |
| Clk3 | -2.86 | -3.72 | -1.86 |
| Gtf2h4 | -4 | -5.13 | -2.47 |
| Bcl11b | -4.03 | -4.77 | -1.76 |
|  |  |  |  |
| Tmem66 |  | -1.75 | 3.73 |
|  |  |  |  |
| Ctse |  | 5.57 | 9.86 |
| Ndg2 |  | 4.26 | 4.59 |
| Mgst1 |  | 4.04 | 5.76 |
| S100a8 |  | 4.02 | 4.7 |
| Hba-a1 |  | 3.84 | 8.33 |
| Rasl12 |  | 3.74 | 5.74 |
| Lpl |  | 3.69 | 5.91 |
| Cav1 |  | 3.65 | 5.37 |
| Sdpr |  | 3.37 | 3.64 |
| Eif4ebp1 |  | 3.29 | 3.56 |
| Aoc3 |  | 3.28 | 5.57 |
| Ndn |  | 3.27 | 3.6 |
| Dci |  | 3.16 | 4.21 |
| Cd59a |  | 3.08 | 4.23 |
| Pnpla2 |  | 2.82 | 4.24 |
| Pygl |  | 2.75 | 4.39 |
| Gng10 |  | 2.69 | 3.87 |
| Eno1 |  | 2.69 | 4.29 |
| Dbi |  | 2.62 | 3.7 |
| LOC100047934 |  | 2.61 | 3.19 |
| H2-K1 |  | 2.6 | 3.68 |
| Hspa8 |  | 2.59 | 4.89 |
| Tmem38b |  | 2.46 | 2.08 |
| Actb |  | 2.34 | 3.12 |
| Mylc2b |  | 2.3 | 2.31 |
| Pik3cg |  | 2.26 | 3.48 |
| Sec61b |  | 2.25 | 1.73 |
| Serpinf1 |  | 2.22 | 2.1 |
| Mid1ip1 |  | 2.21 | 2.18 |
| Gstm1 |  | 2.2 | 3.47 |
| Eef2 |  | 2.18 | 2.26 |
| Cpt2 |  | 2.17 | 3.55 |
| Aldoa |  | 2.13 | 2.1 |
| Cxcl12 |  | 2.11 | 3.19 |
| Idh1 |  | 2.06 | 3.33 |
| Inppl1 |  | 2.01 | 1.51 |
| Idh3g |  | 1.99 | 4.32 |
| Sod2 |  | 1.99 | 2.41 |
| Ywhag |  | 1.94 | 3.18 |
| Suclg1 |  | 1.93 | 2.51 |
| Fmo1 |  | 1.93 | 3.62 |
| Gja1 |  | 1.93 | 3.53 |
| Pfkl |  | 1.91 | 3.08 |
| Oxct1 |  | 1.89 | 2.2 |
| Adi1 |  | 1.88 | 5.38 |
| Spcs1 |  | 1.86 | 2.53 |
| Gfer |  | 1.81 | 2.55 |
| Atp5l |  | 1.8 | 1.82 |
| 1700037H04Rik |  | 1.78 | 3.39 |
| Ppp1ca |  | 1.77 | 3.51 |
| Prelp |  | 1.76 | 3.33 |
| Tmem126a |  | 1.76 | 1.57 |
| Tmem33 |  | 1.75 | 2.99 |
| Ptp4a2 |  | 1.73 | 3.07 |
| Rnase4 |  | 1.72 | 2.89 |
| Ndufc1 |  | 1.71 | 3.1 |
| Pfn1 |  | 1.66 | 2.59 |
| MGC18837 |  | 1.66 | 2.02 |
| 2310016E02Rik |  | 1.65 | 1.72 |
| Gstp1 |  | 1.65 | 2.15 |
| Msn |  | 1.65 | 2.64 |
| Dph3 |  | 1.65 | 2.2 |
| Cox6a1 |  | 1.64 | 2.77 |
| Csrp2 |  | 1.64 | 1.72 |
| Cd151 |  | 1.63 | 2.68 |
| Adk |  | 1.61 | 2.68 |
| Yif1a |  | 1.61 | 1.77 |
| Gnas |  | 1.6 | 2.63 |
| Kras |  | 1.6 | 4.16 |
| Ndufb10 |  | 1.6 | 2.44 |
| Tspo |  | 1.58 | 2.46 |
| Sdhb |  | 1.57 | 3.42 |
| Cox6b1 |  | 1.57 | 2.54 |
| Slc25a1 |  | 1.55 | 2.63 |
| Gpx4 |  | 1.54 | 2.26 |
| Sypl |  | 1.53 | 2.56 |
| Cox5a |  | 1.53 | 2.34 |
| Tuba1a |  | 1.52 | 2.24 |
| Rnaset2 |  | 1.52 | 2.21 |
| Ndufb6 |  | 1.52 | 3.24 |
| Atp5f1 |  | 1.51 | 3.64 |
|  |  |  |  |
| Ttc14 |  | -1.5 | -1.97 |
| Scrib |  | -1.51 | -1.78 |
| Atp9b |  | -1.51 | -2.05 |
| Rnf145 |  | -1.51 | -1.66 |
| Tmc6 |  | -1.52 | -2.34 |
| BC017643 |  | -1.55 | -2.72 |
| Utx |  | -1.55 | -1.56 |
| Lyrm2 |  | -1.55 | -1.61 |
| Ppp1r9b |  | -1.55 | -1.85 |
| Dscr1l2 |  | -1.55 | -1.87 |
| Ubtf |  | -1.56 | -2.01 |
| Rgl2 |  | -1.56 | -2.17 |
| Usp52 |  | -1.56 | -1.83 |
| Zmym3 |  | -1.57 | -1.99 |
| Vav1 |  | -1.57 | -2.02 |
| Mark3 |  | -1.57 | -1.98 |
| Slc4a2 |  | -1.58 | -1.68 |
| 1810073G14Rik |  | -1.58 | -2.73 |
| 9-Sep |  | -1.58 | -2.05 |
| Arhgap4 |  | -1.59 | -2.55 |
| Dbp |  | -1.61 | -3.35 |
| LOC676420 |  | -1.62 | -1.94 |
| Slc44a2 |  | -1.63 | -2.27 |
| LOC100047369 |  | -1.63 | -1.74 |
| A430107D22Rik |  | -1.63 | -1.79 |
| LOC545056 |  | -1.64 | -2.63 |
| Nxf1 |  | -1.64 | -2.04 |
| Helb |  | -1.65 | -1.62 |
| Akap8l |  | -1.65 | -1.52 |
| Csnk1g2 |  | -1.65 | -2.06 |
| Ldb1 |  | -1.66 | -1.58 |
| Peci |  | -1.66 | -1.59 |
| Dgka |  | -1.67 | -1.9 |
| Purb |  | -1.68 | -2.7 |
| Sin3a |  | -1.68 | -2.55 |
| Mdm2 |  | -1.68 | -1.86 |
| Brd9 |  | -1.68 | -1.97 |
| Lysmd1 |  | -1.69 | -1.59 |
| Bat2 |  | -1.69 | -2.31 |
| Zbtb17 |  | -1.7 | -1.53 |
| Ars2 |  | -1.7 | -1.74 |
| Rab27a |  | -1.71 | -2.65 |
| Map4k2 |  | -1.72 | -1.87 |
| AI450540 |  | -1.72 | -1.72 |
| Gtf2h1 |  | -1.72 | -2.39 |
| Cdkn2aipnl |  | -1.73 | -1.67 |
| Stk11 |  | -1.73 | -2.65 |
| Scarf2 |  | -1.74 | -1.92 |
| Dcp1b |  | -1.74 | -1.59 |
| 4833420G17Rik |  | -1.75 | -1.57 |
| Notch1 |  | -1.77 | -1.75 |
| Tcof1 |  | -1.8 | -2.37 |
| Sfrs14 |  | -1.8 | -1.86 |
| LOC100045005 |  | -1.8 | -1.68 |
| Slc25a37 |  | -1.8 | -2.28 |
| Ager |  | -1.83 | -2.4 |
| Ercc5 |  | -1.85 | -2.49 |
| Iap |  | -1.86 | -2.18 |
| Snrp70 |  | -1.86 | -1.62 |
| Pold3 |  | -1.87 | -2.37 |
| Pik3r1 |  | -1.87 | -2.1 |
| Taf15 |  | -1.87 | -2.48 |
| Ankrd54 |  | -1.89 | -1.97 |
| Trpv2 |  | -1.91 | -2.08 |
| Trim39 |  | -1.92 | -2.14 |
| Kctd2 |  | -1.96 | -1.72 |
| Btbd12 |  | -1.97 | -1.81 |
| Pstpip1 |  | -1.99 | -1.52 |
| Nrbp2 |  | -2.01 | -2.14 |
| Saps3 |  | -2.01 | -2.09 |
| Rbak |  | -2.01 | -1.66 |
| Myst4 |  | -2.05 | -2.35 |
| 2610207I05Rik |  | -2.07 | -2.41 |
| Trim56 |  | -2.08 | -1.61 |
| Mum1 |  | -2.12 | -2.23 |
| BC057552 |  | -2.13 | -2.56 |
| Ptprs |  | -2.15 | -2.78 |
| Sfrs16 |  | -2.16 | -2.95 |
| Nfrkb |  | -2.16 | -1.64 |
| Brf1 |  | -2.17 | -2.05 |
| Tnrc6c |  | -2.18 | -1.53 |
| Galnt10 |  | -2.19 | -3.06 |
| Snapc3 |  | -2.21 | -2.15 |
| Gpc1 |  | -2.25 | -1.61 |
| Vars2 |  | -2.29 | -1.72 |
| AI467606 |  | -2.39 | -1.96 |
| 2310061F22Rik |  | -2.4 | -2.11 |
| Hmha1 |  | -2.45 | -2.92 |
| Nfyb |  | -2.48 | -2.14 |
| Spsb3 |  | -2.5 | -2.12 |
| Rcsd1 |  | -2.63 | -2.56 |
| D230007K08Rik |  | -2.7 | -3.31 |
| Bsdc1 |  | -2.92 | -5.89 |
| Cecr5 |  | -2.98 | -3.3 |
| Psg23 |  | -2.99 | -3.43 |
| LOC100044862 |  | -3.17 | -5.08 |
| Emb |  | -4.42 | -10.7 |
| Mgst2 |  | -4.8 | -7.34 |
|  |  |  |  |
| Rgs1 | 3.02 |  | -2.54 |
| Edem2 | 3 |  | -1.76 |
| Sirpa | 2.92 |  | -2.99 |
| Ctgf | 2.84 |  | -2.54 |
| Traf1 | 2.82 |  | -2.89 |
| Gpr114 | 2.79 |  | -3.21 |
| Il18r1 | 2.78 |  | -2.35 |
| Cd86 | 2.69 |  | -2.1 |
| Hap1 | 2.67 |  | -2.37 |
| Syvn1 | 2.64 |  | -2.02 |
| Klk8 | 2.56 |  | -2.54 |
| LOC100045780 | 2.5 |  | -1.71 |
| Dusp7 | 2.46 |  | -2.67 |
| Tmem23 | 2.35 |  | -1.63 |
| Ly6d | 2.35 |  | -2.31 |
| Relb | 2.34 |  | -1.82 |
| Slc24a6 | 2.34 |  | -1.58 |
| Amica1 | 2.29 |  | -3.15 |
| Ptpn1 | 2.27 |  | -2.78 |
| Tnfrsf4 | 2.22 |  | -3.83 |
| Arhgap17 | 2.21 |  | -1.76 |
| Rpl29 | 2.1 |  | -2.47 |
| Ptpre | 2.09 |  | -1.71 |
| Gba | 2.08 |  | -1.84 |
| Junb | 2.05 |  | -1.78 |
| Erp29 | 2.03 |  | -1.79 |
| Slc35c2 | 2.02 |  | -1.99 |
| Pik3cd | 1.99 |  | -2.31 |
| Dkk3 | 1.99 |  | -3.21 |
| LOC100048445 | 1.98 |  | -1.6 |
| Mapk11 | 1.96 |  | -2.05 |
| Psmb9 | 1.86 |  | -1.94 |
| Psap | 1.84 |  | -1.68 |
| Cyp4f13 | 1.8 |  | -2.92 |
| Drctnnb1a | 1.8 |  | -2.38 |
| Hcst | 1.79 |  | -1.72 |
| C230093N12Rik | 1.76 |  | -1.85 |
| Ccnd2 | 1.75 |  | -1.67 |
| Epsti1 | 1.71 |  | -3.37 |
| Ube1l | 1.69 |  | -2.23 |
| Cbx7 | 1.68 |  | -1.9 |
| Atp6ap1 | 1.67 |  | -1.69 |
| Mif4gd | 1.64 |  | -1.53 |
| Cdipt | 1.62 |  | -1.66 |
| Il6st | 1.61 |  | -1.54 |
| Ndrg1 | 1.61 |  | -1.55 |
| Tap1 | 1.6 |  | -2.2 |
| Rag1ap1 | 1.59 |  | -1.78 |
| Vars | 1.57 |  | -2.15 |
| Akna | 1.57 |  | -1.77 |
| Trabd | 1.56 |  | -1.89 |
| Icos | 1.54 |  | -2.47 |
| Birc2 | 1.53 |  | -1.94 |
| Tcirg1 | 1.51 |  | -2.72 |
| Gsdmdc1 | 1.5 |  | -1.93 |
| Gimap6 | 1.5 |  | -1.86 |
| Stat1 | 1.5 |  | -1.63 |
| Drg1 | -1.51 |  | 2.23 |
| Ube2g1 | -1.52 |  | 2.63 |
| Mrpl3 | -1.54 |  | 2.93 |
| Rb1 | -1.54 |  | 1.92 |
| B230219D22Rik | -1.61 |  | 1.99 |
| Snrpd1 | -1.61 |  | 1.93 |
| Hnrnpa2b1 | -1.7 |  | 2.7 |
| H2afz | -1.73 |  | 1.92 |
| Mcm5 | -1.75 |  | 1.69 |
| Emp1 | -1.78 |  | 3.09 |
| Plekhf2 | -1.89 |  | 1.54 |
| Zeb1 | -2 |  | 1.91 |
| Rpa3 | -2.21 |  | 1.96 |
| Orc6l | -2.33 |  | 1.52 |
| Tipin | -2.36 |  | 1.93 |
| Cbr3 | -2.83 |  | 5.04 |
| Mcm6 | -3.05 |  | 2.6 |
|  |  |  |  |
| Cd209b | 9.54 | 9.09 |  |
| Cxcl13 | 9.07 | 7.98 |  |
| LOC100046120 | 7.13 | 6.39 |  |
| Serpina3f | 6.74 | 5.16 |  |
| Ccl19 | 6.39 | 6.66 |  |
| Serpina3n | 6.26 | 4.61 |  |
| Faim3 | 6.12 | 5.18 |  |
| 2010001M09Rik | 6.07 | 4.18 |  |
| Mfge8 | 6.07 | 5.11 |  |
| Plvap | 6.06 | 5.65 |  |
| Cpxm1 | 6.05 | 5.12 |  |
| Cd22 | 6.01 | 4.55 |  |
| Cd79b | 5.96 | 5.59 |  |
| LOC100047815 | 5.9 | 5.26 |  |
| Slpi | 5.9 | 6.26 |  |
| Pou2af1 | 5.8 | 4.9 |  |
| Tnfsf13b | 5.78 | 5.23 |  |
| Cd55 | 5.61 | 5.69 |  |
| Tnfrsf13c | 5.58 | 4.73 |  |
| Chst3 | 5.53 | 5.02 |  |
| Srpk3 | 5.49 | 4.59 |  |
| Il4i1 | 5.48 | 3.79 |  |
| Napsa | 5.38 | 4.29 |  |
| Cxcr5 | 5.27 | 4.34 |  |
| Serpina3g | 5.19 | 3.41 |  |
| Ccr6 | 5.19 | 3.23 |  |
| H2-Ob | 5.18 | 4.01 |  |
| Txndc5 | 5.13 | 4.42 |  |
| Fcrla | 5.12 | 4.12 |  |
| LOC100044439 | 5.08 | 3.59 |  |
| Lrg1 | 4.99 | 5.14 |  |
| Mef2c | 4.92 | 4.32 |  |
| Lyzs | 4.86 | 4.68 |  |
| Btk | 4.75 | 3.69 |  |
| Dok3 | 4.69 | 4.01 |  |
| Ccl21c | 4.68 | 4.51 |  |
| Igfbp7 | 4.67 | 4.48 |  |
| C4b | 4.65 | 5.18 |  |
| Serping1 | 4.63 | 5.49 |  |
| LOC100041504 | 4.6 | 3.99 |  |
| Lmo2 | 4.57 | 4.73 |  |
| Gpr18 | 4.57 | 3.8 |  |
| Il33 | 4.57 | 3.97 |  |
| LOC100048721 | 4.5 | 3.96 |  |
| Enpp2 | 4.5 | 3.51 |  |
| Ly86 | 4.5 | 3.4 |  |
| Lyz2 | 4.48 | 5.72 |  |
| Zcchc18 | 4.44 | 3.63 |  |
| Plcg2 | 4.44 | 3.65 |  |
| Cxcl9 | 4.38 | 3.97 |  |
| Chst1 | 4.33 | 3.84 |  |
| Ramp2 | 4.32 | 4.02 |  |
| Ncf4 | 4.31 | 3.22 |  |
| Reln | 4.3 | 4.23 |  |
| Flrt3 | 4.22 | 4.14 |  |
| Rasgrp3 | 4.2 | 3.43 |  |
| Hhex | 4.19 | 3.88 |  |
| Blk | 4.18 | 2.93 |  |
| Cnr2 | 4.17 | 3.55 |  |
| Egfl7 | 4.15 | 3.95 |  |
| Dennd3 | 4.14 | 3.25 |  |
| C2 | 4.14 | 4.26 |  |
| Adcy4 | 4.13 | 3.37 |  |
| Ccl5 | 4.13 | 3.52 |  |
| Nuak2 | 4.11 | 3.03 |  |
| Mylk | 4.09 | 3.67 |  |
| Sidt1 | 4.08 | 3.2 |  |
| Cldn5 | 4.02 | 4.47 |  |
| LOC100044538 | 3.99 | 2.91 |  |
| Dnase1l3 | 3.91 | 3.73 |  |
| Ccl21b | 3.89 | 3.73 |  |
| Anxa3 | 3.89 | 3.17 |  |
| Lat2 | 3.88 | 2.73 |  |
| Cp | 3.88 | 4.44 |  |
| Ctsh | 3.83 | 3.54 |  |
| Rnase6 | 3.82 | 2.84 |  |
| Klf2 | 3.81 | 3.35 |  |
| Tgm2 | 3.79 | 3.69 |  |
| Unc93b1 | 3.74 | 3.05 |  |
| Slamf9 | 3.73 | 3.15 |  |
| Snx8 | 3.72 | 2.53 |  |
| Nkg7 | 3.71 | 3.27 |  |
| Creld2 | 3.68 | 2.95 |  |
| C3 | 3.68 | 3.36 |  |
| Bgn | 3.67 | 4.09 |  |
| Fgd2 | 3.67 | 4.39 |  |
| Socs3 | 3.66 | 2.83 |  |
| Cyp1b1 | 3.65 | 3.7 |  |
| Mmrn2 | 3.65 | 3.59 |  |
| Ebi2 | 3.64 | 2.9 |  |
| Glipr2 | 3.63 | 2.96 |  |
| Zc3h12d | 3.6 | 2.71 |  |
| Fxyd6 | 3.59 | 3.48 |  |
| Cygb | 3.58 | 3.92 |  |
| Apol7c | 3.57 | 3.41 |  |
| Robo4 | 3.56 | 3.17 |  |
| Myl4 | 3.55 | 3.04 |  |
| Swap70 | 3.53 | 2.97 |  |
| Ldb2 | 3.52 | 2.97 |  |
| Irak3 | 3.51 | 3.02 |  |
| Des | 3.5 | 3.73 |  |
| Samhd1 | 3.49 | 2.42 |  |
| Fcgr2b | 3.49 | 3.13 |  |
| Srgn | 3.48 | 3.17 |  |
| Csf1r | 3.47 | 3.42 |  |
| Socs2 | 3.47 | 2.88 |  |
| Tspan7 | 3.46 | 4.02 |  |
| Lyz | 3.45 | 3.44 |  |
| Ppap2a | 3.43 | 3.68 |  |
| Sox18 | 3.4 | 3.9 |  |
| Gimap7 | 3.39 | 3.35 |  |
| Pdia4 | 3.39 | 3.2 |  |
| Ciita | 3.38 | 2.32 |  |
| Nod1 | 3.34 | 2.66 |  |
| Scotin | 3.33 | 2.55 |  |
| Fscn1 | 3.32 | 2.91 |  |
| P2ry13 | 3.3 | 3.05 |  |
| Ffar2 | 3.29 | 2.7 |  |
| Nr1h3 | 3.27 | 2.36 |  |
| Pglyrp1 | 3.26 | 2.63 |  |
| Pold4 | 3.25 | 2.72 |  |
| Eng | 3.23 | 3.2 |  |
| Golm1 | 3.23 | 2.77 |  |
| H2-Oa | 3.22 | 2.55 |  |
| Ifi30 | 3.2 | 3.19 |  |
| Tmem2 | 3.14 | 3.21 |  |
| LOC100045981 | 3.12 | 2.39 |  |
| Capg | 3.11 | 2.81 |  |
| Cd37 | 3.11 | 2.71 |  |
| Sel1l | 3.11 | 2.65 |  |
| Lyz1 | 3.11 | 3.45 |  |
| Sorl1 | 3.1 | 3.77 |  |
| EG630499 | 3.05 | 2.86 |  |
| Fcna | 3.05 | 3.3 |  |
| Emcn | 3.03 | 3.24 |  |
| Zfp318 | 2.99 | 2 |  |
| Ly6c1 | 2.97 | 3.56 |  |
| LOC547343 | 2.96 | 2.66 |  |
| Stard8 | 2.94 | 2.51 |  |
| Esam1 | 2.94 | 2.7 |  |
| ENSMUSG00000068790 | 2.93 | 1.72 |  |
| B3gnt5 | 2.92 | 3.04 |  |
| Gm2a | 2.91 | 2.5 |  |
| H2-DMb2 | 2.9 | 3.64 |  |
| Cfp | 2.9 | 2.02 |  |
| Lrrk1 | 2.9 | 2.12 |  |
| H2-Q8 | 2.88 | 2.75 |  |
| Gata2 | 2.87 | 2.62 |  |
| Cd40 | 2.85 | 2.41 |  |
| Aldh3b1 | 2.84 | 1.85 |  |
| Timp3 | 2.83 | 2.13 |  |
| Prdx4 | 2.83 | 3.28 |  |
| Casp1 | 2.83 | 2.42 |  |
| Cd53 | 2.82 | 2.19 |  |
| Gadd45b | 2.82 | 1.93 |  |
| Slc11a1 | 2.81 | 2.95 |  |
| Zeb2 | 2.8 | 1.96 |  |
| Xbp1 | 2.8 | 2.48 |  |
| Gbp3 | 2.8 | 2.83 |  |
| Cd52 | 2.8 | 1.88 |  |
| Slc15a3 | 2.79 | 2.68 |  |
| E330036I19Rik | 2.79 | 3.1 |  |
| Irf5 | 2.77 | 1.72 |  |
| Tpst1 | 2.76 | 2.97 |  |
| Tmem86a | 2.74 | 2.52 |  |
| BC004728 | 2.73 | 2.37 |  |
| Rogdi | 2.73 | 2.23 |  |
| Aqp1 | 2.72 | 2.23 |  |
| Nt5e | 2.7 | 1.72 |  |
| Centd3 | 2.7 | 2.08 |  |
| Fes | 2.69 | 2.19 |  |
| Rgl1 | 2.68 | 2.6 |  |
| Cd74 | 2.68 | 2.92 |  |
| BC028528 | 2.68 | 2.98 |  |
| H2-Eb1 | 2.68 | 1.85 |  |
| EG667977 | 2.67 | 2.49 |  |
| Prr13 | 2.66 | 1.72 |  |
| Evi2a | 2.65 | 3.08 |  |
| Irak2 | 2.64 | 2.36 |  |
| Hyal2 | 2.63 | 2.49 |  |
| D3Ucla1 | 2.62 | 2.41 |  |
| Col16a1 | 2.61 | 2.21 |  |
| LOC621823 | 2.61 | 1.95 |  |
| Ctsz | 2.59 | 1.73 |  |
| Sh3tc1 | 2.59 | 2.45 |  |
| Plac8 | 2.59 | 2.13 |  |
| Add3 | 2.58 | 2.48 |  |
| LOC100046883 | 2.57 | 2.14 |  |
| Ppp3ca | 2.56 | 1.88 |  |
| Tmem204 | 2.56 | 3.15 |  |
| Cd63 | 2.56 | 2.24 |  |
| St6galnac2 | 2.55 | 2.84 |  |
| H2-DMa | 2.55 | 2.38 |  |
| Sdc3 | 2.54 | 2.28 |  |
| Rps5 | 2.51 | 2.13 |  |
| Asb2 | 2.5 | 1.65 |  |
| Bin1 | 2.5 | 1.54 |  |
| Ssr4 | 2.49 | 2.31 |  |
| Myadm | 2.47 | 2.47 |  |
| Sepp1 | 2.47 | 2.98 |  |
| Gusb | 2.47 | 1.65 |  |
| Tmem49 | 2.46 | 2.57 |  |
| Uap1l1 | 2.45 | 2.3 |  |
| Litaf | 2.45 | 2.44 |  |
| Atp10d | 2.45 | 2.36 |  |
| Aif1 | 2.45 | 2.54 |  |
| Angptl4 | 2.44 | 3.11 |  |
| App | 2.44 | 2.63 |  |
| Ctsw | 2.44 | 1.62 |  |
| 1200002N14Rik | 2.42 | 2.94 |  |
| Grn | 2.4 | 2.29 |  |
| Sepn1 | 2.39 | 2.05 |  |
| Lhfp | 2.38 | 2.53 |  |
| Flt1 | 2.36 | 2.04 |  |
| Vasn | 2.36 | 2.7 |  |
| Arhgef3 | 2.36 | 1.64 |  |
| Blvrb | 2.34 | 2.11 |  |
| Sema4a | 2.32 | 1.67 |  |
| Ly6a | 2.31 | 2.16 |  |
| Ltb | 2.31 | 1.78 |  |
| Ankrd47 | 2.3 | 1.56 |  |
| Hs3st1 | 2.3 | 2.08 |  |
| X99384 | 2.3 | 2.8 |  |
| Lst1 | 2.29 | 2.21 |  |
| Il10ra | 2.28 | 1.97 |  |
| Ppm1m | 2.28 | 1.86 |  |
| Sas | 2.25 | 1.89 |  |
| P2ry6 | 2.25 | 2.48 |  |
| Cdk2ap2 | 2.24 | 2.45 |  |
| Rpl12 | 2.24 | 1.54 |  |
| C1qa | 2.24 | 2.1 |  |
| Psme1 | 2.23 | 1.85 |  |
| Cotl1 | 2.22 | 1.69 |  |
| Gns | 2.22 | 1.75 |  |
| Tyrobp | 2.22 | 2.5 |  |
| Rrbp1 | 2.21 | 1.89 |  |
| Prrc1 | 2.21 | 2.01 |  |
| Pros1 | 2.21 | 1.85 |  |
| Gbp2 | 2.2 | 1.94 |  |
| Fbxo4 | 2.2 | 1.71 |  |
| Snx2 | 2.19 | 1.53 |  |
| Lcp1 | 2.18 | 2.59 |  |
| Mvp | 2.18 | 1.9 |  |
| H2-Q7 | 2.18 | 2.55 |  |
| Ostf1 | 2.17 | 2.31 |  |
| H2-Ab1 | 2.17 | 2.25 |  |
| Tmem176b | 2.16 | 2.41 |  |
| H47 | 2.16 | 1.64 |  |
| Sgk1 | 2.16 | 1.79 |  |
| Fchsd2 | 2.15 | 1.71 |  |
| Samsn1 | 2.14 | 2.53 |  |
| LOC100046953 | 2.14 | 2.17 |  |
| Vegfa | 2.14 | 2.92 |  |
| Rab31 | 2.12 | 1.69 |  |
| Mmp14 | 2.12 | 1.55 |  |
| Eef1b2 | 2.11 | 2.53 |  |
| Rhoj | 2.11 | 2.15 |  |
| Aadacl1 | 2.1 | 1.61 |  |
| LOC100045343 | 2.1 | 1.91 |  |
| Rab6ip1 | 2.1 | 2.09 |  |
| Lgals3bp | 2.09 | 1.82 |  |
| Fcgrt | 2.08 | 2.52 |  |
| Rassf3 | 2.06 | 2.06 |  |
| Pdgfra | 2.06 | 2.76 |  |
| Zfp608 | 2.06 | 1.7 |  |
| Abca3 | 2.05 | 1.86 |  |
| Tpst2 | 2.05 | 1.83 |  |
| Lgmn | 2.05 | 2.26 |  |
| Obrgrp | 2.04 | 1.63 |  |
| LOC641240 | 2.04 | 1.64 |  |
| Notch4 | 2.04 | 1.56 |  |
| Scara3 | 2.03 | 2.26 |  |
| Pea15 | 2.03 | 1.86 |  |
| Smpdl3a | 2.02 | 2.24 |  |
| LOC100043391 | 2.02 | 1.99 |  |
| Gucy1a3 | 2.01 | 2.63 |  |
| Cyba | 2 | 1.82 |  |
| Pml | 1.99 | 1.57 |  |
| 1700021K19Rik | 1.99 | 1.55 |  |
| Cyp4v3 | 1.99 | 2.13 |  |
| Ctsc | 1.98 | 2.22 |  |
| Evl | 1.98 | 1.58 |  |
| Gstm2 | 1.98 | 2.24 |  |
| Ehd4 | 1.97 | 2.09 |  |
| Arpc1b | 1.95 | 1.55 |  |
| B2m | 1.92 | 2 |  |
| Dab2 | 1.91 | 2.22 |  |
| Bola2 | 1.9 | 1.54 |  |
| Cd93 | 1.9 | 2.28 |  |
| Hspg2 | 1.9 | 1.75 |  |
| Nucb1 | 1.89 | 2.09 |  |
| Plekho2 | 1.88 | 1.87 |  |
| Tmsb10 | 1.87 | 1.77 |  |
| Sec11c | 1.86 | 1.57 |  |
| Plod3 | 1.86 | 1.87 |  |
| Ms4a6d | 1.85 | 2.2 |  |
| Rps3 | 1.84 | 1.96 |  |
| Foxo1 | 1.83 | 1.89 |  |
| Rassf4 | 1.83 | 1.88 |  |
| Rpl39 | 1.82 | 1.61 |  |
| Apoe | 1.82 | 1.68 |  |
| Cebpb | 1.82 | 2.46 |  |
| Arsb | 1.82 | 2.19 |  |
| Sult1a1 | 1.81 | 1.7 |  |
| Grb10 | 1.81 | 2.01 |  |
| Slc46a3 | 1.81 | 2.28 |  |
| Pdlim7 | 1.8 | 1.84 |  |
| Tmem176a | 1.8 | 1.71 |  |
| Dnajb9 | 1.79 | 2.31 |  |
| Trf | 1.79 | 2.98 |  |
| Arsa | 1.78 | 1.54 |  |
| Rnf135 | 1.78 | 1.64 |  |
| H2-T23 | 1.75 | 1.61 |  |
| 1110059E24Rik | 1.74 | 1.57 |  |
| Apobec1 | 1.71 | 2.16 |  |
| Rps15 | 1.7 | 1.66 |  |
| C1qb | 1.68 | 2.18 |  |
| Adssl1 | 1.67 | 2.01 |  |
| Pfdn5 | 1.66 | 1.73 |  |
| Ifi47 | 1.65 | 1.72 |  |
| Arrdc4 | 1.64 | 1.95 |  |
| 4632417K18Rik | 1.62 | 1.82 |  |
| Aatk | 1.61 | 2.1 |  |
| Asns | 1.61 | 2.52 |  |
| Pqlc3 | 1.61 | 1.88 |  |
| Rps7 | 1.6 | 1.72 |  |
| Krtcap2 | 1.6 | 1.66 |  |
| Rabac1 | 1.59 | 1.62 |  |
| Rpl18 | 1.57 | 1.82 |  |
| Ptprb | 1.57 | 2.01 |  |
| Hsd3b7 | 1.56 | 1.52 |  |
| Rpl34 | 1.56 | 1.51 |  |
| Rps9 | 1.53 | 1.51 |  |
| Tdrd7 | 1.52 | 1.59 |  |
| C1qc | 1.52 | 2.04 |  |
| Sspn | 1.52 | 1.89 |  |
| Rhbdf1 | 1.52 | 1.55 |  |
| Tnfrsf21 | 1.51 | 1.76 |  |
| Col6a1 | 1.5 | 1.51 |  |
|  |  |  |  |
| Chfr | -1.5 | -2.15 |  |
| Klf7 | -1.51 | -1.69 |  |
| Slc9a3r1 | -1.52 | -1.88 |  |
| Abtb1 | -1.55 | -2.18 |  |
| 2810046L04Rik | -1.55 | -1.61 |  |
| Ints7 | -1.56 | -1.94 |  |
| Casp2 | -1.56 | -1.62 |  |
| Zfp251 | -1.57 | -2 |  |
| Epc1 | -1.57 | -1.61 |  |
| Mllt11 | -1.59 | -1.68 |  |
| Tbl1x | -1.61 | -1.73 |  |
| Anapc5 | -1.62 | -1.86 |  |
| Usp3 | -1.63 | -1.58 |  |
| AA536749 | -1.64 | -1.77 |  |
| Bcl7a | -1.68 | -1.6 |  |
| Hpgd | -1.69 | -1.5 |  |
| Zap70 | -1.69 | -2.28 |  |
| Pum2 | -1.7 | -1.5 |  |
| Cnot2 | -1.71 | -1.93 |  |
| Slc29a1 | -1.71 | -1.86 |  |
| Metrn | -1.72 | -2.11 |  |
| Aff1 | -1.74 | -2.36 |  |
| Slco3a1 | -1.75 | -1.71 |  |
| Trp53inp1 | -1.75 | -1.71 |  |
| Ncaph2 | -1.77 | -1.94 |  |
| Cd6 | -1.78 | -2.02 |  |
| Pdpk1 | -1.78 | -1.72 |  |
| Cstf2 | -1.79 | -1.52 |  |
| Ddx17 | -1.79 | -2.54 |  |
| Lbr | -1.8 | -2.23 |  |
| Aldh4a1 | -1.8 | -1.58 |  |
| Cdan1 | -1.82 | -2.31 |  |
| Egr1 | -1.82 | -2.39 |  |
| LOC100045040 | -1.85 | -1.64 |  |
| D16Ertd472e | -1.85 | -2.36 |  |
| Hdac2 | -1.86 | -1.89 |  |
| Impdh1 | -1.87 | -1.88 |  |
| Pdk1 | -1.87 | -2.02 |  |
| Ckap5 | -1.87 | -1.78 |  |
| Nasp | -1.89 | -1.85 |  |
| Zfp238 | -1.9 | -1.52 |  |
| Anxa2 | -1.9 | -1.94 |  |
| 5830405N20Rik | -1.93 | -2.33 |  |
| Fas | -1.93 | -1.93 |  |
| Pou6f1 | -1.95 | -2.39 |  |
| Jmjd3 | -1.96 | -1.76 |  |
| Rbmx | -1.96 | -2.11 |  |
| Cdk2 | -1.97 | -1.89 |  |
| Lip1 | -1.98 | -2.65 |  |
| Tfrc | -1.98 | -1.8 |  |
| Eif4e3 | -2.01 | -1.63 |  |
| Uspl1 | -2.01 | -2.44 |  |
| Cstb | -2.01 | -2.04 |  |
| Zdhhc14 | -2.01 | -2.23 |  |
| Phc1 | -2.02 | -2.23 |  |
| Arid2 | -2.03 | -2.73 |  |
| Als2 | -2.04 | -1.96 |  |
| Gadd45a | -2.04 | -1.74 |  |
| Jakmip1 | -2.05 | -2.66 |  |
| Dnmt1 | -2.05 | -2.33 |  |
| Cenpa | -2.06 | -2.28 |  |
| Fyb | -2.06 | -2 |  |
| Hes6 | -2.08 | -1.66 |  |
| Fbxl14 | -2.09 | -1.7 |  |
| Lrig1 | -2.11 | -2.05 |  |
| Tbcel | -2.12 | -2.31 |  |
| Hist1h2af | -2.16 | -2.48 |  |
| Col18a1 | -2.18 | -1.75 |  |
| A130092J06Rik | -2.18 | -2.67 |  |
| Lig1 | -2.22 | -2.43 |  |
| Lman2l | -2.23 | -2.77 |  |
| Bcl6 | -2.26 | -2.42 |  |
| Tbc1d10c | -2.29 | -2.97 |  |
| Khdrbs1 | -2.3 | -1.63 |  |
| Def6 | -2.32 | -2.56 |  |
| Psat1 | -2.35 | -1.7 |  |
| Hist1h2an | -2.35 | -2.69 |  |
| Csnk1e | -2.37 | -2.58 |  |
| Cd3d | -2.38 | -2.48 |  |
| Jmjd1a | -2.38 | -1.92 |  |
| Agtrap | -2.41 | -2.91 |  |
| Sla | -2.45 | -2.1 |  |
| Gse1 | -2.49 | -2.62 |  |
| Rrm1 | -2.49 | -1.64 |  |
| 1190002H23Rik | -2.49 | -1.93 |  |
| Atp1b1 | -2.49 | -2.95 |  |
| Dyrk1b | -2.5 | -2.25 |  |
| Ncapd3 | -2.51 | -2.01 |  |
| 1500031L02Rik | -2.56 | -1.7 |  |
| Tpcn1 | -2.6 | -2.67 |  |
| Cd3e | -2.61 | -2.59 |  |
| Cdc7 | -2.65 | -3.1 |  |
| Tuft1 | -2.65 | -2.85 |  |
| Cdc20 | -2.69 | -2.7 |  |
| Hist1h2ad | -2.7 | -2.56 |  |
| LOC100044475 | -2.76 | -2.9 |  |
| Hdac7 | -2.77 | -3.3 |  |
| 1500011H22Rik | -2.8 | -2.38 |  |
| Ilvbl | -2.83 | -2.8 |  |
| Birc5 | -2.83 | -2.3 |  |
| Rasgrp1 | -2.86 | -3.42 |  |
| 1110049F12Rik | -2.88 | -2.21 |  |
| Cenpl | -2.88 | -2.58 |  |
| 4930572J05Rik | -2.88 | -2.48 |  |
| Phf2 | -2.94 | -3.26 |  |
| D15Wsu75e | -2.97 | -3.52 |  |
| Hist1h2ak | -2.98 | -2.92 |  |
| Cdca3 | -2.99 | -2.77 |  |
| H2afx | -3.02 | -3.13 |  |
| Tapt1 | -3.07 | -3.37 |  |
| Kif22 | -3.08 | -3.62 |  |
| Satb1 | -3.09 | -3.48 |  |
| Lmnb1 | -3.1 | -2.97 |  |
| Cd27 | -3.1 | -3.7 |  |
| Fbxl12 | -3.15 | -3.5 |  |
| Bhlhb9 | -3.16 | -2.64 |  |
| Anp32e | -3.21 | -2.22 |  |
| Hist1h2ah | -3.22 | -2.95 |  |
| Cyp2f2 | -3.22 | -3.7 |  |
| Prc1 | -3.24 | -2.72 |  |
| Itk | -3.25 | -3.07 |  |
| Cd8b1 | -3.29 | -3.61 |  |
| Hnrpll | -3.29 | -2.41 |  |
| Akap12 | -3.3 | -2.58 |  |
| Msh6 | -3.3 | -3.13 |  |
| Aprin | -3.34 | -2.36 |  |
| P2ry5 | -3.38 | -3.07 |  |
| Hey1 | -3.43 | -2.95 |  |
| Thy1 | -3.48 | -3.83 |  |
| LOC100048845 | -3.5 | -3.28 |  |
| Uhrf1 | -3.53 | -3.35 |  |
| Lat | -3.61 | -4.16 |  |
| Cd3g | -3.62 | -2.94 |  |
| Nusap1 | -3.65 | -3.39 |  |
| Ccnd3 | -3.66 | -3.6 |  |
| Lck | -3.67 | -3.51 |  |
| Hist1h2ag | -3.68 | -3.33 |  |
| Pard6g | -3.74 | -4.28 |  |
| Ezh2 | -3.99 | -3.42 |  |
| E2f2 | -4.05 | -3.56 |  |
| Mier1 | -4.07 | -3.71 |  |
| Xrcc6 | -4.1 | -4.57 |  |
| Hrbl | -4.4 | -3.97 |  |
| Ets2 | -4.52 | -4.29 |  |
| Rasl11b | -4.56 | -4.54 |  |
| Ramp1 | -5.12 | -4.94 |  |
| Cdca7 | -5.3 | -5 |  |
| Mns1 | -5.75 | -6.32 |  |
| Wfdc2 | -6.29 | -8.92 |  |
| Cbr2 | -7.74 | -6.85 |  |
|  |  |  |  |
| Gcnt2 | 2.51 |  |  |
| Sell | 2.44 |  |  |
| Adam15 | 2.4 |  |  |
| Il7r | 2.33 |  |  |
| St6gal1 | 2.33 |  |  |
| H2-M3 | 2.3 |  |  |
| AI451557 | 2.29 |  |  |
| Parp14 | 2.27 |  |  |
| Pdgfb | 2.23 |  |  |
| P2ry14 | 2.23 |  |  |
| Pdlim4 | 2.19 |  |  |
| Myd88 | 2.17 |  |  |
| BC006779 | 2.16 |  |  |
| Spcs3 | 2.13 |  |  |
| H2-Aa | 2.12 |  |  |
| Sh3bp2 | 2.12 |  |  |
| Gimap5 | 2.11 |  |  |
| Bst2 | 2.08 |  |  |
| Dpp7 | 2.07 |  |  |
| Il27ra | 2.07 |  |  |
| Adrb2 | 2.06 |  |  |
| Bhlhb2 | 2.04 |  |  |
| Lamp1 | 2.04 |  |  |
| Tmem51 | 2.03 |  |  |
| Alox5ap | 2.01 |  |  |
| Rin3 | 2 |  |  |
| Iigp2 | 2 |  |  |
| D10Ertd610e | 1.99 |  |  |
| D14Ertd668e | 1.98 |  |  |
| Icam1 | 1.97 |  |  |
| Arid3a | 1.96 |  |  |
| Orai1 | 1.95 |  |  |
| Msc | 1.95 |  |  |
| Rps6ka1 | 1.95 |  |  |
| Lgals3 | 1.94 |  |  |
| Myo1f | 1.94 |  |  |
| Stat4 | 1.89 |  |  |
| Asah1 | 1.85 |  |  |
| Nav1 | 1.84 |  |  |
| Mapkapk2 | 1.83 |  |  |
| Cdc42bpb | 1.82 |  |  |
| Trim26 | 1.81 |  |  |
| Rab32 | 1.8 |  |  |
| Ptpn6 | 1.8 |  |  |
| Myd116 | 1.79 |  |  |
| LOC100046056 | 1.78 |  |  |
| Ly96 | 1.77 |  |  |
| 0610007C21Rik | 1.77 |  |  |
| Nfatc1 | 1.76 |  |  |
| Rai14 | 1.76 |  |  |
| Dapk2 | 1.75 |  |  |
| Cd82 | 1.74 |  |  |
| Snn | 1.71 |  |  |
| Rpl24 | 1.7 |  |  |
| Ctsb | 1.69 |  |  |
| Manea | 1.68 |  |  |
| Hexa | 1.66 |  |  |
| D6Wsu176e | 1.66 |  |  |
| Ptpn18 | 1.66 |  |  |
| Rpl35 | 1.66 |  |  |
| Clptm1l | 1.65 |  |  |
| Rpn2 | 1.64 |  |  |
| Renbp | 1.64 |  |  |
| B3gnt8 | 1.63 |  |  |
| Snx30 | 1.63 |  |  |
| Entpd4 | 1.63 |  |  |
| Glt25d1 | 1.61 |  |  |
| Prnp | 1.61 |  |  |
| Lpxn | 1.6 |  |  |
| Stat3 | 1.6 |  |  |
| Elovl1 | 1.6 |  |  |
| Rps16 | 1.58 |  |  |
| LOC100039532 | 1.58 |  |  |
| Itpr3 | 1.58 |  |  |
| Mfng | 1.56 |  |  |
| Gpnmb | 1.56 |  |  |
| Mgrn1 | 1.54 |  |  |
| Myo1g | 1.54 |  |  |
| Pon2 | 1.54 |  |  |
| LOC100047963 | 1.53 |  |  |
| Lypla3 | 1.53 |  |  |
| Nupr1 | 1.52 |  |  |
| Snx18 | 1.52 |  |  |
| Myl6 | 1.52 |  |  |
| Smap2 | 1.51 |  |  |
| Ugcg | 1.51 |  |  |
| Efnb1 | 1.51 |  |  |
|  |  |  |  |
| Dek | -1.51 |  |  |
| LOC100045551 | -1.51 |  |  |
| Mreg | -1.52 |  |  |
| Hnrph1 | -1.53 |  |  |
| Trim28 | -1.53 |  |  |
| Lsm8 | -1.53 |  |  |
| Senp7 | -1.53 |  |  |
| Sap30 | -1.54 |  |  |
| Bbc3 | -1.55 |  |  |
| Ppid | -1.56 |  |  |
| LOC100047856 | -1.57 |  |  |
| Uba2 | -1.58 |  |  |
| Dcakd | -1.59 |  |  |
| Tacstd2 | -1.6 |  |  |
| Dnajb1 | -1.6 |  |  |
| Vezf1 | -1.6 |  |  |
| Fubp3 | -1.6 |  |  |
| Lcmt1 | -1.61 |  |  |
| Sh3kbp1 | -1.61 |  |  |
| Ssna1 | -1.61 |  |  |
| Pfkm | -1.62 |  |  |
| 2010007H12Rik | -1.63 |  |  |
| 3110009E18Rik | -1.64 |  |  |
| 4932441K18Rik | -1.64 |  |  |
| Siva1 | -1.67 |  |  |
| Hmgn2 | -1.67 |  |  |
| Lsm3 | -1.67 |  |  |
| Tmem41a | -1.74 |  |  |
| Usp7 | -1.78 |  |  |
| Med30 | -1.8 |  |  |
| Psip1 | -1.83 |  |  |
| Cdca4 | -1.86 |  |  |
| Ahdc1 | -1.86 |  |  |
| Mcm2 | -1.88 |  |  |
| Casp6 | -1.88 |  |  |
| Tfdp1 | -1.95 |  |  |
| Cdkn2c | -2.02 |  |  |
| LOC100044177 | -2.03 |  |  |
| Gmcl1 | -2.12 |  |  |
| Uck2 | -2.24 |  |  |
| Igfbp5 | -3.19 |  |  |
|  |  |  |  |
| S100a9 |  | 3.79 |  |
| Ccl9 |  | 3.18 |  |
| Col4a2 |  | 2.97 |  |
| Etfb |  | 2.22 |  |
| Cd9 |  | 2.02 |  |
| 2610103J23Rik |  | 1.99 |  |
| Dhrs7 |  | 1.97 |  |
| Rbp1 |  | 1.97 |  |
| Laptm4a |  | 1.96 |  |
| Aifm2 |  | 1.95 |  |
| Emp3 |  | 1.92 |  |
| Sept15 |  | 1.91 |  |
| 9130213B05Rik |  | 1.88 |  |
| Mtdh |  | 1.83 |  |
| Tspan17 |  | 1.83 |  |
| LOC100045567 |  | 1.83 |  |
| Ube2e2 |  | 1.83 |  |
| Mapre2 |  | 1.79 |  |
| Clec4n |  | 1.77 |  |
| Id2 |  | 1.77 |  |
| Tpm4 |  | 1.72 |  |
| Rpl7a |  | 1.72 |  |
| Ndfip1 |  | 1.72 |  |
| Slc25a20 |  | 1.72 |  |
| Atf5 |  | 1.69 |  |
| Acadvl |  | 1.68 |  |
| Cst3 |  | 1.67 |  |
| Fermt2 |  | 1.67 |  |
| Asah3l |  | 1.66 |  |
| Tmed10 |  | 1.65 |  |
| LOC100047998 |  | 1.65 |  |
| Fkbp9 |  | 1.64 |  |
| Snx10 |  | 1.64 |  |
| Cd81 |  | 1.63 |  |
| Rpl36al |  | 1.63 |  |
| Ech1 |  | 1.62 |  |
| Hist1h1c |  | 1.62 |  |
| Mbc2 |  | 1.61 |  |
| LOC381629 |  | 1.61 |  |
| Tmem205 |  | 1.57 |  |
| 2310001A20Rik |  | 1.57 |  |
| Tmem109 |  | 1.56 |  |
| Dad1 |  | 1.55 |  |
| Cnn2 |  | 1.55 |  |
| Tmbim4 |  | 1.52 |  |
| Galnt11 |  | 1.52 |  |
| Srm |  | 1.52 |  |
| Tbc1d20 |  | 1.51 |  |
| Rras |  | 1.51 |  |
| Irf2 |  | 1.5 |  |
|  |  |  |  |
| C230071H18Rik |  | -1.51 |  |
| Sgpl1 |  | -1.51 |  |
| Ankrd10 |  | -1.52 |  |
| Ikzf1 |  | -1.53 |  |
| Fntb |  | -1.53 |  |
| Tlk2 |  | -1.53 |  |
| St5 |  | -1.53 |  |
| 2610020O08Rik |  | -1.53 |  |
| H13 |  | -1.54 |  |
| Hbs1l |  | -1.54 |  |
| Zfp148 |  | -1.54 |  |
| Ints1 |  | -1.55 |  |
| Suv420h1 |  | -1.55 |  |
| 2410127E18Rik |  | -1.56 |  |
| Irf2bp1 |  | -1.57 |  |
| 4930455F23Rik |  | -1.57 |  |
| 5730419I09Rik |  | -1.57 |  |
| Ttc3 |  | -1.58 |  |
| Cxcr4 |  | -1.58 |  |
| Cugbp2 |  | -1.58 |  |
| 5730593F17Rik |  | -1.58 |  |
| A730098D12Rik |  | -1.59 |  |
| Wdr51b |  | -1.59 |  |
| Fcho1 |  | -1.6 |  |
| 1810020D17Rik |  | -1.62 |  |
| Zfp212 |  | -1.62 |  |
| Xlr4a |  | -1.62 |  |
| Hist2h2ac |  | -1.63 |  |
| Dap3 |  | -1.63 |  |
| Rbm38 |  | -1.65 |  |
| 2310016C08Rik |  | -1.66 |  |
| Sbf1 |  | -1.67 |  |
| Tollip |  | -1.67 |  |
| Tra2a |  | -1.68 |  |
| Plekhg2 |  | -1.69 |  |
| Sh2d2a |  | -1.69 |  |
| Rtn3 |  | -1.7 |  |
| Tmem63b |  | -1.71 |  |
| Smox |  | -1.73 |  |
| Traf6 |  | -1.73 |  |
| Llgl1 |  | -1.74 |  |
| LOC100045887 |  | -1.75 |  |
| Rasa1 |  | -1.75 |  |
| Mllt3 |  | -1.8 |  |
| Zfp292 |  | -1.8 |  |
| Med24 |  | -1.84 |  |
| Dmtf1 |  | -1.89 |  |
| Nrf1 |  | -1.9 |  |
| Nipbl |  | -1.9 |  |
| Rcor1 |  | -1.9 |  |
| Yeats2 |  | -1.91 |  |
| Helz |  | -1.91 |  |
| Ctcf |  | -1.92 |  |
| 5930416I19Rik |  | -1.94 |  |
| Rnasen |  | -1.98 |  |
| Gprasp1 |  | -1.98 |  |
| Hdc |  | -2.01 |  |
| LOC100046039 |  | -2.03 |  |
| Smarcd2 |  | -2.06 |  |
| Zfp512 |  | -2.13 |  |
| 4921505C17Rik |  | -2.13 |  |
|  |  |  |  |
| Scd1 |  |  | 7.41 |
| LOC100043671 |  |  | 5.98 |
| Cdo1 |  |  | 5.02 |
| Ces3 |  |  | 4.38 |
| LOC100044190 |  |  | 4.13 |
| Fhl1 |  |  | 3.88 |
| S100a1 |  |  | 3.86 |
| Glo1 |  |  | 3.83 |
| Pdhb |  |  | 3.67 |
| Fh1 |  |  | 3.55 |
| EG433923 |  |  | 3.44 |
| Psmb7 |  |  | 3.32 |
| Ndufa12 |  |  | 3.26 |
| Sdcbp |  |  | 3.22 |
| Caprin1 |  |  | 3.2 |
| Smu1 |  |  | 3.15 |
| Mgst3 |  |  | 3.07 |
| Sept15 |  |  | 3.07 |
| EG434858 |  |  | 3.01 |
| Txnl4a |  |  | 2.94 |
| Eif5a |  |  | 2.92 |
| Scp2 |  |  | 2.9 |
| Nola3 |  |  | 2.89 |
| Hnrpk |  |  | 2.87 |
| Cyc1 |  |  | 2.85 |
| Sfrs5 |  |  | 2.8 |
| Tspan3 |  |  | 2.8 |
| Ndufa8 |  |  | 2.78 |
| Vdac3 |  |  | 2.74 |
| Etfa |  |  | 2.7 |
| Supt4h2 |  |  | 2.69 |
| Pno1 |  |  | 2.67 |
| Pdha1 |  |  | 2.66 |
| Cdr2 |  |  | 2.65 |
| Nola2 |  |  | 2.65 |
| Tram1 |  |  | 2.61 |
| Acly |  |  | 2.59 |
| Gstk1 |  |  | 2.59 |
| Hadhb |  |  | 2.58 |
| Naca |  |  | 2.57 |
| Gpd2 |  |  | 2.53 |
| Sqle |  |  | 2.51 |
| Acss2 |  |  | 2.5 |
| Isca1 |  |  | 2.48 |
| Nudt4 |  |  | 2.46 |
| Psmd7 |  |  | 2.45 |
| Acadm |  |  | 2.44 |
| H3f3a |  |  | 2.42 |
| Bola3 |  |  | 2.42 |
| LOC100047353 |  |  | 2.41 |
| Lamp2 |  |  | 2.41 |
| Hsd17b12 |  |  | 2.4 |
| Mmd |  |  | 2.39 |
| Dcps |  |  | 2.38 |
| Pnrc2 |  |  | 2.37 |
| Mgll |  |  | 2.36 |
| Usp39 |  |  | 2.33 |
| Rnf11 |  |  | 2.32 |
| Ndufa4 |  |  | 2.31 |
| 1810035L17Rik |  |  | 2.29 |
| Bxdc2 |  |  | 2.28 |
| 1110001J03Rik |  |  | 2.28 |
| Aacs |  |  | 2.28 |
| Zfp207 |  |  | 2.28 |
| LOC654426 |  |  | 2.28 |
| Echs1 |  |  | 2.23 |
| Sdhc |  |  | 2.22 |
| Ppa1 |  |  | 2.21 |
| Coro1a |  |  | 2.21 |
| Stx8 |  |  | 2.21 |
| Eif3s10 |  |  | 2.21 |
| Timm8b |  |  | 2.17 |
| LOC100048480 |  |  | 2.17 |
| Prdx3 |  |  | 2.16 |
| Ndufa9 |  |  | 2.16 |
| Txnl4 |  |  | 2.15 |
| Rps27a |  |  | 2.15 |
| Hnrpa1 |  |  | 2.15 |
| LOC100047155 |  |  | 2.14 |
| Nudt5 |  |  | 2.12 |
| Dhx15 |  |  | 2.1 |
| Nap1l1 |  |  | 2.08 |
| Tmem14c |  |  | 2.07 |
| Pmp22 |  |  | 2.07 |
| Eif5 |  |  | 2.06 |
| 1500032D16Rik |  |  | 2.04 |
| Gabarap |  |  | 2.04 |
| Fdps |  |  | 2.04 |
| Pcyox1 |  |  | 2.03 |
| Hsp90ab1 |  |  | 2.03 |
| 2610029G23Rik |  |  | 2.02 |
| 9430029K10Rik |  |  | 2 |
| Smarce1 |  |  | 2 |
| Fundc1 |  |  | 2 |
| Snap23 |  |  | 1.99 |
| Ndufa5 |  |  | 1.99 |
| Ndufs4 |  |  | 1.99 |
| Slc25a5 |  |  | 1.96 |
| Cs |  |  | 1.95 |
| Commd1 |  |  | 1.93 |
| Mdh2 |  |  | 1.92 |
| D10Ertd322e |  |  | 1.92 |
| Capns1 |  |  | 1.9 |
| Hspa9 |  |  | 1.9 |
| Coq5 |  |  | 1.88 |
| Prkaca |  |  | 1.88 |
| Pcbp1 |  |  | 1.85 |
| Mrps33 |  |  | 1.85 |
| Arl6ip5 |  |  | 1.84 |
| 1110008P14Rik |  |  | 1.84 |
| Gpsn2 |  |  | 1.83 |
| 1200003C05Rik |  |  | 1.82 |
| Mthfd1 |  |  | 1.82 |
| 5730437N04Rik |  |  | 1.82 |
| Rnf167 |  |  | 1.81 |
| Ppm1a |  |  | 1.8 |
| Hsd11b1 |  |  | 1.79 |
| 2610204L23Rik |  |  | 1.78 |
| Hmox2 |  |  | 1.78 |
| Lsm2 |  |  | 1.78 |
| Tcp1 |  |  | 1.76 |
| Ndufs2 |  |  | 1.75 |
| LOC100048613 |  |  | 1.74 |
| Mrps18b |  |  | 1.74 |
| Hbp1 |  |  | 1.74 |
| Arpc1a |  |  | 1.73 |
| Vps29 |  |  | 1.73 |
| Tomm7 |  |  | 1.71 |
| Ghitm |  |  | 1.71 |
| Atp5c1 |  |  | 1.71 |
| LOC100046393 |  |  | 1.7 |
| Arpc5 |  |  | 1.69 |
| Tank |  |  | 1.69 |
| Ppp2r1a |  |  | 1.69 |
| LOC100046855 |  |  | 1.69 |
| Gnb1 |  |  | 1.68 |
| EG622339 |  |  | 1.68 |
| l7Rn6 |  |  | 1.68 |
| Tmem50b |  |  | 1.67 |
| Tmem59 |  |  | 1.66 |
| 2010316F05Rik |  |  | 1.66 |
| Lin54 |  |  | 1.65 |
| Mrpl30 |  |  | 1.65 |
| Magoh |  |  | 1.64 |
| Dynll1 |  |  | 1.64 |
| Ube2k |  |  | 1.64 |
| Phlda1 |  |  | 1.63 |
| Lgals1 |  |  | 1.63 |
| LOC668492 |  |  | 1.62 |
| Atp5j |  |  | 1.61 |
| Hsd17b4 |  |  | 1.61 |
| Hprt1 |  |  | 1.6 |
| Ahsa1 |  |  | 1.6 |
| Csde1 |  |  | 1.59 |
| Snrpb |  |  | 1.59 |
| Ormdl3 |  |  | 1.59 |
| Ak3 |  |  | 1.58 |
| Rab35 |  |  | 1.58 |
| Scoc |  |  | 1.58 |
| Cyb5r4 |  |  | 1.57 |
| Ccdc47 |  |  | 1.56 |
| Use1 |  |  | 1.55 |
| Nme3 |  |  | 1.54 |
| LOC100045617 |  |  | 1.54 |
| Cops6 |  |  | 1.54 |
| Rnps1 |  |  | 1.54 |
| Ddx47 |  |  | 1.54 |
| Sqstm1 |  |  | 1.53 |
| Cnot7 |  |  | 1.53 |
| Atf4 |  |  | 1.53 |
| Atp6v0e |  |  | 1.53 |
| Fkbp4 |  |  | 1.52 |
| Tufm |  |  | 1.51 |
| Ebp |  |  | 1.51 |
|  |  |  |  |
| Prf1 |  |  | -1.5 |
| Mecp2 |  |  | -1.51 |
| 5133401N09Rik |  |  | -1.51 |
| Sertad2 |  |  | -1.51 |
| Pigo |  |  | -1.52 |
| Lias |  |  | -1.52 |
| Unc84b |  |  | -1.52 |
| Hdgfrp2 |  |  | -1.52 |
| Atg16l1 |  |  | -1.52 |
| Dctn5 |  |  | -1.53 |
| 1810008A18Rik |  |  | -1.53 |
| Igtp |  |  | -1.53 |
| Luzp1 |  |  | -1.53 |
| Whsc2 |  |  | -1.53 |
| Trp53bp1 |  |  | -1.53 |
| Napg |  |  | -1.53 |
| Ier3 |  |  | -1.53 |
| Usp20 |  |  | -1.54 |
| Atp2a3 |  |  | -1.54 |
| Clk2 |  |  | -1.54 |
| Rcan3 |  |  | -1.54 |
| Gapvd1 |  |  | -1.55 |
| Rnf8 |  |  | -1.55 |
| Klhl6 |  |  | -1.55 |
| Statip1 |  |  | -1.55 |
| Tmem63a |  |  | -1.56 |
| ORF61 |  |  | -1.56 |
| D11Wsu47e |  |  | -1.56 |
| Zc3h18 |  |  | -1.56 |
| Pick1 |  |  | -1.56 |
| Krtcap3 |  |  | -1.56 |
| Nagk |  |  | -1.57 |
| Ubp1 |  |  | -1.57 |
| 3300001P08Rik |  |  | -1.57 |
| Prpf19 |  |  | -1.57 |
| Arhgef1 |  |  | -1.58 |
| Trit1 |  |  | -1.58 |
| Phc2 |  |  | -1.58 |
| LOC100044298 |  |  | -1.58 |
| Atp6v0a2 |  |  | -1.58 |
| 1810027O10Rik |  |  | -1.58 |
| Golga2 |  |  | -1.59 |
| Cant1 |  |  | -1.59 |
| 4930432O21Rik |  |  | -1.59 |
| Hgs |  |  | -1.6 |
| Wsb2 |  |  | -1.6 |
| Cnp |  |  | -1.61 |
| Degs1 |  |  | -1.61 |
| Ep400 |  |  | -1.62 |
| Rbm5 |  |  | -1.62 |
| Ift172 |  |  | -1.62 |
| Daxx |  |  | -1.63 |
| Agrn |  |  | -1.63 |
| Pip4k2b |  |  | -1.63 |
| Ehmt2 |  |  | -1.63 |
| Txlna |  |  | -1.64 |
| Hp1bp3 |  |  | -1.64 |
| BC025076 |  |  | -1.64 |
| Phkg2 |  |  | -1.64 |
| Pacs1 |  |  | -1.66 |
| Eps15l1 |  |  | -1.66 |
| D4Wsu132e |  |  | -1.67 |
| LOC100045882 |  |  | -1.67 |
| Tagap |  |  | -1.67 |
| Tomm22 |  |  | -1.68 |
| 6430527G18Rik |  |  | -1.68 |
| Slc35b3 |  |  | -1.71 |
| Dhx38 |  |  | -1.71 |
| 2400003C14Rik |  |  | -1.72 |
| Preb |  |  | -1.72 |
| Ppox |  |  | -1.72 |
| Ppp4c |  |  | -1.73 |
| Med15 |  |  | -1.73 |
| Zfp263 |  |  | -1.73 |
| Ccdc130 |  |  | -1.74 |
| Kns2 |  |  | -1.75 |
| Skiv2l |  |  | -1.76 |
| Yeats4 |  |  | -1.76 |
| Mycbp2 |  |  | -1.76 |
| Specc1l |  |  | -1.76 |
| Med23 |  |  | -1.76 |
| 2700087H15Rik |  |  | -1.77 |
| Pqlc1 |  |  | -1.77 |
| Pacs2 |  |  | -1.77 |
| Ap3m2 |  |  | -1.77 |
| Vps16 |  |  | -1.77 |
| ENSMUSG00000053178 | |  | -1.78 |
| Cdc2l1 |  |  | -1.78 |
| 2410001C21Rik |  |  | -1.78 |
| Slc25a45 |  |  | -1.78 |
| Arrdc3 |  |  | -1.78 |
| Keap1 |  |  | -1.79 |
| Slc25a28 |  |  | -1.79 |
| Sppl3 |  |  | -1.79 |
| Zkscan6 |  |  | -1.79 |
| Oma1 |  |  | -1.8 |
| Ddx6 |  |  | -1.8 |
| Snx17 |  |  | -1.8 |
| 3110056O03Rik |  |  | -1.8 |
| Clk1 |  |  | -1.81 |
| Slc25a38 |  |  | -1.81 |
| Vat1 |  |  | -1.81 |
| Inmt |  |  | -1.82 |
| Gripap1 |  |  | -1.82 |
| Arhgap9 |  |  | -1.83 |
| 4933439C20Rik |  |  | -1.84 |
| Bat1a |  |  | -1.84 |
| BC067047 |  |  | -1.84 |
| Bcl9l |  |  | -1.85 |
| Aasdh |  |  | -1.87 |
| 6330569M22Rik |  |  | -1.87 |
| Camta2 |  |  | -1.88 |
| Ddx24 |  |  | -1.89 |
| Faah |  |  | -1.9 |
| Tmem68 |  |  | -1.9 |
| Sidt2 |  |  | -1.9 |
| Med12 |  |  | -1.92 |
| Mapk1ip1 |  |  | -1.93 |
| Psmd13 |  |  | -1.95 |
| 1110038F14Rik |  |  | -1.96 |
| Foxj2 |  |  | -1.96 |
| Dhrsx |  |  | -1.96 |
| 2410002F23Rik |  |  | -1.96 |
| Mterf |  |  | -1.96 |
| Acox3 |  |  | -1.97 |
| Il11ra1 |  |  | -1.98 |
| Prpf38b |  |  | -1.98 |
| Ascc2 |  |  | -2 |
| Lrrfip1 |  |  | -2 |
| Chkb |  |  | -2.02 |
| Ihpk1 |  |  | -2.02 |
| Map3k7ip2 |  |  | -2.02 |
| Clk4 |  |  | -2.04 |
| Trib2 |  |  | -2.04 |
| Elp2 |  |  | -2.04 |
| EG665378 |  |  | -2.05 |
| Sgpp1 |  |  | -2.05 |
| Acss1 |  |  | -2.08 |
| 8430432M10Rik |  |  | -2.08 |
| Riok1 |  |  | -2.09 |
| Pkm2 |  |  | -2.12 |
| Zc3h7a |  |  | -2.12 |
| BC037034 |  |  | -2.13 |
| Suv420h2 |  |  | -2.13 |
| 2410025L10Rik |  |  | -2.14 |
| Brd2 |  |  | -2.15 |
| Hsd3b2 |  |  | -2.15 |
| Ifngr2 |  |  | -2.16 |
| 2310021P13Rik |  |  | -2.17 |
| Tnrc6a |  |  | -2.2 |
| Zfp523 |  |  | -2.2 |
| Tsc2 |  |  | -2.2 |
| Gpatch2 |  |  | -2.23 |
| Neu1 |  |  | -2.23 |
| Gdi1 |  |  | -2.25 |
| Iqgap1 |  |  | -2.26 |
| LOC622404 |  |  | -2.27 |
| Ccdc88b |  |  | -2.28 |
| Fcgr4 |  |  | -2.29 |
| Pde1b |  |  | -2.3 |
| LOC100048020 |  |  | -2.31 |
| Nisch |  |  | -2.33 |
| Mta3 |  |  | -2.33 |
| 4930504E06Rik |  |  | -2.37 |
| Fbxw17 |  |  | -2.39 |
| Atpbd1b |  |  | -2.4 |
| Cc2d1b |  |  | -2.4 |
| Psmd4 |  |  | -2.41 |
| Mib2 |  |  | -2.43 |
| Tcf25 |  |  | -2.44 |
| Ddx26b |  |  | -2.45 |
| Prkcbp1 |  |  | -2.45 |
| Mt1 |  |  | -2.45 |
| Txnip |  |  | -2.47 |
| Abca7 |  |  | -2.55 |
| Rell1 |  |  | -2.58 |
| LOC100047214 |  |  | -2.59 |
| Haghl |  |  | -2.63 |
| Myo9b |  |  | -2.63 |
| Rnf40 |  |  | -2.63 |
| Ckb |  |  | -2.67 |
| BC030476 |  |  | -2.69 |
| Arid3b |  |  | -2.69 |
| Araf |  |  | -2.75 |
| Lgals4 |  |  | -2.82 |
| Gnptg |  |  | -2.84 |
| Dennd1c |  |  | -2.86 |
| Nup210 |  |  | -2.89 |
| Dpagt1 |  |  | -2.93 |
| Per2 |  |  | -2.94 |
| Dnajc7 |  |  | -2.96 |
| Tap2 |  |  | -3.02 |
| Psmb1 |  |  | -3.06 |
| Axud1 |  |  | -3.07 |
| Per1 |  |  | -3.19 |
| D17H6S56E-5 |  |  | -3.22 |
| Tef |  |  | -3.27 |
| Ddit4 |  |  | -4.14 |
| Rps3a |  |  | -4.16 |
| Tsc22d3 |  |  | -4.54 |
