# Supplementary material for: Genomic deletion of GIT2 induces a premature age-related thymic dysfunction and systemic immune system disruption
Source: Aging (Albany NY). 2017 Mar 4;9(3):706–30. doi: 10.18632/aging.101185 (PMC5391227; doi:10.18632/aging.101185)
Supplement: Supplementary file 8 [file aging-09-706-s008.docx]

**Table S7. *Textrous!-*based natural language collective processing output from the core of 30 GIT2KO PTL-regulated transcripts**. For each extracted word, associated with the full 30 transcript data collection used as the basic input, the resultant Cosine Similarity score, total Z-score and semantic association probability (Probability) are indicated.

| **Word** | **Cosine Similarity** | **Z-score** | **Probability** |
| --- | --- | --- | --- |
| presenilin | 0.825013517 | 4.020744835 | 2.89758E-05 |
| gamma-secretase | 0.823840135 | 4.015095788 | 2.97229E-05 |
| beta-peptide | 0.821894434 | 4.005728543 | 3.08778E-05 |
| presenilins | 0.819939651 | 3.996317582 | 3.22109E-05 |
| fad | 0.8187734 | 3.990702863 | 3.28976E-05 |
| beta-protein | 0.801328508 | 3.90671744 | 4.67246E-05 |
| paraparesis | 0.778315257 | 3.795924112 | 7.35247E-05 |
| amyloid | 0.76240558 | 3.719329712 | 0.000100007 |
| beta-amyloid | 0.75875842 | 3.70177109 | 0.000106953 |
| aspartyl | 0.75122796 | 3.665516987 | 0.000123187 |
| precursor | 0.721463777 | 3.522222461 | 0.000214152 |
| amyloidogenic | 0.689832533 | 3.369939292 | 0.000375841 |
| early-onset | 0.634458465 | 3.103350389 | 0.000957848 |
| beta-secretase | 0.585086261 | 2.865656422 | 0.002078471 |
| alzheimer's | 0.561652326 | 2.752837783 | 0.002952595 |
| dysarthria | 0.523063584 | 2.567058937 | 0.005129131 |
| angiopathy | 0.517780132 | 2.541622671 | 0.005511009 |
| spastic | 0.507484488 | 2.492056066 | 0.006351302 |
| alzheimer | 0.507068882 | 2.490055202 | 0.006387155 |
| familial | 0.462718936 | 2.276540037 | 0.011393113 |
| gsk | 0.459137781 | 2.259299182 | 0.011941692 |
| knock-in | 0.454428725 | 2.236628242 | 0.01264317 |
| senile | 0.439986048 | 2.167096466 | 0.015117427 |
| impairments | 0.428173325 | 2.110226146 | 0.017429178 |
| hemorrhages | 0.422825929 | 2.084482033 | 0.018580084 |
| sulfide | 0.422488681 | 2.082858407 | 0.018625612 |
| single-photon | 0.417058902 | 2.056717672 | 0.019843113 |
| hydrophilic | 0.408878664 | 2.01733533 | 0.021847757 |
| cleavages | 0.403666709 | 1.99224327 | 0.023185529 |
| late-onset | 0.3915689 | 1.934000455 | 0.026556564 |
| gingiva | 0.38682031 | 1.911139184 | 0.02800229 |
| neurogenesis | 0.383104773 | 1.893251373 | 0.029178935 |
| deposits | 0.367190399 | 1.816634364 | 0.034608557 |
| pharynx | 0.350304259 | 1.735338953 | 0.041370403 |
| deficits | 0.33268503 | 1.650514212 | 0.049369287 |
| onset | 0.33129591 | 1.643826534 | 0.050088101 |
| proteolytic | 0.330421156 | 1.639615181 | 0.050502583 |
| cyclin | 0.321362806 | 1.59600532 | 0.055244397 |
| hsu | 0.319733561 | 1.588161601 | 0.05614317 |
| myoclonus | 0.313235697 | 1.556878755 | 0.059735243 |
| fragments | 0.310667556 | 1.544514881 | 0.061173132 |
| transmembrane | 0.309931068 | 1.540969188 | 0.061658393 |
| proteolysis | 0.309360861 | 1.538224024 | 0.062024307 |
| cleavage | 0.306263703 | 1.523313288 | 0.063879353 |
| emission-computed | 0.304337218 | 1.514038559 | 0.06501292 |
| presymptomatic | 0.303916955 | 1.512015276 | 0.065266931 |
| hyaluronate | 0.299755791 | 1.491982068 | 0.067849573 |
| hyaluronic | 0.299322896 | 1.48989797 | 0.068112118 |
| neuritic | 0.298498657 | 1.485929816 | 0.06863956 |
| brdu | 0.296323622 | 1.475458484 | 0.070106272 |
| hyaluronan | 0.294722797 | 1.467751587 | 0.071052111 |
| plaques | 0.293139674 | 1.460129914 | 0.072145037 |
| age-related | 0.29289106 | 1.458933004 | 0.072282554 |
| csc | 0.292527095 | 1.457180761 | 0.07255819 |
| cyclins | 0.291384036 | 1.4516777 | 0.073250803 |
| hyaluronan-binding | 0.281464348 | 1.403921069 | 0.080159425 |
| transendothelial | 0.275784266 | 1.376575294 | 0.084256124 |
| congo | 0.273881831 | 1.367416351 | 0.085812653 |
| huvec | 0.271602602 | 1.356443395 | 0.087549584 |
| endoglycosidase | 0.266934611 | 1.333970156 | 0.091101928 |
| cyclin-dependent | 0.262263324 | 1.311481054 | 0.094928882 |
| co-immunoprecipitations | 0.259637647 | 1.298840181 | 0.096971965 |
| metastasis | 0.257856895 | 1.290267059 | 0.098525329 |
| proliferative | 0.256663135 | 1.284519908 | 0.099396142 |
| cell | 0.255086605 | 1.276929976 | 0.100801124 |
| microbeads | 0.254991032 | 1.276469854 | 0.10097776 |
| m-phase | 0.254246278 | 1.272884365 | 0.101509021 |
| chondroitinase | 0.254206879 | 1.272694688 | 0.101509021 |
| headache | 0.252651793 | 1.265207991 | 0.102935664 |
| integrin-associated | 0.251891988 | 1.26155004 | 0.103474393 |
| brains | 0.250906335 | 1.256804788 | 0.104376818 |
| cell-surface | 0.250422178 | 1.254473894 | 0.104921003 |
| cytometry | 0.250404407 | 1.254388338 | 0.104921003 |
| generators | 0.24906763 | 1.247952658 | 0.106015529 |
| cutting | 0.248976868 | 1.2475157 | 0.106015529 |
| melanocytic | 0.24862337 | 1.245813842 | 0.106382198 |
| hematopoetic | 0.248439664 | 1.244929423 | 0.106565875 |
| leptomeningeal | 0.248132404 | 1.243450173 | 0.106933917 |
| blocking | 0.247594705 | 1.240861517 | 0.107302874 |
| cdk | 0.24726137 | 1.239256729 | 0.107672749 |
| c-met | 0.245880708 | 1.232609771 | 0.108787882 |
| chondroitin | 0.24583872 | 1.232407629 | 0.108974542 |
| standard | 0.245755486 | 1.232006913 | 0.108974542 |
| endothelium | 0.244672283 | 1.226792019 | 0.109911295 |
| recurrence | 0.244259797 | 1.224806174 | 0.11028761 |
| sulindac | 0.241045399 | 1.209331007 | 0.113331423 |
| circumscribed | 0.240926549 | 1.208758826 | 0.113331423 |
| subiculum | 0.240706703 | 1.207700417 | 0.113523631 |
| glycosaminoglycans | 0.239359956 | 1.201216735 | 0.114875601 |
| node | 0.238427738 | 1.19672873 | 0.115653277 |
| umbilical | 0.238247724 | 1.195862088 | 0.11584828 |
| thymomas | 0.237685628 | 1.193155971 | 0.116434687 |
| tetraspanin | 0.237671834 | 1.193089564 | 0.116434687 |
| sulfonamides | 0.23575657 | 1.183868855 | 0.118206531 |
| oligodendrogliomas | 0.235520502 | 1.182732347 | 0.118404574 |
| colony-forming | 0.235100251 | 1.180709121 | 0.118801362 |
| staging | 0.23424798 | 1.176606007 | 0.119597753 |
| progression | 0.23188598 | 1.165234565 | 0.122009496 |
| cell-like | 0.230344491 | 1.157813333 | 0.123432018 |
| tetraspanins | 0.230247407 | 1.15734594 | 0.12363618 |
