# Supplementary material for: Genomic deletion of GIT2 induces a premature age-related thymic dysfunction and systemic immune system disruption
Source: Aging (Albany NY). 2017 Mar 4;9(3):706–30. doi: 10.18632/aging.101185 (PMC5391227; doi:10.18632/aging.101185)
Supplement: Supplementary file 9 [file aging-09-706-s009.docx]

**Table S8. Canonical signaling pathway analysis of transcripts significantly regulated in GIT2KO thymus compared to WT thymus.** Ingenuity Pathway Analysis (IPA)-based canonical signaling pathway analysis (combining Cellular Signaling and Metabolic Pathway Signaling) was performed using the input transcripts significantly regulated in a differential manner in the GIT2KO thymus compared to the WT thymus at 12 months of age. The enrichment probability (indicated as a negative log_10_ of p-value) and ratio for each significantly-populated pathway is indicated. The numbers (and percentages) of transcripts (downregulated, upregulated, unchanged or non-overlapping) from the input experimental data populating the specific IPA pathways are indicated. For downregulated/upregulated transcripts each value indicates the number of identified experimental transcripts as a percentage of the total number of transcripts comprising that pathway (experimental/total) expressed as a percentage of the total pathway (% in parentheses). All pathways are populated by at least two independent experimental transcripts with a probability of at least <0.05. Signaling pathways are ranked in order of the pathways with the greatest percentage downregulated of constituent transcripts first.

| **Canonical Pathways** | **-log(p-value)** | **Ratio** | **Downregulated** | **No change** | **Upregulated** | **No overlap with dataset** |
| --- | --- | --- | --- | --- | --- | --- |
| GADD45 Signaling | 1.69 | 0.13 | 3/24 (13%) | 0/24 (0%) | 0/24 (0%) | 21/24 (88%) |
| Role of p14/p19ARF in Tumor Suppression | 3.67 | 0.17 | 4/35 (11%) | 0/35 (0%) | 2/35 (6%) | 29/35 (83%) |
| Interferon Signaling | 1.75 | 0.11 | 4/36 (11%) | 0/36 (0%) | 0/36 (0%) | 32/36 (89%) |
| Role of JAK family kinases in IL-6-type Cytokine Signaling | 1.43 | 0.11 | 3/28 (11%) | 0/28 (0%) | 0/28 (0%) | 25/28 (89%) |
| T Helper Cell Differentiation | 3.01 | 0.11 | 7/72 (10%) | 0/72 (0%) | 1/72 (1%) | 64/72 (89%) |
| Antigen Presentation Pathway | 2.35 | 0.12 | 4/42 (10%) | 0/42 (0%) | 1/42 (2%) | 37/42 (88%) |
| Melanoma Signaling | 3.67 | 0.14 | 4/50 (8%) | 0/50 (0%) | 3/50 (6%) | 43/50 (86%) |
| Lymphotoxin β Receptor Signaling | 2.29 | 0.10 | 5/62 (8%) | 0/62 (0%) | 1/62 (2%) | 56/62 (90%) |
| EGF Signaling | 2.21 | 0.09 | 5/64 (8%) | 0/64 (0%) | 1/64 (2%) | 58/64 (91%) |
| IL-9 Signaling | 1.75 | 0.10 | 3/40 (8%) | 0/40 (0%) | 1/40 (3%) | 36/40 (90%) |
| Nucleotide Excision Repair Pathway | 1.71 | 0.11 | 3/36 (8%) | 0/36 (0%) | 1/36 (3%) | 32/36 (89%) |
| iNOS Signaling | 1.39 | 0.08 | 4/53 (8%) | 0/53 (0%) | 0/53 (0%) | 49/53 (92%) |
| HER-2 Signaling in Breast Cancer | 2.73 | 0.10 | 6/82 (7%) | 0/82 (0%) | 2/82 (2%) | 74/82 (90%) |
| CNTF Signaling | 2.37 | 0.11 | 4/57 (7%) | 0/57 (0%) | 2/57 (4%) | 51/57 (89%) |
| Prostate Cancer Signaling | 5.23 | 0.12 | 6/103 (6%) | 0/103 (0%) | 6/103 (6%) | 91/103 (88%) |
| Pancreatic Adenocarcinoma Signaling | 4.10 | 0.09 | 8/128 (6%) | 0/128 (0%) | 4/128 (3%) | 116/128 (91%) |
| Non-Small Cell Lung Cancer Signaling | 3.18 | 0.10 | 5/83 (6%) | 0/83 (0%) | 3/83 (4%) | 75/83 (90%) |
| Small Cell Lung Cancer Signaling | 2.93 | 0.09 | 6/94 (6%) | 0/94 (0%) | 2/94 (2%) | 86/94 (91%) |
| Remodeling of Epithelial Adherens Junctions | 2.40 | 0.10 | 4/70 (6%) | 0/70 (0%) | 3/70 (4%) | 63/70 (90%) |
| CD40 Signaling | 1.96 | 0.08 | 4/71 (6%) | 0/71 (0%) | 2/71 (3%) | 65/71 (92%) |
| GM-CSF Signaling | 1.96 | 0.09 | 4/68 (6%) | 0/68 (0%) | 2/68 (3%) | 62/68 (91%) |
| iCOS-iCOSL Signaling in T Helper Cells | 1.84 | 0.06 | 7/126 (6%) | 0/126 (0%) | 1/126 (1%) | 118/126 (94%) |
| JAK/Stat Signaling | 1.84 | 0.08 | 4/71 (6%) | 0/71 (0%) | 2/71 (3%) | 65/71 (92%) |
| Type I Diabetes Mellitus Signaling | 1.82 | 0.07 | 7/121 (6%) | 0/121 (0%) | 1/121 (1%) | 113/121 (93%) |
| ATM Signaling | 1.49 | 0.08 | 4/66 (6%) | 0/66 (0%) | 1/66 (2%) | 61/66 (92%) |
| MSP-RON Signaling Pathway | 1.36 | 0.08 | 3/51 (6%) | 0/51 (0%) | 1/51 (2%) | 47/51 (92%) |
| Cell Cycle: G1/S Checkpoint Regulation | 1.34 | 0.07 | 4/72 (6%) | 0/72 (0%) | 1/72 (1%) | 67/72 (93%) |
| Graft-versus-Host Disease Signaling | 1.33 | 0.08 | 3/51 (6%) | 0/51 (0%) | 1/51 (2%) | 47/51 (92%) |
| TNFR2 Signaling | 1.31 | 0.09 | 2/34 (6%) | 0/34 (0%) | 1/34 (3%) | 31/34 (91%) |
| NRF2-mediated Oxidative Stress Response | 7.69 | 0.11 | 10/195 (5%) | 0/195 (0%) | 12/195 (6%) | 173/195 (89%) |
| Nitric Oxide Signaling in the Cardiovascular System | 3.08 | 0.08 | 6/125 (5%) | 0/125 (0%) | 4/125 (3%) | 115/125 (92%) |
| FLT3 Signaling in Hematopoietic Progenitor Cells | 2.77 | 0.10 | 4/79 (5%) | 0/79 (0%) | 4/79 (5%) | 71/79 (90%) |
| Role of NFAT in Regulation of the Immune Response | 2.74 | 0.07 | 9/200 (5%) | 0/200 (0%) | 4/200 (2%) | 187/200 (94%) |
| Chronic Myeloid Leukemia Signaling | 2.72 | 0.08 | 5/106 (5%) | 0/106 (0%) | 4/106 (4%) | 97/106 (92%) |
| CD28 Signaling in T Helper Cells | 2.62 | 0.07 | 7/136 (5%) | 0/136 (0%) | 3/136 (2%) | 126/136 (93%) |
| UVB-Induced MAPK Signaling | 2.33 | 0.10 | 3/58 (5%) | 0/58 (0%) | 3/58 (5%) | 52/58 (90%) |
| Renin-Angiotensin Signaling | 2.29 | 0.07 | 6/126 (5%) | 0/126 (0%) | 3/126 (2%) | 117/126 (93%) |
| IL-4 Signaling | 2.20 | 0.09 | 4/80 (5%) | 0/80 (0%) | 3/80 (4%) | 73/80 (91%) |
| Fcγ Receptor-mediated Phagocytosis in Macrophages and Monocytes | 2.19 | 0.08 | 5/106 (5%) | 0/106 (0%) | 3/106 (3%) | 98/106 (92%) |
| SAPK/JNK Signaling | 2.14 | 0.08 | 5/105 (5%) | 0/105 (0%) | 3/105 (3%) | 97/105 (92%) |
| Acute Myeloid Leukemia Signaling | 2.10 | 0.08 | 4/84 (5%) | 0/84 (0%) | 3/84 (4%) | 77/84 (92%) |
| p53 Signaling | 2.06 | 0.07 | 6/113 (5%) | 0/113 (0%) | 2/113 (2%) | 105/113 (93%) |
| GDNF Family Ligand-Receptor Interactions | 1.81 | 0.08 | 4/76 (5%) | 0/76 (0%) | 2/76 (3%) | 70/76 (92%) |
| Endometrial Cancer Signaling | 1.73 | 0.08 | 3/60 (5%) | 0/60 (0%) | 2/60 (3%) | 55/60 (92%) |
| HMGB1 Signaling | 1.68 | 0.06 | 5/109 (5%) | 0/109 (0%) | 2/109 (2%) | 102/109 (94%) |
| Prolactin Signaling | 1.67 | 0.07 | 4/84 (5%) | 0/84 (0%) | 2/84 (2%) | 78/84 (93%) |
| Thrombopoietin Signaling | 1.63 | 0.08 | 3/64 (5%) | 0/64 (0%) | 2/64 (3%) | 59/64 (92%) |
| ErbB2-ErbB3 Signaling | 1.57 | 0.08 | 3/63 (5%) | 0/63 (0%) | 2/63 (3%) | 58/63 (92%) |
| Glioma Invasiveness Signaling | 1.54 | 0.08 | 3/66 (5%) | 0/66 (0%) | 2/66 (3%) | 61/66 (92%) |
| Autoimmune Thyroid Disease Signaling | 1.30 | 0.06 | 3/62 (5%) | 0/62 (0%) | 1/62 (2%) | 58/62 (94%) |
| PI3K/AKT Signaling | 4.12 | 0.09 | 6/152 (4%) | 0/152 (0%) | 7/152 (5%) | 139/152 (91%) |
| Protein Ubiquitination Pathway | 3.63 | 0.07 | 11/270 (4%) | 0/270 (0%) | 8/270 (3%) | 251/270 (93%) |
| PEDF Signaling | 3.61 | 0.11 | 3/79 (4%) | 0/79 (0%) | 6/79 (8%) | 70/79 (89%) |
| Estrogen-Dependent Breast Cancer Signaling | 3.28 | 0.11 | 3/73 (4%) | 0/73 (0%) | 5/73 (7%) | 65/73 (89%) |
| RhoGDI Signaling | 2.67 | 0.06 | 8/202 (4%) | 0/202 (0%) | 5/202 (2%) | 189/202 (94%) |
| Gap Junction Signaling | 2.61 | 0.07 | 7/181 (4%) | 0/181 (0%) | 5/181 (3%) | 169/181 (93%) |
| 14-3-3-mediated Signaling | 2.59 | 0.08 | 5/121 (4%) | 0/121 (0%) | 5/121 (4%) | 111/121 (92%) |
| Production of Nitric Oxide and Reactive Oxygen Species in Macrophages | 2.56 | 0.06 | 8/212 (4%) | 0/212 (0%) | 5/212 (2%) | 199/212 (94%) |
| Rac Signaling | 2.42 | 0.07 | 5/128 (4%) | 0/128 (0%) | 4/128 (3%) | 119/128 (93%) |
| Virus Entry via Endocytic Pathways | 2.31 | 0.08 | 4/101 (4%) | 0/101 (0%) | 4/101 (4%) | 93/101 (92%) |
| Docosahexaenoic Acid (DHA) Signaling | 2.25 | 0.10 | 2/50 (4%) | 0/50 (0%) | 3/50 (6%) | 45/50 (90%) |
| Aldosterone Signaling in Epithelial Cells | 2.22 | 0.07 | 6/169 (4%) | 0/169 (0%) | 5/169 (3%) | 158/169 (93%) |
| Molecular Mechanisms of Cancer | 2.16 | 0.05 | 14/388 (4%) | 0/388 (0%) | 5/388 (1%) | 369/388 (95%) |
| Dendritic Cell Maturation | 2.15 | 0.06 | 9/211 (4%) | 0/211 (0%) | 3/211 (1%) | 199/211 (94%) |
| Glioma Signaling | 2.14 | 0.07 | 5/113 (4%) | 0/113 (0%) | 3/113 (3%) | 105/113 (93%) |
| RhoA Signaling | 2.06 | 0.07 | 5/123 (4%) | 0/123 (0%) | 4/123 (3%) | 114/123 (93%) |
| Natural Killer Cell Signaling | 1.77 | 0.07 | 5/118 (4%) | 0/118 (0%) | 3/118 (3%) | 110/118 (93%) |
| Germ Cell-Sertoli Cell Junction Signaling | 1.74 | 0.06 | 6/169 (4%) | 0/169 (0%) | 4/169 (2%) | 159/169 (94%) |
| IL-12 Signaling and Production in Macrophages | 1.73 | 0.06 | 6/157 (4%) | 0/157 (0%) | 3/157 (2%) | 148/157 (94%) |
| PKCθ Signaling in T Lymphocytes | 1.65 | 0.06 | 6/144 (4%) | 0/144 (0%) | 2/144 (1%) | 136/144 (94%) |
| Paxillin Signaling | 1.51 | 0.06 | 5/117 (4%) | 0/117 (0%) | 2/117 (2%) | 110/117 (94%) |
| Glioblastoma Multiforme Signaling | 1.50 | 0.05 | 6/168 (4%) | 0/168 (0%) | 3/168 (2%) | 159/168 (95%) |
| ErbB4 Signaling | 1.49 | 0.07 | 3/69 (4%) | 0/69 (0%) | 2/69 (3%) | 64/69 (93%) |
| Endothelin-1 Signaling | 1.48 | 0.05 | 7/192 (4%) | 0/192 (0%) | 3/192 (2%) | 182/192 (95%) |
| IL-15 Signaling | 1.46 | 0.07 | 3/72 (4%) | 0/72 (0%) | 2/72 (3%) | 67/72 (93%) |
| Leukocyte Extravasation Signaling | 1.46 | 0.05 | 9/210 (4%) | 0/210 (0%) | 2/210 (1%) | 199/210 (95%) |
| Estrogen Receptor Signaling | 1.45 | 0.06 | 6/136 (4%) | 0/136 (0%) | 2/136 (1%) | 128/136 (94%) |
| Role of JAK1 and JAK3 in γc Cytokine Signaling | 1.43 | 0.07 | 3/68 (4%) | 0/68 (0%) | 2/68 (3%) | 63/68 (93%) |
| Angiopoietin Signaling | 1.36 | 0.07 | 3/75 (4%) | 0/75 (0%) | 2/75 (3%) | 70/75 (93%) |
| AMPK Signaling | 3.71 | 0.07 | 5/181 (3%) | 0/181 (0%) | 8/181 (4%) | 168/181 (93%) |
| Glucocorticoid Receptor Signaling | 3.07 | 0.06 | 10/299 (3%) | 0/299 (0%) | 8/299 (3%) | 281/299 (94%) |
| Aryl Hydrocarbon Receptor Signaling | 3.01 | 0.07 | 5/171 (3%) | 0/171 (0%) | 7/171 (4%) | 159/171 (93%) |
| fMLP Signaling in Neutrophils | 2.82 | 0.08 | 4/132 (3%) | 0/132 (0%) | 6/132 (5%) | 122/132 (92%) |
| eNOS Signaling | 2.74 | 0.07 | 5/155 (3%) | 0/155 (0%) | 6/155 (4%) | 144/155 (93%) |
| Breast Cancer Regulation by Stathmin1 | 2.72 | 0.07 | 6/214 (3%) | 0/214 (0%) | 8/214 (4%) | 200/214 (93%) |
| Insulin Receptor Signaling | 2.71 | 0.07 | 4/149 (3%) | 0/149 (0%) | 7/149 (5%) | 138/149 (93%) |
| Systemic Lupus Erythematosus Signaling | 2.55 | 0.06 | 7/256 (3%) | 0/256 (0%) | 8/256 (3%) | 241/256 (94%) |
| Role of NFAT in Cardiac Hypertrophy | 2.50 | 0.06 | 7/209 (3%) | 0/209 (0%) | 6/209 (3%) | 196/209 (94%) |
| α-Adrenergic Signaling | 2.34 | 0.07 | 3/109 (3%) | 0/109 (0%) | 5/109 (5%) | 101/109 (93%) |
| Ovarian Cancer Signaling | 2.20 | 0.07 | 5/152 (3%) | 0/152 (0%) | 5/152 (3%) | 142/152 (93%) |
| PDGF Signaling | 2.10 | 0.08 | 3/86 (3%) | 0/86 (0%) | 4/86 (5%) | 79/86 (92%) |
| IGF-1 Signaling | 2.08 | 0.07 | 3/107 (3%) | 0/107 (0%) | 5/107 (5%) | 99/107 (93%) |
| Clathrin-mediated Endocytosis Signaling | 2.03 | 0.06 | 6/198 (3%) | 0/198 (0%) | 6/198 (3%) | 186/198 (94%) |
| IL-8 Signaling | 1.99 | 0.05 | 7/225 (3%) | 0/225 (0%) | 5/225 (2%) | 213/225 (95%) |
| Telomerase Signaling | 1.98 | 0.08 | 3/106 (3%) | 0/106 (0%) | 5/106 (5%) | 98/106 (92%) |
| Antiproliferative Role of Somatostatin Receptor 2 | 1.93 | 0.08 | 2/72 (3%) | 0/72 (0%) | 4/72 (6%) | 66/72 (92%) |
| Actin Cytoskeleton Signaling | 1.93 | 0.05 | 7/242 (3%) | 0/242 (0%) | 6/242 (2%) | 229/242 (95%) |
| mTOR Signaling | 1.92 | 0.06 | 7/213 (3%) | 0/213 (0%) | 5/213 (2%) | 201/213 (94%) |
| Epithelial Adherens Junction Signaling | 1.92 | 0.06 | 5/154 (3%) | 0/154 (0%) | 5/154 (3%) | 144/154 (94%) |
| Melanocyte Development and Pigmentation Signaling | 1.85 | 0.07 | 3/95 (3%) | 0/95 (0%) | 4/95 (4%) | 88/95 (93%) |
| Cell Cycle Regulation by BTG Family Proteins | 1.71 | 0.10 | 1/39 (3%) | 0/39 (0%) | 3/39 (8%) | 35/39 (90%) |
| CCR3 Signaling in Eosinophils | 1.67 | 0.06 | 4/134 (3%) | 0/134 (0%) | 4/134 (3%) | 126/134 (94%) |
| Myc Mediated Apoptosis Signaling | 1.54 | 0.08 | 2/63 (3%) | 0/63 (0%) | 3/63 (5%) | 58/63 (92%) |
| FcγRIIB Signaling in B Lymphocytes | 1.48 | 0.06 | 2/64 (3%) | 0/64 (0%) | 2/64 (3%) | 60/64 (94%) |
| PI3K Signaling in B Lymphocytes | 1.43 | 0.06 | 5/143 (3%) | 0/143 (0%) | 3/143 (2%) | 135/143 (94%) |
| TR/RXR Activation | 1.39 | 0.06 | 3/109 (3%) | 0/109 (0%) | 3/109 (3%) | 103/109 (94%) |
| Fc Epsilon RI Signaling | 1.36 | 0.06 | 4/117 (3%) | 0/117 (0%) | 3/117 (3%) | 110/117 (94%) |
| FAK Signaling | 1.35 | 0.06 | 3/106 (3%) | 0/106 (0%) | 3/106 (3%) | 100/106 (94%) |
| Hypoxia Signaling in the Cardiovascular System | 1.34 | 0.07 | 2/68 (3%) | 0/68 (0%) | 3/68 (4%) | 63/68 (93%) |
| Neuregulin Signaling | 1.33 | 0.06 | 3/104 (3%) | 0/104 (0%) | 3/104 (3%) | 98/104 (94%) |
| CTLA4 Signaling in Cytotoxic T Lymphocytes | 1.33 | 0.06 | 3/96 (3%) | 0/96 (0%) | 3/96 (3%) | 90/96 (94%) |
| B Cell Receptor Signaling | 1.32 | 0.05 | 5/175 (3%) | 0/175 (0%) | 4/175 (2%) | 166/175 (95%) |
| Xenobiotic Metabolism Signaling | 2.50 | 0.06 | 7/288 (2%) | 0/288 (0%) | 10/288 (3%) | 271/288 (94%) |
| ERK/MAPK Signaling | 2.38 | 0.06 | 4/211 (2%) | 0/211 (0%) | 9/211 (4%) | 198/211 (94%) |
| Huntington's Disease Signaling | 2.16 | 0.06 | 5/252 (2%) | 0/252 (0%) | 9/252 (4%) | 238/252 (94%) |
| P2Y Purigenic Receptor Signaling Pathway | 1.97 | 0.06 | 3/144 (2%) | 0/144 (0%) | 6/144 (4%) | 135/144 (94%) |
| Protein Kinase A Signaling | 1.69 | 0.05 | 10/409 (2%) | 0/409 (0%) | 9/409 (2%) | 390/409 (95%) |
| Signaling by Rho Family GTPases | 1.68 | 0.05 | 6/263 (2%) | 0/263 (0%) | 7/263 (3%) | 250/263 (95%) |
| Acute Phase Response Signaling | 1.53 | 0.06 | 4/181 (2%) | 0/181 (0%) | 6/181 (3%) | 171/181 (94%) |
| Integrin Signaling | 1.47 | 0.05 | 4/208 (2%) | 0/208 (0%) | 7/208 (3%) | 197/208 (95%) |
| CREB Signaling in Neurons | 1.44 | 0.05 | 4/207 (2%) | 0/207 (0%) | 6/207 (3%) | 197/207 (95%) |
| PPARα/RXRα Activation | 1.41 | 0.05 | 4/200 (2%) | 0/200 (0%) | 6/200 (3%) | 190/200 (95%) |
| Cardiac β-adrenergic Signaling | 1.32 | 0.05 | 3/158 (2%) | 0/158 (0%) | 5/158 (3%) | 150/158 (95%) |
| Mitochondrial Dysfunction | 11.40 | 0.12 | 1/215 (0%) | 0/215 (0%) | 25/215 (12%) | 189/215 (88%) |
| G Beta Gamma Signaling | 1.31 | 0.05 | 0/121 (0%) | 0/121 (0%) | 6/121 (5%) | 115/121 (95%) |
| Cell Cycle Control of Chromosomal Replication | 1.31 | 0.09 | 0/34 (0%) | 0/34 (0%) | 3/34 (9%) | 31/34 (91%) |
