# Supplementary material for: Genomic deletion of GIT2 induces a premature age-related thymic dysfunction and systemic immune system disruption
Source: Aging (Albany NY). 2017 Mar 4;9(3):706–30. doi: 10.18632/aging.101185 (PMC5391227; doi:10.18632/aging.101185)
Supplement: Supplementary file 10 [file aging-09-706-s010.docx]

**Table S9. Canonical signaling pathway analysis of transcripts significantly regulated in GIT2KO PTLs compared to GIT2KO thymus.** Canonical signaling pathway analysis (IPA: combining Cellular Signaling and Metabolic Pathway Signaling) was performed using the input transcripts significantly regulated in a differential manner in the GIT2KO thymus compared to the WT thymus at 12 months of age. The enrichment probability (indicated as a negative log_10_ of p-value) and ratio for each significantly-populated pathway is indicated. The numbers (and percentages) of transcripts (downregulated, upregulated, unchanged or non-overlapping) from the input experimental data populating the specific IPA pathways are indicated. For downregulated/upregulated transcripts each value indicates the number of identified experimental transcripts as a percentage of the total number of transcripts comprising that pathway (experimental/total) expressed as a percentage of the total pathway (% in parentheses). All pathways are populated by at least two independent experimental transcripts with a probability of at least <0.05. Signaling pathways are ranked in order of the pathways with the greatest percentage upregulated of constituent transcripts first.

| **Canonical Pathways** | **-log(p-value)** | **Ratio** | **Downregulated** | **No change** | **Upregulated** | **No overlap with dataset** |
| --- | --- | --- | --- | --- | --- | --- |
| Antigen Presentation Pathway | 12.20 | 0.36 | 0/42 (0%) | 0/42 (0%) | 15/42 (36%) | 27/42 (64%) |
| B Cell Development | 8.18 | 0.31 | 0/36 (0%) | 0/36 (0%) | 11/36 (31%) | 25/36 (69%) |
| T Helper Cell Differentiation | 11.70 | 0.26 | 1/72 (1%) | 0/72 (0%) | 18/72 (25%) | 53/72 (74%) |
| Graft-versus-Host Disease Signaling | 8.37 | 0.26 | 1/51 (2%) | 0/51 (0%) | 12/51 (24%) | 38/51 (75%) |
| Complement System | 3.89 | 0.20 | 0/35 (0%) | 0/35 (0%) | 7/35 (20%) | 28/35 (80%) |
| Pathogenesis of Multiple Sclerosis | 1.42 | 0.20 | 0/10 (0%) | 0/10 (0%) | 2/10 (20%) | 8/10 (80%) |
| Autoimmune Thyroid Disease Signaling | 8.25 | 0.21 | 1/62 (2%) | 0/62 (0%) | 12/62 (19%) | 49/62 (79%) |
| Role of JAK2 in Hormone-like Cytokine Signaling | 3.80 | 0.19 | 0/37 (0%) | 0/37 (0%) | 7/37 (19%) | 30/37 (81%) |
| Role of JAK family kinases in IL-6-type Cytokine Signaling | 2.79 | 0.18 | 0/28 (0%) | 0/28 (0%) | 5/28 (18%) | 23/28 (82%) |
| Altered T Cell and B Cell Signaling in Rheumatoid Arthritis | 9.01 | 0.18 | 1/100 (1%) | 0/100 (0%) | 17/100 (17%) | 82/100 (82%) |
| GADD45 Signaling | 5.45 | 0.29 | 3/24 (13%) | 0/24 (0%) | 4/24 (17%) | 17/24 (71%) |
| Nur77 Signaling in T Lymphocytes | 8.32 | 0.22 | 4/64 (6%) | 0/64 (0%) | 10/64 (16%) | 50/64 (78%) |
| IL-22 Signaling | 2.03 | 0.16 | 0/25 (0%) | 0/25 (0%) | 4/25 (16%) | 21/25 (84%) |
| Calcium-induced T Lymphocyte Apoptosis | 10.50 | 0.24 | 7/71 (10%) | 0/71 (0%) | 10/71 (14%) | 54/71 (76%) |
| Type I Diabetes Mellitus Signaling | 9.77 | 0.17 | 4/121 (3%) | 0/121 (0%) | 17/121 (14%) | 100/121 (83%) |
| Cytotoxic T Lymphocyte-mediated Apoptosis of Target Cells | 9.46 | 0.21 | 6/88 (7%) | 0/88 (0%) | 12/88 (14%) | 70/88 (80%) |
| Allograft Rejection Signaling | 7.54 | 0.17 | 2/97 (2%) | 0/97 (0%) | 14/97 (14%) | 81/97 (84%) |
| OX40 Signaling Pathway | 8.14 | 0.18 | 4/97 (4%) | 0/97 (0%) | 13/97 (13%) | 80/97 (82%) |
| TREM1 Signaling | 4.42 | 0.13 | 0/75 (0%) | 0/75 (0%) | 10/75 (13%) | 65/75 (87%) |
| IL-4 Signaling | 4.25 | 0.14 | 1/80 (1%) | 0/80 (0%) | 10/80 (13%) | 69/80 (86%) |
| iNOS Signaling | 3.08 | 0.13 | 0/53 (0%) | 0/53 (0%) | 7/53 (13%) | 46/53 (87%) |
| IL-9 Signaling | 2.19 | 0.13 | 0/40 (0%) | 0/40 (0%) | 5/40 (13%) | 35/40 (88%) |
| Dendritic Cell Maturation | 9.76 | 0.13 | 1/211 (0%) | 0/211 (0%) | 26/211 (12%) | 184/211 (87%) |
| Hepatic Fibrosis / Hepatic Stellate Cell Activation | 7.00 | 0.13 | 2/155 (1%) | 0/155 (0%) | 18/155 (12%) | 135/155 (87%) |
| Inhibition of Angiogenesis by TSP1 | 2.19 | 0.12 | 0/42 (0%) | 0/42 (0%) | 5/42 (12%) | 37/42 (88%) |
| Primary Immunodeficiency Signaling | 5.79 | 0.17 | 4/64 (6%) | 0/64 (0%) | 7/64 (11%) | 53/64 (83%) |
| Communication between Innate and Adaptive Immune Cells | 4.73 | 0.12 | 1/112 (1%) | 0/112 (0%) | 12/112 (11%) | 99/112 (88%) |
| JAK/Stat Signaling | 2.61 | 0.11 | 0/71 (0%) | 0/71 (0%) | 8/71 (11%) | 63/71 (89%) |
| B Cell Activating Factor Signaling | 2.58 | 0.13 | 1/46 (2%) | 0/46 (0%) | 5/46 (11%) | 40/46 (87%) |
| Oncostatin M Signaling | 1.51 | 0.11 | 0/35 (0%) | 0/35 (0%) | 4/35 (11%) | 31/35 (89%) |
| CD28 Signaling in T Helper Cells | 11.70 | 0.18 | 10/136 (7%) | 0/136 (0%) | 14/136 (10%) | 112/136 (82%) |
| Role of NFAT in Regulation of the Immune Response | 11.70 | 0.15 | 10/200 (5%) | 0/200 (0%) | 19/200 (10%) | 171/200 (86%) |
| iCOS-iCOSL Signaling in T Helper Cells | 11.60 | 0.18 | 10/126 (8%) | 0/126 (0%) | 13/126 (10%) | 103/126 (82%) |
| Acute Phase Response Signaling | 5.05 | 0.11 | 1/181 (1%) | 0/181 (0%) | 18/181 (10%) | 162/181 (90%) |
| Growth Hormone Signaling | 3.15 | 0.12 | 1/78 (1%) | 0/78 (0%) | 8/78 (10%) | 69/78 (88%) |
| Prolactin Signaling | 2.97 | 0.11 | 1/84 (1%) | 0/84 (0%) | 8/84 (10%) | 75/84 (89%) |
| STAT3 Pathway | 2.38 | 0.10 | 0/80 (0%) | 0/80 (0%) | 8/80 (10%) | 72/80 (90%) |
| CD40 Signaling | 2.17 | 0.10 | 0/71 (0%) | 0/71 (0%) | 7/71 (10%) | 64/71 (90%) |
| Cdc42 Signaling | 7.90 | 0.12 | 6/186 (3%) | 0/186 (0%) | 17/186 (9%) | 163/186 (88%) |
| Crosstalk between Dendritic Cells and Natural Killer Cells | 3.48 | 0.10 | 1/106 (1%) | 0/106 (0%) | 10/106 (9%) | 95/106 (90%) |
| Role of Pattern Recognition Receptors in Recognition of Bacteria and Viruses | 2.67 | 0.09 | 0/109 (0%) | 0/109 (0%) | 10/109 (9%) | 99/109 (91%) |
| IL-10 Signaling | 1.99 | 0.09 | 0/78 (0%) | 0/78 (0%) | 7/78 (9%) | 71/78 (91%) |
| Neuroprotective Role of THOP1 in Alzheimer's Disease | 1.89 | 0.09 | 0/55 (0%) | 0/55 (0%) | 5/55 (9%) | 50/55 (91%) |
| Toll-like Receptor Signaling | 1.86 | 0.09 | 0/64 (0%) | 0/64 (0%) | 6/64 (9%) | 58/64 (91%) |
| Role of JAK1 and JAK3 in γc Cytokine Signaling | 1.65 | 0.09 | 0/68 (0%) | 0/68 (0%) | 6/68 (9%) | 62/68 (91%) |
| CNTF Signaling | 1.44 | 0.09 | 0/57 (0%) | 0/57 (0%) | 5/57 (9%) | 52/57 (91%) |
| PKCθ Signaling in T Lymphocytes | 7.61 | 0.13 | 7/144 (5%) | 0/144 (0%) | 12/144 (8%) | 125/144 (87%) |
| PI3K Signaling in B Lymphocytes | 3.75 | 0.10 | 3/143 (2%) | 0/143 (0%) | 11/143 (8%) | 129/143 (90%) |
| IGF-1 Signaling | 3.20 | 0.10 | 2/107 (2%) | 0/107 (0%) | 9/107 (8%) | 96/107 (90%) |
| Agranulocyte Adhesion and Diapedesis | 3.03 | 0.08 | 0/192 (0%) | 0/192 (0%) | 16/192 (8%) | 176/192 (92%) |
| FcγRIIB Signaling in B Lymphocytes | 2.52 | 0.09 | 1/64 (2%) | 0/64 (0%) | 5/64 (8%) | 58/64 (91%) |
| Reelin Signaling in Neurons | 2.17 | 0.09 | 1/85 (1%) | 0/85 (0%) | 7/85 (8%) | 77/85 (91%) |
| p38 MAPK Signaling | 2.04 | 0.08 | 1/120 (1%) | 0/120 (0%) | 9/120 (8%) | 110/120 (92%) |
| Lymphotoxin β Receptor Signaling | 1.93 | 0.10 | 1/62 (2%) | 0/62 (0%) | 5/62 (8%) | 56/62 (90%) |
| Antiproliferative Role of TOB in T Cell Signaling | 1.90 | 0.15 | 2/26 (8%) | 0/26 (0%) | 2/26 (8%) | 22/26 (85%) |
| EGF Signaling | 1.86 | 0.09 | 1/64 (2%) | 0/64 (0%) | 5/64 (8%) | 58/64 (91%) |
| FLT3 Signaling in Hematopoietic Progenitor Cells | 1.77 | 0.09 | 1/79 (1%) | 0/79 (0%) | 6/79 (8%) | 72/79 (91%) |
| IL-3 Signaling | 1.40 | 0.08 | 0/75 (0%) | 0/75 (0%) | 6/75 (8%) | 69/75 (92%) |
| Role of IL-17A in Arthritis | 1.38 | 0.08 | 0/64 (0%) | 0/64 (0%) | 5/64 (8%) | 59/64 (92%) |
| Role of Macrophages, Fibroblasts and Endothelial Cells in Rheumatoid Arthritis | 4.03 | 0.07 | 1/342 (0%) | 0/342 (0%) | 24/342 (7%) | 317/342 (93%) |
| Leukocyte Extravasation Signaling | 3.15 | 0.08 | 3/210 (1%) | 0/210 (0%) | 14/210 (7%) | 193/210 (92%) |
| Renin-Angiotensin Signaling | 2.78 | 0.09 | 2/126 (2%) | 0/126 (0%) | 9/126 (7%) | 115/126 (91%) |
| Sphingosine-1-phosphate Signaling | 2.74 | 0.09 | 3/123 (2%) | 0/123 (0%) | 8/123 (7%) | 112/123 (91%) |
| EIF2 Signaling | 2.63 | 0.07 | 1/201 (0%) | 0/201 (0%) | 14/201 (7%) | 186/201 (93%) |
| Granulocyte Adhesion and Diapedesis | 2.44 | 0.08 | 1/182 (1%) | 0/182 (0%) | 13/182 (7%) | 168/182 (92%) |
| IL-12 Signaling and Production in Macrophages | 2.09 | 0.07 | 0/157 (0%) | 0/157 (0%) | 11/157 (7%) | 146/157 (93%) |
| Tumoricidal Function of Hepatic Natural Killer Cells | 2.03 | 0.15 | 2/27 (7%) | 0/27 (0%) | 2/27 (7%) | 23/27 (85%) |
| GDNF Family Ligand-Receptor Interactions | 1.99 | 0.09 | 2/76 (3%) | 0/76 (0%) | 5/76 (7%) | 69/76 (91%) |
| Leptin Signaling in Obesity | 1.77 | 0.08 | 1/85 (1%) | 0/85 (0%) | 6/85 (7%) | 78/85 (92%) |
| IL-6 Signaling | 1.66 | 0.07 | 0/124 (0%) | 0/124 (0%) | 9/124 (7%) | 115/124 (93%) |
| Regulation of Actin-based Motility by Rho | 1.56 | 0.08 | 1/92 (1%) | 0/92 (0%) | 6/92 (7%) | 85/92 (92%) |
| April Mediated Signaling | 1.35 | 0.09 | 1/44 (2%) | 0/44 (0%) | 3/44 (7%) | 40/44 (91%) |
| Notch Signaling | 1.35 | 0.09 | 1/43 (2%) | 0/43 (0%) | 3/43 (7%) | 39/43 (91%) |
| Caveolar-mediated Endocytosis Signaling | 1.33 | 0.07 | 0/85 (0%) | 0/85 (0%) | 6/85 (7%) | 79/85 (93%) |
| Phospholipase C Signaling | 7.73 | 0.11 | 13/265 (5%) | 0/265 (0%) | 15/265 (6%) | 237/265 (89%) |
| Systemic Lupus Erythematosus Signaling | 6.42 | 0.10 | 9/256 (4%) | 0/256 (0%) | 16/256 (6%) | 231/256 (90%) |
| B Cell Receptor Signaling | 3.27 | 0.09 | 4/175 (2%) | 0/175 (0%) | 11/175 (6%) | 160/175 (91%) |
| eNOS Signaling | 2.62 | 0.08 | 3/155 (2%) | 0/155 (0%) | 9/155 (6%) | 143/155 (92%) |
| NF-κB Signaling | 2.46 | 0.08 | 3/181 (2%) | 0/181 (0%) | 11/181 (6%) | 167/181 (92%) |
| Natural Killer Cell Signaling | 2.25 | 0.08 | 3/118 (3%) | 0/118 (0%) | 7/118 (6%) | 108/118 (92%) |
| mTOR Signaling | 2.08 | 0.07 | 2/213 (1%) | 0/213 (0%) | 12/213 (6%) | 199/213 (93%) |
| Production of Nitric Oxide and Reactive Oxygen Species in Macrophages | 1.94 | 0.06 | 0/212 (0%) | 0/212 (0%) | 13/212 (6%) | 199/212 (94%) |
| Clathrin-mediated Endocytosis Signaling | 1.83 | 0.07 | 2/198 (1%) | 0/198 (0%) | 11/198 (6%) | 185/198 (93%) |
| Amyotrophic Lateral Sclerosis Signaling | 1.62 | 0.06 | 1/126 (1%) | 0/126 (0%) | 7/126 (6%) | 118/126 (94%) |
| Virus Entry via Endocytic Pathways | 1.42 | 0.07 | 1/101 (1%) | 0/101 (0%) | 6/101 (6%) | 94/101 (93%) |
| VEGF Signaling | 1.35 | 0.06 | 0/109 (0%) | 0/109 (0%) | 7/109 (6%) | 102/109 (94%) |
| Docosahexaenoic Acid (DHA) Signaling | 1.32 | 0.08 | 1/50 (2%) | 0/50 (0%) | 3/50 (6%) | 46/50 (92%) |
| p53 Signaling | 4.38 | 0.12 | 7/113 (6%) | 0/113 (0%) | 6/113 (5%) | 100/113 (88%) |
| CTLA4 Signaling in Cytotoxic T Lymphocytes | 4.21 | 0.13 | 7/96 (7%) | 0/96 (0%) | 5/96 (5%) | 84/96 (88%) |
| Pancreatic Adenocarcinoma Signaling | 3.39 | 0.09 | 5/128 (4%) | 0/128 (0%) | 7/128 (5%) | 116/128 (91%) |
| Glucocorticoid Receptor Signaling | 2.98 | 0.07 | 5/299 (2%) | 0/299 (0%) | 15/299 (5%) | 279/299 (93%) |
| Thrombin Signaling | 2.47 | 0.07 | 4/211 (2%) | 0/211 (0%) | 11/211 (5%) | 196/211 (93%) |
| Tec Kinase Signaling | 2.43 | 0.07 | 3/184 (2%) | 0/184 (0%) | 10/184 (5%) | 171/184 (93%) |
| ATM Signaling | 2.29 | 0.11 | 4/66 (6%) | 0/66 (0%) | 3/66 (5%) | 59/66 (89%) |
| Glioblastoma Multiforme Signaling | 2.21 | 0.07 | 4/168 (2%) | 0/168 (0%) | 8/168 (5%) | 156/168 (93%) |
| Cellular Effects of Sildenafil (Viagra) | 2.21 | 0.07 | 3/155 (2%) | 0/155 (0%) | 8/155 (5%) | 144/155 (93%) |
| Endothelin-1 Signaling | 2.08 | 0.07 | 4/192 (2%) | 0/192 (0%) | 9/192 (5%) | 179/192 (93%) |
| Corticotropin Releasing Hormone Signaling | 1.72 | 0.06 | 2/145 (1%) | 0/145 (0%) | 7/145 (5%) | 136/145 (94%) |
| Cardiac Hypertrophy Signaling | 1.53 | 0.06 | 1/250 (0%) | 0/250 (0%) | 13/250 (5%) | 236/250 (94%) |
| Regulation of eIF4 and p70S6K Signaling | 1.42 | 0.06 | 1/175 (1%) | 0/175 (0%) | 9/175 (5%) | 165/175 (94%) |
| Gαq Signaling | 1.42 | 0.06 | 2/171 (1%) | 0/171 (0%) | 8/171 (5%) | 161/171 (94%) |
| Role of Tissue Factor in Cancer | 1.36 | 0.06 | 2/130 (2%) | 0/130 (0%) | 6/130 (5%) | 122/130 (94%) |
| T Cell Receptor Signaling | 5.10 | 0.13 | 10/109 (9%) | 0/109 (0%) | 4/109 (4%) | 95/109 (87%) |
| Cell Cycle: G1/S Checkpoint Regulation | 4.73 | 0.15 | 8/72 (11%) | 0/72 (0%) | 3/72 (4%) | 61/72 (85%) |
| Aryl Hydrocarbon Receptor Signaling | 3.34 | 0.08 | 7/171 (4%) | 0/171 (0%) | 7/171 (4%) | 157/171 (92%) |
| Regulation of IL-2 Expression in Activated and Anergic T Lymphocytes | 3.34 | 0.11 | 6/89 (7%) | 0/89 (0%) | 4/89 (4%) | 79/89 (89%) |
| Estrogen-mediated S-phase Entry | 2.88 | 0.18 | 4/28 (14%) | 0/28 (0%) | 1/28 (4%) | 23/28 (82%) |
| Glioma Signaling | 2.19 | 0.08 | 4/113 (4%) | 0/113 (0%) | 5/113 (4%) | 104/113 (92%) |
| Non-Small Cell Lung Cancer Signaling | 2.10 | 0.08 | 4/83 (5%) | 0/83 (0%) | 3/83 (4%) | 76/83 (92%) |
| Prostate Cancer Signaling | 2.04 | 0.08 | 4/103 (4%) | 0/103 (0%) | 4/103 (4%) | 95/103 (92%) |
| Small Cell Lung Cancer Signaling | 1.90 | 0.07 | 3/94 (3%) | 0/94 (0%) | 4/94 (4%) | 87/94 (93%) |
| Role of NFAT in Cardiac Hypertrophy | 1.89 | 0.06 | 4/209 (2%) | 0/209 (0%) | 9/209 (4%) | 196/209 (94%) |
| Protein Kinase A Signaling | 1.75 | 0.05 | 5/409 (1%) | 0/409 (0%) | 17/409 (4%) | 387/409 (95%) |
| Gap Junction Signaling | 1.64 | 0.06 | 4/181 (2%) | 0/181 (0%) | 7/181 (4%) | 170/181 (94%) |
| Hepatic Cholestasis | 1.58 | 0.05 | 2/183 (1%) | 0/183 (0%) | 8/183 (4%) | 173/183 (95%) |
| IL-8 Signaling | 1.46 | 0.05 | 2/225 (1%) | 0/225 (0%) | 10/225 (4%) | 213/225 (95%) |
| RAR Activation | 1.30 | 0.06 | 3/195 (2%) | 0/195 (0%) | 8/195 (4%) | 184/195 (94%) |
| Hereditary Breast Cancer Signaling | 2.09 | 0.07 | 6/134 (4%) | 0/134 (0%) | 4/134 (3%) | 124/134 (93%) |
| Molecular Mechanisms of Cancer | 1.97 | 0.05 | 10/388 (3%) | 0/388 (0%) | 11/388 (3%) | 367/388 (95%) |
| CCR5 Signaling in Macrophages | 1.93 | 0.07 | 4/97 (4%) | 0/97 (0%) | 3/97 (3%) | 90/97 (93%) |
| Chronic Myeloid Leukemia Signaling | 1.74 | 0.08 | 5/106 (5%) | 0/106 (0%) | 3/106 (3%) | 98/106 (92%) |
| Cell Cycle Regulation by BTG Family Proteins | 1.47 | 0.10 | 3/39 (8%) | 0/39 (0%) | 1/39 (3%) | 35/39 (90%) |
| Protein Ubiquitination Pathway | 1.41 | 0.06 | 6/270 (2%) | 0/270 (0%) | 9/270 (3%) | 255/270 (94%) |
| Cyclins and Cell Cycle Regulation | 3.29 | 0.10 | 8/96 (8%) | 0/96 (0%) | 2/96 (2%) | 86/96 (90%) |
| Cell Cycle Control of Chromosomal Replication | 3.43 | 0.18 | 6/34 (18%) | 0/34 (0%) | 0/34 (0%) | 28/34 (82%) |
| DNA Methylation and Transcriptional Repression Signaling | 1.49 | 0.13 | 3/23 (13%) | 0/23 (0%) | 0/23 (0%) | 20/23 (87%) |
