# Supplementary material for: Genomic deletion of GIT2 induces a premature age-related thymic dysfunction and systemic immune system disruption
Source: Aging (Albany NY). 2017 Mar 4;9(3):706–30. doi: 10.18632/aging.101185 (PMC5391227; doi:10.18632/aging.101185)
Supplement: Supplementary file 11 [file aging-09-706-s011.docx]

**Table S10. Transcripts significantly regulated differentially between the GIT2KO inguinal lymph node (ILN) and WT ILN.** The official Gene Symbol, transcript description and associated Z ratios for the comparison of the GIT2KO ILN *vs.* WT ILN at the 12 month time point are indicated. Each transcript was significantly regulated at p<0.05, with a Z ratio >± 1.5.

| **Symbol** | **Description** | **Z ratio** |
| --- | --- | --- |
| Ctse | cathepsin E (Ctse) | 14.23 |
| Kras | v-Ki-ras2 Kirsten rat sarcoma viral oncogene homolog (Kras) | 7.67 |
| H2-T10 | histocompatibility 2, T region locus 10 (H2-T10) | 7.08 |
| Pik3cg | phosphoinositide-3-kinase, catalytic, gamma polypeptide (Pik3cg) | 6.03 |
| Hp | haptoglobin (Hp) | 6 |
| Hp | haptoglobin (Hp) | 5.8 |
| LOC100038882 | hypothetical protein LOC100038882 (LOC100038882) | 5.41 |
| St6galnac2 | ST6 (alpha-N-acetyl-neuraminyl-2,3-beta-galactosyl-1, 3)-N-acetylgalactosaminide alpha-2,6-sialyltransferase 2 (St6galnac2) | 5.4 |
| Cxcl9 | chemokine (C-X-C motif) ligand 9 (Cxcl9) | 4.91 |
| Lpl | lipoprotein lipase (Lpl) | 4.57 |
| Gfer | growth factor, erv1 (S. cerevisiae)-like (augmenter of liver regeneration) (Gfer) | 4.25 |
| Ndufb10 | NADH dehydrogenase (ubiquinone) 1 beta subcomplex, 10 (Ndufb10) | 4.21 |
| Scd1 | stearoyl-Coenzyme A desaturase 1 (Scd1) | 4.18 |
| Cxcl13 | chemokine (C-X-C motif) ligand 13 (Cxcl13) | 4.17 |
| Vegfa | vascular endothelial growth factor A (Vegfa), transcript variant 2 | 4.15 |
| Hspa8 | heat shock protein 8 (Hspa8) | 4.08 |
| Lyz1 | lysozyme 1 (Lyz1) | 4.06 |
| Glo1 | glyoxalase 1 (Glo1) | 4.01 |
| Fgd2 | FYVE, RhoGEF and PH domain containing 2 (Fgd2) | 3.94 |
| LOC100043671 | hypothetical protein LOC100043671 (LOC100043671) | 3.93 |
| Cbr2 | carbonyl reductase 2 (Cbr2) | 3.9 |
| Tmem66 | transmembrane protein 66 (Tmem66) | 3.88 |
| Adi1 | acireductone dioxygenase 1 (Adi1) | 3.79 |
| Apoc1 | apolipoprotein C-I (Apoc1) | 3.74 |
| Sqle | squalene epoxidase (Sqle) | 3.62 |
| Lyz2 | lysozyme 2 (Lyz2) | 3.6 |
| Ifi27 | interferon, alpha-inducible protein 27 (Ifi27) | 3.58 |
| LOC100048346 | similar to ubiquitin specific protease UBP43 (LOC100048346) | 3.57 |
| Ffar2 | free fatty acid receptor 2 (Ffar2) | 3.57 |
| Usp18 | ubiquitin specific peptidase 18 (Usp18) | 3.49 |
| Rsad2 | radical S-adenosyl methionine domain containing 2 (Rsad2) | 3.45 |
| Hnrpk | heterogeneous nuclear ribonucleoprotein K (Hnrpk) | 3.41 |
| Actb | actin, beta, cytoplasmic (Actb) | 3.39 |
| Stat1 | signal transducer and activator of transcription 1 (Stat1) | 3.35 |
| Hebp1 | heme binding protein 1 (Hebp1) | 3.33 |
| Cav1 | caveolin, caveolae protein 1 (Cav1) | 3.23 |
| Cd84 | CD84 antigen (Cd84) | 3.23 |
| Phlda1 | pleckstrin homology-like domain, family A, member 1 (Phlda1) | 3.22 |
| Clec4n | C-type lectin domain family 4, member n (Clec4n) | 3.19 |
| 9130213B05Rik | RIKEN cDNA 9130213B05 gene (9130213B05Rik) | 3.18 |
| Pdia4 | protein disulfide isomerase associated 4 (Pdia4) | 3.17 |
| Gng10 | guanine nucleotide binding protein (G protein), gamma 10 (Gng10) | 3.14 |
| Aoc3 | amine oxidase, copper containing 3 (Aoc3) | 3.11 |
| Cap1 | CAP, adenylate cyclase-associated protein 1 (yeast) (Cap1) | 3.1 |
| B3gnt5 | UDP-GlcNAc:betaGal beta-1,3-N-acetylglucosaminyltransferase 5 (B3gnt5) | 3.07 |
| H2-DMb2 | histocompatibility 2, class II, locus Mb2 (H2-DMb2) | 3.06 |
| H2-M2 | histocompatibility 2, M region locus 2 (H2-M2) | 3.05 |
| Hnrpa1 | heterogeneous nuclear ribonucleoprotein A1 (Hnrpa1), transcript variant 2 | 3.05 |
| Asns | asparagine synthetase (Asns) | 3 |
| Lyzs | lysozyme (Lyzs) | 2.93 |
| Loxl1 | lysyl oxidase-like 1 (Loxl1) | 2.9 |
| Eno1 | enolase 1, alpha non-neuron (Eno1) | 2.85 |
| Fh1 | fumarate hydratase 1 (Fh1) | 2.82 |
| Ppa1 | pyrophosphatase (inorganic) 1 (Ppa1) | 2.74 |
| 5830472M02Rik | RIKEN cDNA 5830472M02 gene (5830472M02Rik), transcript variant 1 | 2.74 |
| Eif5a | eukaryotic translation initiation factor 5A (Eif5a) | 2.73 |
| Srpk3 | serine/arginine-rich protein specific kinase 3 (Srpk3) | 2.73 |
| Pfn1 | profilin 1 (Pfn1) | 2.71 |
| Cd274 | CD274 antigen (Cd274) | 2.68 |
| 4632417K18Rik | RIKEN cDNA 4632417K18 gene (4632417K18Rik) | 2.67 |
| Tacstd2 | tumor-associated calcium signal transducer 2 (Tacstd2) | 2.65 |
| BC006779 | cDNA sequence BC006779 (BC006779) | 2.64 |
| Cd79b | CD79B antigen (Cd79b) | 2.63 |
| Hnrnpa2b1 | heterogeneous nuclear ribonucleoprotein A2/B1 (Hnrnpa2b1), transcript variant 2 | 2.62 |
| LOC100040592 | similar to Hmgcs1 protein, transcript variant 1 (LOC100040592) | 2.61 |
| Socs3 | suppressor of cytokine signaling 3 (Socs3) | 2.6 |
| Col6a1 | procollagen, type VI, alpha 1 (Col6a1) | 2.59 |
| Ptp4a2 | protein tyrosine phosphatase 4a2 (Ptp4a2) | 2.58 |
| Cnot7 | CCR4-NOT transcription complex, subunit 7 (Cnot7) | 2.58 |
| Cd9 | CD9 antigen (Cd9) | 2.58 |
| Nap1l1 | nucleosome assembly protein 1-like 1 (Nap1l1) | 2.58 |
| Snx30 | sorting nexin family member 30 (Snx30) | 2.57 |
| Igfbp5 | insulin-like growth factor binding protein 5 (Igfbp5) | 2.51 |
| Eif2ak2 | eukaryotic translation initiation factor 2-alpha kinase 2 (Eif2ak2) | 2.49 |
| Fcrla | Fc receptor-like A (Fcrla) | 2.49 |
| Hsp90ab1 | heat shock protein 90kDa alpha (cytosolic), class B member 1 (Hsp90ab1) | 2.49 |
| EG434858 | predicted gene, EG434858 (EG434858) on chromosome X. | 2.48 |
| 1200002N14Rik | RIKEN cDNA 1200002N14 gene (1200002N14Rik) | 2.46 |
| Tnfrsf13c | tumor necrosis factor receptor superfamily, member 13c (Tnfrsf13c) | 2.46 |
| Ppp1ca | protein phosphatase 1, catalytic subunit, alpha isoform (Ppp1ca) | 2.41 |
| Arhgap24 | Rho GTPase activating protein 24 (Arhgap24), transcript variant 1 | 2.41 |
| Sec61b | Sec61 beta subunit (Sec61b) | 2.4 |
| Cxcr5 | chemochine (C-X-C motif) receptor 5 (Cxcr5) | 2.39 |
| Lst1 | leukocyte specific transcript 1 (Lst1) | 2.38 |
| Sh3bgrl | SH3-binding domain glutamic acid-rich protein like (Sh3bgrl) | 2.37 |
| Ccr6 | chemokine (C-C motif) receptor 6 (Ccr6) | 2.37 |
| Idh3g | isocitrate dehydrogenase 3 (NAD+), gamma (Idh3g), nuclear gene encoding mitochondrial protein | 2.35 |
| Sfrs7 | splicing factor, arginine/serine-rich 7 (Sfrs7) | 2.35 |
| Stx8 | syntaxin 8 (Stx8) | 2.34 |
| Tank | TRAF family member-associated Nf-kappa B activator (Tank) | 2.34 |
| Sparc | secreted acidic cysteine rich glycoprotein (Sparc) | 2.34 |
| Rps27a | ribosomal protein S27a (Rps27a), transcript variant 2 | 2.33 |
| Rasgrp3 | RAS, guanyl releasing protein 3 (Rasgrp3) | 2.33 |
| Vars2 | valyl-tRNA synthetase 2, mitochondrial (putative) (Vars2) | 2.31 |
| Ubd | ubiquitin D (Ubd) | 2.29 |
| Ly6d | lymphocyte antigen 6 complex, locus D (Ly6d) | 2.25 |
| LOC100047934 | hypothetical protein LOC100047934 (LOC100047934) | 2.25 |
| Des | desmin (Des) | 2.24 |
| LOC100047155 | similar to Small nuclear ribonucleoprotein polypeptide A (LOC100047155) | 2.23 |
| Lrg1 | leucine-rich alpha-2-glycoprotein 1 (Lrg1) | 2.22 |
| Msn | moesin (Msn) | 2.2 |
| Gstm2 | glutathione S-transferase, mu 2 (Gstm2) | 2.2 |
| Sh3bp2 | SH3-domain binding protein 2 (Sh3bp2) | 2.2 |
| Zfp238 | zinc finger protein 238 (Zfp238), transcript variant 2 | 2.19 |
| Atp5f1 | ATP synthase, H+ transporting, mitochondrial F0 complex, subunit b, isoform 1 (Atp5f1) | 2.19 |
| Unc45a | unc-45 homolog A (C. elegans) (Unc45a) | 2.17 |
| Gnb1 | guanine nucleotide binding protein (G protein), beta 1 (Gnb1) | 2.16 |
| Emp2 | epithelial membrane protein 2 (Emp2) | 2.16 |
| Caprin1 | cell cycle associated protein 1 (Caprin1) | 2.15 |
| Pfn1 | profilin 1 (Pfn1) | 2.15 |
| H2-Ab1 | histocompatibility 2, class II antigen A, beta 1 (H2-Ab1) | 2.14 |
| Swap70 | SWA-70 protein (Swap70) | 2.14 |
| Sfrs5 | splicing factor, arginine/serine-rich 5 (SRp40, HRS) (Sfrs5), transcript variant 2 | 2.13 |
| Csrp1 | cysteine and glycine-rich protein 1 (Csrp1) | 2.13 |
| Nudt4 | nudix (nucleoside diphosphate linked moiety X)-type motif 4 (Nudt4) | 2.13 |
| Vwf | Von Willebrand factor homolog (Vwf) | 2.13 |
| Hsp90ab1 | heat shock protein 90kDa alpha (cytosolic), class B member 1 (Hsp90ab1) | 2.12 |
| Abi3 | ABI gene family, member 3 (Abi3) | 2.12 |
| Lmtk2 | lemur tyrosine kinase 2 (Lmtk2) | 2.11 |
| H2-Ob | histocompatibility 2, O region beta locus (H2-Ob) | 2.11 |
| EG433923 | predicted gene, EG433923 (EG433923) | 2.11 |
| Hist1h2ak | histone cluster 1, H2ak (Hist1h2ak) | 2.09 |
| LOC100047963 | similar to ADIR1 (LOC100047963) | 2.09 |
| Cd55 | CD55 antigen (Cd55) | 2.08 |
| Nudt5 | nudix (nucleoside diphosphate linked moiety X)-type motif 5 (Nudt5) | 2.08 |
| Hhex | hematopoietically expressed homeobox (Hhex) | 2.08 |
| Cd22 | CD22 antigen (Cd22), transcript variant 2 | 2.05 |
| Txnl4a | thioredoxin-like 4A (Txnl4a), transcript variant 2 | 2.04 |
| Ctsc | cathepsin C (Ctsc) | 2.04 |
| Cnr2 | cannabinoid receptor 2 (macrophage) (Cnr2) | 2 |
| Tuba1a | tubulin, alpha 1A (Tuba1a) | 2 |
| Oxct1 | 3-oxoacid CoA transferase 1 (Oxct1) | 1.99 |
| Mthfd1 | methylenetetrahydrofolate dehydrogenase (NADP+ dependent), methenyltetrahydrofolate cyclohydrolase, formyltetrahydrofolate synthase (Mthfd1) | 1.99 |
| Uck2 | uridine-cytidine kinase 2 (Uck2) | 1.98 |
| Mef2c | myocyte enhancer factor 2C (Mef2c) | 1.98 |
| Efnb1 | ephrin B1 (Efnb1) | 1.98 |
| Dhx15 | DEAH (Asp-Glu-Ala-His) box polypeptide 15 (Dhx15), transcript variant 1 | 1.96 |
| Inppl1 | inositol polyphosphate phosphatase-like 1 (Inppl1) | 1.96 |
| Ywhag | tyrosine 3-monooxygenase/tryptophan 5-monooxygenase activation protein, gamma polypeptide (Ywhag) | 1.95 |
| Mylk | myosin, light polypeptide kinase (Mylk) | 1.94 |
| Rnf11 | ring finger protein 11 (Rnf11) | 1.94 |
| Ccnd1 | cyclin D1 (Ccnd1) | 1.93 |
| Pbrm1 | polybromo 1 (Pbrm1) | 1.93 |
| Tubb2c | tubulin, beta 2c (Tubb2c) | 1.93 |
| Sfrs2 | splicing factor, arginine/serine-rich 2 (SC-35) (Sfrs2) | 1.93 |
| Sdhc | succinate dehydrogenase complex, subunit C, integral membrane protein (Sdhc), nuclear gene encoding mitochondrial protein | 1.93 |
| Sep15 | selenoprotein (Sep15) | 1.92 |
| Il33 | interleukin 33 (Il33) | 1.91 |
| Sphk1 | sphingosine kinase 1 (Sphk1), transcript variant 1 | 1.9 |
| Gstm2 | glutathione S-transferase, mu 2 (Gstm2) | 1.9 |
| Plac8 | placenta-specific 8 (Plac8) | 1.89 |
| Gch1 | GTP cyclohydrolase 1 (Gch1) | 1.89 |
| Arpc5 | actin related protein 2/3 complex, subunit 5 (Arpc5) | 1.88 |
| Napsa | napsin A aspartic peptidase (Napsa) | 1.88 |
| Rb1 | retinoblastoma 1 (Rb1) | 1.88 |
| Asb2 | ankyrin repeat and SOCS box-containing protein 2 (Asb2) | 1.86 |
| Tdrd7 | tudor domain containing 7 (Tdrd7) | 1.85 |
| Ndufc1 | NADH dehydrogenase (ubiquinone) 1, subcomplex unknown, 1 (Ndufc1) | 1.84 |
| 2610204L23Rik | coiled-coil domain containing 47 (Ccdc47) | 1.84 |
| Prkcb1 | protein kinase C, beta 1 (Prkcb1) | 1.84 |
| Vdac2 | voltage-dependent anion channel 2 (Vdac2) | 1.84 |
| Sepx1 | selenoprotein X 1 (Sepx1) | 1.84 |
| Lxn | latexin (Lxn) | 1.83 |
| Rnf11 | ring finger protein 11 (Rnf11) | 1.83 |
| Eif5 | eukaryotic translation initiation factor 5 (Eif5), transcript variant 2 | 1.82 |
| Mrpl3 | mitochondrial ribosomal protein L3 (Mrpl3), nuclear gene encoding mitochondrial protein | 1.82 |
| LOC100046855 | similar to BKLF (LOC100046855) | 1.82 |
| Adam17 | a disintegrin and metallopeptidase domain 17 (Adam17) | 1.82 |
| Mef2c | myocyte enhancer factor 2C (Mef2c) | 1.81 |
| 4833426J09Rik | RIKEN cDNA 4833426J09 gene (4833426J09Rik) | 1.79 |
| Gtf2e1 | general transcription factor II E, polypeptide 1 (alpha subunit) (Gtf2e1) | 1.78 |
| Chrnb1 | cholinergic receptor, nicotinic, beta polypeptide 1 (muscle) (Chrnb1) | 1.78 |
| Mylc2b | myosin light chain, regulatory B (Mylc2b) | 1.78 |
| Sorl1 | sortilin-related receptor, LDLR class A repeats-containing (Sorl1) | 1.77 |
| Gpd2 | glycerol phosphate dehydrogenase 2, mitochondrial (Gpd2), nuclear gene encoding mitochondrial protein | 1.77 |
| Galnt11 | UDP-N-acetyl-alpha-D-galactosamine:polypeptide N-acetylgalactosaminyltransferase 11 (Galnt11) | 1.76 |
| Psmd7 | proteasome (prosome, macropain) 26S subunit, non-ATPase, 7 (Psmd7) | 1.76 |
| Sparc | secreted acidic cysteine rich glycoprotein (Sparc) | 1.76 |
| Arpc1a | actin related protein 2/3 complex, subunit 1A (Arpc1a) | 1.74 |
| Ppp2r5c | protein phosphatase 2, regulatory subunit B (B56), gamma isoform (Ppp2r5c), transcript variant 3 | 1.74 |
| Smu1 | smu-1 suppressor of mec-8 and unc-52 homolog (C. elegans) (Smu1) | 1.74 |
| 2310008M10Rik | RIKEN cDNA 2310008M10 gene (2310008M10Rik) | 1.74 |
| Slc15a3 | solute carrier family 15, member 3 (Slc15a3) | 1.73 |
| Hspd1 | heat shock protein 1 (chaperonin) (Hspd1) | 1.73 |
| Hmgn2 | high mobility group nucleosomal binding domain 2 (Hmgn2) | 1.73 |
| Ndn | necdin (Ndn) | 1.73 |
| Col4a2 | collagen, type IV, alpha 2 (Col4a2) | 1.72 |
| Ndufa12 | NADH dehydrogenase (ubiquinone) 1 alpha subcomplex, 12 (Ndufa12) | 1.72 |
| Hnrph1 | heterogeneous nuclear ribonucleoprotein H1 (Hnrph1) | 1.71 |
| H2-Q8 | histocompatibility 2, Q region locus 8 (H2-Q8) | 1.71 |
| Adamts2 | a disintegrin-like and metallopeptidase (reprolysin type) with thrombospondin type 1 motif, 2 (Adamts2) | 1.71 |
| Tmem93 | transmembrane protein 93 (Tmem93) | 1.71 |
| Aacs | acetoacetyl-CoA synthetase (Aacs) | 1.7 |
| Gnas | GNAS (guanine nucleotide binding protein, alpha stimulating) complex locus (Gnas), transcript variant 3 | 1.7 |
| Plcg2 | phospholipase C, gamma 2 (Plcg2) | 1.69 |
| H3f3a | H3 histone, family 3A (H3f3a) | 1.69 |
| Tpi1 | triosephosphate isomerase 1 (Tpi1) | 1.68 |
| Swap70 | SWA-70 protein (Swap70) | 1.68 |
| Rnase4 | ribonuclease, RNase A family 4 (Rnase4), transcript variant 1 | 1.68 |
| Tgfbr1 | transforming growth factor, beta receptor I (Tgfbr1) | 1.67 |
| Slc38a2 | solute carrier family 38, member 2 (Slc38a2) | 1.67 |
| Fcrla | Fc receptor-like A (Fcrla) | 1.67 |
| Mylc2b | myosin light chain, regulatory B (Mylc2b) | 1.66 |
| Pfdn4 | prefoldin 4 (Pfdn4), transcript variant 2 | 1.66 |
| Bhlhb2 | basic helix-loop-helix domain containing, class B2 (Bhlhb2) | 1.65 |
| Mfge8 | milk fat globule-EGF factor 8 protein (Mfge8) | 1.65 |
| Hnrph1 | heterogeneous nuclear ribonucleoprotein H1 (Hnrph1) | 1.64 |
| H2-K1 | histocompatibility 2, K1, K region (H2-K1) | 1.64 |
| Hsd17b12 | hydroxysteroid (17-beta) dehydrogenase 12 (Hsd17b12) | 1.64 |
| Fes | feline sarcoma oncogene (Fes) | 1.64 |
| Snrpd1 | small nuclear ribonucleoprotein D1 (Snrpd1) | 1.64 |
| Coro1a | coronin, actin binding protein 1A (Coro1a) | 1.64 |
| Dhrs1 | dehydrogenase/reductase (SDR family) member 1 (Dhrs1) | 1.63 |
| Mgst1 | microsomal glutathione S-transferase 1 (Mgst1) | 1.63 |
| Snap23 | synaptosomal-associated protein 23 (Snap23) | 1.62 |
| Pno1 | partner of NOB1 homolog (S. cerevisiae) (Pno1) | 1.61 |
| Ifi47 | interferon gamma inducible protein 47 (Ifi47) | 1.6 |
| Inppl1 | inositol polyphosphate phosphatase-like 1 (Inppl1) | 1.6 |
| Cdc42 | cell division cycle 42 homolog (S. cerevisiae) (Cdc42) | 1.58 |
| Fah | fumarylacetoacetate hydrolase (Fah) | 1.58 |
| Rpa3 | replication protein A3 (Rpa3) | 1.58 |
| Ccr6 | chemokine (C-C motif) receptor 6 (Ccr6) | 1.58 |
| Fdps | farnesyl diphosphate synthetase (Fdps) | 1.58 |
| Plod3 | procollagen-lysine, 2-oxoglutarate 5-dioxygenase 3 (Plod3) | 1.58 |
| Nola2 | nucleolar protein family A, member 2 (Nola2) | 1.57 |
| Swap70 | SWA-70 protein (Swap70) | 1.57 |
| D3Ucla1 | DNA segment, Chr 3, University of California at Los Angeles 1 (D3Ucla1) | 1.56 |
| Ccdc41 | coiled-coil domain containing 41 (Ccdc41) | 1.56 |
| Coq5 | coenzyme Q5 homolog, methyltransferase (yeast) (Coq5) | 1.56 |
| LOC100045617 | similar to Eukaryotic translation initiation factor 4A2 (LOC100045617) | 1.55 |
| Cd2bp2 | CD2 antigen (cytoplasmic tail) binding protein 2 (Cd2bp2) | 1.55 |
| Ddx54 | DEAD (Asp-Glu-Ala-Asp) box polypeptide 54 (Ddx54) | 1.55 |
| Col4a1 | procollagen, type IV, alpha 1 (Col4a1) | 1.54 |
| Cd40 | CD40 antigen (Cd40), transcript variant 5 | 1.54 |
| Caprin1 | cell cycle associated protein 1 (Caprin1) | 1.53 |
| Gstm2 | glutathione S-transferase, mu 2 (Gstm2) | 1.53 |
| B3gnt8 | UDP-GlcNAc:betaGal beta-1,3-N-acetylglucosaminyltransferase 8 (B3gnt8), transcript variant 1 | 1.53 |
| Dcp1a | DCP1 decapping enzyme homolog A (S. cerevisiae) (Dcp1a) | 1.53 |
| Uso1 | USO1 homolog, vesicle docking protein (yeast) (Uso1) | 1.52 |
| Ythdf2 | YTH domain family 2 (Ythdf2) | 1.52 |
| Stard4 | StAR-related lipid transfer (START) domain containing 4 (Stard4) | 1.52 |
| Cd44 | CD44 antigen (Cd44), transcript variant 2 | 1.52 |
| Pcbp1 | poly(rC) binding protein 1 (Pcbp1) | 1.51 |
| Lcp1 | lymphocyte cytosolic protein 1 (Lcp1) | 1.5 |
| Atf7ip | activating transcription factor 7 interacting protein (Atf7ip) | -1.5 |
| Smad1 | MAD homolog 1 (Drosophila) (Smad1) | -1.51 |
| Tgfbi | transforming growth factor, beta induced (Tgfbi) | -1.51 |
| Brd2 | bromodomain containing 2 (Brd2), transcript variant 1 | -1.52 |
| Ehmt2 | euchromatic histone lysine N-methyltransferase 2 (Ehmt2), transcript variant short | -1.52 |
| Ei24 | etoposide induced 2.4 mRNA (Ei24) | -1.52 |
| Cd6 | CD6 antigen (Cd6), transcript variant 2 | -1.52 |
| D10Ertd641e | DNA segment, Chr 10, ERATO Doi 641, expressed (D10Ertd641e) | -1.53 |
| Dgkz | diacylglycerol kinase zeta (Dgkz) | -1.54 |
| Golm1 | golgi membrane protein 1 (Golm1), transcript variant 1 | -1.54 |
| ORF61 | open reading frame 61 (ORF61) | -1.55 |
| Map3k7ip1 | mitogen-activated protein kinase kinase kinase 7 interacting protein 1 (Map3k7ip1) | -1.55 |
| Aff1 | AF4/FMR2 family, member 1 (Aff1), transcript variant 2 | -1.55 |
| Ccs | copper chaperone for superoxide dismutase (Ccs) | -1.56 |
| Gtf3c2 | general transcription factor IIIC, polypeptide 2, beta (Gtf3c2) | -1.56 |
| Ramp2 | receptor (calcitonin) activity modifying protein 2 (Ramp2) | -1.58 |
| Tnrc6a | trinucleotide repeat containing 6a (Tnrc6a) | -1.59 |
| Hp1bp3 | heterochromatin protein 1, binding protein 3 (Hp1bp3) | -1.59 |
| Zfyve21 | zinc finger, FYVE domain containing 21 (Zfyve21) | -1.6 |
| Bcl11b | B-cell leukemia/lymphoma 11B (Bcl11b), transcript variant 2 | -1.6 |
| Entpd4 | ectonucleoside triphosphate diphosphohydrolase 4 (Entpd4) | -1.6 |
| H2afx | H2A histone family, member X (H2afx) | -1.61 |
| Acaa2 | acetyl-Coenzyme A acyltransferase 2 (mitochondrial 3-oxoacyl-Coenzyme A thiolase) (Acaa2) | -1.61 |
| Slc11a1 | solute carrier family 11 (proton-coupled divalent metal ion transporters), member 1 (Slc11a1) | -1.61 |
| Wdr45 | WD repeat domain 45 (Wdr45) | -1.61 |
| Ankle2 | ankyrin repeat and LEM domain containing 2 (Ankle2) | -1.62 |
| Ccl9 | chemokine (C-C motif) ligand 9 (Ccl9) | -1.62 |
| Acox3 | acyl-Coenzyme A oxidase 3, pristanoyl (Acox3) | -1.63 |
| Add1 | adducin 1 (alpha) (Add1), transcript variant 1 | -1.63 |
| Centd3 | centaurin, delta 3 (Centd3) | -1.63 |
| Slc6a6 | solute carrier family 6 (neurotransmitter transporter, taurine), member 6 (Slc6a6) | -1.64 |
| Arhgef1 | Rho guanine nucleotide exchange factor (GEF) 1 (Arhgef1) | -1.66 |
| Fbxo34 | F-box protein 34 (Fbxo34) | -1.66 |
| Pik3r1 | phosphatidylinositol 3-kinase, regulatory subunit, polypeptide 1 (p85 alpha) (Pik3r1), transcript variant 1 | -1.66 |
| Pacs2 | phosphofurin acidic cluster sorting protein 2 (Pacs2) | -1.66 |
| Cbx7 | chromobox homolog 7 (Cbx7) | -1.67 |
| Fxyd6 | FXYD domain-containing ion transport regulator 6 (Fxyd6) | -1.67 |
| Ttc3 | tetratricopeptide repeat domain 3 (Ttc3) | -1.67 |
| Ascc1 | activating signal cointegrator 1 complex subunit 1 (Ascc1) | -1.67 |
| Cxcr4 | chemokine (C-X-C motif) receptor 4 (Cxcr4) | -1.68 |
| Cyhr1 | cysteine and histidine rich 1 (Cyhr1), transcript variant 2 | -1.68 |
| Igf2r | insulin-like growth factor 2 receptor (Igf2r) | -1.68 |
| Ttc3 | tetratricopeptide repeat domain 3 (Ttc3) | -1.7 |
| Mmrn2 | multimerin 2 (Mmrn2) | -1.7 |
| Srd5a3 | steroid 5 alpha-reductase 3 (Srd5a3) | -1.71 |
| Fads1 | fatty acid desaturase 1 (Fads1) | -1.71 |
| Usp3 | ubiquitin specific peptidase 3 (Usp3) | -1.72 |
| Vars | valyl-tRNA synthetase (Vars) | -1.73 |
| Mfap3 | microfibrillar-associated protein 3 (Mfap3), transcript variant 2 | -1.75 |
| BC025076 | membrane magnesium transporter 2 (Mmgt2) | -1.75 |
| C2 | complement component 2 (within H-2S) (C2) | -1.75 |
| Leprotl1 | leptin receptor overlapping transcript-like 1 (Leprotl1) | -1.77 |
| Pde1b | phosphodiesterase 1B, Ca2+-calmodulin dependent (Pde1b) | -1.77 |
| Adcy4 | adenylate cyclase 4 (Adcy4) | -1.77 |
| Mllt3 | myeloid/lymphoid or mixed-lineage leukemia (trithorax homolog, Drosophila); translocated to, 3 (Mllt3), transcript variant 1 | -1.78 |
| Sh3tc1 | SH3 domain and tetratricopeptide repeats 1 (Sh3tc1) | -1.78 |
| Cenpa | centromere protein A (Cenpa) | -1.79 |
| Itgb7 | integrin beta 7 (Itgb7) | -1.79 |
| Mrpl48 | mitochondrial ribosomal protein L48 (Mrpl48), nuclear gene encoding mitochondrial protein | -1.79 |
| Egfl7 | EGF-like domain 7 (Egfl7), transcript variant b | -1.79 |
| Gprasp1 | G protein-coupled receptor associated sorting protein 1 (Gprasp1), transcript variant 3 | -1.79 |
| Zfp579 | zinc finger protein 579 (Zfp579) | -1.8 |
| Ppic | peptidylprolyl isomerase C (Ppic) | -1.81 |
| Hspb6 | heat shock protein, alpha-crystallin-related, B6 (Hspb6) | -1.81 |
| Dscr1l2 | Down syndrome critical region gene 1-like 2 (Dscr1l2) | -1.82 |
| Ttc3 | tetratricopeptide repeat domain 3 (Ttc3) | -1.82 |
| Ctsw | cathepsin W (Ctsw) | -1.83 |
| Lat | linker for activation of T cells (Lat) | -1.83 |
| Il18r1 | interleukin 18 receptor 1 (Il18r1) | -1.84 |
| Hmha1 | histocompatibility (minor) HA-1 (Hmha1) | -1.84 |
| Mib2 | mindbomb homolog 2 (Drosophila) (Mib2) | -1.84 |
| Cd27 | CD27 antigen (Cd27), transcript variant 1 | -1.86 |
| Gimap5 | GTPase, IMAP family member 5 (Gimap5) | -1.86 |
| Meis2 | Meis homeobox 2 (Meis2) | -1.86 |
| Nfix | nuclear factor I/X (Nfix), transcript variant 2 | -1.87 |
| Centb5 | centaurin, beta 5 (Centb5) | -1.88 |
| 1810020D17Rik | RIKEN cDNA 1810020D17 gene (1810020D17Rik) | -1.88 |
| 0610007C21Rik | RIKEN cDNA 0610007C21 gene (0610007C21Rik), transcript variant 2 | -1.88 |
| Arhgef18 | rho/rac guanine nucleotide exchange factor (GEF) 18 (Arhgef18) | -1.88 |
| Eif4el3 | eukaryotic translation initiation factor 4E member 2 (Eif4el3) | -1.89 |
| Selplg | selectin, platelet (p-selectin) ligand (Selplg) | -1.9 |
| Gltp | glycolipid transfer protein (Gltp) | -1.91 |
| Chka | choline kinase alpha (Chka), transcript variant 2 | -1.91 |
| Slc9a3r2 | solute carrier family 9 (sodium/hydrogen exchanger), member 3 regulator 2 (Slc9a3r2), transcript variant A | -1.91 |
| Lck | lymphocyte protein tyrosine kinase (Lck) | -1.91 |
| Dusp1 | dual specificity phosphatase 1 (Dusp1) | -1.92 |
| Grasp | GRP1 (general receptor for phosphoinositides 1)-associated scaffold protein (Grasp) | -1.92 |
| Cyp1b1 | cytochrome P450, family 1, subfamily b, polypeptide 1 (Cyp1b1) | -1.93 |
| Cish | cytokine inducible SH2-containing protein (Cish) | -1.94 |
| H2-M3 | histocompatibility 2, M region locus 3 (H2-M3) | -1.94 |
| Trib2 | tribbles homolog 2 (Trib2) | -1.95 |
| Psen2 | presenilin 2 (Psen2) | -1.95 |
| Trf | transferrin (Trf) | -1.95 |
| Cd8b1 | CD8 antigen, beta chain 1 (Cd8b1) | -1.96 |
| Ctsa | cathepsin A (Ctsa), transcript variant 2 | -1.96 |
| Plrg1 | pleiotropic regulator 1, PRL1 homolog (Arabidopsis) (Plrg1) | -1.97 |
| 2610207I05Rik | RIKEN cDNA 2610207I05 gene (2610207I05Rik) | -1.97 |
| Ptprb | protein tyrosine phosphatase, receptor type, B (Ptprb) | -1.97 |
| Ppox | protoporphyrinogen oxidase (Ppox), nuclear gene encoding mitochondrial protein | -1.97 |
| Nt5e | 5' nucleotidase, ecto (Nt5e) | -1.98 |
| Pik3r1 | phosphatidylinositol 3-kinase, regulatory subunit, polypeptide 1 (p85 alpha) (Pik3r1), transcript variant 2 | -1.98 |
| Spsb3 | splA/ryanodine receptor domain and SOCS box containing 3 (Spsb3) | -1.98 |
| Il6st | interleukin 6 signal transducer (Il6st) | -2 |
| Hdac7 | histone deacetylase 7 (Hdac7) | -2 |
| Gtf2h1 | general transcription factor II H, polypeptide 1 (Gtf2h1) | -2.01 |
| Prkcbp1 | protein kinase C binding protein 1 (Prkcbp1) | -2.02 |
| D230007K08Rik | RIKEN cDNA D230007K08 gene, transcript variant 5 (D230007K08Rik) | -2.03 |
| Fntb | farnesyltransferase, CAAX box, beta (Fntb) | -2.05 |
| Mmp2 | matrix metallopeptidase 2 (Mmp2) | -2.05 |
| Mrpl9 | mitochondrial ribosomal protein L9 (Mrpl9), nuclear gene encoding mitochondrial protein | -2.06 |
| Nupr1 | nuclear protein 1 (Nupr1) | -2.07 |
| Ldb2 | LIM domain binding 2 (Ldb2) | -2.07 |
| Ramp1 | receptor (calcitonin) activity modifying protein 1 (Ramp1) | -2.07 |
| Mrpl48 | mitochondrial ribosomal protein L48 (Mrpl48), transcript variant 1 | -2.07 |
| Ssbp3 | single-stranded DNA binding protein 3 (Ssbp3), transcript variant 1 | -2.07 |
| Tgfbr3 | transforming growth factor, beta receptor III (Tgfbr3) | -2.08 |
| 1810073G14Rik | RIKEN cDNA 1810073G14 gene (1810073G14Rik) | -2.08 |
| 4933439C20Rik | RIKEN cDNA 4933439C20 gene (4933439C20Rik) | -2.1 |
| Cyp2d22 | cytochrome P450, family 2, subfamily d, polypeptide 22 (Cyp2d22) | -2.13 |
| Dcp1b | DCP1 decapping enzyme homolog b (S. cerevisiae) (Dcp1b) | -2.15 |
| Zap70 | zeta-chain (TCR) associated protein kinase (Zap70) | -2.15 |
| Dgka | diacylglycerol kinase, alpha (Dgka) | -2.15 |
| Bok | BCL2-related ovarian killer protein (Bok) | -2.15 |
| Timp2 | tissue inhibitor of metalloproteinase 2 (Timp2) | -2.16 |
| 2310039H08Rik | RIKEN cDNA 2310039H08 gene (2310039H08Rik) | -2.17 |
| Cd8b1 | CD8 antigen, beta chain 1 (Cd8b1) | -2.17 |
| Btla | B and T lymphocyte associated (Btla), transcript variant 2 | -2.18 |
| Tcf25 | transcription factor 25 (basic helix-loop-helix) (Tcf25), transcript variant 3 | -2.2 |
| Ephx1 | epoxide hydrolase 1, microsomal (Ephx1) | -2.21 |
| Tmem68 | transmembrane protein 68 (Tmem68) | -2.21 |
| Gsto1 | glutathione S-transferase omega 1 (Gsto1) | -2.23 |
| Fxyd5 | FXYD domain-containing ion transport regulator 5 (Fxyd5) | -2.24 |
| Rcan3 | regulator of calcineurin 3 (Rcan3) | -2.25 |
| Itk | IL2-inducible T-cell kinase (Itk) | -2.28 |
| Igfbp4 | insulin-like growth factor binding protein 4 (Igfbp4) | -2.31 |
| Pdk1 | pyruvate dehydrogenase kinase, isoenzyme 1 (Pdk1), nuclear gene encoding mitochondrial protein | -2.32 |
| Fcgrt | Fc receptor, IgG, alpha chain transporter (Fcgrt) | -2.33 |
| Ptpn22 | protein tyrosine phosphatase, non-receptor type 22 (lymphoid) (Ptpn22) | -2.36 |
| Mgst1 | microsomal glutathione S-transferase 1 (Mgst1) | -2.36 |
| 6030443O07Rik | RIKEN cDNA 6030443O07 gene (6030443O07Rik) | -2.36 |
| Hcst | hematopoietic cell signal transducer (Hcst) | -2.37 |
| Lrig1 | leucine-rich repeats and immunoglobulin-like domains 1 (Lrig1) | -2.37 |
| Klk8 | kallikrein related-peptidase 8 (Klk8) | -2.41 |
| Nrp1 | neuropilin 1 (Nrp1) | -2.42 |
| Mycl1 | v-myc myelocytomatosis viral oncogene homolog 1, lung carcinoma derived (avian) (Mycl1) | -2.42 |
| Lip1 | lysosomal acid lipase A (Lip1) | -2.44 |
| Sesn1 | sestrin 1 (Sesn1) | -2.45 |
| Cecr5 | cat eye syndrome chromosome region, candidate 5 homolog (human) (Cecr5) | -2.45 |
| Gabarapl1 | gamma-aminobutyric acid (GABA(A)) receptor-associated protein-like 1 (Gabarapl1) | -2.46 |
| Sidt2 | SID1 transmembrane family, member 2 (Sidt2) | -2.5 |
| Dhrs7 | dehydrogenase/reductase (SDR family) member 7 (Dhrs7) | -2.51 |
| Abhd14b | abhydrolase domain containing 14b (Abhd14b) | -2.52 |
| Snrk | SNF related kinase (Snrk) | -2.53 |
| Sidt2 | SID1 transmembrane family, member 2 (Sidt2) | -2.54 |
| Gnptg | N-acetylglucosamine-1-phosphotransferase, gamma subunit (Gnptg) | -2.55 |
| Nrp1 | neuropilin 1 (Nrp1) | -2.56 |
| Scarf2 | scavenger receptor class F, member 2 (Scarf2) | -2.57 |
| Timp3 | tissue inhibitor of metalloproteinase 3 (Timp3) | -2.59 |
| Hmgcs2 | 3-hydroxy-3-methylglutaryl-Coenzyme A synthase 2 (Hmgcs2), nuclear gene encoding mitochondrial protein | -2.6 |
| AI450540 | expressed sequence AI450540 (AI450540) | -2.6 |
| Ihpk1 | inositol hexaphosphate kinase 1 (Ihpk1) | -2.6 |
| Dgka | diacylglycerol kinase, alpha (Dgka) | -2.6 |
| Dnajc15 | DnaJ (Hsp40) homolog, subfamily C, member 15 (Dnajc15) | -2.61 |
| X99384 | cDNA sequence X99384 (X99384) | -2.63 |
| Slc25a20 | solute carrier family 25 (mitochondrial carnitine/acylcarnitine translocase), member 20 (Slc25a20) | -2.66 |
| 4930432O21Rik | RIKEN cDNA 4930432O21 gene (4930432O21Rik) | -2.66 |
| Cxcl12 | chemokine (C-X-C motif) ligand 12 (Cxcl12) | -2.67 |
| Appl2 | adaptor protein, phosphotyrosine interaction, PH domain and leucine zipper containing 2 (Appl2) | -2.69 |
| Trim56 | tripartite motif-containing 56 (Trim56) | -2.71 |
| Sin3a | transcriptional regulator, SIN3A (yeast) (Sin3a) | -2.72 |
| Stard8 | START domain containing 8 (Stard8) | -2.72 |
| Dctn6 | dynactin 6 (Dctn6) | -2.72 |
| Sepp1 | selenoprotein P, plasma, 1 (Sepp1), transcript variant 2 | -2.73 |
| LOC545056 | ubiquitin-conjugating enzyme E2, J2 homolog pseudogene (LOC545056) on chromosome 14. | -2.75 |
| Psg23 | pregnancy-specific glycoprotein 23 (Psg23) | -2.76 |
| Zap70 | zeta-chain (TCR) associated protein kinase (Zap70) | -2.76 |
| Cd3e | CD3 antigen, epsilon polypeptide (Cd3e) | -2.8 |
| Gnptg | N-acetylglucosamine-1-phosphotransferase, gamma subunit (Gnptg) | -2.85 |
| Rab27a | RAB27A, member RAS oncogene family (Rab27a) | -2.86 |
| Gpr114 | G protein-coupled receptor 114 (Gpr114) | -2.86 |
| Igfbp4 | insulin-like growth factor binding protein 4 (Igfbp4) | -2.87 |
| H6pd | hexose-6-phosphate dehydrogenase (glucose 1-dehydrogenase) (H6pd) | -2.88 |
| Vat1 | vesicle amine transport protein 1 homolog (T californica) (Vat1) | -2.88 |
| Acot1 | acyl-CoA thioesterase 1 (Acot1) | -2.89 |
| Dcn | decorin (Dcn) | -2.89 |
| Dcn | decorin (Dcn) | -2.91 |
| Stard8 | START domain containing 8 (Stard8) | -2.92 |
| Mgp | matrix Gla protein (Mgp) | -2.98 |
| Cyp27a1 | cytochrome P450, family 27, subfamily a, polypeptide 1 (Cyp27a1) | -3 |
| Tsc2 | tuberous sclerosis 2 (Tsc2), transcript variant 2 | -3.05 |
| Robo4 | roundabout homolog 4 (Drosophila) (Robo4) | -3.08 |
| Dpagt1 | dolichyl-phosphate (UDP-N-acetylglucosamine) acetylglucosaminephosphotransferase 1 (GlcNAc-1-P transferase) (Dpagt1) | -3.08 |
| Pkm2 | pyruvate kinase, muscle (Pkm2) | -3.08 |
| Cyp27a1 | cytochrome P450, family 27, subfamily a, polypeptide 1 (Cyp27a1) | -3.13 |
| Cd3d | CD3 antigen, delta polypeptide (Cd3d) | -3.13 |
| AI467606 | expressed sequence AI467606 (AI467606) | -3.13 |
| Tpcn1 | two pore channel 1 (Tpcn1) | -3.26 |
| Ankrd47 | ankyrin repeat domain 47 (Ankrd47) | -3.27 |
| Atp1b1 | ATPase, Na+/K+ transporting, beta 1 polypeptide (Atp1b1) | -3.28 |
| Sult1a1 | sulfotransferase family 1A, phenol-preferring, member 1 (Sult1a1) | -3.31 |
| Cldn5 | claudin 5 (Cldn5) | -3.32 |
| Cd3g | CD3 antigen, gamma polypeptide (Cd3g) | -3.33 |
| Ltbp4 | latent transforming growth factor beta binding protein 4 (Ltbp4) | -3.34 |
| Cdkn1c | cyclin-dependent kinase inhibitor 1C (P57) (Cdkn1c) | -3.35 |
| Dusp7 | dual specificity phosphatase 7 (Dusp7) | -3.38 |
| Hs3st1 | heparan sulfate (glucosamine) 3-O-sulfotransferase 1 (Hs3st1) | -3.4 |
| EG434197 | predicted gene, EG434197 (EG434197) | -3.47 |
| Ltbp4 | latent transforming growth factor beta binding protein 4 (Ltbp4) | -3.5 |
| Bcl9l | B cell CLL/lymphoma 9-like (Bcl9l) | -3.5 |
| Per2 | period homolog 2 (Drosophila) (Per2) | -3.56 |
| Asah3l | N-acylsphingosine amidohydrolase 3-like (Asah3l) | -3.59 |
| Sdpr | serum deprivation response (Sdpr) | -3.64 |
| Tle1 | transducin-like enhancer of split 1, homolog of Drosophila E(spl) (Tle1) | -3.71 |
| Hdc | histidine decarboxylase (Hdc) | -3.75 |
| Mt1 | metallothionein 1 (Mt1) | -3.91 |
| Ctgf | connective tissue growth factor (Ctgf) | -3.98 |
| Acpl2 | acid phosphatase-like 2 (Acpl2) | -4.02 |
| Fcna | ficolin A (Fcna) | -4.1 |
| Txnip | thioredoxin interacting protein (Txnip), transcript variant 1 | -4.14 |
| Fmo1 | flavin containing monooxygenase 1 (Fmo1) | -4.15 |
| Tef | thyrotroph embryonic factor (Tef), transcript variant 1 | -4.16 |
| Il7r | interleukin 7 receptor (Il7r) | -4.17 |
| Enpp5 | ectonucleotide pyrophosphatase/phosphodiesterase 5 (Enpp5) | -4.18 |
| Dkk3 | dickkopf homolog 3 (Xenopus laevis) (Dkk3) | -4.23 |
| Galnt10 | UDP-N-acetyl-alpha-D-galactosamine:polypeptide N-acetylgalactosaminyltransferase 10 (Galnt10) | -4.27 |
| Reln | reelin (Reln) | -4.3 |
| Tnfrsf4 | tumor necrosis factor receptor superfamily, member 4 (Tnfrsf4) | -4.3 |
| Bbc3 | Bcl-2 binding component 3 (Bbc3) | -4.32 |
| S100a9 | S100 calcium binding protein A9 (calgranulin B) (S100a9) | -4.4 |
| Lgals4 | lectin, galactose binding, soluble 4 (Lgals4) | -4.42 |
| LOC100047214 | similar to PTEN induced putative kinase 1 (LOC100047214) | -4.45 |
| Igf2 | insulin-like growth factor 2 (Igf2) | -4.46 |
| Slco2b1 | solute carrier organic anion transporter family, member 2b1 (Slco2b1) | -4.54 |
| Rps3a | ribosomal protein S3a (Rps3a) | -4.62 |
| Tsc22d3 | TSC22 domain family 3 (Tsc22d3), transcript variant 1 | -4.75 |
| Tmem66 | transmembrane protein 66 (Tmem66) | -5 |
| Tspan3 | tetraspanin 3 (Tspan3) | -5.06 |
| Bsdc1 | BSD domain containing 1 (Bsdc1) | -5.09 |
| Prg2 | proteoglycan 2, bone marrow (Prg2) | -5.1 |
| Dkk3 | dickkopf homolog 3 (Xenopus laevis) (Dkk3) | -5.1 |
| Klf9 | Kruppel-like factor 9 (Klf9) | -5.21 |
| Angptl4 | angiopoietin-like 4 (Angptl4) | -5.53 |
| Gpx3 | glutathione peroxidase 3 (Gpx3), transcript variant 2 | -5.67 |
| LOC100044862 | similar to Fbxl3 protein (LOC100044862) | -5.91 |
| 6430706D22Rik | RIKEN cDNA 6430706D22 gene (6430706D22Rik) | -6.05 |
| Per1 | period homolog 1 (Drosophila) (Per1) | -6.09 |
| Dbp | D site albumin promoter binding protein (Dbp) | -7.24 |
| Hba-a1 | hemoglobin alpha, adult chain 1 (Hba-a1) | -7.67 |
| Ddit4 | DNA-damage-inducible transcript 4 (Ddit4) | -7.77 |
| Mgst2 | microsomal glutathione S-transferase 2 (Mgst2) | -8.26 |
| Cyp2f2 | cytochrome P450, family 2, subfamily f, polypeptide 2 (Cyp2f2) | -11.16 |
| Inmt | indolethylamine N-methyltransferase (Inmt) | -12.71 |
| Emb | embigin (Emb) | -14.52 |
