# Supplementary material for: Genomic deletion of GIT2 induces a premature age-related thymic dysfunction and systemic immune system disruption
Source: Aging (Albany NY). 2017 Mar 4;9(3):706–30. doi: 10.18632/aging.101185 (PMC5391227; doi:10.18632/aging.101185)
Supplement: Supplementary file 12 [file aging-09-706-s012.docx]

**Table S11. Transcripts significantly regulated differentially between the GIT2KO mesenteric lymph node (MLN) and WT MLN.** The official Gene Symbol, transcript description and associated Z ratios for the comparison of the GIT2KO MLN *vs.* WT MLN at the 12 month time point are indicated. Each transcript was significantly regulated at p<0.05.

| **Symbol** | **Description** | **Z ratio** |
| --- | --- | --- |
| Ctse | cathepsin E (Ctse) | 13.47 |
| H2-T10 | histocompatibility 2, T region locus 10 (H2-T10) | 8.5 |
| Pik3cg | phosphoinositide-3-kinase, catalytic, gamma polypeptide (Pik3cg) | 6.48 |
| St6galnac2 | ST6 (alpha-N-acetyl-neuraminyl-2,3-beta-galactosyl-1, 3)-N-acetylgalactosaminide alpha-2,6-sialyltransferase 2 (St6galnac2) | 6.28 |
| Tmem66 | transmembrane protein 66 (Tmem66) | 5.97 |
| Kras | v-Ki-ras2 Kirsten rat sarcoma viral oncogene homolog (Kras) | 5.88 |
| Emr1 | EGF-like module containing, mucin-like, hormone receptor-like sequence 1 (Emr1) | 5.64 |
| Adi1 | acireductone dioxygenase 1 (Adi1) | 4.99 |
| Glo1 | glyoxalase 1 (Glo1) | 4.91 |
| Cxcl9 | chemokine (C-X-C motif) ligand 9 (Cxcl9) | 4.85 |
| LOC100043671 | hypothetical protein LOC100043671 (LOC100043671) | 4.77 |
| Hp | haptoglobin (Hp) | 4.35 |
| Scd1 | stearoyl-Coenzyme A desaturase 1 (Scd1) | 4.08 |
| Ndufb10 | NADH dehydrogenase (ubiquinone) 1 beta subcomplex, 10 (Ndufb10) | 4.08 |
| Cap1 | CAP, adenylate cyclase-associated protein 1 (yeast) (Cap1) | 3.93 |
| Cyp1b1 | cytochrome P450, family 1, subfamily b, polypeptide 1 (Cyp1b1) | 3.78 |
| Hpgd | hydroxyprostaglandin dehydrogenase 15 (NAD) (Hpgd) | 3.76 |
| Hp | haptoglobin (Hp) | 3.73 |
| Rb1 | retinoblastoma 1 (Rb1) | 3.73 |
| Ndn | necdin (Ndn) | 3.5 |
| Ccl19 | chemokine (C-C motif) ligand 19 (Ccl19) | 3.48 |
| Srgn | serglycin (Srgn) | 3.44 |
| Igtp | interferon gamma induced GTPase (Igtp) | 3.38 |
| Serpina3f | serine (or cysteine) peptidase inhibitor, clade A, member 3F (Serpina3f) | 3.33 |
| Ubd | ubiquitin D (Ubd) | 3.33 |
| Sc4mol | sterol-C4-methyl oxidase-like (Sc4mol) | 3.26 |
| Flrt3 | fibronectin leucine rich transmembrane protein 3 (Flrt3) | 3.23 |
| Gbp3 | guanylate nucleotide binding protein 3 (Gbp3) | 3.23 |
| Gfer | growth factor, erv1 (S. cerevisiae)-like (augmenter of liver regeneration) (Gfer) | 3.23 |
| Id2 | inhibitor of DNA binding 2 (Id2) | 3.01 |
| Sqle | squalene epoxidase (Sqle) | 3 |
| Hp | haptoglobin (Hp) | 3 |
| Txn1 | thioredoxin 1 (Txn1) | 2.99 |
| Il18r1 | interleukin 18 receptor 1 (Il18r1) | 2.95 |
| Prg2 | proteoglycan 2, bone marrow (Prg2) | 2.93 |
| Cp | ceruloplasmin (Cp), transcript variant 2 | 2.92 |
| Cd55 | CD55 antigen (Cd55) | 2.86 |
| Atp5a1 | ATP synthase, H+ transporting, mitochondrial F1 complex, alpha subunit, isoform 1 (Atp5a1), nuclear gene encoding mitochondrial protein | 2.85 |
| Rnase4 | ribonuclease, RNase A family 4 (Rnase4), transcript variant 1 | 2.77 |
| S100a10 | S100 calcium binding protein A10 (calpactin) (S100a10) | 2.76 |
| Fgd2 | FYVE, RhoGEF and PH domain containing 2 (Fgd2) | 2.7 |
| Slain2 | SLAIN motif family, member 2 (Slain2) | 2.7 |
| Ptges3 | prostaglandin E synthase 3 (cytosolic) (Ptges3) | 2.62 |
| Zfp238 | zinc finger protein 238 (Zfp238), transcript variant 2 | 2.6 |
| Sepp1 | selenoprotein P, plasma, 1 (Sepp1), transcript variant 2 | 2.59 |
| Ibtk | inhibitor of Bruton agammaglobulinemia tyrosine kinase (Ibtk) | 2.57 |
| 1110002B05Rik | RIKEN cDNA 1110002B05 gene (1110002B05Rik) | 2.57 |
| 2700055A20Rik | membrane-associated ring finger (C3HC4) 5 (March5) | 2.55 |
| LOC100048721 | similar to fibronectin leucine rich transmembrane protein 3, transcript variant 1 (LOC100048721) | 2.54 |
| Cyb5 | cytochrome b-5 (Cyb5) | 2.53 |
| Cdr2 | cerebellar degeneration-related 2 (Cdr2) | 2.53 |
| Furin | furin (paired basic amino acid cleaving enzyme) (Furin) | 2.5 |
| Cox6c | cytochrome c oxidase, subunit VIc (Cox6c) | 2.48 |
| Igsf4a | cell adhesion molecule 1 (Cadm1) | 2.47 |
| Col18a1 | procollagen, type XVIII, alpha 1 (Col18a1) | 2.45 |
| Rnf11 | ring finger protein 11 (Rnf11) | 2.45 |
| Gbp3 | guanylate nucleotide binding protein 3 (Gbp3) | 2.43 |
| 9130422G05Rik | tetratricopeptide repeat domain 39B (Ttc39b) | 2.4 |
| Prdx5 | peroxiredoxin 5 (Prdx5), nuclear gene encoding mitochondrial protein | 2.39 |
| P2ry5 | purinergic receptor P2Y, G-protein coupled, 5 (P2ry5) | 2.39 |
| Ndufs4 | NADH dehydrogenase (ubiquinone) Fe-S protein 4 (Ndufs4), nuclear gene encoding mitochondrial protein | 2.39 |
| Chchd7 | coiled-coil-helix-coiled-coil-helix domain containing 7 (Chchd7) | 2.36 |
| Pdgfa | platelet derived growth factor, alpha (Pdgfa) | 2.35 |
| Nab1 | Ngfi-A binding protein 1 (Nab1) | 2.32 |
| Ifi47 | interferon gamma inducible protein 47 (Ifi47) | 2.3 |
| Itm2b | integral membrane protein 2B (Itm2b) | 2.3 |
| LOC100047963 | similar to ADIR1 (LOC100047963) | 2.29 |
| Cd84 | CD84 antigen (Cd84) | 2.29 |
| Loh11cr2a | loss of heterozygosity, 11, chromosomal region 2, gene A homolog (human) (Loh11cr2a) | 2.28 |
| Pcyox1 | prenylcysteine oxidase 1 (Pcyox1) | 2.27 |
| Lhfp | lipoma HMGIC fusion partner (Lhfp) | 2.25 |
| Vapa | vesicle-associated membrane protein, associated protein A (Vapa) | 2.24 |
| Hnrpdl | heterogeneous nuclear ribonucleoprotein D-like (Hnrpdl) | 2.23 |
| Sec11c | SEC11 homolog C (S. cerevisiae) (Sec11c) | 2.22 |
| Hsp90b1 | heat shock protein 90, beta (Grp94), member 1 (Hsp90b1) | 2.21 |
| Dok2 | docking protein 2 (Dok2) | 2.2 |
| Atp6v1a | ATPase, H+ transporting, lysosomal V1 subunit A (Atp6v1a) | 2.2 |
| Hadhb | hydroxyacyl-Coenzyme A dehydrogenase/3-ketoacyl-Coenzyme A thiolase/enoyl-Coenzyme A hydratase (trifunctional protein), beta subunit (Hadhb) | 2.2 |
| Ndufs4 | NADH dehydrogenase (ubiquinone) Fe-S protein 4 (Ndufs4), nuclear gene encoding mitochondrial protein | 2.15 |
| LOC100048480 | similar to ubiquinol-cytochrome c reductase binding protein (LOC100048480) | 2.15 |
| 2610204L23Rik | coiled-coil domain containing 47 (Ccdc47) | 2.12 |
| Hebp1 | heme binding protein 1 (Hebp1) | 2.11 |
| Rbp1 | retinol binding protein 1, cellular (Rbp1) | 2.11 |
| Idh2 | isocitrate dehydrogenase 2 (NADP+), mitochondrial (Idh2), nuclear gene encoding mitochondrial protein | 2.09 |
| Agtrap | angiotensin II, type I receptor-associated protein (Agtrap) | 2.09 |
| Atp10d | ATPase, Class V, type 10D (Atp10d) | 2.08 |
| Eif4e3 | eukaryotic translation initiation factor 4E member 3 (Eif4e3) | 2.07 |
| P2ry6 | pyrimidinergic receptor P2Y, G-protein coupled, 6 (P2ry6) | 2.03 |
| Pmp22 | peripheral myelin protein (Pmp22) | 2.03 |
| Slc38a2 | solute carrier family 38, member 2 (Slc38a2) | 2.03 |
| Hrbl | HIV-1 Rev binding protein-like (Hrbl), transcript variant 2 | 2.02 |
| Emp1 | epithelial membrane protein 1 (Emp1) | 2.02 |
| LOC547343 | similar to H-2 class I histocompatibility antigen, L-D alpha chain precursor (LOC547343) | 2.01 |
| Stard4 | StAR-related lipid transfer (START) domain containing 4 (Stard4) | 2.01 |
| Myo6 | myosin VI (Myo6) | 2 |
| Birc2 | baculoviral IAP repeat-containing 2 (Birc2) | 1.98 |
| Lrg1 | leucine-rich alpha-2-glycoprotein 1 (Lrg1) | 1.98 |
| Lip1 | lysosomal acid lipase A (Lip1) | 1.97 |
| Gng10 | guanine nucleotide binding protein (G protein), gamma 10 (Gng10) | 1.97 |
| Hspe1 | heat shock protein 1 (chaperonin 10) (Hspe1) | 1.97 |
| Vegfa | vascular endothelial growth factor A (Vegfa), transcript variant 2 | 1.96 |
| Rbms3 | RNA binding motif, single stranded interacting protein (Rbms3) | 1.94 |
| Lat | linker for activation of T cells (Lat) | 1.94 |
| Tmem33 | transmembrane protein 33 (Tmem33), transcript variant 1 | 1.94 |
| 1200002N14Rik | RIKEN cDNA 1200002N14 gene (1200002N14Rik) | 1.94 |
| Lgals1 | lectin, galactose binding, soluble 1 (Lgals1) | 1.92 |
| Vti1b | vesicle transport through interaction with t-SNAREs 1B homolog (Vti1b) | 1.91 |
| Sepx1 | selenoprotein X 1 (Sepx1) | 1.9 |
| Csf1r | colony stimulating factor 1 receptor (Csf1r) | 1.89 |
| Rbm47 | RNA binding motif protein 47 (Rbm47) | 1.89 |
| Picalm | phosphatidylinositol binding clathrin assembly protein (Picalm) | 1.87 |
| Nuak1 | NUAK family, SNF1-like kinase, 1 (Nuak1) | 1.86 |
| Pja2 | praja 2, RING-H2 motif containing (Pja2), transcript variant 2 | 1.85 |
| Cnot7 | CCR4-NOT transcription complex, subunit 7 (Cnot7) | 1.84 |
| Fes | feline sarcoma oncogene (Fes) | 1.84 |
| Eed | embryonic ectoderm development (Eed) | 1.84 |
| Slc2a1 | solute carrier family 2 (facilitated glucose transporter), member 1 (Slc2a1) | 1.83 |
| Prkra | protein kinase, interferon inducible double stranded RNA dependent activator (Prkra) | 1.83 |
| Ogt | O-linked N-acetylglucosamine (GlcNAc) transferase (UDP-N-acetylglucosamine:polypeptide-N-acetylglucosaminyl transferase) (Ogt) | 1.8 |
| Atp5h | ATP synthase, H+ transporting, mitochondrial F0 complex, subunit d (Atp5h), nuclear gene encoding mitochondrial protein | 1.8 |
| Atg5 | autophagy-related 5 (yeast) (Atg5) | 1.8 |
| Pdpk1 | 3-phosphoinositide dependent protein kinase-1 (Pdpk1), transcript variant 2 | 1.79 |
| 3110050N22Rik | RIKEN cDNA 3110050N22 gene (3110050N22Rik) | 1.78 |
| Tmem55a | transmembrane protein 55A (Tmem55a) | 1.77 |
| Lman1 | lectin, mannose-binding, 1 (Lman1) | 1.77 |
| Ss18 | synovial sarcoma translocation, Chromosome 18 (Ss18) | 1.77 |
| Iigp2 | interferon inducible GTPase 2 (Iigp2) | 1.77 |
| Igfbp5 | insulin-like growth factor binding protein 5 (Igfbp5) | 1.77 |
| C1qc | complement component 1, q subcomponent, C chain (C1qc) | 1.75 |
| Evi2a | ecotropic viral integration site 2a (Evi2a), transcript variant 2 | 1.74 |
| Cugbp2 | CUG triplet repeat, RNA binding protein 2 (Cugbp2), transcript variant 6 | 1.73 |
| 1810035L17Rik | RIKEN cDNA 1810035L17 gene (1810035L17Rik) | 1.72 |
| Vars2 | valyl-tRNA synthetase 2, mitochondrial (putative) (Vars2) | 1.72 |
| LOC100044177 | hypothetical protein LOC100044177 (LOC100044177) | 1.71 |
| Dusp19 | dual specificity phosphatase 19 (Dusp19) | 1.71 |
| Lgals8 | lectin, galactose binding, soluble 8 (Lgals8) | 1.71 |
| Acadsb | acyl-Coenzyme A dehydrogenase, short/branched chain (Acadsb), nuclear gene encoding mitochondrial protein | 1.71 |
| Vdac2 | voltage-dependent anion channel 2 (Vdac2) | 1.71 |
| Snap29 | synaptosomal-associated protein 29 (Snap29) | 1.7 |
| Fez2 | fasciculation and elongation protein zeta 2 (zygin II) (Fez2) | 1.7 |
| 4930572J05Rik | RIKEN cDNA 4930572J05 gene (4930572J05Rik) | 1.7 |
| Rnf11 | ring finger protein 11 (Rnf11) | 1.7 |
| Chrnb1 | cholinergic receptor, nicotinic, beta polypeptide 1 (muscle) (Chrnb1) | 1.69 |
| Cyp4v3 | cytochrome P450, family 4, subfamily v, polypeptide 3 (Cyp4v3) | 1.69 |
| Adam17 | a disintegrin and metallopeptidase domain 17 (Adam17) | 1.69 |
| Cd2bp2 | CD2 antigen (cytoplasmic tail) binding protein 2 (Cd2bp2) | 1.68 |
| Gosr2 | golgi SNAP receptor complex member 2 (Gosr2) | 1.68 |
| Il33 | interleukin 33 (Il33) | 1.67 |
| Btbd14a | BTB (POZ) domain containing 14A (Btbd14a), transcript variant 2 | 1.66 |
| Selk | selenoprotein K (Selk) | 1.66 |
| Slc25a17 | solute carrier family 25 (mitochondrial carrier, peroxisomal membrane protein), member 17 (Slc25a17), nuclear gene encoding mitochondrial protein | 1.66 |
| D9Wsu20e | transmembrane protein 30A (Tmem30a) | 1.66 |
| Etfa | electron transferring flavoprotein, alpha polypeptide (Etfa), nuclear gene encoding mitochondrial protein | 1.66 |
| Ebpl | emopamil binding protein-like (Ebpl) | 1.66 |
| Phlda1 | pleckstrin homology-like domain, family A, member 1 (Phlda1) | 1.65 |
| Pigp | phosphatidylinositol glycan anchor biosynthesis, class P (Pigp) | 1.65 |
| Ube3c | ubiquitin protein ligase E3C (Ube3c) | 1.64 |
| Rnasek | ribonuclease, RNase K (Rnasek) | 1.64 |
| Idh1 | isocitrate dehydrogenase 1 (NADP+), soluble (Idh1) | 1.64 |
| 3110009E18Rik | RIKEN cDNA 3110009E18 gene (3110009E18Rik) | 1.63 |
| Tax1bp1 | Tax1 (human T-cell leukemia virus type I) binding protein 1 (Tax1bp1) | 1.63 |
| Il16 | interleukin 16 (Il16) | 1.63 |
| Zdhhc21 | zinc finger, DHHC domain containing 21 (Zdhhc21) | 1.63 |
| 2310056P07Rik | RIKEN cDNA 2310056P07 gene (2310056P07Rik) | 1.62 |
| Atxn1 | ataxin 1 (Atxn1) | 1.61 |
| Osbpl2 | oxysterol binding protein-like 2 (Osbpl2) | 1.61 |
| Ak3 | adenylate kinase 3 (Ak3) | 1.61 |
| Ddx3x | DEAD/H (Asp-Glu-Ala-Asp/His) box polypeptide 3, X-linked (Ddx3x) | 1.61 |
| Nhlrc2 | NHL repeat containing 2 (Nhlrc2) | 1.6 |
| Ndufs4 | NADH dehydrogenase (ubiquinone) Fe-S protein 4 (Ndufs4), nuclear gene encoding mitochondrial protein | 1.6 |
| Npc2 | Niemann Pick type C2 (Npc2) | 1.6 |
| LOC100040592 | similar to Hmgcs1 protein, transcript variant 1 (LOC100040592) | 1.58 |
| Tgfbr1 | transforming growth factor, beta receptor I (Tgfbr1) | 1.57 |
| LOC100046775 | similar to CMP-sialic acid transporter (LOC100046775) | 1.56 |
| Unc45a | unc-45 homolog A (C. elegans) (Unc45a) | 1.56 |
| Nfib | nuclear factor I/B (Nfib) | 1.56 |
| Supt4h1 | suppressor of Ty 4 homolog 1 (S. cerevisiae) (Supt4h1) | 1.56 |
| Dcun1d5 | DCN1, defective in cullin neddylation 1, domain containing 5 (S. cerevisiae) (Dcun1d5) | 1.56 |
| Slc25a1 | solute carrier family 25 (mitochondrial carrier, citrate transporter), member 1 (Slc25a1), nuclear gene encoding mitochondrial protein | 1.55 |
| Eno1 | enolase 1, alpha non-neuron (Eno1) | 1.54 |
| Nck1 | non-catalytic region of tyrosine kinase adaptor protein 1 (Nck1) | 1.54 |
| Ankrd10 | ankyrin repeat domain 10 (Ankrd10) | 1.54 |
| Pscd3 | pleckstrin homology, Sec7 and coiled-coil domains 3 (Pscd3) | 1.54 |
| Rbbp9 | retinoblastoma binding protein 9 (Rbbp9) | 1.53 |
| Fnbp1l | formin binding protein 1-like (Fnbp1l) | 1.52 |
| Cnpy2 | canopy 2 homolog (zebrafish) (Cnpy2) | 1.52 |
| Smek2 | SMEK homolog 2, suppressor of mek1 (Dictyostelium) (Smek2) | 1.51 |
| Epc1 | enhancer of polycomb homolog 1 (Drosophila) (Epc1), transcript variant 1 | -1.5 |
| LOC100044829 | similar to Fibrillarin, transcript variant 1 (LOC100044829) | -1.5 |
| Setd1a | SET domain containing 1A (Setd1a) | -1.51 |
| Actr1a | ARP1 actin-related protein 1 homolog A (yeast) (Actr1a) | -1.52 |
| Phkg2 | phosphorylase kinase, gamma 2 (testis) (Phkg2) | -1.53 |
| Bax | Bcl2-associated X protein (Bax) | -1.54 |
| Cnot4 | CCR4-NOT transcription complex, subunit 4 (Cnot4) | -1.54 |
| Gnptg | N-acetylglucosamine-1-phosphotransferase, gamma subunit (Gnptg) | -1.55 |
| 4930432O21Rik | RIKEN cDNA 4930432O21 gene (4930432O21Rik) | -1.55 |
| Ebi2 | G protein-coupled receptor 183 (Gpr183) | -1.56 |
| Rab27a | RAB27A, member RAS oncogene family (Rab27a) | -1.57 |
| Ssbp3 | single-stranded DNA binding protein 3 (Ssbp3), transcript variant 1 | -1.58 |
| Gart | phosphoribosylglycinamide formyltransferase (Gart) | -1.6 |
| 5430437P03Rik | RIKEN cDNA 5430437P03 gene (5430437P03Rik) | -1.6 |
| Per2 | period homolog 2 (Drosophila) (Per2) | -1.61 |
| Yeats4 | YEATS domain containing 4 (Yeats4) | -1.61 |
| Suz12 | suppressor of zeste 12 homolog (Drosophila) (Suz12) | -1.62 |
| Spsb3 | splA/ryanodine receptor domain and SOCS box containing 3 (Spsb3) | -1.62 |
| Gtf2h1 | general transcription factor II H, polypeptide 1 (Gtf2h1) | -1.62 |
| Sf3b2 | splicing factor 3b, subunit 2 (Sf3b2) | -1.63 |
| Mrpl48 | mitochondrial ribosomal protein L48 (Mrpl48), transcript variant 1 | -1.63 |
| Cd3e | CD3 antigen, epsilon polypeptide (Cd3e) | -1.63 |
| Hagh | hydroxyacyl glutathione hydrolase (Hagh) | -1.64 |
| Pip5k1a | phosphatidylinositol-4-phosphate 5-kinase, type 1 alpha (Pip5k1a) | -1.64 |
| Irs2 | insulin receptor substrate 2 (Irs2) | -1.64 |
| Psg23 | pregnancy-specific glycoprotein 23 (Psg23) | -1.65 |
| Mrps7 | mitchondrial ribosomal protein S7 (Mrps7) | -1.66 |
| Uba1 | ubiquitin-like modifier activating enzyme 1 (Uba1) | -1.66 |
| Zmynd11 | zinc finger, MYND domain containing 11 (Zmynd11) | -1.67 |
| H2-M3 | histocompatibility 2, M region locus 3 (H2-M3) | -1.67 |
| Tnrc6a | trinucleotide repeat containing 6a (Tnrc6a) | -1.69 |
| D4Ertd22e | DNA segment, Chr 4, ERATO Doi 22, expressed (D4Ertd22e), transcript variant 1 | -1.69 |
| Samsn1 | SAM domain, SH3 domain and nuclear localization signals, 1 (Samsn1) | -1.69 |
| Ogdh | oxoglutarate dehydrogenase (lipoamide) (Ogdh), nuclear gene encoding mitochondrial protein | -1.7 |
| Arhgef18 | rho/rac guanine nucleotide exchange factor (GEF) 18 (Arhgef18) | -1.7 |
| Sbds | Shwachman-Bodian-Diamond syndrome homolog (human) (Sbds) | -1.72 |
| Abcf1 | ATP-binding cassette, sub-family F (GCN20), member 1 (Abcf1) | -1.72 |
| Pafah1b3 | platelet-activating factor acetylhydrolase, isoform 1b, alpha1 subunit (Pafah1b3) | -1.73 |
| 2310036O22Rik | RIKEN cDNA 2310036O22 gene (2310036O22Rik) | -1.74 |
| Ugcg | UDP-glucose ceramide glucosyltransferase (Ugcg) | -1.75 |
| Trim56 | tripartite motif-containing 56 (Trim56) | -1.78 |
| AI450540 | expressed sequence AI450540 (AI450540) | -1.78 |
| Rbm38 | RNA binding motif protein 38 (Rbm38) | -1.79 |
| Rbm38 | RNA binding motif protein 38 (Rbm38) | -1.8 |
| Arpc5 | actin related protein 2/3 complex, subunit 5 (Arpc5) | -1.81 |
| Cd83 | CD83 antigen (Cd83) | -1.82 |
| Dync1h1 | dynein cytoplasmic 1 heavy chain 1 (Dync1h1) | -1.83 |
| Eif3i | eukaryotic translation initiation factor 3, subunit I (Eif3i) | -1.85 |
| Vars | valyl-tRNA synthetase (Vars) | -1.85 |
| 6430510M02Rik | RIKEN cDNA 6430510M02 gene (6430510M02Rik) | -1.87 |
| Nfkbia | nuclear factor of kappa light polypeptide gene enhancer in B-cells inhibitor, alpha (Nfkbia) | -1.87 |
| 2310039H08Rik | RIKEN cDNA 2310039H08 gene (2310039H08Rik) | -1.88 |
| Mrpl9 | mitochondrial ribosomal protein L9 (Mrpl9), nuclear gene encoding mitochondrial protein | -1.88 |
| Akna | AT-hook transcription factor (Akna) | -1.9 |
| Mrps34 | mitochondrial ribosomal protein S34 (Mrps34), nuclear gene encoding mitochondrial protein | -1.91 |
| Ppp3ca | protein phosphatase 3, catalytic subunit, alpha isoform (Ppp3ca) | -1.93 |
| Rps6 | ribosomal protein S6 (Rps6) | -1.94 |
| Ypel3 | yippee-like 3 (Drosophila) (Ypel3) | -1.98 |
| E330018D03Rik | RIKEN cDNA E330018D03 gene (E330018D03Rik) | -1.99 |
| D930015E06Rik | RIKEN cDNA D930015E06 gene (D930015E06Rik) | -1.99 |
| 1700021K19Rik | RIKEN cDNA 1700021K19 gene (1700021K19Rik) | -1.99 |
| Dgkz | diacylglycerol kinase zeta (Dgkz) | -2 |
| Cd79b | CD79B antigen (Cd79b) | -2 |
| Chst3 | carbohydrate (chondroitin 6/keratan) sulfotransferase 3 (Chst3) | -2 |
| Mrpl48 | mitochondrial ribosomal protein L48 (Mrpl48), nuclear gene encoding mitochondrial protein | -2.03 |
| Rpo1-3 | RNA polymerase 1-3 (Rpo1-3), transcript variant 1 | -2.04 |
| Sesn1 | sestrin 1 (Sesn1) | -2.04 |
| Xrcc1 | X-ray repair complementing defective repair in Chinese hamster cells 1 (Xrcc1) | -2.05 |
| Scrib | scribbled homolog (Drosophila) (Scrib) | -2.05 |
| Atpbd1b | ATP binding domain 1 family, member B (Atpbd1b) | -2.07 |
| Ep300 | E1A binding protein p300 (Ep300) | -2.07 |
| Cox7a2l | cytochrome c oxidase subunit VIIa polypeptide 2-like (Cox7a2l) | -2.07 |
| Tmem42 | transmembrane protein 42 (Tmem42) | -2.08 |
| Ephx1 | epoxide hydrolase 1, microsomal (Ephx1) | -2.08 |
| Lsm12 | LSM12 homolog (S. cerevisiae) (Lsm12) | -2.08 |
| Necap2 | NECAP endocytosis associated 2 (Necap2) | -2.09 |
| LOC676420 | similar to ceramide kinases (LOC676420), misc RNA. | -2.09 |
| Sidt2 | SID1 transmembrane family, member 2 (Sidt2) | -2.1 |
| Hvcn1 | hydrogen voltage-gated channel 1 (Hvcn1), transcript variant 1 | -2.1 |
| Vars | valyl-tRNA synthetase (Vars) | -2.11 |
| Ctsa | cathepsin A (Ctsa), transcript variant 2 | -2.12 |
| Pla2g12a | phospholipase A2, group XIIA (Pla2g12a), transcript variant 1 | -2.13 |
| Slc25a19 | solute carrier family 25 (mitochondrial thiamine pyrophosphate carrier), member 19 (Slc25a19), nuclear gene encoding mitochondrial protein | -2.16 |
| Lgals3 | lectin, galactose binding, soluble 3 (Lgals3) | -2.19 |
| Rnf144a | ring finger protein 144A (Rnf144a), transcript variant 2 | -2.19 |
| Napsa | napsin A aspartic peptidase (Napsa) | -2.2 |
| Ift140 | intraflagellar transport 140 homolog (Chlamydomonas) (Ift140) | -2.2 |
| Dusp2 | dual specificity phosphatase 2 (Dusp2) | -2.21 |
| AI467606 | expressed sequence AI467606 (AI467606) | -2.23 |
| Ppie | peptidylprolyl isomerase E (cyclophilin E) (Ppie) | -2.24 |
| Ptp4a3 | protein tyrosine phosphatase 4a3 (Ptp4a3) | -2.25 |
| Eif4el3 | eukaryotic translation initiation factor 4E member 2 (Eif4el3) | -2.31 |
| 4933439C20Rik | RIKEN cDNA 4933439C20 gene (4933439C20Rik) | -2.36 |
| Hvcn1 | hydrogen voltage-gated channel 1 (Hvcn1), transcript variant 1 | -2.37 |
| Rpo1-3 | RNA polymerase 1-3 (Rpo1-3), transcript variant 1 | -2.39 |
| Mgst1 | microsomal glutathione S-transferase 1 (Mgst1) | -2.4 |
| LOC545056 | ubiquitin-conjugating enzyme E2, J2 homolog pseudogene (LOC545056) on chromosome 14. | -2.41 |
| Vat1 | vesicle amine transport protein 1 homolog (T californica) (Vat1) | -2.41 |
| Zfp91 | zinc finger protein 91 (Zfp91) | -2.45 |
| Zfp592 | zinc finger protein 592 (Zfp592) | -2.46 |
| Atp1b1 | ATPase, Na+/K+ transporting, beta 1 polypeptide (Atp1b1) | -2.51 |
| Iqgap1 | IQ motif containing GTPase activating protein 1 (Iqgap1) | -2.53 |
| Btla | B and T lymphocyte associated (Btla), transcript variant 2 | -2.56 |
| BC067047 | cDNA sequence BC067047 (BC067047) | -2.63 |
| Klhl6 | kelch-like 6 (Drosophila) (Klhl6) | -2.64 |
| Tnfrsf4 | tumor necrosis factor receptor superfamily, member 4 (Tnfrsf4) | -2.65 |
| BC017643 | cDNA sequence BC017643 (BC017643) | -2.67 |
| Mysm1 | myb-like, SWIRM and MPN domains 1 (Mysm1) | -2.74 |
| Cyp4f18 | cytochrome P450, family 4, subfamily f, polypeptide 18 (Cyp4f18) | -2.77 |
| BC025076 | membrane magnesium transporter 2 (Mmgt2) | -2.85 |
| LOC100044439 | similar to cytochrome P450 CYP4F18 (LOC100044439) | -2.86 |
| LOC100046039 | similar to histone deacetylase HD1 (LOC100046039) | -2.88 |
| Arid3b | AT rich interactive domain 3B (Bright like) (Arid3b) | -2.92 |
| Rps3a | ribosomal protein S3a (Rps3a) | -2.92 |
| Dpagt1 | dolichyl-phosphate (UDP-N-acetylglucosamine) acetylglucosaminephosphotransferase 1 (GlcNAc-1-P transferase) (Dpagt1) | -2.95 |
| Tef | thyrotroph embryonic factor (Tef), transcript variant 1 | -2.96 |
| Sin3a | transcriptional regulator, SIN3A (yeast) (Sin3a) | -3.03 |
| Nfatc3 | nuclear factor of activated T-cells, cytoplasmic, calcineurin-dependent 3 (Nfatc3) | -3.04 |
| Klhl6 | kelch-like 6 (Drosophila) (Klhl6) | -3.06 |
| Tmem66 | transmembrane protein 66 (Tmem66) | -3.12 |
| Tsc2 | tuberous sclerosis 2 (Tsc2), transcript variant 2 | -3.12 |
| Bbc3 | Bcl-2 binding component 3 (Bbc3) | -3.12 |
| Hs3st1 | heparan sulfate (glucosamine) 3-O-sulfotransferase 1 (Hs3st1) | -3.12 |
| LOC100047214 | similar to PTEN induced putative kinase 1 (LOC100047214) | -3.22 |
| LOC100043257 | similar to RNA binding motif protein 3 (LOC100043257) | -3.25 |
| Nt5e | 5' nucleotidase, ecto (Nt5e) | -3.27 |
| Tspan3 | tetraspanin 3 (Tspan3) | -3.3 |
| Bcl9l | B cell CLL/lymphoma 9-like (Bcl9l) | -3.4 |
| Per1 | period homolog 1 (Drosophila) (Per1) | -3.42 |
| Acpl2 | acid phosphatase-like 2 (Acpl2) | -3.65 |
| Iap | intracisternal A particles (Iap) | -3.72 |
| Mllt3 | myeloid/lymphoid or mixed-lineage leukemia (trithorax homolog, Drosophila); translocated to, 3 (Mllt3), transcript variant 1 | -3.76 |
| Dusp7 | dual specificity phosphatase 7 (Dusp7) | -3.77 |
| Gsto1 | glutathione S-transferase omega 1 (Gsto1) | -3.81 |
| Dbp | D site albumin promoter binding protein (Dbp) | -3.88 |
| LOC100044862 | similar to Fbxl3 protein (LOC100044862) | -3.92 |
| Mgst2 | microsomal glutathione S-transferase 2 (Mgst2) | -3.93 |
| Galnt10 | UDP-N-acetyl-alpha-D-galactosamine:polypeptide N-acetylgalactosaminyltransferase 10 (Galnt10) | -3.94 |
| Tsc22d3 | TSC22 domain family 3 (Tsc22d3), transcript variant 1 | -3.98 |
| Axud1 | AXIN1 up-regulated 1 (Axud1) | -4.12 |
| 6430706D22Rik | RIKEN cDNA 6430706D22 gene (6430706D22Rik) | -4.14 |
| Pkm2 | pyruvate kinase, muscle (Pkm2) | -4.21 |
| Ddx6 | DEAD (Asp-Glu-Ala-Asp) box polypeptide 6 (Ddx6) | -4.47 |
| Bsdc1 | BSD domain containing 1 (Bsdc1) | -4.82 |
| Mef2c | myocyte enhancer factor 2C (Mef2c) | -4.95 |
| Bach1 | BTB and CNC homology 1 (Bach1) | -5.41 |
| Ddit4 | DNA-damage-inducible transcript 4 (Ddit4) | -5.8 |
| Zbtb7a | zinc finger and BTB domain containing 7a (Zbtb7a) | -6.57 |
