# Supplementary material for: Genomic deletion of GIT2 induces a premature age-related thymic dysfunction and systemic immune system disruption
Source: Aging (Albany NY). 2017 Mar 4;9(3):706–30. doi: 10.18632/aging.101185 (PMC5391227; doi:10.18632/aging.101185)
Supplement: Supplementary file 13 [file aging-09-706-s013.docx]

**Table S12. Transcripts significantly regulated differentially between the GIT2KO spleen and WT spleen.** The official Gene Symbol, transcript description and associated Z ratios for the comparison of the GIT2KO spleen *vs.* WT spleen at the 12 month time point are indicated. Each transcript was significantly regulated at p<0.05, with a Z ratio >± 1.5.

| **Gene Symbol** | **Description** | **Z ratio** |
| --- | --- | --- |
| Hspa8 | heat shock protein 8 (Hspa8) | 7.87 |
| Eef2 | eukaryotic translation elongation factor 2 (Eef2) | 6.06 |
| Hp | haptoglobin (Hp) | 5.97 |
| Sparc | secreted acidic cysteine rich glycoprotein (Sparc) | 5.96 |
| Hp | haptoglobin (Hp) | 5.76 |
| Eef2 | eukaryotic translation elongation factor 2 (Eef2) | 5.63 |
| Ces3 | carboxylesterase 3 (Ces3) | 5.44 |
| Cxcl12 | chemokine (C-X-C motif) ligand 12 (Cxcl12), transcript variant 3 | 5.32 |
| Actb | actin, beta, cytoplasmic (Actb) | 5.24 |
| Hp | haptoglobin (Hp) | 5.18 |
| Aqp1 | aquaporin 1 (Aqp1) | 5.14 |
| Cxcl12 | chemokine (C-X-C motif) ligand 12 (Cxcl12), transcript variant 1 | 5 |
| Eif5a | eukaryotic translation initiation factor 5A (Eif5a) | 4.99 |
| Mgst3 | microsomal glutathione S-transferase 3 (Mgst3) | 4.95 |
| Eef2 | eukaryotic translation elongation factor 2 (Eef2) | 4.95 |
| Gpnmb | glycoprotein (transmembrane) nmb (Gpnmb) | 4.9 |
| Hist1h2bj | histone cluster 1, H2bj (Hist1h2bj) | 4.89 |
| Hist1h2bf | histone cluster 1, H2bf (Hist1h2bf) | 4.82 |
| Hist1h2bh | histone cluster 1, H2bh (Hist1h2bh) | 4.77 |
| Hist1h2ah | histone cluster 1, H2ah (Hist1h2ah) | 4.76 |
| H2-T10 | histocompatibility 2, T region locus 10 (H2-T10) | 4.57 |
| Ppp1ca | protein phosphatase 1, catalytic subunit, alpha isoform (Ppp1ca) | 4.55 |
| Rnase4 | ribonuclease, RNase A family 4 (Rnase4), transcript variant 1 | 4.51 |
| Aqp1 | aquaporin 1 (Aqp1) | 4.28 |
| Hist1h2ag | histone cluster 1, H2ag (Hist1h2ag) | 4.23 |
| Clec4n | C-type lectin domain family 4, member n (Clec4n) | 4.22 |
| Idh3g | isocitrate dehydrogenase 3 (NAD+), gamma (Idh3g), nuclear gene encoding mitochondrial protein | 4.19 |
| Hist1h2ad | histone cluster 1, H2ad (Hist1h2ad) | 4.15 |
| Hist1h2ak | histone cluster 1, H2ak (Hist1h2ak) | 4.12 |
| Lmna | lamin A (Lmna), transcript variant 2 | 4.06 |
| Hnrpk | heterogeneous nuclear ribonucleoprotein K (Hnrpk) | 3.97 |
| Txnl4a | thioredoxin-like 4A (Txnl4a), transcript variant 2 | 3.92 |
| Mylk | myosin, light polypeptide kinase (Mylk) | 3.84 |
| Hsp90ab1 | heat shock protein 90kDa alpha (cytosolic), class B member 1 (Hsp90ab1) | 3.83 |
| Actn4 | actinin alpha 4 (Actn4) | 3.73 |
| Lgmn | legumain (Lgmn) | 3.73 |
| Kras | v-Ki-ras2 Kirsten rat sarcoma viral oncogene homolog (Kras) | 3.72 |
| Hsp90ab1 | heat shock protein 90kDa alpha (cytosolic), class B member 1 (Hsp90ab1) | 3.7 |
| Tmem14c | transmembrane protein 14C (Tmem14c) | 3.58 |
| Pld3 | phospholipase D family, member 3 (Pld3) | 3.57 |
| Tmem33 | transmembrane protein 33 (Tmem33), transcript variant 2 | 3.52 |
| Gstp1 | glutathione S-transferase, pi 1 (Gstp1) | 3.51 |
| Adi1 | acireductone dioxygenase 1 (Adi1) | 3.49 |
| S100a1 | S100 calcium binding protein A1 (S100a1) | 3.48 |
| Tnfaip2 | tumor necrosis factor, alpha-induced protein 2 (Tnfaip2) | 3.43 |
| Arpc1a | actin related protein 2/3 complex, subunit 1A (Arpc1a) | 3.41 |
| Sepp1 | selenoprotein P, plasma, 1 (Sepp1), transcript variant 1 | 3.4 |
| Actn4 | actinin alpha 4 (Actn4) | 3.39 |
| Gnas | GNAS (guanine nucleotide binding protein, alpha stimulating) complex locus (Gnas), transcript variant 3 | 3.39 |
| Atp5f1 | ATP synthase, H+ transporting, mitochondrial F0 complex, subunit b, isoform 1 (Atp5f1) | 3.39 |
| Hmgb2 | high mobility group box 2 (Hmgb2) | 3.32 |
| Asns | asparagine synthetase (Asns) | 3.32 |
| EG434858 | predicted gene, EG434858 (EG434858) on chromosome X. | 3.32 |
| Ccl19 | chemokine (C-C motif) ligand 19 (Ccl19) | 3.32 |
| Sept15 | selenoprotein (Sep15) | 3.31 |
| Adk | adenosine kinase (Adk) | 3.28 |
| LOC100044204 | hypothetical protein LOC100044204 (LOC100044204) | 3.28 |
| Trf | transferrin (Trf) | 3.26 |
| Hnrnpa2b1 | heterogeneous nuclear ribonucleoprotein A2/B1 (Hnrnpa2b1), transcript variant 2 | 3.23 |
| Cap1 | CAP, adenylate cyclase-associated protein 1 (yeast) (Cap1) | 3.21 |
| Col4a2 | collagen, type IV, alpha 2 (Col4a2) | 3.2 |
| Mpp1 | membrane protein, palmitoylated (Mpp1) | 3.2 |
| Tubb2b | tubulin, beta 2b (Tubb2b) | 3.19 |
| Glo1 | glyoxalase 1 (Glo1) | 3.1 |
| Lamp2 | lysosomal-associated membrane protein 2 (Lamp2), transcript variant 1 | 3.08 |
| 5730437N04Rik | RIKEN cDNA 5730437N04 gene (5730437N04Rik) | 3.03 |
| Tbc1d20 | TBC1 domain family, member 20 (Tbc1d20) | 3.02 |
| Pcyt1a | phosphate cytidylyltransferase 1, choline, alpha isoform (Pcyt1a) | 3.02 |
| Gch1 | GTP cyclohydrolase 1 (Gch1) | 3.02 |
| Sphk1 | sphingosine kinase 1 (Sphk1), transcript variant 1 | 3.02 |
| Rnasek | ribonuclease, RNase K (Rnasek) | 3.02 |
| Psmd7 | proteasome (prosome, macropain) 26S subunit, non-ATPase, 7 (Psmd7) | 3.02 |
| C1qc | complement component 1, q subcomponent, C chain (C1qc) | 3.01 |
| EG433923 | predicted gene, EG433923 (EG433923) | 3.01 |
| Naca | nascent polypeptide-associated complex alpha polypeptide (Naca) | 3 |
| LOC100038882 | hypothetical protein LOC100038882 (LOC100038882) | 3 |
| Dcn | decorin (Dcn) | 2.99 |
| Lmna | lamin A (Lmna), transcript variant 2 | 2.98 |
| D10Ertd322e | DNA segment, Chr 10, ERATO Doi 322, expressed (D10Ertd322e) | 2.98 |
| Enpp4 | ectonucleotide pyrophosphatase/phosphodiesterase 4 (Enpp4) | 2.97 |
| Gstm1 | glutathione S-transferase, mu 1 (Gstm1) | 2.96 |
| Cd63 | Cd63 antigen (Cd63), transcript variant 2 | 2.94 |
| Csrp1 | cysteine and glycine-rich protein 1 (Csrp1) | 2.93 |
| Spcs1 | signal peptidase complex subunit 1 homolog (S. cerevisiae) (Spcs1) | 2.91 |
| Ptp4a2 | protein tyrosine phosphatase 4a2 (Ptp4a2) | 2.89 |
| Atf4 | activating transcription factor 4 (Atf4) | 2.86 |
| Caprin1 | cell cycle associated protein 1 (Caprin1) | 2.85 |
| Bola3 | bolA-like 3 (E. coli) (Bola3) | 2.85 |
| C1qb | complement component 1, q subcomponent, beta polypeptide (C1qb) | 2.84 |
| Mylk | myosin, light polypeptide kinase (Mylk) | 2.83 |
| Mgst1 | microsomal glutathione S-transferase 1 (Mgst1) | 2.82 |
| Psmb7 | proteasome (prosome, macropain) subunit, beta type 7 (Psmb7) | 2.81 |
| Rarres2 | retinoic acid receptor responder (tazarotene induced) 2 (Rarres2) | 2.81 |
| Fgd2 | FYVE, RhoGEF and PH domain containing 2 (Fgd2) | 2.8 |
| Pfn1 | profilin 1 (Pfn1) | 2.79 |
| Coro1a | coronin, actin binding protein 1A (Coro1a) | 2.79 |
| Ifitm2 | interferon induced transmembrane protein 2 (Ifitm2) | 2.78 |
| Dcps | decapping enzyme, scavenger (Dcps) | 2.78 |
| Ezh2 | enhancer of zeste homolog 2 (Drosophila) (Ezh2) | 2.77 |
| Sparc | secreted acidic cysteine rich glycoprotein (Sparc) | 2.77 |
| Mthfd1 | methylenetetrahydrofolate dehydrogenase (NADP+ dependent), methenyltetrahydrofolate cyclohydrolase, formyltetrahydrofolate synthase (Mthfd1) | 2.73 |
| Gpsn2 | glycoprotein, synaptic 2 (Gpsn2) | 2.72 |
| Ly6c1 | lymphocyte antigen 6 complex, locus C1 (Ly6c1) | 2.72 |
| Mgp | matrix Gla protein (Mgp) | 2.71 |
| Hrbl | HIV-1 Rev binding protein-like (Hrbl), transcript variant 2 | 2.71 |
| Cdca3 | cell division cycle associated 3 (Cdca3) | 2.71 |
| Tmem205 | transmembrane protein 205 (Tmem205) | 2.69 |
| Pdhb | pyruvate dehydrogenase (lipoamide) beta (Pdhb) | 2.69 |
| Pcbp1 | poly(rC) binding protein 1 (Pcbp1) | 2.68 |
| Tuba1a | tubulin, alpha 1A (Tuba1a) | 2.68 |
| Cd9 | CD9 antigen (Cd9) | 2.68 |
| Prdx3 | peroxiredoxin 3 (Prdx3) | 2.67 |
| Oxct1 | 3-oxoacid CoA transferase 1 (Oxct1) | 2.66 |
| H13 | histocompatibility 13 (H13) | 2.66 |
| Tspo | translocator protein (Tspo) | 2.65 |
| Tspo | translocator protein (Tspo) | 2.64 |
| Cd209b | CD209b antigen (Cd209b), transcript variant 1 | 2.63 |
| Hist1h2bk | histone cluster 1, H2bk (Hist1h2bk) | 2.63 |
| Fkbp2 | FK506 binding protein 2 (Fkbp2) | 2.63 |
| Stx8 | syntaxin 8 (Stx8) | 2.63 |
| Ppm1g | protein phosphatase 1G (formerly 2C), magnesium-dependent, gamma isoform (Ppm1g) | 2.63 |
| Mylc2b | myosin light chain, regulatory B (Mylc2b) | 2.61 |
| Arpc5 | actin related protein 2/3 complex, subunit 5 (Arpc5) | 2.6 |
| Rpl38 | ribosomal protein L38 (Rpl38), transcript variant 1 | 2.6 |
| Rps27a | ribosomal protein S27a (Rps27a), transcript variant 2 | 2.59 |
| Mrpl53 | mitochondrial ribosomal protein L53 (Mrpl53), nuclear gene encoding mitochondrial protein | 2.58 |
| Hist1h2af | histone cluster 1, H2af (Hist1h2af) | 2.58 |
| Fxn | frataxin (Fxn) | 2.56 |
| Vwf | Von Willebrand factor homolog (Vwf) | 2.56 |
| Mcm2 | minichromosome maintenance deficient 2 mitotin (S. cerevisiae) (Mcm2) | 2.55 |
| Capzb | capping protein (actin filament) muscle Z-line, beta (Capzb), transcript variant 1 | 2.52 |
| Naca | nascent polypeptide-associated complex alpha polypeptide (Naca) | 2.5 |
| Arl6ip5 | ADP-ribosylation factor-like 6 interacting protein 5 (Arl6ip5) | 2.49 |
| Sqle | squalene epoxidase (Sqle) | 2.49 |
| EG622339 | predicted gene, EG622339 (EG622339) | 2.48 |
| Xpnpep1 | X-prolyl aminopeptidase (aminopeptidase P) 1, soluble (Xpnpep1) | 2.46 |
| Zfp91 | zinc finger protein 91 (Zfp91) | 2.46 |
| Gnb1 | guanine nucleotide binding protein (G protein), beta 1 (Gnb1) | 2.46 |
| Snrpd2 | small nuclear ribonucleoprotein D2 (Snrpd2) | 2.46 |
| Cox6b1 | cytochrome c oxidase, subunit VIb polypeptide 1 (Cox6b1) | 2.46 |
| Nde1 | nuclear distribution gene E homolog 1 (A nidulans) (Nde1) | 2.46 |
| Hagh | hydroxyacyl glutathione hydrolase (Hagh) | 2.45 |
| Ppp2r5c | protein phosphatase 2, regulatory subunit B (B56), gamma isoform (Ppp2r5c), transcript variant 3 | 2.45 |
| Mcm5 | minichromosome maintenance deficient 5, cell division cycle 46 (S. cerevisiae) (Mcm5) | 2.43 |
| Eno1 | enolase 1, alpha non-neuron (Eno1) | 2.42 |
| Sec61b | Sec61 beta subunit (Sec61b) | 2.41 |
| Slc11a1 | solute carrier family 11 (proton-coupled divalent metal ion transporters), member 1 (Slc11a1) | 2.4 |
| Vdac3 | voltage-dependent anion channel 3 (Vdac3) | 2.4 |
| Gabarapl1 | gamma-aminobutyric acid (GABA(A)) receptor-associated protein-like 1 (Gabarapl1) | 2.39 |
| Cd97 | CD97 antigen (Cd97) | 2.39 |
| Ddx47 | DEAD (Asp-Glu-Ala-Asp) box polypeptide 47 (Ddx47) | 2.38 |
| Hsd11b1 | hydroxysteroid 11-beta dehydrogenase 1 (Hsd11b1), transcript variant 1 | 2.38 |
| Idh1 | isocitrate dehydrogenase 1 (NADP+), soluble (Idh1) | 2.36 |
| Snrpd1 | small nuclear ribonucleoprotein D1 (Snrpd1) | 2.36 |
| Mboat5 | membrane bound O-acyltransferase domain containing 5 (Mboat5) | 2.35 |
| Tmem2 | transmembrane protein 2 (Tmem2), transcript variant 2 | 2.35 |
| Ndufc1 | NADH dehydrogenase (ubiquinone) 1, subcomplex unknown, 1 (Ndufc1) | 2.35 |
| Nudt4 | nudix (nucleoside diphosphate linked moiety X)-type motif 4 (Nudt4) | 2.35 |
| MGC18837 | transmembrane protein 205 (Tmem205) | 2.32 |
| Map3k3 | mitogen-activated protein kinase kinase kinase 3 (Map3k3) | 2.3 |
| Ndufa12 | NADH dehydrogenase (ubiquinone) 1 alpha subcomplex, 12 (Ndufa12) | 2.29 |
| Cygb | cytoglobin (Cygb) | 2.29 |
| Plp2 | proteolipid protein 2 (Plp2) | 2.28 |
| Bckdk | branched chain ketoacid dehydrogenase kinase (Bckdk), nuclear gene encoding mitochondrial protein | 2.28 |
| LOC100041835 | similar to H+ ATP synthase (LOC100041835) | 2.28 |
| Fermt3 | fermitin family homolog 3 (Drosophila) (Fermt3) | 2.27 |
| Sumo3 | SMT3 suppressor of mif two 3 homolog 3 (yeast) (Sumo3) | 2.27 |
| Aebp1 | AE binding protein 1 (Aebp1) | 2.26 |
| Usp39 | ubiquitin specific peptidase 39 (Usp39) | 2.26 |
| Psmb2 | proteasome (prosome, macropain) subunit, beta type 2 (Psmb2) | 2.25 |
| Prkcdbp | protein kinase C, delta binding protein (Prkcdbp) | 2.25 |
| H13 | histocompatibility 13 (H13) | 2.24 |
| Nola2 | nucleolar protein family A, member 2 (Nola2) | 2.24 |
| Cd151 | CD151 antigen (Cd151) | 2.24 |
| Col4a1 | procollagen, type IV, alpha 1 (Col4a1) | 2.24 |
| C1qa | complement component 1, q subcomponent, alpha polypeptide (C1qa) | 2.24 |
| Samd14 | sterile alpha motif domain containing 14 (Samd14), transcript variant 1 | 2.23 |
| Lyz2 | lysozyme 2 (Lyz2) | 2.23 |
| Psma7 | proteasome (prosome, macropain) subunit, alpha type 7 (Psma7) | 2.22 |
| Supt4h2 | suppressor of Ty 4 homolog 2 (S. cerevisiae) (Supt4h2) | 2.22 |
| Cox6a1 | cytochrome c oxidase subunit VIa polypeptide 1 (Cox6a1) | 2.21 |
| Dci | dodecenoyl-Coenzyme A delta isomerase (3,2 trans-enoyl-Coenyme A isomerase) (Dci), nuclear gene encoding mitochondrial protein | 2.21 |
| Emp1 | epithelial membrane protein 1 (Emp1) | 2.2 |
| D8Ertd738e | DNA segment, Chr 8, ERATO Doi 738, expressed (D8Ertd738e) | 2.2 |
| Vps25 | vacuolar protein sorting 25 (yeast) (Vps25) | 2.19 |
| Smu1 | smu-1 suppressor of mec-8 and unc-52 homolog (C. elegans) (Smu1) | 2.19 |
| Gfer | growth factor, erv1 (S. cerevisiae)-like (augmenter of liver regeneration) (Gfer) | 2.19 |
| Psma7 | proteasome (prosome, macropain) subunit, alpha type 7 (Psma7) | 2.18 |
| Galnt1 | UDP-N-acetyl-alpha-D-galactosamine:polypeptide N-acetylgalactosaminyltransferase 1 (Galnt1) | 2.18 |
| Cox6a1 | cytochrome c oxidase, subunit VI a, polypeptide 1 (Cox6a1), nuclear gene encoding mitochondrial protein | 2.18 |
| Gstm2 | glutathione S-transferase, mu 2 (Gstm2) | 2.17 |
| Tmem9 | transmembrane protein 9 (Tmem9) | 2.16 |
| Ppp2r1a | protein phosphatase 2 (formerly 2A), regulatory subunit A (PR 65), alpha isoform (Ppp2r1a) | 2.16 |
| Eng | endoglin (Eng) | 2.15 |
| Yif1b | Yip1 interacting factor homolog B (S. cerevisiae) (Yif1b) | 2.15 |
| Pscd3 | pleckstrin homology, Sec7 and coiled-coil domains 3 (Pscd3) | 2.15 |
| Hip2 | ubiquitin-conjugating enzyme E2K (UBC1 homolog, yeast) (Ube2k) | 2.15 |
| Slco3a1 | solute carrier organic anion transporter family, member 3a1 (Slco3a1), transcript variant 1 | 2.14 |
| Eif5 | eukaryotic translation initiation factor 5 (Eif5), transcript variant 1 | 2.14 |
| Ndufa5 | NADH dehydrogenase (ubiquinone) 1 alpha subcomplex, 5 (Ndufa5), nuclear gene encoding mitochondrial protein | 2.12 |
| Ak2 | adenylate kinase 2 (Ak2), transcript variant 2 | 2.12 |
| Cyc1 | cytochrome c-1 (Cyc1) | 2.12 |
| Hebp1 | heme binding protein 1 (Hebp1) | 2.12 |
| Fdps | farnesyl diphosphate synthetase (Fdps) | 2.12 |
| Mid1ip1 | Mid1 interacting protein 1 (gastrulation specific G12-like (zebrafish)) (Mid1ip1) | 2.11 |
| Timm8b | translocase of inner mitochondrial membrane 8 homolog b (yeast) (Timm8b) | 2.11 |
| Hist1h2an | histone cluster 1, H2an (Hist1h2an) | 2.11 |
| Ndufc1 | NADH dehydrogenase (ubiquinone) 1, subcomplex unknown, 1 (Ndufc1) | 2.1 |
| 2510006D16Rik | RIKEN cDNA 2510006D16 gene (2510006D16Rik) | 2.09 |
| 5730593F17Rik | RIKEN cDNA 5730593F17 gene (5730593F17Rik) | 2.09 |
| Col6a1 | procollagen, type VI, alpha 1 (Col6a1) | 2.09 |
| Aoc3 | amine oxidase, copper containing 3 (Aoc3) | 2.08 |
| Srm | spermidine synthase (Srm) | 2.08 |
| Nucb1 | nucleobindin 1 (Nucb1) | 2.08 |
| Tnfrsf21 | tumor necrosis factor receptor superfamily, member 21 (Tnfrsf21) | 2.08 |
| 2310016E02Rik | RIKEN cDNA 2310016E02 gene (2310016E02Rik) | 2.07 |
| 9430029K10Rik | Yip1 interacting factor homolog B (S. cerevisiae) (Yif1b) | 2.07 |
| Snrpd1 | small nuclear ribonucleoprotein D1 (Snrpd1) | 2.07 |
| Tfdp1 | transcription factor Dp 1 (Tfdp1) | 2.07 |
| Rpl7a | ribosomal protein L7a (Rpl7a) | 2.06 |
| Vps29 | vacuolar protein sorting 29 (S. pombe) (Vps29) | 2.06 |
| Ilk | integrin linked kinase (Ilk) | 2.05 |
| Lpl | lipoprotein lipase (Lpl) | 2.05 |
| 1190002H23Rik | RIKEN cDNA 1190002H23 gene (1190002H23Rik) | 2.05 |
| Dhx15 | DEAH (Asp-Glu-Ala-His) box polypeptide 15 (Dhx15), transcript variant 1 | 2.05 |
| Hsd11b1 | hydroxysteroid 11-beta dehydrogenase 1 (Hsd11b1), transcript variant 1 | 2.02 |
| Mrpl34 | mitochondrial ribosomal protein L34 (Mrpl34), nuclear gene encoding mitochondrial protein | 2.02 |
| Psmb7 | proteasome (prosome, macropain) subunit, beta type 7 (Psmb7) | 2.01 |
| Lcp1 | lymphocyte cytosolic protein 1 (Lcp1) | 2.01 |
| 1810009A15Rik | RIKEN cDNA 1810009A15 gene (1810009A15Rik) | 2.01 |
| Trappc1 | trafficking protein particle complex 1 (Trappc1) | 2.01 |
| Vkorc1 | vitamin K epoxide reductase complex, subunit 1 (Vkorc1) | 2 |
| BC017647 | cDNA sequence BC017647 (BC017647) | 2 |
| Pik3cg | phosphoinositide-3-kinase, catalytic, gamma polypeptide (Pik3cg) | 1.99 |
| Tmem14c | transmembrane protein 14C (Tmem14c) | 1.99 |
| Akap12 | A kinase (PRKA) anchor protein (gravin) 12 (Akap12) | 1.98 |
| Stard10 | START domain containing 10 (Stard10) | 1.97 |
| Emp2 | epithelial membrane protein 2 (Emp2) | 1.97 |
| P2ry6 | pyrimidinergic receptor P2Y, G-protein coupled, 6 (P2ry6) | 1.97 |
| Zfp207 | zinc finger protein 207 (Zfp207) | 1.97 |
| Rpl24 | ribosomal protein L24 (Rpl24) | 1.96 |
| Bcap31 | B-cell receptor-associated protein 31 (Bcap31) | 1.96 |
| 4933434E20Rik | RIKEN cDNA 4933434E20 gene (4933434E20Rik), transcript variant 1 | 1.95 |
| Fcna | ficolin A (Fcna) | 1.95 |
| Tmem204 | transmembrane protein 204 (Tmem204) | 1.95 |
| Gde1 | glycerophosphodiester phosphodiesterase 1 (Gde1) | 1.95 |
| Nomo1 | nodal modulator 1 (Nomo1) | 1.94 |
| LOC100046650 | similar to PRELI domain containing 1 (LOC100046650) | 1.94 |
| Calu | calumenin (Calu), transcript variant 1 | 1.94 |
| Chmp2a | chromatin modifying protein 2A (Chmp2a) | 1.94 |
| Fhl1 | four and a half LIM domains 1 (Fhl1), transcript variant 2 | 1.94 |
| Fhl1 | four and a half LIM domains 1 (Fhl1), transcript variant 1 | 1.94 |
| Tbc1d2b | TBC1 domain family, member 2B (Tbc1d2b) | 1.93 |
| Mrps28 | mitochondrial ribosomal protein S28 (Mrps28), nuclear gene encoding mitochondrial protein | 1.93 |
| Prpf19 | PRP19/PSO4 pre-mRNA processing factor 19 homolog (S. cerevisiae) (Prpf19) | 1.92 |
| App | amyloid precursor protein (App) | 1.91 |
| Nudt5 | nudix (nucleoside diphosphate linked moiety X)-type motif 5 (Nudt5) | 1.91 |
| Ssna1 | Sjogren's syndrome nuclear autoantigen 1 (Ssna1) | 1.9 |
| H2afz | H2A histone family, member Z (H2afz) | 1.89 |
| Cope | coatomer protein complex, subunit epsilon (Cope) | 1.89 |
| Cd151 | CD151 antigen (Cd151) | 1.89 |
| Eif4a1 | eukaryotic translation initiation factor 4A1 (Eif4a1) | 1.88 |
| Ube2k | ubiquitin-conjugating enzyme E2K (UBC1 homolog, yeast) (Ube2k) | 1.88 |
| Polr2f | polymerase (RNA) II (DNA directed) polypeptide F (Polr2f) | 1.87 |
| Papola | poly (A) polymerase alpha (Papola) | 1.87 |
| Fbxo9 | f-box protein 9 (Fbxo9), transcript variant 1 | 1.87 |
| Dhrs1 | dehydrogenase/reductase (SDR family) member 1 (Dhrs1) | 1.86 |
| Cxcl9 | chemokine (C-X-C motif) ligand 9 (Cxcl9) | 1.85 |
| H2afx | H2A histone family, member X (H2afx) | 1.85 |
| 2310022B05Rik | RIKEN cDNA 2310022B05 gene (2310022B05Rik) | 1.85 |
| Sepx1 | selenoprotein X 1 (Sepx1) | 1.85 |
| Vps25 | vacuolar protein sorting 25 (yeast) (Vps25) | 1.84 |
| Hsd11b1 | hydroxysteroid 11-beta dehydrogenase 1 (Hsd11b1), transcript variant 1 | 1.84 |
| Tmem2 | transmembrane protein 2 (Tmem2), transcript variant 1 | 1.84 |
| Mmp2 | matrix metallopeptidase 2 (Mmp2) | 1.84 |
| Lsm2 | LSM2 homolog, U6 small nuclear RNA associated (S. cerevisiae) (Lsm2) | 1.84 |
| Ndufb10 | NADH dehydrogenase (ubiquinone) 1 beta subcomplex, 10 (Ndufb10) | 1.84 |
| Ap2s1 | adaptor-related protein complex 2, sigma 1 subunit (Ap2s1) | 1.83 |
| Nfix | nuclear factor I/X (Nfix), transcript variant 2 | 1.83 |
| Sertad2 | SERTA domain containing 2 (Sertad2), transcript variant 1 | 1.83 |
| Cmtm7 | CKLF-like MARVEL transmembrane domain containing 7 (Cmtm7) | 1.82 |
| Cnot7 | CCR4-NOT transcription complex, subunit 7 (Cnot7) | 1.82 |
| Cope | coatomer protein complex, subunit epsilon (Cope) | 1.82 |
| Pfkm | phosphofructokinase, muscle (Pfkm) | 1.82 |
| Capns1 | calpain, small subunit 1 (Cans1) | 1.81 |
| 1500032L24Rik | RIKEN cDNA 1500032L24 gene (1500032L24Rik) | 1.81 |
| Snrpb | small nuclear ribonucleoprotein B (Snrpb) | 1.8 |
| Plvap | plasmalemma vesicle associated protein (Plvap) | 1.8 |
| Ndufa8 | NADH dehydrogenase (ubiquinone) 1 alpha subcomplex, 8 (Ndufa8), nuclear gene encoding mitochondrial protein | 1.79 |
| Ahsa1 | AHA1, activator of heat shock protein ATPase homolog 1 (yeast) (Ahsa1) | 1.79 |
| Igfbp4 | insulin-like growth factor binding protein 4 (Igfbp4) | 1.79 |
| Cox17 | cytochrome c oxidase, subunit XVII assembly protein homolog (yeast) (Cox17) | 1.79 |
| Atp6v0d1 | ATPase, H+ transporting, lysosomal V0 subunit D1 (Atp6v0d1) | 1.79 |
| Ruvbl2 | RuvB-like protein 2 (Ruvbl2) | 1.79 |
| LOC100048613 | similar to cytochrome c oxidase, subunit VIIc, transcript variant 1 (LOC100048613) | 1.78 |
| Asna1 | arsA (bacterial) arsenite transporter, ATP-binding, homolog 1 (Asna1) | 1.78 |
| Ppap2a | phosphatidic acid phosphatase 2a (Ppap2a), transcript variant 1 | 1.78 |
| Nucb1 | nucleobindin 1 (Nucb1) | 1.77 |
| Cnr2 | cannabinoid receptor 2 (macrophage) (Cnr2) | 1.77 |
| Gtf3a | general transcription factor III A (Gtf3a) | 1.77 |
| Mrpl3 | mitochondrial ribosomal protein L3 (Mrpl3), nuclear gene encoding mitochondrial protein | 1.76 |
| Gas6 | growth arrest specific 6 (Gas6) | 1.76 |
| March2 | membrane-associated ring finger (C3HC4) 2 (March2) | 1.76 |
| Ndufs2 | NADH dehydrogenase (ubiquinone) Fe-S protein 2 (Ndufs2) | 1.76 |
| Tmem86a | transmembrane protein 86A (Tmem86a) | 1.76 |
| Gpx4 | glutathione peroxidase 4 (Gpx4), transcript variant 1 | 1.75 |
| Clcn3 | chloride channel 3 (Clcn3), transcript variant a | 1.75 |
| Cd79b | CD79B antigen (Cd79b) | 1.75 |
| Bud31 | BUD31 homolog (yeast) (Bud31) | 1.75 |
| Yif1a | Yip1 interacting factor homolog A (S. cerevisiae) (Yif1a) | 1.75 |
| Map2k3 | mitogen activated protein kinase kinase 3 (Map2k3) | 1.75 |
| Cdkn2c | cyclin-dependent kinase inhibitor 2C (p18, inhibits CDK4) (Cdkn2c) | 1.74 |
| Ncapd3 | non-SMC condensin II complex, subunit D3 (Ncapd3) | 1.74 |
| Ebp | phenylalkylamine Ca2+ antagonist (emopamil) binding protein (Ebp) | 1.74 |
| Igtp | interferon gamma induced GTPase (Igtp) | 1.73 |
| Add1 | adducin 1 (alpha) (Add1), transcript variant 1 | 1.73 |
| Gse1 | genetic suppressor element 1 (Gse1) | 1.73 |
| Cdt1 | chromatin licensing and DNA replication factor 1 (Cdt1) | 1.72 |
| Fbxo42 | F-box protein 42 (Fbxo42) | 1.72 |
| LOC100044294 | hypothetical protein LOC100044294 (LOC100044294) | 1.72 |
| Adamts2 | a disintegrin-like and metallopeptidase (reprolysin type) with thrombospondin type 1 motif, 2 (Adamts2) | 1.72 |
| Dguok | deoxyguanosine kinase (Dguok), nuclear gene encoding mitochondrial protein | 1.72 |
| Apoa1bp | apolipoprotein A-I binding protein (Apoa1bp) | 1.71 |
| Fxyd5 | FXYD domain-containing ion transport regulator 5 (Fxyd5) | 1.71 |
| H2-Ab1 | histocompatibility 2, class II antigen A, beta 1 (H2-Ab1) | 1.71 |
| LOC100043671 | hypothetical protein LOC100043671 (LOC100043671) | 1.71 |
| Smarcb1 | SWI/SNF related, matrix associated, actin dependent regulator of chromatin, subfamily b, member 1 (Smarcb1) | 1.7 |
| Nola3 | nucleolar protein family A, member 3 (Nola3) | 1.7 |
| Bnip2 | BCL2/adenovirus E1B interacting protein 1, NIP2 (Bnip2), transcript variant alpha | 1.7 |
| Ngfrap1 | nerve growth factor receptor (TNFRSF16) associated protein 1 (Ngfrap1) | 1.69 |
| Stim1 | stromal interaction molecule 1 (Stim1) | 1.69 |
| Exosc10 | exosome component 10 (Exosc10) | 1.68 |
| 0610006I08Rik | RIKEN cDNA 0610006I08 gene (0610006I08Rik) | 1.67 |
| Arpc1a | actin related protein 2/3 complex, subunit 1A (Arpc1a) | 1.67 |
| Sae1 | SUMO1 activating enzyme subunit 1 (Sae1) | 1.66 |
| Smarce1 | SWI/SNF related, matrix associated, actin dependent regulator of chromatin, subfamily e, member 1 (Smarce1) | 1.66 |
| Fen1 | flap structure specific endonuclease 1 (Fen1) | 1.65 |
| Maged2 | melanoma antigen, family D, 2 (Maged2) | 1.65 |
| Dph3 | DPH3 homolog (KTI11, S. cerevisiae) (Dph3), transcript variant 1 | 1.64 |
| Snx15 | sorting nexin 15 (Snx15) | 1.63 |
| Uck2 | uridine-cytidine kinase 2 (Uck2) | 1.62 |
| 2610029G23Rik | RIKEN cDNA 2610029G23 gene (2610029G23Rik) | 1.62 |
| Ndufs4 | NADH dehydrogenase (ubiquinone) Fe-S protein 4 (Ndufs4), nuclear gene encoding mitochondrial protein | 1.62 |
| Prmt5 | protein arginine N-methyltransferase 5 (Prmt5) | 1.61 |
| Zfand2a | zinc finger, AN1-type domain 2A (Zfand2a) | 1.6 |
| Ssr2 | signal sequence receptor, beta (Ssr2) | 1.59 |
| Cops6 | COP9 (constitutive photomorphogenic) homolog, subunit 6 (Arabidopsis thaliana) (Cops6) | 1.59 |
| Loxl1 | lysyl oxidase-like 1 (Loxl1) | 1.59 |
| Snrpa | small nuclear ribonucleoprotein polypeptide A (Snrpa), transcript variant 2 | 1.58 |
| Sdf2 | stromal cell derived factor 2 (Sdf2) | 1.57 |
| Gpsn2 | glycoprotein, synaptic 2 (Gpsn2) | 1.56 |
| Eif2ak1 | eukaryotic translation initiation factor 2 alpha kinase 1 (Eif2ak1) | 1.56 |
| Stip1 | stress-induced phosphoprotein 1 (Stip1) | 1.56 |
| Ndufb6 | NADH dehydrogenase (ubiquinone) 1 beta subcomplex, 6 (Ndufb6), nuclear gene encoding mitochondrial protein | 1.56 |
| Vim | vimentin (Vim) | 1.56 |
| Elovl1 | elongation of very long chain fatty acids (FEN1/Elo2, SUR4/Elo3, yeast)-like 1 (Elovl1), transcript variant 2 | 1.55 |
| Rsl1d1 | ribosomal L1 domain containing 1 (Rsl1d1) | 1.54 |
| Suclg2 | succinate-Coenzyme A ligase, GDP-forming, beta subunit (Suclg2) | 1.54 |
| Tmem33 | transmembrane protein 33 (Tmem33), transcript variant 1 | 1.53 |
| Fermt2 | fermitin family homolog 2 (Drosophila) (Fermt2) | 1.53 |
| Cpt2 | carnitine palmitoyltransferase 2 (Cpt2) | 1.52 |
| Smarce1 | SWI/SNF related, matrix associated, actin dependent regulator of chromatin, subfamily e, member 1 (Smarce1) | 1.51 |
| Wdr1 | WD repeat domain 1 (Wdr1) | 1.51 |
| Vars2 | valyl-tRNA synthetase 2, mitochondrial (putative) (Vars2) | 1.5 |
| Tyrobp | TYRO protein tyrosine kinase binding protein (Tyrobp) | 1.5 |
| Ttc3 | tetratricopeptide repeat domain 3 (Ttc3) | -1.5 |
| Hnrpl | heterogeneous nuclear ribonucleoprotein L (Hnrpl) | -1.5 |
| Vamp2 | vesicle-associated membrane protein 2 (Vamp2) | -1.5 |
| S100a10 | S100 calcium binding protein A10 (calpactin) (S100a10) | -1.51 |
| Mrps6 | mitochondrial ribosomal protein S6 (Mrps6), nuclear gene encoding mitochondrial protein | -1.51 |
| Arhgap17 | Rho GTPase activating protein 17 (Arhgap17) | -1.51 |
| Entpd4 | ectonucleoside triphosphate diphosphohydrolase 4 (Entpd4) | -1.51 |
| Trim26 | tripartite motif-containing 26 (Trim26), transcript variant 1 | -1.51 |
| Mrps33 | mitochondrial ribosomal protein S33 (Mrps33), nuclear gene encoding mitochondrial protein, transcript variant 2 | -1.51 |
| Polr2h | polymerase (RNA) II (DNA directed) polypeptide H (Polr2h) | -1.51 |
| Sfrs6 | splicing factor, arginine/serine-rich 6 (Sfrs6) | -1.51 |
| Rpl22 | ribosomal protein L22 (Rpl22) | -1.52 |
| Ifi47 | interferon gamma inducible protein 47 (Ifi47) | -1.52 |
| Elf1 | E74-like factor 1 (Elf1) | -1.52 |
| LOC100041504 | similar to beta chemokine Exodus-2 (LOC100041504) | -1.53 |
| Nol6 | nucleolar protein family 6 (RNA-associated) (Nol6) | -1.53 |
| Arhgef1 | Rho guanine nucleotide exchange factor (GEF) 1 (Arhgef1) | -1.53 |
| M6pr | mannose-6-phosphate receptor, cation dependent (M6pr) | -1.53 |
| Atp2a3 | ATPase, Ca++ transporting, ubiquitous (Atp2a3) | -1.53 |
| Mrpl24 | mitochondrial ribosomal protein L24 (Mrpl24) | -1.53 |
| Slc6a6 | solute carrier family 6 (neurotransmitter transporter, taurine), member 6 (Slc6a6) | -1.53 |
| Wdr51b | WD repeat domain 51B (Wdr51b) | -1.53 |
| Vps54 | vacuolar protein sorting 54 (yeast) (Vps54) | -1.54 |
| Arf2 | ADP-ribosylation factor 2 (Arf2) | -1.54 |
| 2410025L10Rik | RIKEN cDNA 2410025L10 gene (2410025L10Rik) | -1.54 |
| Znrd1 | zinc ribbon domain containing, 1 (Znrd1) | -1.54 |
| Trappc4 | trafficking protein particle complex 4 (Trappc4) | -1.54 |
| Rgl2 | ral guanine nucleotide dissociation stimulator-like 2 (Rgl2) | -1.54 |
| Pip4k2b | phosphatidylinositol-5-phosphate 4-kinase, type II, beta (Pip4k2b) | -1.54 |
| Panx1 | pannexin 1 (Panx1) | -1.55 |
| Atp5sl | ATP5S-like (Atp5sl) | -1.55 |
| Dgka | diacylglycerol kinase, alpha (Dgka) | -1.55 |
| Tpd52 | tumor protein D52 (Tpd52), transcript variant 5 | -1.55 |
| Mapre2 | microtubule-associated protein, RP/EB family, member 2 (Mapre2) | -1.56 |
| H2-T23 | histocompatibility 2, T region locus 23 (H2-T23) | -1.56 |
| LOC100045300 | similar to Dr1 associated protein 1 (negative cofactor 2 alpha) (LOC100045300) | -1.56 |
| Prpf38b | PRP38 pre-mRNA processing factor 38 (yeast) domain containing B (Prpf38b) | -1.56 |
| D10Ertd641e | DNA segment, Chr 10, ERATO Doi 641, expressed (D10Ertd641e) | -1.57 |
| Myo9b | myosin IXb (Myo9b) | -1.57 |
| Il10ra | interleukin 10 receptor, alpha (Il10ra) | -1.57 |
| Zmym3 | zinc finger, MYM-type 3 (Zmym3) | -1.57 |
| Nisch | nischarin (Nisch) | -1.57 |
| Snapc3 | small nuclear RNA activating complex, polypeptide 3 (Snapc3) | -1.57 |
| Wdr68 | WD repeat domain 68 (Wdr68) | -1.57 |
| Etfa | electron transferring flavoprotein, alpha polypeptide (Etfa), nuclear gene encoding mitochondrial protein | -1.57 |
| Manba | mannosidase, beta A, lysosomal (Manba) | -1.57 |
| Slc22a5 | solute carrier family 22 (organic cation transporter), member 5 (Slc22a5) | -1.58 |
| Suz12 | suppressor of zeste 12 homolog (Drosophila) (Suz12) | -1.58 |
| Rab2a | RAB2A, member RAS oncogene family (Rab2a) | -1.58 |
| Foxj2 | forkhead box J2 (Foxj2) | -1.58 |
| Nras | neuroblastoma ras oncogene (Nras) | -1.58 |
| Dpp4 | dipeptidylpeptidase 4 (Dpp4) | -1.59 |
| LOC100046996 | similar to farnesyltransferase alpha subunit (LOC100046996) | -1.59 |
| Wdsof1 | WD repeats and SOF domain containing 1 (Wdsof1) | -1.59 |
| Zc3h7a | zinc finger CCCH type containing 7 A (Zc3h7a) | -1.59 |
| Add3 | adducin 3 (gamma) (Add3) | -1.59 |
| Vapa | vesicle-associated membrane protein, associated protein A (Vapa) | -1.59 |
| Soat1 | sterol O-acyltransferase 1 (Soat1) | -1.59 |
| LOC100044776 | similar to Enhancer of polycomb homolog 2, transcript variant 1 (LOC100044776) | -1.6 |
| Araf | v-raf murine sarcoma 3611 viral oncogene homolog (Araf) | -1.6 |
| Dusp1 | dual specificity phosphatase 1 (Dusp1) | -1.6 |
| Tnfrsf13c | tumor necrosis factor receptor superfamily, member 13c (Tnfrsf13c) | -1.6 |
| LOC100047167 | similar to mKIAA0990 protein (LOC100047167) | -1.61 |
| Dennd3 | DENN/MADD domain containing 3 (Dennd3) | -1.61 |
| Ikzf1 | IKAROS family zinc finger 1 (Ikzf1), transcript variant 1 | -1.61 |
| Smc1a | structural maintenance of chromosomes 1A (Smc1a) | -1.61 |
| Dpysl2 | dihydropyrimidinase-like 2 (Dpysl2) | -1.61 |
| BC021381 | cDNA sequence BC021381 (BC021381) | -1.61 |
| Ttc3 | tetratricopeptide repeat domain 3 (Ttc3) | -1.61 |
| Per2 | period homolog 2 (Drosophila) (Per2) | -1.61 |
| Mrps7 | mitchondrial ribosomal protein S7 (Mrps7) | -1.61 |
| AI451557 | expressed sequence AI451557 (AI451557) | -1.61 |
| LOC621823 | similar to Proteasome activator complex subunit 2 (Proteasome activator 28-beta subunit) (PA28beta) (PA28b) | -1.61 |
| Scotin | scotin gene (Scotin), transcript variant 1 | -1.62 |
| Zcchc18 | zinc finger, CCHC domain containing 18 (Zcchc18), transcript variant 3 | -1.62 |
| Eml4 | echinoderm microtubule associated protein like 4 (Eml4) | -1.62 |
| Fcrla | Fc receptor-like A (Fcrla) | -1.62 |
| BC013529 | cDNA sequence BC013529 (BC013529) | -1.62 |
| Serpina3n | serine (or cysteine) peptidase inhibitor, clade A, member 3N (Serpina3n) | -1.62 |
| Pex19 | peroxisome biogenesis factor 19 (Pex19) | -1.62 |
| Cd6 | CD6 antigen (Cd6), transcript variant 2 | -1.63 |
| Cybasc3 | cytochrome b, ascorbate dependent 3 (Cybasc3) | -1.63 |
| Elp2 | elongation protein 2 homolog (S. cerevisiae) (Elp2) | -1.63 |
| LOC100047369 | similar to 0610007P22Rik protein (LOC100047369), misc RNA. | -1.63 |
| Adrb2 | adrenergic receptor, beta 2 (Adrb2) | -1.63 |
| Ccdc88b | coiled-coil domain containing 88B (Ccdc88b) | -1.64 |
| Lpxn | leupaxin (Lpxn) | -1.64 |
| Gnptg | N-acetylglucosamine-1-phosphotransferase, gamma subunit (Gnptg) | -1.64 |
| Sfrs5 | splicing factor, arginine/serine-rich 5 (SRp40, HRS) (Sfrs5), transcript variant 1 | -1.64 |
| Psmd4 | proteasome (prosome, macropain) 26S subunit, non-ATPase, 4 (Psmd4) | -1.64 |
| Dusp11 | dual specificity phosphatase 11 (RNA/RNP complex 1-interacting) (Dusp11) | -1.65 |
| Pdlim4 | PDZ and LIM domain 4 (Pdlim4) | -1.65 |
| Zfp36 | zinc finger protein 36 (Zfp36) | -1.65 |
| LOC100044538 | similar to immunity-associated nucleotide 4 (LOC100044538), misc RNA. | -1.65 |
| Cdk5rap3 | CDK5 regulatory subunit associated protein 3 (Cdk5rap3) | -1.65 |
| Cd83 | CD83 antigen (Cd83) | -1.65 |
| Slc35b3 | solute carrier family 35, member B3 (Slc35b3) | -1.66 |
| Sh3bgrl3 | SH3 domain binding glutamic acid-rich protein-like 3 (Sh3bgrl3) | -1.66 |
| Kcnab2 | potassium voltage-gated channel, shaker-related subfamily, beta member 2 (Kcnab2) | -1.66 |
| Arl1 | ADP-ribosylation factor-like 1 (Arl1) | -1.66 |
| LOC100046891 | similar to Smad5 (LOC100046891), misc RNA. | -1.67 |
| Trip12 | thyroid hormone receptor interactor 12 (Trip12) | -1.67 |
| Pih1d1 | PIH1 domain containing 1 (Pih1d1) | -1.67 |
| Snn | stannin (Snn) | -1.67 |
| Med23 | mediator complex subunit 23 (Med23) | -1.67 |
| Fcgr4 | Fc receptor, IgG, low affinity IV (Fcgr4) | -1.68 |
| Hn1 | hematological and neurological expressed sequence 1 (Hn1) | -1.68 |
| Txndc15 | thioredoxin domain containing 15 (Txndc15) | -1.68 |
| Mdm2 | transformed mouse 3T3 cell double minute 2 (Mdm2) | -1.68 |
| Myo9b | myosin IXb (Myo9b) | -1.68 |
| Cdc2l6 | cell division cycle 2-like 6 (CDK8-like) (Cdc2l6) | -1.69 |
| Samhd1 | SAM domain and HD domain, 1 (Samhd1) | -1.69 |
| Pecam1 | platelet/endothelial cell adhesion molecule 1 (Pecam1), transcript variant 2 | -1.69 |
| Npc2 | Niemann Pick type C2 (Npc2) | -1.69 |
| Skiv2l | superkiller viralicidic activity 2-like (S. cerevisiae) (Skiv2l) | -1.69 |
| Cugbp2 | CUG triplet repeat, RNA binding protein 2 (Cugbp2), transcript variant 6 | -1.69 |
| LOC100048105 | similar to Ubc protein, transcript variant 1 (LOC100048105) | -1.69 |
| Pop5 | processing of precursor 5, ribonuclease P/MRP family (S. cerevisiae) (Pop5) | -1.7 |
| Mycl1 | v-myc myelocytomatosis viral oncogene homolog 1, lung carcinoma derived (avian) (Mycl1) | -1.7 |
| Mib2 | mindbomb homolog 2 (Drosophila) (Mib2) | -1.7 |
| Ctnnb1 | catenin (cadherin associated protein), beta 1 (Ctnnb1) | -1.7 |
| D19Wsu162e | DNA segment, Chr 19, Wayne State University 162, expressed (D19Wsu162e) | -1.7 |
| Pigx | phosphatidylinositol glycan anchor biosynthesis, class X (Pigx) | -1.7 |
| Kctd2 | potassium channel tetramerisation domain containing 2 (Kctd2) | -1.7 |
| Drbp1 | RNA binding motif protein 45 (Rbm45) | -1.7 |
| Kns2 | kinesin 2 (Kns2), transcript variant d | -1.7 |
| Ercc5 | excision repair cross-complementing rodent repair deficiency, complementation group 5 (Ercc5) | -1.71 |
| Csnk1g2 | casein kinase 1, gamma 2 (Csnk1g2) | -1.71 |
| Il4i1 | interleukin 4 induced 1 (Il4i1) | -1.71 |
| Anxa11 | annexin A11 (Anxa11) | -1.72 |
| LOC100045887 | similar to PTB-associated splicing factor (LOC100045887) | -1.72 |
| H2-Ob | histocompatibility 2, O region beta locus (H2-Ob) | -1.72 |
| Peli1 | pellino 1 (Peli1) | -1.72 |
| Smek2 | SMEK homolog 2, suppressor of mek1 (Dictyostelium) (Smek2) | -1.72 |
| H2-DMb1 | histocompatibility 2, class II, locus Mb1 (H2-DMb1) | -1.72 |
| Nfyc | nuclear transcription factor-Y gamma (Nfyc), transcript variant 1 | -1.72 |
| F2r | coagulation factor II (thrombin) receptor (F2r) | -1.72 |
| Dkk3 | dickkopf homolog 3 (Xenopus laevis) (Dkk3) | -1.72 |
| Ptges3 | prostaglandin E synthase 3 (cytosolic) (Ptges3) | -1.73 |
| Hcst | hematopoietic cell signal transducer (Hcst) | -1.73 |
| BC087945 | cDNA sequence BC087945 (BC087945) | -1.73 |
| Tmem184b | transmembrane protein 184b (Tmem184b) | -1.73 |
| Asb8 | ankyrin repeat and SOCS box-containing protein 8 (Asb8) | -1.73 |
| Stx12 | syntaxin 12 (Stx12) | -1.73 |
| Morc3 | microrchidia 3 (Morc3) | -1.74 |
| Pgs1 | phosphatidylglycerophosphate synthase 1 (Pgs1) | -1.74 |
| LOC100048020 | similar to RIKEN cDNA 0610009K11 gene (LOC100048020) | -1.74 |
| Gimap1 | GTPase, IMAP family member 1 (Gimap1), transcript variant 1 | -1.75 |
| BC031353 | cDNA sequence BC031353 (BC031353) | -1.75 |
| Zfp608 | zinc finger protein 608 (Zfp608) | -1.75 |
| Rab33b | RAB33B, member of RAS oncogene family (Rab33b) | -1.75 |
| Gsdmdc1 | gasdermin domain containing 1 (Gsdmdc1) | -1.76 |
| Rnf103 | ring finger protein 103 (Rnf103) | -1.76 |
| Traf1 | Tnf receptor-associated factor 1 (Traf1) | -1.76 |
| Cog3 | component of oligomeric golgi complex 3 (Cog3) | -1.77 |
| Hmgcs2 | 3-hydroxy-3-methylglutaryl-Coenzyme A synthase 2 (Hmgcs2), nuclear gene encoding mitochondrial protein | -1.77 |
| Bcl6 | B-cell leukemia/lymphoma 6 (Bcl6) | -1.77 |
| Hgsnat | heparan-alpha-glucosaminide N-acetyltransferase (Hgsnat) | -1.77 |
| Tspan32 | tetraspanin 32 (Tspan32) | -1.77 |
| Rbak | RB-associated KRAB repressor (Rbak), transcript variant 1 | -1.77 |
| Mum1 | melanoma associated antigen (mutated) 1 (Mum1) | -1.77 |
| Klhl6 | kelch-like 6 (Drosophila) (Klhl6) | -1.78 |
| Inpp5d | inositol polyphosphate-5-phosphatase D (Inpp5d) | -1.78 |
| Stx5a | syntaxin 5A (Stx5a) | -1.78 |
| Smek2 | SMEK homolog 2, suppressor of mek1 (Dictyostelium) (Smek2) | -1.78 |
| Emg1 | EMG1 nucleolar protein homolog (S. cerevisiae) (Emg1) | -1.79 |
| Slc25a19 | solute carrier family 25 (mitochondrial thiamine pyrophosphate carrier), member 19 (Slc25a19), nuclear gene encoding mitochondrial protein | -1.79 |
| Trip12 | thyroid hormone receptor interactor 12 (Trip12) | -1.79 |
| Zap70 | zeta-chain (TCR) associated protein kinase (Zap70) | -1.79 |
| Pstpip1 | proline-serine-threonine phosphatase-interacting protein 1 (Pstpip1) | -1.8 |
| LOC100047834 | similar to translin associated protein X (LOC100047834) | -1.8 |
| Spsb3 | splA/ryanodine receptor domain and SOCS box containing 3 (Spsb3) | -1.8 |
| Ccdc85b | coiled-coil domain containing 85B (Ccdc85b) | -1.8 |
| Traf6 | Tnf receptor-associated factor 6 (Traf6) | -1.8 |
| Ddx21 | DEAD (Asp-Glu-Ala-Asp) box polypeptide 21 (Ddx21) | -1.81 |
| Limd1 | LIM domains containing 1 (Limd1) | -1.81 |
| Slc35b3 | solute carrier family 35, member B3 (Slc35b3) | -1.82 |
| Tusc4 | tumor suppressor candidate 4 (Tusc4) | -1.82 |
| Cwf19l2 | CWF19-like 2, cell cycle control (S. pombe) (Cwf19l2) | -1.82 |
| Cbfb | core binding factor beta (Cbfb) | -1.82 |
| Nxf1 | nuclear RNA export factor 1 homolog (S. cerevisiae) (Nxf1) | -1.82 |
| Slain2 | SLAIN motif family, member 2 (Slain2) | -1.83 |
| Cdc42se1 | CDC42 small effector 1 (Cdc42se1), transcript variant 1 | -1.83 |
| Tctex1d2 | Tctex1 domain containing 2 (Tctex1d2) | -1.84 |
| Ptp4a2 | protein tyrosine phosphatase 4a2 (Ptp4a2) | -1.84 |
| 2310039H08Rik | RIKEN cDNA 2310039H08 gene (2310039H08Rik) | -1.85 |
| Skap2 | src family associated phosphoprotein 2 (Skap2) | -1.85 |
| Elavl1 | ELAV (embryonic lethal, abnormal vision, Drosophila)-like 1 (Hu antigen R) (Elavl1) | -1.85 |
| Zbtb33 | zinc finger and BTB domain containing 33 (Zbtb33), transcript variant 2 | -1.85 |
| Elovl5 | ELOVL family member 5, elongation of long chain fatty acids (yeast) (Elovl5) | -1.85 |
| BC057552 | cDNA sequence BC057552 (BC057552) | -1.85 |
| Yeats4 | YEATS domain containing 4 (Yeats4) | -1.85 |
| Fubp3 | far upstream element (FUSE) binding protein 3 (Fubp3) | -1.85 |
| Slc4a2 | solute carrier family 4 (anion exchanger), member 2 (Slc4a2) | -1.86 |
| Rasgrp1 | RAS guanyl releasing protein 1 (Rasgrp1) | -1.86 |
| 2410002F23Rik | RIKEN cDNA 2410002F23 gene (2410002F23Rik) | -1.86 |
| 4121402D02Rik | RIKEN cDNA 4121402D02 gene (4121402D02Rik) | -1.86 |
| Herpud1 | homocysteine-inducible, endoplasmic reticulum stress-inducible, ubiquitin-like domain member 1 (Herpud1) | -1.86 |
| Rcsd1 | RCSD domain containing 1 (Rcsd1), transcript variant 1 | -1.87 |
| Tmed9 | transmembrane emp24 protein transport domain containing 9 (Tmed9) | -1.87 |
| Rbm5 | RNA binding motif protein 5 (Rbm5) | -1.88 |
| 0610010E21Rik | RIKEN cDNA 0610010E21 gene (0610010E21Rik) | -1.88 |
| Gorasp2 | golgi reassembly stacking protein 2 (Gorasp2) | -1.88 |
| Ednrb | endothelin receptor type B (Ednrb) | -1.88 |
| Vti1b | vesicle transport through interaction with t-SNAREs 1B homolog (Vti1b) | -1.88 |
| 2500003M10Rik | RIKEN cDNA 2500003M10 gene (2500003M10Rik) | -1.88 |
| Crlf3 | cytokine receptor-like factor 3 (Crlf3) | -1.88 |
| Zc3h12d | zinc finger CCCH type containing 12D (Zc3h12d) | -1.89 |
| Atf7ip | activating transcription factor 7 interacting protein (Atf7ip) | -1.89 |
| Gmfg | glia maturation factor, gamma (Gmfg), transcript variant 1 | -1.89 |
| Ccdc100 | coiled-coil domain containing 100 (Ccdc100) | -1.9 |
| Vav1 | vav 1 oncogene (Vav1) | -1.9 |
| Sin3a | transcriptional regulator, SIN3A (yeast) (Sin3a) | -1.9 |
| Glt25d1 | glycosyltransferase 25 domain containing 1 (Glt25d1) | -1.9 |
| Fcrla | Fc receptor-like A (Fcrla) | -1.91 |
| Ctcf | CCCTC-binding factor (Ctcf) | -1.91 |
| Sult1a1 | sulfotransferase family 1A, phenol-preferring, member 1 (Sult1a1) | -1.92 |
| Uspl1 | ubiquitin specific peptidase like 1 (Uspl1) | -1.92 |
| Add3 | adducin 3 (gamma) (Add3) | -1.93 |
| Vamp4 | vesicle-associated membrane protein 4 (Vamp4) | -1.93 |
| Sla | src-like adaptor (Sla), transcript variant 2 | -1.93 |
| Zfp292 | zinc finger protein 292, transcript variant 4 (Zfp292) | -1.93 |
| Hexb | hexosaminidase B (Hexb) | -1.94 |
| Mapk11 | mitogen-activated protein kinase 11 (Mapk11) | -1.94 |
| Tram1 | translocating chain-associating membrane protein 1 (Tram1) | -1.95 |
| Cbfb | core binding factor beta (Cbfb) | -1.95 |
| Mreg | melanoregulin (Mreg) | -1.95 |
| Polr2g | polymerase (RNA) II (DNA directed) polypeptide G (Polr2g) | -1.95 |
| Sidt1 | SID1 transmembrane family, member 1 (Sidt1) | -1.96 |
| 1700047I17Rik1 | RIKEN cDNA 1700047I17 gene 1 (1700047I17Rik1) | -1.96 |
| Acss1 | acyl-CoA synthetase short-chain family member 1 (Acss1), nuclear gene encoding mitochondrial protein | -1.96 |
| Cd6 | CD6 antigen (Cd6), transcript variant 2 | -1.96 |
| Pkm2 | pyruvate kinase, muscle (Pkm2) | -1.96 |
| Pdgfra | platelet derived growth factor receptor, alpha polypeptide (Pdgfra), transcript variant 1 | -1.96 |
| Irf1 | interferon regulatory factor 1 (Irf1) | -1.97 |
| Zfp512 | zinc finger protein 512 (Zfp512) | -1.97 |
| 1500001L15Rik | neuroguidin, EIF4E binding protein (Ngdn) | -1.97 |
| LOC100047963 | similar to ADIR1 (LOC100047963) | -1.97 |
| Gnptg | N-acetylglucosamine-1-phosphotransferase, gamma subunit (Gnptg) | -1.97 |
| Klk8 | kallikrein related-peptidase 8 (Klk8) | -1.98 |
| Ate1 | arginine-tRNA-protein transferase 1 (Ate1), transcript variant 1 | -1.99 |
| Gimap6 | GTPase, IMAP family member 6 (Gimap6) | -1.99 |
| Arglu1 | arginine and glutamate rich 1 (Arglu1) | -1.99 |
| Akap8l | A kinase (PRKA) anchor protein 8-like (Akap8l) | -1.99 |
| Errfi1 | ERBB receptor feedback inhibitor 1 (Errfi1) | -1.99 |
| Atp2a3 | ATPase, Ca++ transporting, ubiquitous (Atp2a3) | -1.99 |
| B230342M21Rik | RIKEN cDNA B230342M21 gene (B230342M21Rik) | -2 |
| Stt3b | STT3, subunit of the oligosaccharyltransferase complex, homolog B (S. cerevisiae) (Stt3b) | -2 |
| Ptp4a2 | protein tyrosine phosphatase 4a2 (Ptp4a2) | -2 |
| Cyp4f13 | cytochrome P450, family 4, subfamily f, polypeptide 13 (Cyp4f13) | -2.01 |
| BC037034 | cDNA sequence BC037034 (BC037034) | -2.01 |
| Mettl3 | methyltransferase-like 3 (Mettl3) | -2.02 |
| Zfp597 | zinc finger protein 597 (Zfp597) | -2.02 |
| Stk4 | serine/threonine kinase 4 (Stk4) | -2.02 |
| Hap1 | huntingtin-associated protein 1 (Hap1) | -2.03 |
| Tigd2 | tigger transposable element derived 2 (Tigd2) | -2.03 |
| Rassf3 | Ras association (RalGDS/AF-6) domain family member 3 (Rassf3) | -2.03 |
| Snx2 | sorting nexin 2 (Snx2) | -2.03 |
| Dkk3 | dickkopf homolog 3 (Xenopus laevis) (Dkk3) | -2.03 |
| A230050P20Rik | RIKEN cDNA A230050P20 gene (A230050P20Rik) | -2.04 |
| Rasa1 | RAS p21 protein activator 1 (Rasa1) | -2.04 |
| Spop | speckle-type POZ protein (Spop) | -2.04 |
| 2410002F23Rik | RIKEN cDNA 2410002F23 gene (2410002F23Rik) | -2.04 |
| Rabgap1 | RAB GTPase activating protein 1 (Rabgap1), transcript variant 1 | -2.05 |
| Snx8 | sorting nexin 8 (Snx8) | -2.05 |
| Mfng | MFNG O-fucosylpeptide 3-beta-N-acetylglucosaminyltransferase (Mfng) | -2.05 |
| Lamp2 | lysosomal-associated membrane protein 2 (Lamp2) | -2.05 |
| Sf4 | splicing factor 4 (Sf4) | -2.06 |
| Cstf2 | cleavage stimulation factor, 3' pre-RNA subunit 2 (Cstf2) | -2.06 |
| Cd6 | CD6 antigen (Cd6), transcript variant 2 | -2.06 |
| D6Wsu176e | family with sequence similarity 3, member C (FAm3c) | -2.07 |
| Iqgap1 | IQ motif containing GTPase activating protein 1 (Iqgap1) | -2.07 |
| Gcc2 | GRIP and coiled-coil domain containing 2 (Gcc2) | -2.07 |
| Lsm14a | LSM14 homolog A (SCD6, S. cerevisiae) (Lsm14a) | -2.08 |
| Lamp2 | lysosomal-associated membrane protein 2 (Lamp2), transcript variant 2 | -2.08 |
| Sirpa | signal-regulatory protein alpha (Sirpa) | -2.08 |
| LOC100046120 | similar to clusterin (LOC100046120) | -2.08 |
| Mgst2 | microsomal glutathione S-transferase 2 (Mgst2) | -2.09 |
| Gmfg | glia maturation factor, gamma (Gmfg), transcript variant 1 | -2.09 |
| Myh9 | myosin, heavy polypeptide 9, non-muscle (Myh9) | -2.09 |
| Plekha2 | pleckstrin homology domain-containing, family A (phosphoinositide binding specific) member 2 (Plekha2) | -2.09 |
| Btbd1 | BTB (POZ) domain containing 1 (Btbd1) | -2.1 |
| Tnrc6a | trinucleotide repeat containing 6a (Tnrc6a) | -2.1 |
| 4933439C20Rik | RIKEN cDNA 4933439C20 gene (4933439C20Rik) | -2.11 |
| Abi1 | abl-interactor 1 (Abi1), transcript variant 1 | -2.11 |
| Ttc3 | tetratricopeptide repeat domain 3 (Ttc3) | -2.11 |
| Cbx7 | chromobox homolog 7 (Cbx7) | -2.12 |
| Zcchc8 | zinc finger, CCHC domain containing 8 (Zcchc8) | -2.12 |
| Slc44a2 | solute carrier family 44, member 2 (Slc44a2) | -2.12 |
| Pde1b | phosphodiesterase 1B, Ca2+-calmodulin dependent (Pde1b) | -2.13 |
| Slco2b1 | solute carrier organic anion transporter family, member 2b1 (Slco2b1) | -2.13 |
| Suv420h1 | suppressor of variegation 4-20 homolog 1 (Drosophila) (Suv420h1) | -2.13 |
| Fyb | FYN binding protein (Fyb) | -2.13 |
| Arrdc3 | arrestin domain containing 3 (Arrdc3) | -2.13 |
| Mfge8 | milk fat globule-EGF factor 8 protein (Mfge8) | -2.13 |
| Bbc3 | Bcl-2 binding component 3 (Bbc3) | -2.13 |
| Gpr171 | G protein-coupled receptor 171 (Gpr171) | -2.13 |
| Ubl3 | ubiquitin-like 3 (Ubl3) | -2.13 |
| Iap | CD47 antigen (Rh-related antigen, integrin-associated signal transducer) (Iap) | -2.14 |
| A430107D22Rik | RIKEN cDNA A430107D22 gene (A430107D22Rik) | -2.14 |
| Bat2 | HLA-B associated transcript 2 (Bat2) | -2.14 |
| Lysmd1 | LysM, putative peptidoglycan-binding, domain containing 1 (Lysmd1) | -2.15 |
| Plrg1 | pleiotropic regulator 1, PRL1 homolog (Arabidopsis) (Plrg1) | -2.15 |
| Nuak2 | NUAK family, SNF1-like kinase, 2 (Nuak2) | -2.15 |
| Arhgap4 | Rho GTPase activating protein 4 (Arhgap4) | -2.15 |
| Cd44 | CD44 antigen (Cd44), transcript variant 2 | -2.15 |
| Clec2d | C-type lectin domain family 2, member d (Clec2d) | -2.16 |
| 1110003E01Rik | RIKEN cDNA 1110003E01 gene (1110003E01Rik) | -2.16 |
| Tk2 | thymidine kinase 2, mitochondrial (Tk2), nuclear gene encoding mitochondrial protein | -2.16 |
| Spag9 | sperm associated antigen 9 (Spag9), transcript variant 4 | -2.16 |
| Trit1 | tRNA isopentenyltransferase 1 (Trit1) | -2.17 |
| Csnk1g3 | casein kinase 1, gamma 3 (Csnk1g3) | -2.18 |
| 4921505C17Rik | RIKEN cDNA 4921505C17 gene (4921505C17Rik) | -2.18 |
| LOC676420 | similar to ceramide kinases (LOC676420), misc RNA. | -2.19 |
| Zfp263 | zinc finger protein 263 (Zfp263) | -2.19 |
| Evl | Ena-vasodilator stimulated phosphoprotein (Evl) | -2.19 |
| Rab3gap1 | RAB3 GTPase activating protein subunit 1 (Rab3gap1) | -2.19 |
| Mark3 | MAP/microtubule affinity regulating kinase 3 (Mark3) | -2.2 |
| LOC100044439 | similar to cytochrome P450 CYP4F18 (LOC100044439) | -2.21 |
| Jakmip1 | janus kinase and microtubule interacting protein 1 (Jakmip1) | -2.22 |
| Atp1b1 | ATPase, Na+/K+ transporting, beta 1 polypeptide (Atp1b1) | -2.22 |
| Gdi1 | guanosine diphosphate (GDP) dissociation inhibitor 1 (Gdi1) | -2.23 |
| Med23 | mediator complex subunit 23 (Med23) | -2.23 |
| Atp1b3 | ATPase, Na+/K+ transporting, beta 3 polypeptide (Atp1b3) | -2.23 |
| Wsb1 | WD repeat and SOCS box-containing 1 (Wsb1), transcript variant 2 | -2.23 |
| Brd2 | bromodomain containing 2 (Brd2), transcript variant 1 | -2.24 |
| Ythdf2 | YTH domain family 2 (Ythdf2) | -2.24 |
| Gtf2h1 | general transcription factor II H, polypeptide 1 (Gtf2h1) | -2.24 |
| Aftph | aftiphilin (Aftph) | -2.25 |
| 8430432M10Rik | phosphopantothenoylcysteine decarboxylase (Ppcdc) | -2.25 |
| D6Wsu176e | DNA segment, Chr 6, Wayne State University 176, expressed (D6Wsu176e) | -2.26 |
| 2400003C14Rik | RIKEN cDNA 2400003C14 gene (2400003C14Rik) | -2.26 |
| Prkar1a | protein kinase, cAMP dependent regulatory, type I, alpha (Prkar1a) | -2.27 |
| AI314180 | expressed sequence AI314180 (AI314180) | -2.27 |
| Wdr1 | WD repeat domain 1 (Wdr1) | -2.28 |
| Ap3m2 | adaptor-related protein complex 3, mu 2 subunit (Ap3m2) | -2.28 |
| Ing4 | inhibitor of growth family, member 4 (Ing4) | -2.28 |
| Il16 | interleukin 16 (Il16) | -2.28 |
| Rpl36a | ribosomal protein L36a (Rpl36a) | -2.29 |
| Plekhm1 | pleckstrin homology domain containing, family M (with RUN domain) member 1 (Plekhm1) | -2.29 |
| Tagap | T-cell activation Rho GTPase-activating protein (Tagap) | -2.3 |
| Fchsd2 | FCH and double SH3 domains 2 (Fchsd2) | -2.3 |
| Smarca2 | SWI/SNF related, matrix associated, actin dependent regulator of chromatin, subfamily a, member 2 (Smarca2), transcript variant 1 | -2.31 |
| Lat2 | linker for activation of T cells family, member 2 (Lat2), transcript variant 2 | -2.31 |
| Il11ra1 | interleukin 11 receptor, alpha chain 1 (Il11ra1) | -2.33 |
| Hnrpm | heterogeneous nuclear ribonucleoprotein M (Hnrpm) | -2.34 |
| Cyp4f18 | cytochrome P450, family 4, subfamily f, polypeptide 18 (Cyp4f18) | -2.34 |
| Tap2 | transporter 2, ATP-binding cassette, sub-family B (MDR/TAP) (Tap2) | -2.34 |
| Scd1 | stearoyl-Coenzyme A desaturase 1 (Scd1) | -2.34 |
| Ing4 | inhibitor of growth family, member 4 (Ing4) | -2.35 |
| Swap70 | SWA-70 protein (Swap70) | -2.35 |
| Cyld | cylindromatosis (turban tumor syndrome) (Cyld) | -2.36 |
| Gns | glucosamine (N-acetyl)-6-sulfatase (Gns) | -2.36 |
| Cyp1b1 | cytochrome P450, family 1, subfamily b, polypeptide 1 (Cyp1b1) | -2.37 |
| LOC100048845 | similar to CD28 antigen (LOC100048845) | -2.39 |
| Armc10 | armadillo repeat containing 10 (Armc10) | -2.4 |
| Ctsw | cathepsin W (Ctsw) | -2.4 |
| Bclaf1 | BCL2-associated transcription factor 1 (Bclaf1), transcript variant 3 | -2.41 |
| D230007K08Rik | RIKEN cDNA D230007K08 gene, transcript variant 5 (D230007K08Rik) | -2.41 |
| Snx17 | sorting nexin 17 (Snx17) | -2.41 |
| Ppp3ca | protein phosphatase 3, catalytic subunit, alpha isoform (Ppp3ca) | -2.42 |
| Dennd1c | DENN/MADD domain containing 1C (Dennd1c) | -2.42 |
| Ccdc130 | coiled-coil domain containing 130 (Ccdc130) | -2.42 |
| 2700060E02Rik | RIKEN cDNA 2700060E02 gene (2700060E02Rik) | -2.43 |
| Oasl2 | 2'-5' oligoadenylate synthetase-like 2 (Oasl2) | -2.44 |
| Fgfr1op2 | FGFR1 oncogene partner 2 (Fgfr1op2) | -2.45 |
| Per1 | period homolog 1 (Drosophila) (Per1) | -2.45 |
| Sell | selectin, lymphocyte (Sell) | -2.45 |
| Mylc2b | myosin light chain, regulatory B (Mylc2b) | -2.45 |
| Klf2 | Kruppel-like factor 2 (lung) (Klf2) | -2.45 |
| EG434197 | predicted gene, EG434197 (EG434197) | -2.46 |
| Bhlhb2 | basic helix-loop-helix domain containing, class B2 (Bhlhb2) | -2.46 |
| H2-Ke6 | H2-K region expressed gene 6 (H2-Ke6) | -2.46 |
| LOC100047261 | similar to spermidine/spermine N1-acetyltransferase (LOC100047261), misc RNA. | -2.46 |
| St6gal1 | beta galactoside alpha 2,6 sialyltransferase 1 (St6gal1) | -2.47 |
| Ets1 | E26 avian leukemia oncogene 1, 5' domain (Ets1), transcript variant 2 | -2.48 |
| Cecr5 | cat eye syndrome chromosome region, candidate 5 homolog (human) (Cecr5) | -2.48 |
| Ss18 | synovial sarcoma translocation, Chromosome 18 (Ss18) | -2.48 |
| Ahnak | AHNAK nucleoprotein (desmoyokin) (Ahnak), transcript variant 1 | -2.48 |
| Sdc4 | syndecan 4 (Sdc4) | -2.49 |
| Hnrpm | heterogeneous nuclear ribonucleoprotein M (Hnrpm) | -2.49 |
| Epsti1 | epithelial stromal interaction 1 (breast) (Epsti1), transcript variant a | -2.49 |
| Rgs1 | regulator of G-protein signaling 1 (Rgs1) | -2.51 |
| Sbk | SH3-binding kinase 1 (Sbk) | -2.51 |
| Herc4 | hect domain and RLD 4 (Herc4) | -2.51 |
| Ldb1 | LIM domain binding 1 (Ldb1), transcript variant 3 | -2.52 |
| Rap2c | RAP2C, member of RAS oncogene family (Rap2c) | -2.53 |
| Ugcg | UDP-glucose ceramide glucosyltransferase (Ugcg) | -2.53 |
| Bcl9l | B cell CLL/lymphoma 9-like (Bcl9l) | -2.55 |
| Txnip | thioredoxin interacting protein (Txnip), transcript variant 1 | -2.56 |
| LOC545056 | ubiquitin-conjugating enzyme E2, J2 homolog pseudogene (LOC545056) on chromosome 14. | -2.57 |
| Foxj3 | forkhead box J3 (Foxj3) | -2.57 |
| Btla | B and T lymphocyte associated (Btla), transcript variant 2 | -2.57 |
| Mterf | mitochondrial transcription termination factor (Mterf), nuclear gene encoding mitochondrial protein, transcript variant 2 | -2.57 |
| Sec11c | SEC11 homolog C (S. cerevisiae) (Sec11c) | -2.58 |
| Rps25 | ribosomal protein S25 (Rps25) | -2.59 |
| Mcl1 | myeloid cell leukemia sequence 1 (Mcl1) | -2.59 |
| Clk4 | CDC like kinase 4 (Clk4) | -2.59 |
| 2610207I05Rik | RIKEN cDNA 2610207I05 gene (2610207I05Rik) | -2.6 |
| Purb | purine rich element binding protein B (Purb) | -2.6 |
| Nt5e | 5' nucleotidase, ecto (Nt5e) | -2.62 |
| Swap70 | SWA-70 protein (Swap70) | -2.62 |
| Nfkbid | nuclear factor of kappa light polypeptide gene enhancer in B-cells inhibitor, delta (Nfkbid) | -2.62 |
| Zap70 | zeta-chain (TCR) associated protein kinase (Zap70) | -2.62 |
| Tmem50a | transmembrane protein 50A (Tmem50a) | -2.63 |
| Trp53inp1 | transformation related protein 53 inducible nuclear protein 1 (Trp53inp1) | -2.64 |
| Gprasp1 | G protein-coupled receptor associated sorting protein 1 (Gprasp1), transcript variant 3 | -2.64 |
| Ccl21b | chemokine (C-C motif) ligand 21b (Ccl21b) | -2.65 |
| Mterf | mitochondrial transcription termination factor (Mterf), nuclear gene encoding mitochondrial protein, transcript variant 1 | -2.65 |
| Hmha1 | histocompatibility (minor) HA-1 (Hmha1) | -2.66 |
| Mif4gd | MIF4G domain containing (Mif4gd) | -2.66 |
| Itk | IL2-inducible T-cell kinase (Itk) | -2.67 |
| Hp1bp3 | heterochromatin protein 1, binding protein 3 (Hp1bp3) | -2.68 |
| Arhgef3 | Rho guanine nucleotide exchange factor (GEF) 3 (Arhgef3) | -2.68 |
| Egr1 | early growth response 1 (Egr1) | -2.69 |
| Itgb7 | integrin beta 7 (Itgb7) | -2.72 |
| Tle4 | transducin-like enhancer of split 4, homolog of Drosophila E(spl) (Tle4) | -2.72 |
| Trib2 | tribbles pseudokinase 2 (Trib2) | -2.73 |
| AI467606 | expressed sequence AI467606 (AI467606) | -2.73 |
| Tmem66 | transmembrane protein 66 (Tmem66) | -2.74 |
| Pptc7 | PTC7 protein phosphatase homolog (S. cerevisiae) (Pptc7) | -2.75 |
| Cxcr4 | chemokine (C-X-C motif) receptor 4 (Cxcr4) | -2.75 |
| Dazap2 | DAZ associated protein 2 (Dazap2) | -2.77 |
| Fxyd5 | FXYD domain-containing ion transport regulator 5 (Fxyd5) | -2.79 |
| Dpagt1 | dolichyl-phosphate (UDP-N-acetylglucosamine) acetylglucosaminephosphotransferase 1 (GlcNAc-1-P transferase) (Dpagt1) | -2.8 |
| Skil | SKI-like (Skil), transcript variant 2 | -2.8 |
| Dcp1b | DCP1 decapping enzyme homolog b (S. cerevisiae) (Dcp1b) | -2.81 |
| Lck | lymphocyte protein tyrosine kinase (Lck) | -2.82 |
| Trub2 | TruB pseudouridine (psi) synthase homolog 2 (E. coli) (Trub2) | -2.82 |
| Cd3d | CD3 antigen, delta polypeptide (Cd3d) | -2.83 |
| Trp53inp1 | transformation related protein 53 inducible nuclear protein 1 (Trp53inp1) | -2.84 |
| Rnase6 | ribonuclease, RNase A family, 6 (Rnase6) | -2.85 |
| Dnajc7 | DnaJ heat shock protein family (Hsp40) member C7 (Dnajc7) | -2.87 |
| St8sia4 | ST8 alpha-N-acetyl-neuraminide alpha-2,8-sialyltransferase 4 (St8sia4) | -2.87 |
| Tnfaip3 | tumor necrosis factor, alpha-induced protein 3 (Tnfaip3) | -2.88 |
| Cd3e | CD3 antigen, epsilon polypeptide (Cd3e) | -2.88 |
| ENSMUSG00000053178 | predicted gene, ENSMUSG00000053178 (ENSMUSG00000053178), nuclear gene encoding mitochondrial protein | -2.9 |
| Satb1 | special AT-rich sequence binding protein 1 (Satb1) | -2.91 |
| Vars | valyl-tRNA synthetase (Vars) | -2.93 |
| 4833420G17Rik | RIKEN cDNA 4833420G17 gene (4833420G17Rik) | -2.93 |
| Xlr4a | X-linked lymphocyte-regulated 4A (Xlr4a) | -2.93 |
| Stk17b | serine/threonine kinase 17b (apoptosis-inducing) (Stk17b) | -2.95 |
| Cugbp2 | CUG triplet repeat, RNA binding protein 2 (Cugbp2), transcript variant 6 | -2.96 |
| Tnfrsf4 | tumor necrosis factor receptor superfamily, member 4 (Tnfrsf4) | -2.97 |
| Thy1 | thymus cell antigen 1, theta (Thy1) | -2.99 |
| Tef | thyrotroph embryonic factor (Tef), transcript variant 1 | -3 |
| Gpr114 | G protein-coupled receptor 114 (Gpr114) | -3 |
| Plekha1 | pleckstrin homology domain containing, family A (phosphoinositide binding specific) member 1 (Plekha1) | -3.01 |
| C030048B08Rik | RIKEN cDNA C030048B08 gene (C030048B08Rik) | -3.01 |
| Faim3 | Fas apoptotic inhibitory molecule 3 (Faim3) | -3.02 |
| Herpud1 | homocysteine-inducible, endoplasmic reticulum stress-inducible, ubiquitin-like domain member 1 (Herpud1) | -3.02 |
| Dusp6 | dual specificity phosphatase 6 (Dusp6) | -3.02 |
| Il18r1 | interleukin 18 receptor 1 (Il18r1) | -3.03 |
| Cd53 | CD53 antigen (Cd53) | -3.03 |
| Birc2 | baculoviral IAP repeat-containing 2 (Birc2) | -3.03 |
| Eef1b2 | eukaryotic translation elongation factor 1 beta 2 (Eef1b2) | -3.04 |
| H2-Aa | histocompatibility 2, class II antigen A, alpha (H2-Aa) | -3.06 |
| Cd3g | CD3 antigen, gamma polypeptide (Cd3g) | -3.06 |
| Arhgap30 | Rho GTPase activating protein 30 (Arhgap30) | -3.07 |
| Pscdbp | pleckstrin homology, Sec7 and coiled-coil domains, binding protein (Pscdbp) | -3.07 |
| Hs3st1 | heparan sulfate (glucosamine) 3-O-sulfotransferase 1 (Hs3st1) | -3.09 |
| A730098D12Rik | YTH domain containing 1 (Ythdc1) | -3.1 |
| Tbc1d10c | TBC1 domain family, member 10c (Tbc1d10c) | -3.11 |
| BC017643 | cDNA sequence BC017643 (BC017643) | -3.11 |
| Slc12a2 | solute carrier family 12, member 2 (Slc12a2) | -3.11 |
| Sesn1 | sestrin 1 (Sesn1) | -3.16 |
| Pkig | protein kinase inhibitor, gamma (Pkig), transcript variant 3 | -3.16 |
| Gimap8 | GTPase, IMAP family member 8 (Gimap8), transcript variant 1 | -3.17 |
| Rag1ap1 | recombination activating gene 1 activating protein 1 (Rag1ap1) | -3.18 |
| Btg1 | B-cell translocation gene 1, anti-proliferative (Btg1) | -3.18 |
| B3gnt5 | UDP-GlcNAc:betaGal beta-1,3-N-acetylglucosaminyltransferase 5 (B3gnt5) | -3.2 |
| Cd27 | CD27 antigen (Cd27), transcript variant 1 | -3.23 |
| Birc2 | baculoviral IAP repeat-containing 2 (Birc2) | -3.24 |
| Uap1 | UDP-N-acetylglucosamine pyrophosphorylase 1 (Uap1) | -3.25 |
| Rbm5 | RNA binding motif protein 5 (Rbm5) | -3.26 |
| LOC433801 | similar to RIKEN cDNA 6330416L07 gene (LOC433801) | -3.28 |
| Sfrs16 | CLK4 associating serine/arginine rich protein (Sfrs16) | -3.28 |
| Zc3h7a | zinc finger CCCH type containing 7 A (Zc3h7a) | -3.29 |
| Ift140 | intraflagellar transport 140 homolog (Chlamydomonas) (Ift140) | -3.34 |
| Psg23 | pregnancy-specific glycoprotein 23 (Psg23) | -3.37 |
| Dbp | D site albumin promoter binding protein (Dbp) | -3.4 |
| Map3k1 | mitogen-activated protein kinase kinase kinase 1 (Map3k1) | -3.41 |
| 6430706D22Rik | RIKEN cDNA 6430706D22 gene (6430706D22Rik) | -3.45 |
| Arhgef18 | rho/rac guanine nucleotide exchange factor (GEF) 18 (Arhgef18) | -3.46 |
| Ctgf | connective tissue growth factor (Ctgf) | -3.47 |
| Cd8b1 | CD8 antigen, beta chain 1 (Cd8b1) | -3.49 |
| Lgals4 | lectin, galactose binding, soluble 4 (Lgals4) | -3.5 |
| Cd69 | CD69 antigen (Cd69) | -3.58 |
| Gvin1 | GTPase, very large interferon inducible 1 (Gvin1), transcript variant B | -3.62 |
| Btg1 | B-cell translocation gene 1, anti-proliferative (Btg1) | -3.66 |
| Clk4 | CDC like kinase 4 (Clk4) | -3.68 |
| Tmem68 | transmembrane protein 68 (Tmem68) | -3.71 |
| Chfr | checkpoint with forkhead and ring finger domains (Chfr) | -3.72 |
| Lbh | limb-bud and heart (Lbh) | -3.86 |
| Ptpn22 | protein tyrosine phosphatase, non-receptor type 22 (lymphoid) (Ptpn22) | -3.9 |
| Ddit4 | DNA-damage-inducible transcript 4 (Ddit4) | -3.94 |
| Lat | linker for activation of T cells (Lat) | -3.98 |
| Bsdc1 | BSD domain containing 1 (Bsdc1) | -4 |
| Ccl5 | chemokine (C-C motif) ligand 5 (Ccl5) | -4.29 |
| 4930432O21Rik | RIKEN cDNA 4930432O21 gene (4930432O21Rik) | -4.32 |
| Adam23 | a disintegrin and metallopeptidase domain 23 (Adam23) | -4.86 |
| Rps3a | ribosomal protein S3a (Rps3a) | -4.89 |
| Lyz1 | lysozyme 1 (Lyz1) | -5.08 |
| Il7r | interleukin 7 receptor (Il7r) | -5.11 |
| Serinc3 | serine incorporator 3 (Serinc3) | -5.15 |
| Tsc22d3 | TSC22 domain family 3 (Tsc22d3), transcript variant 1 | -5.8 |
| LOC100044862 | similar to Fbxl3 protein (LOC100044862) | -6.75 |
| Emb | embigin (Emb) | -8.37 |
