# Supplementary material for: Genomic deletion of GIT2 induces a premature age-related thymic dysfunction and systemic immune system disruption
Source: Aging (Albany NY). 2017 Mar 4;9(3):706–30. doi: 10.18632/aging.101185 (PMC5391227; doi:10.18632/aging.101185)
Supplement: Supplementary file 14 [file aging-09-706-s014.docx]

**Table S13. Venn diagram separation of transcripts significantly regulated differentially between the GIT2KO inguinal lymph node (ILN), mesenteric lymph node (MLN), Spleen and Thymus compared to WT tissue counterparts.** The official Gene Symbol and associated Z ratios for the comparisons of the GIT2KO *vs.* WT tissues at the 12 month time point are indicated. Each transcript was significantly regulated at p<0.05, with a Z ratio >± 1.5.

.

| **Gene Symbol** | **ILN** | **MLN** | **Spleen** | **Thymus** |
| --- | --- | --- | --- | --- |
| Kras | 7.67 | 5.88 | 3.72 | 4.16 |
| H2-T10 | 7.08 | 8.5 | 4.57 | 10.36 |
| Pik3cg | 6.03 | 6.48 | 1.99 | 3.48 |
| Hp | 6 | 4.35 | 5.97 | 8.67 |
| Gfer | 4.25 | 3.23 | 2.19 | 2.55 |
| Ndufb10 | 4.21 | 4.08 | 1.84 | 2.44 |
| Glo1 | 4.01 | 4.91 | 3.1 | 3.83 |
| LOC100043671 | 3.93 | 4.77 | 1.71 | 5.98 |
| Adi1 | 3.79 | 4.99 | 3.49 | 5.38 |
| Sqle | 3.62 | 3 | 2.49 | 2.51 |
| Eno1 | 2.85 | 1.54 | 2.42 | 4.29 |
| Cnot7 | 2.58 | 1.84 | 1.82 | 1.53 |
| Rnase4 | 1.68 | 2.77 | 4.51 | 2.89 |
|  |  |  |  |  |
| Tnrc6a | -1.59 | -1.69 | -2.1 | -2.2 |
| Vars | -1.73 | -1.85 | -2.93 | -2.15 |
| Spsb3 | -1.98 | -1.62 | -1.8 | -2.12 |
| Gtf2h1 | -2.01 | -1.62 | -2.24 | -2.39 |
| 4933439C20Rik | -2.1 | -2.36 | -2.11 | -1.84 |
| Btla | -2.18 | -2.56 | -2.57 | -1.55 |
| Gnptg | -2.55 | -1.55 | -1.64 | -2.84 |
| 4930432O21Rik | -2.66 | -1.55 | -4.32 | -1.59 |
| Sin3a | -2.72 | -3.03 | -1.9 | -2.55 |
| LOC545056 | -2.75 | -2.41 | -2.57 | -2.63 |
| Psg23 | -2.76 | -1.65 | -3.37 | -3.43 |
| Dpagt1 | -3.08 | -2.95 | -2.8 | -2.93 |
| Pkm2 | -3.08 | -4.21 | -1.96 | -2.12 |
| AI467606 | -3.13 | -2.23 | -2.73 | -1.96 |
| Bcl9l | -3.5 | -3.4 | -2.55 | -1.85 |
| Per2 | -3.56 | -1.61 | -1.61 | -2.94 |
| Tef | -4.16 | -2.96 | -3 | -3.27 |
| Tnfrsf4 | -4.3 | -2.65 | -2.97 | -3.83 |
| Rps3a | -4.62 | -2.92 | -4.89 | -4.16 |
| Tsc22d3 | -4.75 | -3.98 | -5.8 | -4.54 |
| Bsdc1 | -5.09 | -4.82 | -4 | -5.89 |
| LOC100044862 | -5.91 | -3.92 | -6.75 | -5.08 |
| 6430706D22Rik | -6.05 | -4.14 | -3.45 | -4.61 |
| Per1 | -6.09 | -3.42 | -2.45 | -3.19 |
| Dbp | -7.24 | -3.88 | -3.4 | -3.35 |
| Ddit4 | -7.77 | -5.8 | -3.94 | -4.14 |
| Mgst2 | -8.26 | -3.93 | -2.09 | -7.34 |
|  |  |  |  |  |
| Scd1 | 4.18 | 4.08 | -2.34 | 7.41 |
| Tmem66 | 3.88 | 5.97 | -2.74 | 3.73 |
| Vars2 | 2.31 | 1.72 | 1.5 | -1.72 |
| Arpc5 | 1.88 | -1.81 | 2.6 | 1.69 |
| Mgst1 | 1.63 | -2.4 | 2.82 | 5.76 |
| Il18r1 | -1.84 | 2.95 | -3.03 | -2.35 |
|  |  |  |  |  |
| Lpl | 4.57 |  | 2.05 | 5.91 |
| Hspa8 | 4.08 |  | 7.87 | 4.89 |
| Hnrpk | 3.41 |  | 3.97 | 2.87 |
| Actb | 3.39 |  | 5.24 | 3.12 |
| Aoc3 | 3.11 |  | 2.08 | 5.57 |
| Loxl1 | 2.9 |  | 1.59 | 3.28 |
| Eif5a | 2.73 |  | 4.99 | 2.92 |
| Pfn1 | 2.71 |  | 2.79 | 2.59 |
| Hnrnpa2b1 | 2.62 |  | 3.23 | 2.7 |
| Ptp4a2 | 2.58 |  | 2.89 | 3.07 |
| Hsp90ab1 | 2.49 |  | 3.83 | 2.03 |
| EG434858 | 2.48 |  | 3.32 | 3.01 |
| Ppp1ca | 2.41 |  | 4.55 | 3.51 |
| Sec61b | 2.4 |  | 2.41 | 1.73 |
| Idh3g | 2.35 |  | 4.19 | 4.32 |
| Stx8 | 2.34 |  | 2.63 | 2.21 |
| Sparc | 2.34 |  | 5.96 | 4.29 |
| Rps27a | 2.33 |  | 2.59 | 2.15 |
| Atp5f1 | 2.19 |  | 3.39 | 3.64 |
| Gnb1 | 2.16 |  | 2.46 | 1.68 |
| Caprin1 | 2.15 |  | 2.85 | 3.2 |
| Nudt4 | 2.13 |  | 2.35 | 2.46 |
| EG433923 | 2.11 |  | 3.01 | 3.44 |
| Nudt5 | 2.08 |  | 1.91 | 2.12 |
| Txnl4a | 2.04 |  | 3.92 | 2.94 |
| Tuba1a | 2 |  | 2.68 | 2.24 |
| Oxct1 | 1.99 |  | 2.66 | 2.2 |
| Mthfd1 | 1.99 |  | 2.73 | 1.82 |
| Dhx15 | 1.96 |  | 2.05 | 2.1 |
| Ndufc1 | 1.84 |  | 2.35 | 3.1 |
| Eif5 | 1.82 |  | 2.14 | 2.06 |
| Mrpl3 | 1.82 |  | 1.76 | 2.93 |
| Mylc2b | 1.78 |  | 2.61 | 2.31 |
| Psmd7 | 1.76 |  | 3.02 | 2.45 |
| Arpc1a | 1.74 |  | 3.41 | 1.73 |
| Smu1 | 1.74 |  | 2.19 | 3.15 |
| Ndufa12 | 1.72 |  | 2.29 | 3.26 |
| Gnas | 1.7 |  | 3.39 | 2.63 |
| Snrpd1 | 1.64 |  | 2.36 | 1.93 |
| Coro1a | 1.64 |  | 2.79 | 2.21 |
| Fdps | 1.58 |  | 2.12 | 2.04 |
| Nola2 | 1.57 |  | 2.24 | 2.65 |
| Col4a1 | 1.54 |  | 2.24 | 1.8 |
| Pcbp1 | 1.51 |  | 2.68 | 1.85 |
|  |  |  |  |  |
| Brd2 | -1.52 |  | -2.24 | -2.15 |
| Hp1bp3 | -1.59 |  | -2.68 | -1.64 |
| Arhgef1 | -1.66 |  | -1.53 | -1.58 |
| Cbx7 | -1.67 |  | -2.12 | -1.9 |
| Pde1b | -1.77 |  | -2.13 | -2.3 |
| Itgb7 | -1.79 |  | -2.72 | -1.98 |
| Hmha1 | -1.84 |  | -2.66 | -2.92 |
| Mib2 | -1.84 |  | -1.7 | -2.43 |
| Trib2 | -1.95 |  | -2.73 | -2.04 |
| 2610207I05Rik | -1.97 |  | -2.6 | -2.41 |
| D230007K08Rik | -2.03 |  | -2.41 | -3.31 |
| Dcp1b | -2.15 |  | -2.81 | -1.59 |
| Dgka | -2.15 |  | -1.55 | -1.9 |
| Tmem68 | -2.21 |  | -3.71 | -1.9 |
| Hcst | -2.37 |  | -1.73 | -1.72 |
| Klk8 | -2.41 |  | -1.98 | -2.54 |
| Cecr5 | -2.45 |  | -2.48 | -3.3 |
| Hmgcs2 | -2.6 |  | -1.77 | -2.17 |
| Gpr114 | -2.86 |  | -3 | -3.21 |
| EG434197 | -3.47 |  | -2.46 | -1.7 |
| Ctgf | -3.98 |  | -3.47 | -2.54 |
| Txnip | -4.14 |  | -2.56 | -2.47 |
| Dkk3 | -4.23 |  | -1.72 | -3.21 |
| Lgals4 | -4.42 |  | -3.5 | -2.82 |
| Emb | -14.52 |  | -8.37 | -10.7 |
|  |  |  |  |  |
| Sfrs5 | 2.13 |  | -1.64 | 2.8 |
| Cd44 | 1.52 |  | -2.15 | -1.95 |
| Fxyd5 | -2.24 |  | 1.71 | -1.63 |
| Igfbp4 | -2.31 |  | 1.79 | 2.04 |
| Cxcl12 | -2.67 |  | 5.32 | 3.19 |
| Dcn | -2.89 |  | 2.99 | 2.73 |
|  |  |  |  |  |
| Ctse | 14.23 | 13.47 |  | 9.86 |
| Phlda1 | 3.22 | 1.65 |  | 1.63 |
| Gng10 | 3.14 | 1.97 |  | 3.87 |
| Rnf11 | 1.94 | 2.45 |  | 2.32 |
| Rb1 | 1.88 | 3.73 |  | 1.92 |
| 2610204L23Rik | 1.84 | 2.12 |  | 1.78 |
| Ndn | 1.73 | 3.5 |  | 3.6 |
|  |  |  |  |  |
| Dgkz | -1.54 | -2 |  | -1.96 |
| BC025076 | -1.75 | -2.85 |  | -1.64 |
| Ssbp3 | -2.07 | -1.58 |  | -2.08 |
| Sidt2 | -2.5 | -2.1 |  | -1.9 |
| AI450540 | -2.6 | -1.78 |  | -1.72 |
| Trim56 | -2.71 | -1.78 |  | -1.61 |
| Rab27a | -2.86 | -1.57 |  | -2.65 |
| Vat1 | -2.88 | -2.41 |  | -1.81 |
| Tsc2 | -3.05 | -3.12 |  | -2.2 |
| Dusp7 | -3.38 | -3.77 |  | -2.67 |
| Galnt10 | -4.27 | -3.94 |  | -3.06 |
| LOC100047214 | -4.45 | -3.22 |  | -2.59 |
|  |  |  |  |  |
| Tspan3 | -5.06 | -3.3 |  | 2.8 |
| Prg2 | -5.1 | 2.93 |  | -1.57 |
|  |  |  |  |  |
| Cxcl9 | 4.91 | 4.85 | 1.85 |  |
| Fgd2 | 3.94 | 2.7 | 2.8 |  |
| Hebp1 | 3.33 | 2.11 | 2.12 |  |
| Cap1 | 3.1 | 3.93 | 3.21 |  |
| Sepx1 | 1.84 | 1.9 | 1.85 |  |
|  |  |  |  |  |
| Arhgef18 | -1.88 | -1.7 | -3.46 |  |
| Nt5e | -1.98 | -3.27 | -2.62 |  |
| 2310039H08Rik | -2.17 | -1.88 | -1.85 |  |
| Sesn1 | -2.45 | -2.04 | -3.16 |  |
| Cd3e | -2.8 | -1.63 | -2.88 |  |
| Atp1b1 | -3.28 | -2.51 | -2.22 |  |
| Hs3st1 | -3.4 | -3.12 | -3.09 |  |
| Bbc3 | -4.32 | -3.12 | -2.13 |  |
|  |  |  |  |  |
| Cd79b | 2.63 | -2 | 1.75 |  |
| LOC100047963 | 2.09 | 2.29 | -1.97 |  |
| Ifi47 | 1.6 | 2.3 | -1.52 |  |
| Lat | -1.83 | 1.94 | -3.98 |  |
| Cyp1b1 | -1.93 | 3.78 | -2.37 |  |
| Sepp1 | -2.73 | 2.59 | 3.4 |  |
|  |  |  |  |  |
| Cav1 | 3.23 |  |  | 5.37 |
| Hnrpa1 | 3.05 |  |  | 2.15 |
| Fh1 | 2.82 |  |  | 3.55 |
| Ppa1 | 2.74 |  |  | 2.21 |
| Nap1l1 | 2.58 |  |  | 2.08 |
| Tank | 2.34 |  |  | 1.69 |
| LOC100047934 | 2.25 |  |  | 3.19 |
| LOC100047155 | 2.23 |  |  | 2.14 |
| Msn | 2.2 |  |  | 2.64 |
| Inppl1 | 1.96 |  |  | 1.51 |
| Ywhag | 1.95 |  |  | 3.18 |
| Sdhc | 1.93 |  |  | 2.22 |
| 15-Sep | 1.92 |  |  | 3.07 |
| LOC100046855 | 1.82 |  |  | 1.69 |
| Gpd2 | 1.77 |  |  | 2.53 |
| Aacs | 1.7 |  |  | 2.28 |
| H3f3a | 1.69 |  |  | 2.42 |
| H2-K1 | 1.64 |  |  | 3.68 |
| Hsd17b12 | 1.64 |  |  | 2.4 |
| Snap23 | 1.62 |  |  | 1.99 |
| Pno1 | 1.61 |  |  | 2.67 |
| Rpa3 | 1.58 |  |  | 1.96 |
| Coq5 | 1.56 |  |  | 1.88 |
| LOC100045617 | 1.55 |  |  | 1.54 |
|  |  |  |  |  |
| Tgfbi | -1.51 |  |  | -1.6 |
| Ehmt2 | -1.52 |  |  | -1.63 |
| ORF61 | -1.55 |  |  | -1.56 |
| Map3k7ip1 | -1.55 |  |  | -2.33 |
| Bcl11b | -1.6 |  |  | -1.76 |
| Acox3 | -1.63 |  |  | -1.97 |
| Pik3r1 | -1.66 |  |  | -2.1 |
| Pacs2 | -1.66 |  |  | -1.77 |
| Zfp579 | -1.8 |  |  | -2.24 |
| Dscr1l2 | -1.82 |  |  | -1.87 |
| Psen2 | -1.95 |  |  | -2.76 |
| Ppox | -1.97 |  |  | -1.72 |
| Il6st | -2 |  |  | -1.54 |
| Prkcbp1 | -2.02 |  |  | -2.45 |
| 1810073G14Rik | -2.08 |  |  | -2.73 |
| Tcf25 | -2.2 |  |  | -2.44 |
| Rcan3 | -2.25 |  |  | -1.54 |
| Scarf2 | -2.57 |  |  | -1.92 |
| Ihpk1 | -2.6 |  |  | -2.02 |
| Acot1 | -2.89 |  |  | -2.1 |
| Mt1 | -3.91 |  |  | -2.45 |
| Inmt | -12.71 |  |  | -1.82 |
|  |  |  |  |  |
| Stat1 | 3.35 |  |  | -1.63 |
| Ly6d | 2.25 |  |  | -2.31 |
| Ccnd1 | 1.93 |  |  | -1.59 |
| Slc9a3r2 | -1.91 |  |  | 1.66 |
| Sdpr | -3.64 |  |  | 3.64 |
| Fmo1 | -4.15 |  |  | 3.62 |
| Hba-a1 | -7.67 |  |  | 8.33 |
|  |  |  |  |  |
| LOC100038882 | 5.41 |  | 3 |  |
| Lyz2 | 3.6 |  | 2.23 |  |
| Clec4n | 3.19 |  | 4.22 |  |
| Asns | 3 |  | 3.32 |  |
| Col6a1 | 2.59 |  | 2.09 |  |
| Cd9 | 2.58 |  | 2.68 |  |
| Gstm2 | 2.2 |  | 2.17 |  |
| Emp2 | 2.16 |  | 1.97 |  |
| H2-Ab1 | 2.14 |  | 1.71 |  |
| Csrp1 | 2.13 |  | 2.93 |  |
| Vwf | 2.13 |  | 2.56 |  |
| Hist1h2ak | 2.09 |  | 4.12 |  |
| Cnr2 | 2 |  | 1.77 |  |
| Uck2 | 1.98 |  | 1.62 |  |
| Mylk | 1.94 |  | 3.84 |  |
| Sphk1 | 1.9 |  | 3.02 |  |
| Gch1 | 1.89 |  | 3.02 |  |
| Ppp2r5c | 1.74 |  | 2.45 |  |
| Col4a2 | 1.72 |  | 3.2 |  |
| Adamts2 | 1.71 |  | 1.72 |  |
| Dhrs1 | 1.63 |  | 1.86 |  |
| Lcp1 | 1.5 |  | 2.01 |  |
|  |  |  |  |  |
| Atf7ip | -1.5 |  | -1.89 |  |
| Cd6 | -1.52 |  | -1.63 |  |
| D10Ertd641e | -1.53 |  | -1.57 |  |
| Entpd4 | -1.6 |  | -1.51 |  |
| Slc6a6 | -1.64 |  | -1.53 |  |
| Ttc3 | -1.67 |  | -1.5 |  |
| Cxcr4 | -1.68 |  | -2.75 |  |
| Gprasp1 | -1.79 |  | -2.64 |  |
| Ctsw | -1.83 |  | -2.4 |  |
| Cd27 | -1.86 |  | -3.23 |  |
| Lck | -1.91 |  | -2.82 |  |
| Dusp1 | -1.92 |  | -1.6 |  |
| Cd8b1 | -1.96 |  | -3.49 |  |
| Plrg1 | -1.97 |  | -2.15 |  |
| Zap70 | -2.15 |  | -1.79 |  |
| Itk | -2.28 |  | -2.67 |  |
| Ptpn22 | -2.36 |  | -3.9 |  |
| Mycl1 | -2.42 |  | -1.7 |  |
| Cd3d | -3.13 |  | -2.83 |  |
| Sult1a1 | -3.31 |  | -1.92 |  |
| Cd3g | -3.33 |  | -3.06 |  |
| Il7r | -4.17 |  | -5.11 |  |
| Slco2b1 | -4.54 |  | -2.13 |  |
|  |  |  |  |  |
| Lyz1 | 4.06 |  | -5.08 |  |
| B3gnt5 | 3.07 |  | -3.2 |  |
| Fcrla | 2.49 |  | -1.62 |  |
| Tnfrsf13c | 2.46 |  | -1.6 |  |
| Swap70 | 2.14 |  | -2.35 |  |
| H2-Ob | 2.11 |  | -1.72 |  |
| Bhlhb2 | 1.65 |  | -2.46 |  |
| Mfge8 | 1.65 |  | -2.13 |  |
| Ythdf2 | 1.52 |  | -2.24 |  |
| H2afx | -1.61 |  | 1.85 |  |
| Slc11a1 | -1.61 |  | 2.4 |  |
| Add1 | -1.63 |  | 1.73 |  |
| Nfix | -1.87 |  | 1.83 |  |
| Trf | -1.95 |  | 3.26 |  |
| Mmp2 | -2.05 |  | 1.84 |  |
| Gabarapl1 | -2.46 |  | 2.39 |  |
| Mgp | -2.98 |  | 2.71 |  |
| Fcna | -4.1 |  | 1.95 |  |
|  |  |  |  |  |
| Cdr2 |  | 2.53 |  | 2.65 |
| Pcyox1 |  | 2.27 |  | 2.03 |
| Hadhb |  | 2.2 |  | 2.58 |
| LOC100048480 |  | 2.15 |  | 2.17 |
| Pmp22 |  | 2.03 |  | 2.07 |
| Lgals1 |  | 1.92 |  | 1.63 |
| 1810035L17Rik |  | 1.72 |  | 2.29 |
| Ak3 |  | 1.61 |  | 1.58 |
| Slc25a1 |  | 1.55 |  | 2.63 |
|  |  |  |  |  |
| Phkg2 |  | -1.53 |  | -1.64 |
| Akna |  | -1.9 |  | -1.77 |
| Scrib |  | -2.05 |  | -1.78 |
| Atpbd1b |  | -2.07 |  | -2.4 |
| Hvcn1 |  | -2.1 |  | -2.06 |
| BC067047 |  | -2.63 |  | -1.84 |
| Arid3b |  | -2.92 |  | -2.69 |
| Nfatc3 |  | -3.04 |  | -1.51 |
| Axud1 |  | -4.12 |  | -3.07 |
| Ddx6 |  | -4.47 |  | -1.8 |
| Bach1 |  | -5.41 |  | -2.52 |
|  |  |  |  |  |
| 9130422G05Rik |  | 2.4 |  | -2.14 |
| Hsp90b1 |  | 2.21 |  | -1.77 |
| Dok2 |  | 2.2 |  | -1.76 |
| Rps6 |  | -1.94 |  | 1.89 |
|  |  |  |  |  |
| Ccl19 |  | 3.48 | 3.32 |  |
| P2ry6 |  | 2.03 | 1.97 |  |
| Hrbl |  | 2.02 | 2.71 |  |
| C1qc |  | 1.75 | 3.01 |  |
| Rnasek |  | 1.64 | 3.02 |  |
| Pscd3 |  | 1.54 | 2.15 |  |
|  |  |  |  |  |
| Suz12 |  | -1.62 | -1.58 |  |
| Mrps7 |  | -1.66 | -1.61 |  |
| Ugcg |  | -1.75 | -2.53 |  |
| Cd83 |  | -1.82 | -1.65 |  |
| Ppp3ca |  | -1.93 | -2.42 |  |
| Slc25a19 |  | -2.16 | -1.79 |  |
| Ift140 |  | -2.2 | -3.34 |  |
| LOC100044439 |  | -2.86 | -2.21 |  |
|  |  |  |  |  |
| S100a10 |  | 2.76 | -1.51 |  |
| Slain2 |  | 2.7 | -1.83 |  |
| Ptges3 |  | 2.62 | -1.73 |  |
| Vapa |  | 2.24 | -1.59 |  |
| Sec11c |  | 2.22 | -2.58 |  |
| Vti1b |  | 1.91 | -1.88 |  |
| Ss18 |  | 1.77 | -2.48 |  |
| Cugbp2 |  | 1.73 | -1.69 |  |
| Il16 |  | 1.63 | -2.28 |  |
| Npc2 |  | 1.6 | -1.69 |  |
| Smek2 |  | 1.51 | -1.72 |  |
| Hagh |  | -1.64 | 2.45 |  |
| Zfp91 |  | -2.45 | 2.46 |  |
|  |  |  |  |  |
| St6galnac2 | 5.4 | 6.28 |  |  |
| Vegfa | 4.15 | 1.96 |  |  |
| Cd84 | 3.23 | 2.29 |  |  |
| LOC100040592 | 2.61 | 1.58 |  |  |
| Igfbp5 | 2.51 | 1.77 |  |  |
| 1200002N14Rik | 2.46 | 1.94 |  |  |
| Ubd | 2.29 | 3.33 |  |  |
| Lrg1 | 2.22 | 1.98 |  |  |
| Zfp238 | 2.19 | 2.6 |  |  |
| Unc45a | 2.17 | 1.56 |  |  |
| Cd55 | 2.08 | 2.86 |  |  |
| Il33 | 1.91 | 1.67 |  |  |
| Vdac2 | 1.84 | 1.71 |  |  |
| Adam17 | 1.82 | 1.69 |  |  |
| Chrnb1 | 1.78 | 1.69 |  |  |
| Tgfbr1 | 1.67 | 1.57 |  |  |
| Slc38a2 | 1.67 | 2.03 |  |  |
| Fes | 1.64 | 1.84 |  |  |
| Cd2bp2 | 1.55 | 1.68 |  |  |
| Stard4 | 1.52 | 2.01 |  |  |
|  |  |  |  |  |
| Mllt3 | -1.78 | -3.76 |  |  |
| Mrpl48 | -1.79 | -1.63 |  |  |
| Eif4el3 | -1.89 | -2.31 |  |  |
| H2-M3 | -1.94 | -1.67 |  |  |
| Ctsa | -1.96 | -2.12 |  |  |
| Mrpl9 | -2.06 | -1.88 |  |  |
| Ephx1 | -2.21 | -2.08 |  |  |
| Gsto1 | -2.23 | -3.81 |  |  |
| Acpl2 | -4.02 | -3.65 |  |  |
|  |  |  |  |  |
| Mef2c | 1.98 | -4.95 |  |  |
| Napsa | 1.88 | -2.2 |  |  |
| Lip1 | -2.44 | 1.97 |  |  |
|  |  |  |  |  |
| Eef2 |  |  | 6.06 | 2.26 |
| Ces3 |  |  | 5.44 | 4.38 |
| Mgst3 |  |  | 4.95 | 3.07 |
| Tmem14c |  |  | 3.58 | 2.07 |
| Gstp1 |  |  | 3.51 | 2.15 |
| S100a1 |  |  | 3.48 | 3.86 |
| Hmgb2 |  |  | 3.32 | 1.78 |
| Adk |  |  | 3.28 | 2.68 |
| Lamp2 |  |  | 3.08 | 2.41 |
| 5730437N04Rik |  |  | 3.03 | 1.82 |
| Naca |  |  | 3 | 2.57 |
| D10Ertd322e |  |  | 2.98 | 1.92 |
| Gstm1 |  |  | 2.96 | 3.47 |
| Spcs1 |  |  | 2.91 | 2.53 |
| Atf4 |  |  | 2.86 | 1.53 |
| Bola3 |  |  | 2.85 | 2.42 |
| Psmb7 |  |  | 2.81 | 3.32 |
| Rarres2 |  |  | 2.81 | 3.64 |
| Ifitm2 |  |  | 2.78 | 2 |
| Dcps |  |  | 2.78 | 2.38 |
| Gpsn2 |  |  | 2.72 | 1.83 |
| Pdhb |  |  | 2.69 | 3.67 |
| Prdx3 |  |  | 2.67 | 2.16 |
| Tspo |  |  | 2.65 | 2.46 |
| Fkbp2 |  |  | 2.63 | 2.2 |
| Arl6ip5 |  |  | 2.49 | 1.84 |
| EG622339 |  |  | 2.48 | 1.68 |
| Cox6b1 |  |  | 2.46 | 2.54 |
| Mcm5 |  |  | 2.43 | 1.69 |
| Vdac3 |  |  | 2.4 | 2.74 |
| Ddx47 |  |  | 2.38 | 1.54 |
| Hsd11b1 |  |  | 2.38 | 1.79 |
| MGC18837 |  |  | 2.32 | 2.02 |
| Aebp1 |  |  | 2.26 | 2.6 |
| Usp39 |  |  | 2.26 | 2.33 |
| Prkcdbp |  |  | 2.25 | 1.53 |
| Cd151 |  |  | 2.24 | 2.68 |
| Supt4h2 |  |  | 2.22 | 2.69 |
| Cox6a1 |  |  | 2.21 | 2.77 |
| Dci |  |  | 2.21 | 4.21 |
| Ppp2r1a |  |  | 2.16 | 1.69 |
| Ndufa5 |  |  | 2.12 | 1.99 |
| Cyc1 |  |  | 2.12 | 2.85 |
| Mid1ip1 |  |  | 2.11 | 2.18 |
| Timm8b |  |  | 2.11 | 2.17 |
| 2310016E02Rik |  |  | 2.07 | 1.72 |
| 9430029K10Rik |  |  | 2.07 | 2 |
| Vps29 |  |  | 2.06 | 1.73 |
| Vkorc1 |  |  | 2 | 1.58 |
| Zfp207 |  |  | 1.97 | 2.28 |
| Fhl1 |  |  | 1.94 | 3.88 |
| H2afz |  |  | 1.89 | 1.92 |
| Ube2k |  |  | 1.88 | 1.64 |
| Lsm2 |  |  | 1.84 | 1.78 |
| Capns1 |  |  | 1.81 | 1.9 |
| Snrpb |  |  | 1.8 | 1.59 |
| Ndufa8 |  |  | 1.79 | 2.78 |
| Ahsa1 |  |  | 1.79 | 1.6 |
| LOC100048613 |  |  | 1.78 | 1.74 |
| Ndufs2 |  |  | 1.76 | 1.75 |
| Gpx4 |  |  | 1.75 | 2.26 |
| Yif1a |  |  | 1.75 | 1.77 |
| Ebp |  |  | 1.74 | 1.51 |
| Nola3 |  |  | 1.7 | 2.89 |
| Smarce1 |  |  | 1.66 | 2 |
| Dph3 |  |  | 1.64 | 2.2 |
| 2610029G23Rik |  |  | 1.62 | 2.02 |
| Cops6 |  |  | 1.59 | 1.54 |
| Ndufb6 |  |  | 1.56 | 3.24 |
| Vim |  |  | 1.56 | 1.94 |
| Cpt2 |  |  | 1.52 | 3.55 |
|  |  |  |  |  |
| Arhgap17 |  |  | -1.51 | -1.76 |
| Atp2a3 |  |  | -1.53 | -1.54 |
| 2410025L10Rik |  |  | -1.54 | -2.14 |
| Rgl2 |  |  | -1.54 | -2.17 |
| Pip4k2b |  |  | -1.54 | -1.63 |
| Prpf38b |  |  | -1.56 | -1.98 |
| Myo9b |  |  | -1.57 | -2.63 |
| Zmym3 |  |  | -1.57 | -1.99 |
| Nisch |  |  | -1.57 | -2.33 |
| Snapc3 |  |  | -1.57 | -2.15 |
| Foxj2 |  |  | -1.58 | -1.96 |
| Zc3h7a |  |  | -1.59 | -2.12 |
| Araf |  |  | -1.6 | -2.75 |
| BC021381 |  |  | -1.61 | -3 |
| Cybasc3 |  |  | -1.63 | -1.67 |
| Elp2 |  |  | -1.63 | -2.04 |
| LOC100047369 |  |  | -1.63 | -1.74 |
| Ccdc88b |  |  | -1.64 | -2.28 |
| Psmd4 |  |  | -1.64 | -2.41 |
| Slc35b3 |  |  | -1.66 | -1.71 |
| Med23 |  |  | -1.67 | -1.76 |
| Fcgr4 |  |  | -1.68 | -2.29 |
| Mdm2 |  |  | -1.68 | -1.86 |
| Pecam1 |  |  | -1.69 | -2.61 |
| Skiv2l |  |  | -1.69 | -1.76 |
| Kctd2 |  |  | -1.7 | -1.72 |
| Kns2 |  |  | -1.7 | -1.75 |
| Ercc5 |  |  | -1.71 | -2.49 |
| Csnk1g2 |  |  | -1.71 | -2.06 |
| H2-DMb1 |  |  | -1.72 | -1.63 |
| LOC100048020 |  |  | -1.74 | -2.31 |
| Gsdmdc1 |  |  | -1.76 | -1.93 |
| Traf1 |  |  | -1.76 | -2.89 |
| Rbak |  |  | -1.77 | -1.66 |
| Mum1 |  |  | -1.77 | -2.23 |
| Pstpip1 |  |  | -1.8 | -1.52 |
| Nxf1 |  |  | -1.82 | -2.04 |
| BC057552 |  |  | -1.85 | -2.56 |
| Slc4a2 |  |  | -1.86 | -1.68 |
| 2410002F23Rik |  |  | -1.86 | -1.96 |
| Herpud1 |  |  | -1.86 | -1.9 |
| Rcsd1 |  |  | -1.87 | -2.56 |
| Rbm5 |  |  | -1.88 | -1.62 |
| Gmfg |  |  | -1.89 | -1.75 |
| Vav1 |  |  | -1.9 | -2.02 |
| Mapk11 |  |  | -1.94 | -2.05 |
| Acss1 |  |  | -1.96 | -2.08 |
| Irf1 |  |  | -1.97 | -1.66 |
| Gimap6 |  |  | -1.99 | -1.86 |
| Akap8l |  |  | -1.99 | -1.52 |
| Cyp4f13 |  |  | -2.01 | -2.92 |
| BC037034 |  |  | -2.01 | -2.13 |
| Hap1 |  |  | -2.03 | -2.37 |
| Sirpa |  |  | -2.08 | -2.99 |
| Zcchc8 |  |  | -2.12 | -1.68 |
| Slc44a2 |  |  | -2.12 | -2.27 |
| Arrdc3 |  |  | -2.13 | -1.78 |
| A430107D22Rik |  |  | -2.14 | -1.79 |
| Bat2 |  |  | -2.14 | -2.31 |
| Lysmd1 |  |  | -2.15 | -1.59 |
| Arhgap4 |  |  | -2.15 | -2.55 |
| Trit1 |  |  | -2.17 | -1.58 |
| Zfp263 |  |  | -2.19 | -1.73 |
| Mark3 |  |  | -2.2 | -1.98 |
| Gdi1 |  |  | -2.23 | -2.25 |
| 8430432M10Rik |  |  | -2.25 | -2.08 |
| 2400003C14Rik |  |  | -2.26 | -1.72 |
| Ap3m2 |  |  | -2.28 | -1.77 |
| Tagap |  |  | -2.3 | -1.67 |
| Il11ra1 |  |  | -2.33 | -1.98 |
| Tap2 |  |  | -2.34 | -3.02 |
| Snx17 |  |  | -2.41 | -1.8 |
| Dennd1c |  |  | -2.42 | -2.86 |
| Ccdc130 |  |  | -2.42 | -1.74 |
| Oasl2 |  |  | -2.44 | -2.95 |
| Epsti1 |  |  | -2.49 | -3.37 |
| Rgs1 |  |  | -2.51 | -2.54 |
| Ldb1 |  |  | -2.52 | -1.58 |
| Mterf |  |  | -2.57 | -1.96 |
| Clk4 |  |  | -2.59 | -2.04 |
| Purb |  |  | -2.6 | -2.7 |
| Mif4gd |  |  | -2.66 | -1.53 |
| Dnajc7 |  |  | -2.87 | -2.96 |
| ENSMUSG00000053178 | |  | -2.9 | -1.78 |
| 4833420G17Rik |  |  | -2.93 | -1.57 |
| Rag1ap1 |  |  | -3.18 | -1.78 |
| Sfrs16 |  |  | -3.28 | -2.95 |
| Gvin1 |  |  | -3.62 | -2.41 |
|  |  |  |  |  |
| Tubb2b |  |  | 3.19 | -1.94 |
| Prpf19 |  |  | 1.92 | -1.57 |
| Sertad2 |  |  | 1.83 | -1.51 |
| Gas6 |  |  | 1.76 | -1.65 |
| Mrps33 |  |  | -1.51 | 1.85 |
| Ednrb |  |  | -1.88 | 2.27 |
| Tram1 |  |  | -1.95 | 2.61 |
|  |  |  |  |  |
| Cxcl13 | 4.17 |  |  |  |
| Cbr2 | 3.9 |  |  |  |
| Apoc1 | 3.74 |  |  |  |
| Ifi27 | 3.58 |  |  |  |
| LOC100048346 | 3.57 |  |  |  |
| Ffar2 | 3.57 |  |  |  |
| Usp18 | 3.49 |  |  |  |
| Rsad2 | 3.45 |  |  |  |
| 9130213B05Rik | 3.18 |  |  |  |
| Pdia4 | 3.17 |  |  |  |
| H2-DMb2 | 3.06 |  |  |  |
| H2-M2 | 3.05 |  |  |  |
| Lyzs | 2.93 |  |  |  |
| 5830472M02Rik | 2.74 |  |  |  |
| Srpk3 | 2.73 |  |  |  |
| Cd274 | 2.68 |  |  |  |
| 4632417K18Rik | 2.67 |  |  |  |
| Tacstd2 | 2.65 |  |  |  |
| BC006779 | 2.64 |  |  |  |
| Socs3 | 2.6 |  |  |  |
| Snx30 | 2.57 |  |  |  |
| Eif2ak2 | 2.49 |  |  |  |
| Arhgap24 | 2.41 |  |  |  |
| Cxcr5 | 2.39 |  |  |  |
| Lst1 | 2.38 |  |  |  |
| Sh3bgrl | 2.37 |  |  |  |
| Ccr6 | 2.37 |  |  |  |
| Sfrs7 | 2.35 |  |  |  |
| Rasgrp3 | 2.33 |  |  |  |
| Des | 2.24 |  |  |  |
| Sh3bp2 | 2.2 |  |  |  |
| Abi3 | 2.12 |  |  |  |
| Lmtk2 | 2.11 |  |  |  |
| Hhex | 2.08 |  |  |  |
| Cd22 | 2.05 |  |  |  |
| Ctsc | 2.04 |  |  |  |
| Efnb1 | 1.98 |  |  |  |
| Pbrm1 | 1.93 |  |  |  |
| Tubb2c | 1.93 |  |  |  |
| Sfrs2 | 1.93 |  |  |  |
| Plac8 | 1.89 |  |  |  |
| Asb2 | 1.86 |  |  |  |
| Tdrd7 | 1.85 |  |  |  |
| Prkcb1 | 1.84 |  |  |  |
| Lxn | 1.83 |  |  |  |
| 4833426J09Rik | 1.79 |  |  |  |
| Gtf2e1 | 1.78 |  |  |  |
| Sorl1 | 1.77 |  |  |  |
| Galnt11 | 1.76 |  |  |  |
| 2310008M10Rik | 1.74 |  |  |  |
| Slc15a3 | 1.73 |  |  |  |
| Hspd1 | 1.73 |  |  |  |
| Hmgn2 | 1.73 |  |  |  |
| Hnrph1 | 1.71 |  |  |  |
| H2-Q8 | 1.71 |  |  |  |
| Tmem93 | 1.71 |  |  |  |
| Plcg2 | 1.69 |  |  |  |
| Tpi1 | 1.68 |  |  |  |
| Pfdn4 | 1.66 |  |  |  |
| Cdc42 | 1.58 |  |  |  |
| Fah | 1.58 |  |  |  |
| Plod3 | 1.58 |  |  |  |
| D3Ucla1 | 1.56 |  |  |  |
| Ccdc41 | 1.56 |  |  |  |
| Ddx54 | 1.55 |  |  |  |
| Cd40 | 1.54 |  |  |  |
| B3gnt8 | 1.53 |  |  |  |
| Dcp1a | 1.53 |  |  |  |
| Uso1 | 1.52 |  |  |  |
|  |  |  |  |  |
| Smad1 | -1.51 |  |  |  |
| Ei24 | -1.52 |  |  |  |
| Golm1 | -1.54 |  |  |  |
| Aff1 | -1.55 |  |  |  |
| Ccs | -1.56 |  |  |  |
| Gtf3c2 | -1.56 |  |  |  |
| Ramp2 | -1.58 |  |  |  |
| Zfyve21 | -1.6 |  |  |  |
| Acaa2 | -1.61 |  |  |  |
| Wdr45 | -1.61 |  |  |  |
| Ankle2 | -1.62 |  |  |  |
| Ccl9 | -1.62 |  |  |  |
| Centd3 | -1.63 |  |  |  |
| Fbxo34 | -1.66 |  |  |  |
| Fxyd6 | -1.67 |  |  |  |
| Ascc1 | -1.67 |  |  |  |
| Cyhr1 | -1.68 |  |  |  |
| Igf2r | -1.68 |  |  |  |
| Mmrn2 | -1.7 |  |  |  |
| Srd5a3 | -1.71 |  |  |  |
| Fads1 | -1.71 |  |  |  |
| Usp3 | -1.72 |  |  |  |
| Mfap3 | -1.75 |  |  |  |
| C2 | -1.75 |  |  |  |
| Leprotl1 | -1.77 |  |  |  |
| Adcy4 | -1.77 |  |  |  |
| Sh3tc1 | -1.78 |  |  |  |
| Cenpa | -1.79 |  |  |  |
| Egfl7 | -1.79 |  |  |  |
| Ppic | -1.81 |  |  |  |
| Hspb6 | -1.81 |  |  |  |
| Gimap5 | -1.86 |  |  |  |
| Meis2 | -1.86 |  |  |  |
| Centb5 | -1.88 |  |  |  |
| 1810020D17Rik | -1.88 |  |  |  |
| 0610007C21Rik | -1.88 |  |  |  |
| Selplg | -1.9 |  |  |  |
| Gltp | -1.91 |  |  |  |
| Chka | -1.91 |  |  |  |
| Grasp | -1.92 |  |  |  |
| Cish | -1.94 |  |  |  |
| Ptprb | -1.97 |  |  |  |
| Hdac7 | -2 |  |  |  |
| Fntb | -2.05 |  |  |  |
| Nupr1 | -2.07 |  |  |  |
| Ldb2 | -2.07 |  |  |  |
| Ramp1 | -2.07 |  |  |  |
| Tgfbr3 | -2.08 |  |  |  |
| Cyp2d22 | -2.13 |  |  |  |
| Bok | -2.15 |  |  |  |
| Timp2 | -2.16 |  |  |  |
| Pdk1 | -2.32 |  |  |  |
| Fcgrt | -2.33 |  |  |  |
| 6030443O07Rik | -2.36 |  |  |  |
| Lrig1 | -2.37 |  |  |  |
| Nrp1 | -2.42 |  |  |  |
| Dhrs7 | -2.51 |  |  |  |
| Abhd14b | -2.52 |  |  |  |
| Snrk | -2.53 |  |  |  |
| Timp3 | -2.59 |  |  |  |
| Dnajc15 | -2.61 |  |  |  |
| X99384 | -2.63 |  |  |  |
| Slc25a20 | -2.66 |  |  |  |
| Appl2 | -2.69 |  |  |  |
| Stard8 | -2.72 |  |  |  |
| Dctn6 | -2.72 |  |  |  |
| H6pd | -2.88 |  |  |  |
| Cyp27a1 | -3 |  |  |  |
| Robo4 | -3.08 |  |  |  |
| Tpcn1 | -3.26 |  |  |  |
| Ankrd47 | -3.27 |  |  |  |
| Cldn5 | -3.32 |  |  |  |
| Ltbp4 | -3.34 |  |  |  |
| Cdkn1c | -3.35 |  |  |  |
| Asah3l | -3.59 |  |  |  |
| Tle1 | -3.71 |  |  |  |
| Hdc | -3.75 |  |  |  |
| Enpp5 | -4.18 |  |  |  |
| Reln | -4.3 |  |  |  |
| S100a9 | -4.4 |  |  |  |
| Igf2 | -4.46 |  |  |  |
| Klf9 | -5.21 |  |  |  |
| Angptl4 | -5.53 |  |  |  |
| Gpx3 | -5.67 |  |  |  |
| Cyp2f2 | -11.16 |  |  |  |
|  |  |  |  |  |
| Emr1 |  | 5.64 |  |  |
| Hpgd |  | 3.76 |  |  |
| Srgn |  | 3.44 |  |  |
| Serpina3f |  | 3.33 |  |  |
| Sc4mol |  | 3.26 |  |  |
| Flrt3 |  | 3.23 |  |  |
| Gbp3 |  | 3.23 |  |  |
| Id2 |  | 3.01 |  |  |
| Txn1 |  | 2.99 |  |  |
| Cp |  | 2.92 |  |  |
| Atp5a1 |  | 2.85 |  |  |
| Ibtk |  | 2.57 |  |  |
| 1110002B05Rik |  | 2.57 |  |  |
| 2700055A20Rik |  | 2.55 |  |  |
| LOC100048721 |  | 2.54 |  |  |
| Cyb5 |  | 2.53 |  |  |
| Furin |  | 2.5 |  |  |
| Cox6c |  | 2.48 |  |  |
| Igsf4a |  | 2.47 |  |  |
| Col18a1 |  | 2.45 |  |  |
| Prdx5 |  | 2.39 |  |  |
| P2ry5 |  | 2.39 |  |  |
| Chchd7 |  | 2.36 |  |  |
| Pdgfa |  | 2.35 |  |  |
| Nab1 |  | 2.32 |  |  |
| Itm2b |  | 2.3 |  |  |
| Loh11cr2a |  | 2.28 |  |  |
| Lhfp |  | 2.25 |  |  |
| Hnrpdl |  | 2.23 |  |  |
| Atp6v1a |  | 2.2 |  |  |
| Rbp1 |  | 2.11 |  |  |
| Idh2 |  | 2.09 |  |  |
| Agtrap |  | 2.09 |  |  |
| Atp10d |  | 2.08 |  |  |
| Eif4e3 |  | 2.07 |  |  |
| LOC547343 |  | 2.01 |  |  |
| Myo6 |  | 2 |  |  |
| Hspe1 |  | 1.97 |  |  |
| Rbms3 |  | 1.94 |  |  |
| Csf1r |  | 1.89 |  |  |
| Rbm47 |  | 1.89 |  |  |
| Picalm |  | 1.87 |  |  |
| Nuak1 |  | 1.86 |  |  |
| Pja2 |  | 1.85 |  |  |
| Eed |  | 1.84 |  |  |
| Slc2a1 |  | 1.83 |  |  |
| Prkra |  | 1.83 |  |  |
| Ogt |  | 1.8 |  |  |
| Atp5h |  | 1.8 |  |  |
| Atg5 |  | 1.8 |  |  |
| Pdpk1 |  | 1.79 |  |  |
| 3110050N22Rik |  | 1.78 |  |  |
| Tmem55a |  | 1.77 |  |  |
| Lman1 |  | 1.77 |  |  |
| Iigp2 |  | 1.77 |  |  |
| Evi2a |  | 1.74 |  |  |
| LOC100044177 |  | 1.71 |  |  |
| Dusp19 |  | 1.71 |  |  |
| Lgals8 |  | 1.71 |  |  |
| Acadsb |  | 1.71 |  |  |
| Snap29 |  | 1.7 |  |  |
| Fez2 |  | 1.7 |  |  |
| 4930572J05Rik |  | 1.7 |  |  |
| Cyp4v3 |  | 1.69 |  |  |
| Gosr2 |  | 1.68 |  |  |
| Btbd14a |  | 1.66 |  |  |
| Selk |  | 1.66 |  |  |
| Slc25a17 |  | 1.66 |  |  |
| D9Wsu20e |  | 1.66 |  |  |
| Ebpl |  | 1.66 |  |  |
| Pigp |  | 1.65 |  |  |
| Ube3c |  | 1.64 |  |  |
| 3110009E18Rik |  | 1.63 |  |  |
| Tax1bp1 |  | 1.63 |  |  |
| Zdhhc21 |  | 1.63 |  |  |
| 2310056P07Rik |  | 1.62 |  |  |
| Atxn1 |  | 1.61 |  |  |
| Osbpl2 |  | 1.61 |  |  |
| Ddx3x |  | 1.61 |  |  |
| Nhlrc2 |  | 1.6 |  |  |
| LOC100046775 |  | 1.56 |  |  |
| Nfib |  | 1.56 |  |  |
| Supt4h1 |  | 1.56 |  |  |
| Dcun1d5 |  | 1.56 |  |  |
| Nck1 |  | 1.54 |  |  |
| Ankrd10 |  | 1.54 |  |  |
| Rbbp9 |  | 1.53 |  |  |
| Fnbp1l |  | 1.52 |  |  |
| Cnpy2 |  | 1.52 |  |  |
|  |  |  |  |  |
| Epc1 |  | -1.5 |  |  |
| LOC100044829 |  | -1.5 |  |  |
| Setd1a |  | -1.51 |  |  |
| Actr1a |  | -1.52 |  |  |
| Bax |  | -1.54 |  |  |
| Cnot4 |  | -1.54 |  |  |
| Ebi2 |  | -1.56 |  |  |
| Gart |  | -1.6 |  |  |
| 5430437P03Rik |  | -1.6 |  |  |
| Sf3b2 |  | -1.63 |  |  |
| Pip5k1a |  | -1.64 |  |  |
| Irs2 |  | -1.64 |  |  |
| Uba1 |  | -1.66 |  |  |
| Zmynd11 |  | -1.67 |  |  |
| D4Ertd22e |  | -1.69 |  |  |
| Samsn1 |  | -1.69 |  |  |
| Ogdh |  | -1.7 |  |  |
| Sbds |  | -1.72 |  |  |
| Abcf1 |  | -1.72 |  |  |
| Pafah1b3 |  | -1.73 |  |  |
| 2310036O22Rik |  | -1.74 |  |  |
| Rbm38 |  | -1.79 |  |  |
| Dync1h1 |  | -1.83 |  |  |
| Eif3i |  | -1.85 |  |  |
| 6430510M02Rik |  | -1.87 |  |  |
| Nfkbia |  | -1.87 |  |  |
| Mrps34 |  | -1.91 |  |  |
| Ypel3 |  | -1.98 |  |  |
| E330018D03Rik |  | -1.99 |  |  |
| D930015E06Rik |  | -1.99 |  |  |
| 1700021K19Rik |  | -1.99 |  |  |
| Chst3 |  | -2 |  |  |
| Rpo1-3 |  | -2.04 |  |  |
| Xrcc1 |  | -2.05 |  |  |
| Ep300 |  | -2.07 |  |  |
| Cox7a2l |  | -2.07 |  |  |
| Tmem42 |  | -2.08 |  |  |
| Lsm12 |  | -2.08 |  |  |
| Necap2 |  | -2.09 |  |  |
| Pla2g12a |  | -2.13 |  |  |
| Lgals3 |  | -2.19 |  |  |
| Rnf144a |  | -2.19 |  |  |
| Dusp2 |  | -2.21 |  |  |
| Ppie |  | -2.24 |  |  |
| Ptp4a3 |  | -2.25 |  |  |
| Zfp592 |  | -2.46 |  |  |
| Mysm1 |  | -2.74 |  |  |
| LOC100046039 |  | -2.88 |  |  |
| LOC100043257 |  | -3.25 |  |  |
| Zbtb7a |  | -6.57 |  |  |
|  |  |  |  |  |
| Aqp1 |  |  | 5.14 |  |
| Gpnmb |  |  | 4.9 |  |
| Hist1h2bj |  |  | 4.89 |  |
| Hist1h2bf |  |  | 4.82 |  |
| Hist1h2bh |  |  | 4.77 |  |
| Hist1h2ah |  |  | 4.76 |  |
| Hist1h2ag |  |  | 4.23 |  |
| Hist1h2ad |  |  | 4.15 |  |
| Lmna |  |  | 4.06 |  |
| Actn4 |  |  | 3.73 |  |
| Lgmn |  |  | 3.73 |  |
| Pld3 |  |  | 3.57 |  |
| Tnfaip2 |  |  | 3.43 |  |
| 9/15/2013 0:00 |  |  | 3.31 |  |
| LOC100044204 |  |  | 3.28 |  |
| Mpp1 |  |  | 3.2 |  |
| Tbc1d20 |  |  | 3.02 |  |
| Pcyt1a |  |  | 3.02 |  |
| Enpp4 |  |  | 2.97 |  |
| Cd63 |  |  | 2.94 |  |
| C1qb |  |  | 2.84 |  |
| Ezh2 |  |  | 2.77 |  |
| Ly6c1 |  |  | 2.72 |  |
| Cdca3 |  |  | 2.71 |  |
| Tmem205 |  |  | 2.69 |  |
| H13 |  |  | 2.66 |  |
| Cd209b |  |  | 2.63 |  |
| Hist1h2bk |  |  | 2.63 |  |
| Ppm1g |  |  | 2.63 |  |
| Rpl38 |  |  | 2.6 |  |
| Mrpl53 |  |  | 2.58 |  |
| Hist1h2af |  |  | 2.58 |  |
| Fxn |  |  | 2.56 |  |
| Mcm2 |  |  | 2.55 |  |
| Capzb |  |  | 2.52 |  |
| Xpnpep1 |  |  | 2.46 |  |
| Snrpd2 |  |  | 2.46 |  |
| Nde1 |  |  | 2.46 |  |
| Cd97 |  |  | 2.39 |  |
| Mboat5 |  |  | 2.35 |  |
| Tmem2 |  |  | 2.35 |  |
| Map3k3 |  |  | 2.3 |  |
| Cygb |  |  | 2.29 |  |
| Plp2 |  |  | 2.28 |  |
| Bckdk |  |  | 2.28 |  |
| LOC100041835 |  |  | 2.28 |  |
| Fermt3 |  |  | 2.27 |  |
| Sumo3 |  |  | 2.27 |  |
| Psmb2 |  |  | 2.25 |  |
| C1qa |  |  | 2.24 |  |
| Samd14 |  |  | 2.23 |  |
| Psma7 |  |  | 2.22 |  |
| D8Ertd738e |  |  | 2.2 |  |
| Vps25 |  |  | 2.19 |  |
| Galnt1 |  |  | 2.18 |  |
| Tmem9 |  |  | 2.16 |  |
| Eng |  |  | 2.15 |  |
| Yif1b |  |  | 2.15 |  |
| Hip2 |  |  | 2.15 |  |
| Slco3a1 |  |  | 2.14 |  |
| Ak2 |  |  | 2.12 |  |
| Hist1h2an |  |  | 2.11 |  |
| 2510006D16Rik |  |  | 2.09 |  |
| 5730593F17Rik |  |  | 2.09 |  |
| Srm |  |  | 2.08 |  |
| Nucb1 |  |  | 2.08 |  |
| Tnfrsf21 |  |  | 2.08 |  |
| Tfdp1 |  |  | 2.07 |  |
| Rpl7a |  |  | 2.06 |  |
| Ilk |  |  | 2.05 |  |
| 1190002H23Rik |  |  | 2.05 |  |
| Mrpl34 |  |  | 2.02 |  |
| 1810009A15Rik |  |  | 2.01 |  |
| Trappc1 |  |  | 2.01 |  |
| BC017647 |  |  | 2 |  |
| Akap12 |  |  | 1.98 |  |
| Stard10 |  |  | 1.97 |  |
| Rpl24 |  |  | 1.96 |  |
| Bcap31 |  |  | 1.96 |  |
| 4933434E20Rik |  |  | 1.95 |  |
| Tmem204 |  |  | 1.95 |  |
| Gde1 |  |  | 1.95 |  |
| Nomo1 |  |  | 1.94 |  |
| LOC100046650 |  |  | 1.94 |  |
| Calu |  |  | 1.94 |  |
| Chmp2a |  |  | 1.94 |  |
| Tbc1d2b |  |  | 1.93 |  |
| Mrps28 |  |  | 1.93 |  |
| App |  |  | 1.91 |  |
| Ssna1 |  |  | 1.9 |  |
| Cope |  |  | 1.89 |  |
| Eif4a1 |  |  | 1.88 |  |
| Polr2f |  |  | 1.87 |  |
| Papola |  |  | 1.87 |  |
| Fbxo9 |  |  | 1.87 |  |
| 2310022B05Rik |  |  | 1.85 |  |
| Ap2s1 |  |  | 1.83 |  |
| Cmtm7 |  |  | 1.82 |  |
| Pfkm |  |  | 1.82 |  |
| 1500032L24Rik |  |  | 1.81 |  |
| Plvap |  |  | 1.8 |  |
| Cox17 |  |  | 1.79 |  |
| Atp6v0d1 |  |  | 1.79 |  |
| Ruvbl2 |  |  | 1.79 |  |
| Asna1 |  |  | 1.78 |  |
| Ppap2a |  |  | 1.78 |  |
| Gtf3a |  |  | 1.77 |  |
| 3/2/2013 0:00 |  |  | 1.76 |  |
| Tmem86a |  |  | 1.76 |  |
| Clcn3 |  |  | 1.75 |  |
| Bud31 |  |  | 1.75 |  |
| Map2k3 |  |  | 1.75 |  |
| Cdkn2c |  |  | 1.74 |  |
| Ncapd3 |  |  | 1.74 |  |
| Gse1 |  |  | 1.73 |  |
| Cdt1 |  |  | 1.72 |  |
| Fbxo42 |  |  | 1.72 |  |
| LOC100044294 |  |  | 1.72 |  |
| Dguok |  |  | 1.72 |  |
| Apoa1bp |  |  | 1.71 |  |
| Smarcb1 |  |  | 1.7 |  |
| Bnip2 |  |  | 1.7 |  |
| Ngfrap1 |  |  | 1.69 |  |
| Stim1 |  |  | 1.69 |  |
| Exosc10 |  |  | 1.68 |  |
| 0610006I08Rik |  |  | 1.67 |  |
| Sae1 |  |  | 1.66 |  |
| Fen1 |  |  | 1.65 |  |
| Maged2 |  |  | 1.65 |  |
| Snx15 |  |  | 1.63 |  |
| Prmt5 |  |  | 1.61 |  |
| Zfand2a |  |  | 1.6 |  |
| Ssr2 |  |  | 1.59 |  |
| Snrpa |  |  | 1.58 |  |
| Sdf2 |  |  | 1.57 |  |
| Eif2ak1 |  |  | 1.56 |  |
| Stip1 |  |  | 1.56 |  |
| Elovl1 |  |  | 1.55 |  |
| Rsl1d1 |  |  | 1.54 |  |
| Suclg2 |  |  | 1.54 |  |
| Fermt2 |  |  | 1.53 |  |
| Wdr1 |  |  | 1.51 |  |
| Tyrobp |  |  | 1.5 |  |
|  |  |  |  |  |
| Hnrpl |  |  | -1.5 |  |
| Vamp2 |  |  | -1.5 |  |
| Mrps6 |  |  | -1.51 |  |
| Trim26 |  |  | -1.51 |  |
| Polr2h |  |  | -1.51 |  |
| Sfrs6 |  |  | -1.51 |  |
| Rpl22 |  |  | -1.52 |  |
| Elf1 |  |  | -1.52 |  |
| LOC100041504 |  |  | -1.53 |  |
| Nol6 |  |  | -1.53 |  |
| M6pr |  |  | -1.53 |  |
| Mrpl24 |  |  | -1.53 |  |
| Wdr51b |  |  | -1.53 |  |
| Vps54 |  |  | -1.54 |  |
| Arf2 |  |  | -1.54 |  |
| Znrd1 |  |  | -1.54 |  |
| Trappc4 |  |  | -1.54 |  |
| Panx1 |  |  | -1.55 |  |
| Atp5sl |  |  | -1.55 |  |
| Tpd52 |  |  | -1.55 |  |
| Mapre2 |  |  | -1.56 |  |
| H2-T23 |  |  | -1.56 |  |
| LOC100045300 |  |  | -1.56 |  |
| Il10ra |  |  | -1.57 |  |
| Wdr68 |  |  | -1.57 |  |
| Manba |  |  | -1.57 |  |
| Slc22a5 |  |  | -1.58 |  |
| Rab2a |  |  | -1.58 |  |
| Nras |  |  | -1.58 |  |
| Dpp4 |  |  | -1.59 |  |
| LOC100046996 |  |  | -1.59 |  |
| Wdsof1 |  |  | -1.59 |  |
| Add3 |  |  | -1.59 |  |
| Soat1 |  |  | -1.59 |  |
| LOC100044776 |  |  | -1.6 |  |
| LOC100047167 |  |  | -1.61 |  |
| Dennd3 |  |  | -1.61 |  |
| Ikzf1 |  |  | -1.61 |  |
| Smc1a |  |  | -1.61 |  |
| Dpysl2 |  |  | -1.61 |  |
| AI451557 |  |  | -1.61 |  |
| LOC621823 |  |  | -1.61 |  |
| Scotin |  |  | -1.62 |  |
| Zcchc18 |  |  | -1.62 |  |
| Eml4 |  |  | -1.62 |  |
| BC013529 |  |  | -1.62 |  |
| Serpina3n |  |  | -1.62 |  |
| Pex19 |  |  | -1.62 |  |
| Adrb2 |  |  | -1.63 |  |
| Lpxn |  |  | -1.64 |  |
| Dusp11 |  |  | -1.65 |  |
| Pdlim4 |  |  | -1.65 |  |
| Zfp36 |  |  | -1.65 |  |
| LOC100044538 |  |  | -1.65 |  |
| Cdk5rap3 |  |  | -1.65 |  |
| Sh3bgrl3 |  |  | -1.66 |  |
| Kcnab2 |  |  | -1.66 |  |
| Arl1 |  |  | -1.66 |  |
| LOC100046891 |  |  | -1.67 |  |
| Trip12 |  |  | -1.67 |  |
| Pih1d1 |  |  | -1.67 |  |
| Snn |  |  | -1.67 |  |
| Hn1 |  |  | -1.68 |  |
| Txndc15 |  |  | -1.68 |  |
| Cdc2l6 |  |  | -1.69 |  |
| Samhd1 |  |  | -1.69 |  |
| LOC100048105 |  |  | -1.69 |  |
| Pop5 |  |  | -1.7 |  |
| Ctnnb1 |  |  | -1.7 |  |
| D19Wsu162e |  |  | -1.7 |  |
| Pigx |  |  | -1.7 |  |
| Drbp1 |  |  | -1.7 |  |
| Il4i1 |  |  | -1.71 |  |
| Anxa11 |  |  | -1.72 |  |
| LOC100045887 |  |  | -1.72 |  |
| Peli1 |  |  | -1.72 |  |
| Nfyc |  |  | -1.72 |  |
| F2r |  |  | -1.72 |  |
| BC087945 |  |  | -1.73 |  |
| Tmem184b |  |  | -1.73 |  |
| Asb8 |  |  | -1.73 |  |
| Stx12 |  |  | -1.73 |  |
| Morc3 |  |  | -1.74 |  |
| Pgs1 |  |  | -1.74 |  |
| Gimap1 |  |  | -1.75 |  |
| BC031353 |  |  | -1.75 |  |
| Zfp608 |  |  | -1.75 |  |
| Rab33b |  |  | -1.75 |  |
| Rnf103 |  |  | -1.76 |  |
| Cog3 |  |  | -1.77 |  |
| Bcl6 |  |  | -1.77 |  |
| Hgsnat |  |  | -1.77 |  |
| Tspan32 |  |  | -1.77 |  |
| Inpp5d |  |  | -1.78 |  |
| Stx5a |  |  | -1.78 |  |
| Emg1 |  |  | -1.79 |  |
| LOC100047834 |  |  | -1.8 |  |
| Ccdc85b |  |  | -1.8 |  |
| Traf6 |  |  | -1.8 |  |
| Ddx21 |  |  | -1.81 |  |
| Limd1 |  |  | -1.81 |  |
| Tusc4 |  |  | -1.82 |  |
| Cwf19l2 |  |  | -1.82 |  |
| Cbfb |  |  | -1.82 |  |
| Cdc42se1 |  |  | -1.83 |  |
| Tctex1d2 |  |  | -1.84 |  |
| Skap2 |  |  | -1.85 |  |
| Elavl1 |  |  | -1.85 |  |
| Zbtb33 |  |  | -1.85 |  |
| Elovl5 |  |  | -1.85 |  |
| Fubp3 |  |  | -1.85 |  |
| Rasgrp1 |  |  | -1.86 |  |
| 4121402D02Rik |  |  | -1.86 |  |
| Tmed9 |  |  | -1.87 |  |
| 0610010E21Rik |  |  | -1.88 |  |
| Gorasp2 |  |  | -1.88 |  |
| 2500003M10Rik |  |  | -1.88 |  |
| Crlf3 |  |  | -1.88 |  |
| Zc3h12d |  |  | -1.89 |  |
| Ccdc100 |  |  | -1.9 |  |
| Glt25d1 |  |  | -1.9 |  |
| Ctcf |  |  | -1.91 |  |
| Uspl1 |  |  | -1.92 |  |
| Vamp4 |  |  | -1.93 |  |
| Sla |  |  | -1.93 |  |
| Zfp292 |  |  | -1.93 |  |
| Hexb |  |  | -1.94 |  |
| Mreg |  |  | -1.95 |  |
| Polr2g |  |  | -1.95 |  |
| Sidt1 |  |  | -1.96 |  |
| 1700047I17Rik1 |  |  | -1.96 |  |
| Pdgfra |  |  | -1.96 |  |
| Zfp512 |  |  | -1.97 |  |
| 1500001L15Rik |  |  | -1.97 |  |
| Ate1 |  |  | -1.99 |  |
| Arglu1 |  |  | -1.99 |  |
| Errfi1 |  |  | -1.99 |  |
| B230342M21Rik |  |  | -2 |  |
| Stt3b |  |  | -2 |  |
| Mettl3 |  |  | -2.02 |  |
| Zfp597 |  |  | -2.02 |  |
| Stk4 |  |  | -2.02 |  |
| Tigd2 |  |  | -2.03 |  |
| Rassf3 |  |  | -2.03 |  |
| Snx2 |  |  | -2.03 |  |
| A230050P20Rik |  |  | -2.04 |  |
| Rasa1 |  |  | -2.04 |  |
| Spop |  |  | -2.04 |  |
| Rabgap1 |  |  | -2.05 |  |
| Snx8 |  |  | -2.05 |  |
| Mfng |  |  | -2.05 |  |
| Sf4 |  |  | -2.06 |  |
| Cstf2 |  |  | -2.06 |  |
| D6Wsu176e |  |  | -2.07 |  |
| Gcc2 |  |  | -2.07 |  |
| Lsm14a |  |  | -2.08 |  |
| LOC100046120 |  |  | -2.08 |  |
| Myh9 |  |  | -2.09 |  |
| Plekha2 |  |  | -2.09 |  |
| Btbd1 |  |  | -2.1 |  |
| Abi1 |  |  | -2.11 |  |
| Suv420h1 |  |  | -2.13 |  |
| Fyb |  |  | -2.13 |  |
| Gpr171 |  |  | -2.13 |  |
| Ubl3 |  |  | -2.13 |  |
| Nuak2 |  |  | -2.15 |  |
| Clec2d |  |  | -2.16 |  |
| 1110003E01Rik |  |  | -2.16 |  |
| Tk2 |  |  | -2.16 |  |
| Spag9 |  |  | -2.16 |  |
| Csnk1g3 |  |  | -2.18 |  |
| 4921505C17Rik |  |  | -2.18 |  |
| Evl |  |  | -2.19 |  |
| Rab3gap1 |  |  | -2.19 |  |
| Jakmip1 |  |  | -2.22 |  |
| Atp1b3 |  |  | -2.23 |  |
| Wsb1 |  |  | -2.23 |  |
| Aftph |  |  | -2.25 |  |
| Prkar1a |  |  | -2.27 |  |
| AI314180 |  |  | -2.27 |  |
| Ing4 |  |  | -2.28 |  |
| Rpl36a |  |  | -2.29 |  |
| Plekhm1 |  |  | -2.29 |  |
| Fchsd2 |  |  | -2.3 |  |
| Smarca2 |  |  | -2.31 |  |
| Lat2 |  |  | -2.31 |  |
| Hnrpm |  |  | -2.34 |  |
| Cyld |  |  | -2.36 |  |
| Gns |  |  | -2.36 |  |
| LOC100048845 |  |  | -2.39 |  |
| Armc10 |  |  | -2.4 |  |
| Bclaf1 |  |  | -2.41 |  |
| 2700060E02Rik |  |  | -2.43 |  |
| Fgfr1op2 |  |  | -2.45 |  |
| Sell |  |  | -2.45 |  |
| Klf2 |  |  | -2.45 |  |
| H2-Ke6 |  |  | -2.46 |  |
| LOC100047261 |  |  | -2.46 |  |
| St6gal1 |  |  | -2.47 |  |
| Ets1 |  |  | -2.48 |  |
| Ahnak |  |  | -2.48 |  |
| Sdc4 |  |  | -2.49 |  |
| Sbk |  |  | -2.51 |  |
| Herc4 |  |  | -2.51 |  |
| Rap2c |  |  | -2.53 |  |
| Foxj3 |  |  | -2.57 |  |
| Rps25 |  |  | -2.59 |  |
| Mcl1 |  |  | -2.59 |  |
| Nfkbid |  |  | -2.62 |  |
| Tmem50a |  |  | -2.63 |  |
| Trp53inp1 |  |  | -2.64 |  |
| Ccl21b |  |  | -2.65 |  |
| Arhgef3 |  |  | -2.68 |  |
| Egr1 |  |  | -2.69 |  |
| Tle4 |  |  | -2.72 |  |
| Pptc7 |  |  | -2.75 |  |
| Dazap2 |  |  | -2.77 |  |
| Skil |  |  | -2.8 |  |
| Trub2 |  |  | -2.82 |  |
| Rnase6 |  |  | -2.85 |  |
| St8sia4 |  |  | -2.87 |  |
| Tnfaip3 |  |  | -2.88 |  |
| Satb1 |  |  | -2.91 |  |
| Xlr4a |  |  | -2.93 |  |
| Stk17b |  |  | -2.95 |  |
| Thy1 |  |  | -2.99 |  |
| Plekha1 |  |  | -3.01 |  |
| C030048B08Rik |  |  | -3.01 |  |
| Faim3 |  |  | -3.02 |  |
| Dusp6 |  |  | -3.02 |  |
| Cd53 |  |  | -3.03 |  |
| Eef1b2 |  |  | -3.04 |  |
| H2-Aa |  |  | -3.06 |  |
| Arhgap30 |  |  | -3.07 |  |
| Pscdbp |  |  | -3.07 |  |
| A730098D12Rik |  |  | -3.1 |  |
| Tbc1d10c |  |  | -3.11 |  |
| Slc12a2 |  |  | -3.11 |  |
| Pkig |  |  | -3.16 |  |
| Gimap8 |  |  | -3.17 |  |
| Btg1 |  |  | -3.18 |  |
| Uap1 |  |  | -3.25 |  |
| LOC433801 |  |  | -3.28 |  |
| Map3k1 |  |  | -3.41 |  |
| Cd69 |  |  | -3.58 |  |
| Chfr |  |  | -3.72 |  |
| Lbh |  |  | -3.86 |  |
| Ccl5 |  |  | -4.29 |  |
| Adam23 |  |  | -4.86 |  |
| Serinc3 |  |  | -5.15 |  |
|  |  |  |  |  |
| Rasl12 |  |  |  | 5.74 |
| Cbr3 |  |  |  | 5.04 |
| Cdo1 |  |  |  | 5.02 |
| S100a8 |  |  |  | 4.7 |
| Ndg2 |  |  |  | 4.59 |
| Pygl |  |  |  | 4.39 |
| Pnpla2 |  |  |  | 4.24 |
| Cd59a |  |  |  | 4.23 |
| LOC100044190 |  |  |  | 4.13 |
| Dbi |  |  |  | 3.7 |
| Eif4ebp1 |  |  |  | 3.56 |
| Gja1 |  |  |  | 3.53 |
| Sdhb |  |  |  | 3.42 |
| 1700037H04Rik |  |  |  | 3.39 |
| Prelp |  |  |  | 3.33 |
| Sdcbp |  |  |  | 3.22 |
| Pfkl |  |  |  | 3.08 |
| Scp2 |  |  |  | 2.9 |
| Pdha1 |  |  |  | 2.66 |
| Ube2g1 |  |  |  | 2.63 |
| Mcm6 |  |  |  | 2.6 |
| Acly |  |  |  | 2.59 |
| Gstk1 |  |  |  | 2.59 |
| Sypl |  |  |  | 2.56 |
| Suclg1 |  |  |  | 2.51 |
| Acss2 |  |  |  | 2.5 |
| Nme2 |  |  |  | 2.5 |
| Isca1 |  |  |  | 2.48 |
| Acadm |  |  |  | 2.44 |
| LOC100047353 |  |  |  | 2.41 |
| Sod2 |  |  |  | 2.41 |
| Mmd |  |  |  | 2.39 |
| Pnrc2 |  |  |  | 2.37 |
| Mgll |  |  |  | 2.36 |
| Cox5a |  |  |  | 2.34 |
| Ndufa4 |  |  |  | 2.31 |
| 1110001J03Rik |  |  |  | 2.28 |
| Bxdc2 |  |  |  | 2.28 |
| LOC654426 |  |  |  | 2.28 |
| Drg1 |  |  |  | 2.23 |
| Echs1 |  |  |  | 2.23 |
| Eif3s10 |  |  |  | 2.21 |
| Rnaset2 |  |  |  | 2.21 |
| Ndufa9 |  |  |  | 2.16 |
| Txnl4 |  |  |  | 2.15 |
| Arhgap29 |  |  |  | 2.12 |
| Brp17 |  |  |  | 2.11 |
| Aldoa |  |  |  | 2.1 |
| Serpinf1 |  |  |  | 2.1 |
| Tmem38b |  |  |  | 2.08 |
| 1500032D16Rik |  |  |  | 2.04 |
| Gabarap |  |  |  | 2.04 |
| Chmp1b |  |  |  | 2.01 |
| Fundc1 |  |  |  | 2 |
| B230219D22Rik |  |  |  | 1.99 |
| Slc25a5 |  |  |  | 1.96 |
| Cs |  |  |  | 1.95 |
| Tgfbr2 |  |  |  | 1.94 |
| Commd1 |  |  |  | 1.93 |
| Tipin |  |  |  | 1.93 |
| Mdh2 |  |  |  | 1.92 |
| Zeb1 |  |  |  | 1.91 |
| Hspa9 |  |  |  | 1.9 |
| Prkaca |  |  |  | 1.88 |
| 1110008P14Rik |  |  |  | 1.84 |
| 1200003C05Rik |  |  |  | 1.82 |
| Atp5l |  |  |  | 1.82 |
| Rnf167 |  |  |  | 1.81 |
| Ppm1a |  |  |  | 1.8 |
| Hmox2 |  |  |  | 1.78 |
| Gpx1 |  |  |  | 1.77 |
| Tcp1 |  |  |  | 1.76 |
| Hbp1 |  |  |  | 1.74 |
| Mrps18b |  |  |  | 1.74 |
| Csrp2 |  |  |  | 1.72 |
| Atp5c1 |  |  |  | 1.71 |
| Ghitm |  |  |  | 1.71 |
| Tomm7 |  |  |  | 1.71 |
| LOC100046393 |  |  |  | 1.7 |
| l7Rn6 |  |  |  | 1.68 |
| Tmem50b |  |  |  | 1.67 |
| 2010316F05Rik |  |  |  | 1.66 |
| Tmem59 |  |  |  | 1.66 |
| Lin54 |  |  |  | 1.65 |
| Mrpl30 |  |  |  | 1.65 |
| Dynll1 |  |  |  | 1.64 |
| Magoh |  |  |  | 1.64 |
| LOC668492 |  |  |  | 1.62 |
| Atp5j |  |  |  | 1.61 |
| Hsd17b4 |  |  |  | 1.61 |
| Hprt1 |  |  |  | 1.6 |
| Csde1 |  |  |  | 1.59 |
| Ormdl3 |  |  |  | 1.59 |
| Rab35 |  |  |  | 1.58 |
| Scoc |  |  |  | 1.58 |
| Cyb5r4 |  |  |  | 1.57 |
| Tmem126a |  |  |  | 1.57 |
| Ccdc47 |  |  |  | 1.56 |
| Use1 |  |  |  | 1.55 |
| Nme3 |  |  |  | 1.54 |
| Plekhf2 |  |  |  | 1.54 |
| Rnps1 |  |  |  | 1.54 |
| Rps2 |  |  |  | 1.54 |
| Atp6v0e |  |  |  | 1.53 |
| Sqstm1 |  |  |  | 1.53 |
| Fkbp4 |  |  |  | 1.52 |
| Orc6l |  |  |  | 1.52 |
| Tufm |  |  |  | 1.51 |
|  |  |  |  |  |
| Prf1 |  |  |  | -1.5 |
| 5133401N09Rik |  |  |  | -1.51 |
| Mecp2 |  |  |  | -1.51 |
| Atg16l1 |  |  |  | -1.52 |
| Hdgfrp2 |  |  |  | -1.52 |
| Lias |  |  |  | -1.52 |
| Pigo |  |  |  | -1.52 |
| Unc84b |  |  |  | -1.52 |
| 1810008A18Rik |  |  |  | -1.53 |
| Dctn5 |  |  |  | -1.53 |
| Ier3 |  |  |  | -1.53 |
| Luzp1 |  |  |  | -1.53 |
| Napg |  |  |  | -1.53 |
| Prcp |  |  |  | -1.53 |
| Tnrc6c |  |  |  | -1.53 |
| Trp53bp1 |  |  |  | -1.53 |
| Whsc2 |  |  |  | -1.53 |
| Zbtb17 |  |  |  | -1.53 |
| Clk2 |  |  |  | -1.54 |
| Usp20 |  |  |  | -1.54 |
| Gapvd1 |  |  |  | -1.55 |
| Ndrg1 |  |  |  | -1.55 |
| Prep |  |  |  | -1.55 |
| Rnf8 |  |  |  | -1.55 |
| Statip1 |  |  |  | -1.55 |
| D11Wsu47e |  |  |  | -1.56 |
| Krtcap3 |  |  |  | -1.56 |
| Pick1 |  |  |  | -1.56 |
| Tmem63a |  |  |  | -1.56 |
| Utx |  |  |  | -1.56 |
| Zc3h18 |  |  |  | -1.56 |
| 3300001P08Rik |  |  |  | -1.57 |
| Nagk |  |  |  | -1.57 |
| Pld4 |  |  |  | -1.57 |
| Ubp1 |  |  |  | -1.57 |
| 1810027O10Rik |  |  |  | -1.58 |
| Atp6v0a2 |  |  |  | -1.58 |
| LOC100044298 |  |  |  | -1.58 |
| Phc2 |  |  |  | -1.58 |
| Slc24a6 |  |  |  | -1.58 |
| Cant1 |  |  |  | -1.59 |
| Golga2 |  |  |  | -1.59 |
| Peci |  |  |  | -1.59 |
| Hgs |  |  |  | -1.6 |
| LOC100048445 |  |  |  | -1.6 |
| Wsb2 |  |  |  | -1.6 |
| Cnp |  |  |  | -1.61 |
| Degs1 |  |  |  | -1.61 |
| Gadd45g |  |  |  | -1.61 |
| Gpc1 |  |  |  | -1.61 |
| Lyrm2 |  |  |  | -1.61 |
| Ep400 |  |  |  | -1.62 |
| Helb |  |  |  | -1.62 |
| Ift172 |  |  |  | -1.62 |
| Snrp70 |  |  |  | -1.62 |
| Agrn |  |  |  | -1.63 |
| Daxx |  |  |  | -1.63 |
| Tmem23 |  |  |  | -1.63 |
| Nfrkb |  |  |  | -1.64 |
| Txlna |  |  |  | -1.64 |
| Cdipt |  |  |  | -1.66 |
| Eps15l1 |  |  |  | -1.66 |
| Pacs1 |  |  |  | -1.66 |
| Rnf145 |  |  |  | -1.66 |
| Ccnd2 |  |  |  | -1.67 |
| Cdkn2aipnl |  |  |  | -1.67 |
| D4Wsu132e |  |  |  | -1.67 |
| LOC100045882 |  |  |  | -1.67 |
| 6430527G18Rik |  |  |  | -1.68 |
| LOC100045005 |  |  |  | -1.68 |
| Psap |  |  |  | -1.68 |
| Tomm22 |  |  |  | -1.68 |
| Atp6ap1 |  |  |  | -1.69 |
| Pitpnm2 |  |  |  | -1.7 |
| Dhx38 |  |  |  | -1.71 |
| LOC100045780 |  |  |  | -1.71 |
| Ptpre |  |  |  | -1.71 |
| Preb |  |  |  | -1.72 |
| Med15 |  |  |  | -1.73 |
| Ppp4c |  |  |  | -1.73 |
| Ars2 |  |  |  | -1.74 |
| Abcc5 |  |  |  | -1.75 |
| Notch1 |  |  |  | -1.75 |
| Edem2 |  |  |  | -1.76 |
| Mycbp2 |  |  |  | -1.76 |
| Specc1l |  |  |  | -1.76 |
| 2700087H15Rik |  |  |  | -1.77 |
| Hsdl1 |  |  |  | -1.77 |
| Pqlc1 |  |  |  | -1.77 |
| Vps16 |  |  |  | -1.77 |
| 2410001C21Rik |  |  |  | -1.78 |
| Cdc2l1 |  |  |  | -1.78 |
| Junb |  |  |  | -1.78 |
| Slc25a45 |  |  |  | -1.78 |
| Erp29 |  |  |  | -1.79 |
| Keap1 |  |  |  | -1.79 |
| Slc25a28 |  |  |  | -1.79 |
| Sppl3 |  |  |  | -1.79 |
| Zkscan6 |  |  |  | -1.79 |
| 3110056O03Rik |  |  |  | -1.8 |
| Oma1 |  |  |  | -1.8 |
| Btbd12 |  |  |  | -1.81 |
| Clk1 |  |  |  | -1.81 |
| Irf8 |  |  |  | -1.81 |
| Slc25a38 |  |  |  | -1.81 |
| Gripap1 |  |  |  | -1.82 |
| Relb |  |  |  | -1.82 |
| Arhgap9 |  |  |  | -1.83 |
| Usp52 |  |  |  | -1.83 |
| Bat1a |  |  |  | -1.84 |
| Gba |  |  |  | -1.84 |
| C230093N12Rik |  |  |  | -1.85 |
| Ppp1r9b |  |  |  | -1.85 |
| Clk3 |  |  |  | -1.86 |
| Sfrs14 |  |  |  | -1.86 |
| 6330569M22Rik |  |  |  | -1.87 |
| Aasdh |  |  |  | -1.87 |
| Map4k2 |  |  |  | -1.87 |
| Camta2 |  |  |  | -1.88 |
| Ddx24 |  |  |  | -1.89 |
| Trabd |  |  |  | -1.89 |
| Faah |  |  |  | -1.9 |
| Med12 |  |  |  | -1.92 |
| Mapk1ip1 |  |  |  | -1.93 |
| Psmb9 |  |  |  | -1.94 |
| Psmd13 |  |  |  | -1.95 |
| 1110038F14Rik |  |  |  | -1.96 |
| Dhrsx |  |  |  | -1.96 |
| Ankrd54 |  |  |  | -1.97 |
| Brd9 |  |  |  | -1.97 |
| Ttc14 |  |  |  | -1.97 |
| Slc35c2 |  |  |  | -1.99 |
| Ascc2 |  |  |  | -2 |
| Ehd1 |  |  |  | -2 |
| Lrrfip1 |  |  |  | -2 |
| Ubtf |  |  |  | -2.01 |
| Chkb |  |  |  | -2.02 |
| Map3k7ip2 |  |  |  | -2.02 |
| Syvn1 |  |  |  | -2.02 |
| 9-Sep |  |  |  | -2.05 |
| Atp9b |  |  |  | -2.05 |
| Brf1 |  |  |  | -2.05 |
| EG665378 |  |  |  | -2.05 |
| Sgpp1 |  |  |  | -2.05 |
| Trpv2 |  |  |  | -2.08 |
| Riok1 |  |  |  | -2.09 |
| Saps3 |  |  |  | -2.09 |
| Cd86 |  |  |  | -2.1 |
| 2310061F22Rik |  |  |  | -2.11 |
| Suv420h2 |  |  |  | -2.13 |
| Nfyb |  |  |  | -2.14 |
| Nrbp2 |  |  |  | -2.14 |
| Trim39 |  |  |  | -2.14 |
| Hsd3b2 |  |  |  | -2.15 |
| Ifngr2 |  |  |  | -2.16 |
| 2310021P13Rik |  |  |  | -2.17 |
| Bach2 |  |  |  | -2.2 |
| Tap1 |  |  |  | -2.2 |
| Zfp523 |  |  |  | -2.2 |
| Slc2a6 |  |  |  | -2.22 |
| Gpatch2 |  |  |  | -2.23 |
| Neu1 |  |  |  | -2.23 |
| Ube1l |  |  |  | -2.23 |
| Adcy6 |  |  |  | -2.26 |
| LOC622404 |  |  |  | -2.27 |
| Slc25a37 |  |  |  | -2.28 |
| Pik3cd |  |  |  | -2.31 |
| Mta3 |  |  |  | -2.33 |
| Tmc6 |  |  |  | -2.34 |
| Myst4 |  |  |  | -2.35 |
| 4930504E06Rik |  |  |  | -2.37 |
| Pold3 |  |  |  | -2.37 |
| Tcof1 |  |  |  | -2.37 |
| Drctnnb1a |  |  |  | -2.38 |
| Fbxw17 |  |  |  | -2.39 |
| Ager |  |  |  | -2.4 |
| Cc2d1b |  |  |  | -2.4 |
| Ddx26b |  |  |  | -2.45 |
| Kcnk6 |  |  |  | -2.45 |
| Gtf2h4 |  |  |  | -2.47 |
| Icos |  |  |  | -2.47 |
| Rpl29 |  |  |  | -2.47 |
| Taf15 |  |  |  | -2.48 |
| Abca7 |  |  |  | -2.55 |
| Itpr2 |  |  |  | -2.58 |
| Rell1 |  |  |  | -2.58 |
| Dmn |  |  |  | -2.59 |
| Haghl |  |  |  | -2.63 |
| Rnf40 |  |  |  | -2.63 |
| Stk11 |  |  |  | -2.65 |
| Ckb |  |  |  | -2.67 |
| BC030476 |  |  |  | -2.69 |
| Tcirg1 |  |  |  | -2.72 |
| Ptpn1 |  |  |  | -2.78 |
| Ptprs |  |  |  | -2.78 |
| Nup210 |  |  |  | -2.89 |
| Psmb1 |  |  |  | -3.06 |
| Amica1 |  |  |  | -3.15 |
| D17H6S56E-5 |  |  |  | -3.22 |
