# Supplementary material for: Genomic deletion of GIT2 induces a premature age-related thymic dysfunction and systemic immune system disruption
Source: Aging (Albany NY). 2017 Mar 4;9(3):706–30. doi: 10.18632/aging.101185 (PMC5391227; doi:10.18632/aging.101185)
Supplement: Supplementary file 15 [file aging-09-706-s015.docx]

**Table S14. Collective Processing *Textrous!* analysis of coherently-regulated transcripts common across GIT2KO ILN, MLN, spleen and thymus tissues.** For each semantically-associated word generated by *Textrous!*, the Cosine Similarity score, association probability and association Z score are represented.

| **Word** | **Cosine Similarity** | **p-Value** | **Z Score** |
| --- | --- | --- | --- |
| oscillators | 0.911218295 | 1.22E-05 | 4.220835 |
| clocks | 0.909209776 | 1.27E-05 | 4.211524 |
| circadian | 0.906570405 | 1.34E-05 | 4.199289 |
| rhythms | 0.903709154 | 1.42E-05 | 4.186026 |
| rhythmicity | 0.901788654 | 1.48E-05 | 4.177123 |
| rhythm | 0.894813908 | 1.70E-05 | 4.144791 |
| suprachiasmatic | 0.891559616 | 1.81E-05 | 4.129706 |
| oscillator | 0.880979233 | 2.24E-05 | 4.08066 |
| light-dark | 0.878009289 | 2.38E-05 | 4.066892 |
| rhythmic | 0.87584671 | 2.49E-05 | 4.056867 |
| darkness | 0.87031893 | 2.78E-05 | 4.031243 |
| cryptochrome | 0.859020135 | 3.46E-05 | 3.978867 |
| evening | 0.855741208 | 3.69E-05 | 3.963667 |
| oscillations | 0.851434953 | 4.01E-05 | 3.943705 |
| pacemaker | 0.847738002 | 4.30E-05 | 3.926568 |
| diurnal | 0.847346683 | 4.34E-05 | 3.924754 |
| period | 0.840942474 | 4.91E-05 | 3.895066 |
| oscillation | 0.826785757 | 6.43E-05 | 3.829442 |
| night | 0.79293428 | 0.00011986 | 3.672521 |
| neurospora | 0.788772897 | 0.000129597 | 3.653231 |
| cryptochromes | 0.781375073 | 0.000147872 | 3.618938 |
| bioluminescence | 0.780407803 | 0.000150755 | 3.614454 |
| cycles | 0.759682926 | 0.000217406 | 3.518382 |
| morning | 0.759584011 | 0.000217406 | 3.517924 |
| pacemakers | 0.752394544 | 0.000246069 | 3.484597 |
| advance | 0.740025709 | 0.000305145 | 3.42726 |
| luminescence | 0.716398465 | 0.000453322 | 3.317734 |
| photic | 0.711493656 | 0.000492108 | 3.294998 |
| synechococcus | 0.706447827 | 0.000533948 | 3.271607 |
| photoperiods | 0.70164004 | 0.000579057 | 3.249321 |
| sleep-wake | 0.676102961 | 0.000871061 | 3.130942 |
| feedback | 0.674546638 | 0.000892052 | 3.123727 |
| pulses | 0.67371568 | 0.000904255 | 3.119875 |
| cycling | 0.651214364 | 0.001280666 | 3.015569 |
| e-box | 0.650446791 | 0.001297663 | 3.012011 |
| photoperiod | 0.634242539 | 0.001657021 | 2.936895 |
| timing | 0.62984392 | 0.001767079 | 2.916505 |
| cyclicity | 0.629436809 | 0.001778443 | 2.914618 |
| loops | 0.627997047 | 0.001818742 | 2.907944 |
| periodicity | 0.622804948 | 0.001963294 | 2.883875 |
| daytime | 0.617544776 | 0.002124894 | 2.859491 |
| synchrony | 0.601164938 | 0.002684652 | 2.783562 |
| shifts | 0.584032063 | 0.003425514 | 2.704141 |
| schedules | 0.582730431 | 0.003487872 | 2.698107 |
| daily | 0.572374421 | 0.004024589 | 2.650101 |
| venus | 0.562823904 | 0.004580323 | 2.605829 |
| constant | 0.555265807 | 0.005070266 | 2.570793 |
| photolyase | 0.554464814 | 0.005129131 | 2.56708 |
| cyanobacteria | 0.54625535 | 0.005719401 | 2.529024 |
| timed | 0.545603333 | 0.005768474 | 2.526002 |
| crassa | 0.53468901 | 0.006661809 | 2.475408 |
| lights | 0.526357517 | 0.00740484 | 2.436787 |
| nighttime | 0.518962539 | 0.008130594 | 2.402507 |
| sleep | 0.515566699 | 0.008493246 | 2.386765 |
| anticipation | 0.514574856 | 0.008609449 | 2.382167 |
| anticipatory | 0.508458155 | 0.009286306 | 2.353813 |
| schedule | 0.503800684 | 0.009850346 | 2.332223 |
| mathematical | 0.496546684 | 0.01075247 | 2.298597 |
| physiology | 0.495172677 | 0.010952822 | 2.292227 |
| synchronization | 0.486212824 | 0.012192769 | 2.250693 |
| metabolome | 0.485115948 | 0.012352004 | 2.245609 |
| wake | 0.469992492 | 0.01477763 | 2.175503 |
| lag | 0.465173031 | 0.015659339 | 2.153162 |
| pcc | 0.459090705 | 0.016793306 | 2.124967 |
| melatonin | 0.458341466 | 0.016960901 | 2.121494 |
| parameter | 0.448962678 | 0.01885468 | 2.078018 |
| nocturnal | 0.448089955 | 0.019039655 | 2.073972 |
| pulse | 0.442861255 | 0.020182215 | 2.049734 |
| shifting | 0.436724018 | 0.021639883 | 2.021285 |
| rebound | 0.43286683 | 0.022588644 | 2.003404 |
| subjective | 0.424002853 | 0.024881242 | 1.962315 |
| casein | 0.421401927 | 0.02558806 | 1.950258 |
| pas | 0.412737799 | 0.028066607 | 1.910095 |
| llc | 0.409908115 | 0.028913969 | 1.896978 |
| helix-loop-helix | 0.407315924 | 0.029714919 | 1.884961 |
| behavioral | 0.398951685 | 0.032446103 | 1.846188 |
| nidulans | 0.396441849 | 0.033252845 | 1.834554 |
| light | 0.390429832 | 0.03538114 | 1.806685 |
| drive | 0.382957325 | 0.038197279 | 1.772046 |
| loop | 0.379227671 | 0.039629654 | 1.754756 |
| photochemical | 0.377429897 | 0.040405476 | 1.746423 |
| dd | 0.376730254 | 0.040666807 | 1.74318 |
| dark | 0.376240355 | 0.040841789 | 1.740909 |
| blue | 0.368916766 | 0.043911026 | 1.70696 |
| vasoconstrictors | 0.359786616 | 0.047956378 | 1.664636 |
| lighting | 0.358855899 | 0.048457226 | 1.660322 |
| lengthening | 0.358540123 | 0.048557896 | 1.658858 |
| feeding | 0.356384519 | 0.049573817 | 1.648866 |
