# Supplementary material for: Genomic deletion of GIT2 induces a premature age-related thymic dysfunction and systemic immune system disruption
Source: Aging (Albany NY). 2017 Mar 4;9(3):706–30. doi: 10.18632/aging.101185 (PMC5391227; doi:10.18632/aging.101185)
Supplement: Supplementary file 16 [file aging-09-706-s016.docx]

**Table S15. Ingenuity Pathway Analysis-based canonical signaling pathway analysis of significantly regulated transcripts differential between GIT2KO ILN compared to WT controls.** For each canonical signaling pathway the –log_10_ p value, enrichment ratio (Ratio), downregulated pathway-populating transcript numbers (Downregulated) and upregulated pathway-populating transcript numbers (Upregulated) are represented.

| **Canonical Signaling Pathway** | **-log(p-value)** | **Ratio** | **Downregulated** | **No change** | **Upregulated** | **No overlap with dataset** |
| --- | --- | --- | --- | --- | --- | --- |
| Role of NFAT in Regulation of the Immune Response | 9.98E+00 | 1.17E-01 | 9/171 (5%) | 0/171 (0%) | 11/171 (6%) | 151/171 (88%) |
| Systemic Lupus Erythematosus Signaling | 8.25E+00 | 9.35E-02 | 7/214 (3%) | 0/214 (0%) | 13/214 (6%) | 194/214 (91%) |
| CD28 Signaling in T Helper Cells | 7.24E+00 | 1.19E-01 | 8/118 (7%) | 0/118 (0%) | 6/118 (5%) | 104/118 (88%) |
| OX40 Signaling Pathway | 6.91E+00 | 1.35E-01 | 5/89 (6%) | 0/89 (0%) | 7/89 (8%) | 77/89 (87%) |
| iCOS-iCOSL Signaling in T Helper Cells | 6.84E+00 | 1.20E-01 | 8/108 (7%) | 0/108 (0%) | 5/108 (5%) | 95/108 (88%) |
| PKCθ Signaling in T Lymphocytes | 6.39E+00 | 1.10E-01 | 7/118 (6%) | 0/118 (0%) | 6/118 (5%) | 105/118 (89%) |
| Phospholipase C Signaling | 6.13E+00 | 7.59E-02 | 11/237 (5%) | 0/237 (0%) | 7/237 (3%) | 219/237 (92%) |
| Cdc42 Signaling | 6.12E+00 | 8.98E-02 | 5/167 (3%) | 0/167 (0%) | 10/167 (6%) | 152/167 (91%) |
| CTLA4 Signaling in Cytotoxic T Lymphocytes | 6.05E+00 | 1.25E-01 | 9/88 (10%) | 0/88 (0%) | 2/88 (2%) | 77/88 (88%) |
| T Cell Receptor Signaling | 5.62E+00 | 1.13E-01 | 9/97 (9%) | 0/97 (0%) | 2/97 (2%) | 86/97 (89%) |
| T Helper Cell Differentiation | 5.10E+00 | 1.27E-01 | 2/71 (3%) | 0/71 (0%) | 7/71 (10%) | 62/71 (87%) |
| Type I Diabetes Mellitus Signaling | 5.08E+00 | 1.00E-01 | 4/110 (4%) | 0/110 (0%) | 7/110 (6%) | 99/110 (90%) |
| PDGF Signaling | 4.81E+00 | 1.17E-01 | 1/77 (1%) | 0/77 (0%) | 8/77 (10%) | 68/77 (88%) |
| NF-κB Signaling | 4.57E+00 | 7.56E-02 | 5/172 (3%) | 0/172 (0%) | 8/172 (5%) | 159/172 (92%) |
| Calcium-induced T Lymphocyte Apoptosis | 4.55E+00 | 1.25E-01 | 5/64 (8%) | 0/64 (0%) | 3/64 (5%) | 56/64 (88%) |
| Leukocyte Extravasation Signaling | 4.55E+00 | 7.07E-02 | 9/198 (5%) | 0/198 (0%) | 5/198 (3%) | 184/198 (93%) |
| Allograft Rejection Signaling | 4.50E+00 | 1.07E-01 | 1/84 (1%) | 0/84 (0%) | 8/84 (10%) | 75/84 (89%) |
| Primary Immunodeficiency Signaling | 4.49E+00 | 1.46E-01 | 5/48 (10%) | 0/48 (0%) | 2/48 (4%) | 41/48 (85%) |
| B Cell Development | 4.48E+00 | 1.82E-01 | 1/33 (3%) | 0/33 (0%) | 5/33 (15%) | 27/33 (82%) |
| Role of NFAT in Cardiac Hypertrophy | 4.39E+00 | 7.26E-02 | 5/179 (3%) | 0/179 (0%) | 8/179 (4%) | 166/179 (93%) |
| Natural Killer Cell Signaling | 4.31E+00 | 9.09E-02 | 5/110 (5%) | 0/110 (0%) | 5/110 (5%) | 100/110 (91%) |
| Virus Entry via Endocytic Pathways | 4.30E+00 | 1.01E-01 | 2/89 (2%) | 0/89 (0%) | 7/89 (8%) | 80/89 (90%) |
| Hepatic Fibrosis / Hepatic Stellate Cell Activation | 4.29E+00 | 7.10E-02 | 5/183 (3%) | 0/183 (0%) | 8/183 (4%) | 170/183 (93%) |
| Agranulocyte Adhesion and Diapedesis | 4.14E+00 | 6.88E-02 | 7/189 (4%) | 0/189 (0%) | 6/189 (3%) | 176/189 (93%) |
| Glucocorticoid Receptor Signaling | 4.08E+00 | 5.82E-02 | 7/275 (3%) | 0/275 (0%) | 9/275 (3%) | 259/275 (94%) |
| Glioma Invasiveness Signaling | 4.00E+00 | 1.23E-01 | 4/57 (7%) | 0/57 (0%) | 3/57 (5%) | 50/57 (88%) |
| Nur77 Signaling in T Lymphocytes | 4.00E+00 | 1.23E-01 | 4/57 (7%) | 0/57 (0%) | 3/57 (5%) | 50/57 (88%) |
| Glioma Signaling | 3.97E+00 | 9.18E-02 | 4/98 (4%) | 0/98 (0%) | 5/98 (5%) | 89/98 (91%) |
| Regulation of IL-2 Expression in Activated and Anergic T Lymphocytes | 3.89E+00 | 1.01E-01 | 5/79 (6%) | 0/79 (0%) | 3/79 (4%) | 71/79 (90%) |
| Role of JAK1 and JAK3 in γc Cytokine Signaling | 3.76E+00 | 1.13E-01 | 2/62 (3%) | 0/62 (0%) | 5/62 (8%) | 55/62 (89%) |
| Hereditary Breast Cancer Signaling | 3.73E+00 | 7.75E-02 | 3/129 (2%) | 0/129 (0%) | 7/129 (5%) | 119/129 (92%) |
| Pancreatic Adenocarcinoma Signaling | 3.71E+00 | 8.49E-02 | 2/106 (2%) | 0/106 (0%) | 7/106 (7%) | 97/106 (92%) |
| Autoimmune Thyroid Disease Signaling | 3.59E+00 | 1.28E-01 | 1/47 (2%) | 0/47 (0%) | 5/47 (11%) | 41/47 (87%) |
| Axonal Guidance Signaling | 3.56E+00 | 4.61E-02 | 5/434 (1%) | 0/434 (0%) | 15/434 (3%) | 414/434 (95%) |
| Altered T Cell and B Cell Signaling in Rheumatoid Arthritis | 3.56E+00 | 9.09E-02 | 0/88 (0%) | 0/88 (0%) | 8/88 (9%) | 80/88 (91%) |
| Graft-versus-Host Disease Signaling | 3.54E+00 | 1.25E-01 | 1/48 (2%) | 0/48 (0%) | 5/48 (10%) | 42/48 (88%) |
| G Protein Signaling Mediated by Tubby | 3.49E+00 | 1.56E-01 | 1/32 (3%) | 0/32 (0%) | 4/32 (13%) | 27/32 (84%) |
| CCR5 Signaling in Macrophages | 3.47E+00 | 1.01E-01 | 3/69 (4%) | 0/69 (0%) | 4/69 (6%) | 62/69 (90%) |
| eNOS Signaling | 3.40E+00 | 7.04E-02 | 2/142 (1%) | 0/142 (0%) | 8/142 (6%) | 132/142 (93%) |
| Chronic Myeloid Leukemia Signaling | 3.40E+00 | 8.60E-02 | 3/93 (3%) | 0/93 (0%) | 5/93 (5%) | 85/93 (91%) |
| IL-9 Signaling | 3.36E+00 | 1.47E-01 | 2/34 (6%) | 0/34 (0%) | 3/34 (9%) | 29/34 (85%) |
| Ephrin B Signaling | 3.32E+00 | 9.59E-02 | 2/73 (3%) | 0/73 (0%) | 5/73 (7%) | 66/73 (90%) |
| RhoGDI Signaling | 3.30E+00 | 6.36E-02 | 3/173 (2%) | 0/173 (0%) | 8/173 (5%) | 162/173 (94%) |
| Ephrin Receptor Signaling | 3.28E+00 | 6.32E-02 | 2/174 (1%) | 0/174 (0%) | 9/174 (5%) | 163/174 (94%) |
| IGF-1 Signaling | 3.27E+00 | 8.25E-02 | 3/97 (3%) | 0/97 (0%) | 5/97 (5%) | 89/97 (92%) |
| IL-4 Signaling | 3.25E+00 | 9.33E-02 | 1/75 (1%) | 0/75 (0%) | 6/75 (8%) | 68/75 (91%) |
| Signaling by Rho Family GTPases | 3.23E+00 | 5.56E-02 | 3/234 (1%) | 0/234 (0%) | 10/234 (4%) | 221/234 (94%) |
| HER-2 Signaling in Breast Cancer | 3.21E+00 | 9.21E-02 | 4/76 (5%) | 0/76 (0%) | 3/76 (4%) | 69/76 (91%) |
| Colorectal Cancer Metastasis Signaling | 3.20E+00 | 5.51E-02 | 4/236 (2%) | 0/236 (0%) | 9/236 (4%) | 223/236 (94%) |
| PPARα/RXRα Activation | 3.20E+00 | 6.18E-02 | 2/178 (1%) | 0/178 (0%) | 9/178 (5%) | 167/178 (94%) |
| Integrin Signaling | 3.19E+00 | 5.80E-02 | 3/207 (1%) | 0/207 (0%) | 9/207 (4%) | 195/207 (94%) |
| Aldosterone Signaling in Epithelial Cells | 3.16E+00 | 6.58E-02 | 4/152 (3%) | 0/152 (0%) | 6/152 (4%) | 142/152 (93%) |
| NRF2-mediated Oxidative Stress Response | 3.16E+00 | 6.11E-02 | 7/180 (4%) | 0/180 (0%) | 4/180 (2%) | 169/180 (94%) |
| Clathrin-mediated Endocytosis Signaling | 3.06E+00 | 5.95E-02 | 3/185 (2%) | 0/185 (0%) | 8/185 (4%) | 174/185 (94%) |
| Tec Kinase Signaling | 3.06E+00 | 6.37E-02 | 3/157 (2%) | 0/157 (0%) | 7/157 (4%) | 147/157 (94%) |
| Ovarian Cancer Signaling | 3.03E+00 | 6.87E-02 | 3/131 (2%) | 0/131 (0%) | 6/131 (5%) | 122/131 (93%) |
| Prostate Cancer Signaling | 3.02E+00 | 8.54E-02 | 2/82 (2%) | 0/82 (0%) | 5/82 (6%) | 75/82 (91%) |
| ErbB4 Signaling | 3.01E+00 | 1.00E-01 | 2/60 (3%) | 0/60 (0%) | 4/60 (7%) | 54/60 (90%) |
| FcγRIIB Signaling in B Lymphocytes | 2.98E+00 | 1.22E-01 | 1/41 (2%) | 0/41 (0%) | 4/41 (10%) | 36/41 (88%) |
| fMLP Signaling in Neutrophils | 2.96E+00 | 7.41E-02 | 1/108 (1%) | 0/108 (0%) | 7/108 (6%) | 100/108 (93%) |
| Breast Cancer Regulation by Stathmin1 | 2.95E+00 | 5.76E-02 | 4/191 (2%) | 0/191 (0%) | 7/191 (4%) | 180/191 (94%) |
| GM-CSF Signaling | 2.94E+00 | 9.68E-02 | 2/62 (3%) | 0/62 (0%) | 4/62 (6%) | 56/62 (90%) |
| Melanoma Signaling | 2.93E+00 | 1.19E-01 | 1/42 (2%) | 0/42 (0%) | 4/42 (10%) | 37/42 (88%) |
| Superpathway of Inositol Phosphate Compounds | 2.91E+00 | 5.70E-02 | 4/193 (2%) | 0/193 (0%) | 7/193 (4%) | 182/193 (94%) |
| G Beta Gamma Signaling | 2.84E+00 | 7.95E-02 | 0/88 (0%) | 0/88 (0%) | 7/88 (8%) | 81/88 (92%) |
| Non-Small Cell Lung Cancer Signaling | 2.83E+00 | 9.23E-02 | 2/65 (3%) | 0/65 (0%) | 4/65 (6%) | 59/65 (91%) |
| Aryl Hydrocarbon Receptor Signaling | 2.83E+00 | 6.43E-02 | 5/140 (4%) | 0/140 (0%) | 4/140 (3%) | 131/140 (94%) |
| Acute Phase Response Signaling | 2.81E+00 | 5.92E-02 | 4/169 (2%) | 0/169 (0%) | 6/169 (4%) | 159/169 (94%) |
| Communication between Innate and Adaptive Immune Cells | 2.81E+00 | 7.87E-02 | 3/89 (3%) | 0/89 (0%) | 4/89 (4%) | 82/89 (92%) |
| PTEN Signaling | 2.72E+00 | 6.78E-02 | 3/118 (3%) | 0/118 (0%) | 5/118 (4%) | 110/118 (93%) |
| Growth Hormone Signaling | 2.70E+00 | 8.70E-02 | 2/69 (3%) | 0/69 (0%) | 4/69 (6%) | 63/69 (91%) |
| P2Y Purigenic Receptor Signaling Pathway | 2.69E+00 | 6.72E-02 | 2/119 (2%) | 0/119 (0%) | 6/119 (5%) | 111/119 (93%) |
| Glutathione-mediated Detoxification | 2.67E+00 | 1.38E-01 | 3/29 (10%) | 0/29 (0%) | 1/29 (3%) | 25/29 (86%) |
| Geranylgeranyldiphosphate Biosynthesis | 2.67E+00 | 5.00E-01 | 1/4 (25%) | 0/4 (0%) | 1/4 (25%) | 2/4 (50%) |
| Dendritic Cell Maturation | 2.66E+00 | 5.65E-02 | 1/177 (1%) | 0/177 (0%) | 9/177 (5%) | 167/177 (94%) |
| Granulocyte Adhesion and Diapedesis | 2.66E+00 | 5.65E-02 | 6/177 (3%) | 0/177 (0%) | 4/177 (2%) | 167/177 (94%) |
| Renal Cell Carcinoma Signaling | 2.63E+00 | 8.45E-02 | 1/71 (1%) | 0/71 (0%) | 5/71 (7%) | 65/71 (92%) |
| PI3K/AKT Signaling | 2.60E+00 | 6.50E-02 | 2/123 (2%) | 0/123 (0%) | 6/123 (5%) | 115/123 (93%) |
| JAK/Stat Signaling | 2.60E+00 | 8.33E-02 | 2/72 (3%) | 0/72 (0%) | 4/72 (6%) | 66/72 (92%) |
| CXCR4 Signaling | 2.59E+00 | 5.92E-02 | 4/152 (3%) | 0/152 (0%) | 5/152 (3%) | 143/152 (94%) |
| NF-κB Activation by Viruses | 2.57E+00 | 8.22E-02 | 2/73 (3%) | 0/73 (0%) | 4/73 (5%) | 67/73 (92%) |
| Prolactin Signaling | 2.57E+00 | 8.22E-02 | 1/73 (1%) | 0/73 (0%) | 5/73 (7%) | 67/73 (92%) |
| STAT3 Pathway | 2.57E+00 | 8.22E-02 | 3/73 (4%) | 0/73 (0%) | 3/73 (4%) | 67/73 (92%) |
| Leptin Signaling in Obesity | 2.54E+00 | 8.11E-02 | 2/74 (3%) | 0/74 (0%) | 4/74 (5%) | 68/74 (92%) |
| Gap Junction Signaling | 2.53E+00 | 5.81E-02 | 2/155 (1%) | 0/155 (0%) | 7/155 (5%) | 146/155 (94%) |
| Actin Cytoskeleton Signaling | 2.52E+00 | 5.09E-02 | 2/216 (1%) | 0/216 (0%) | 9/216 (4%) | 205/216 (95%) |
| CNTF Signaling | 2.52E+00 | 9.62E-02 | 2/52 (4%) | 0/52 (0%) | 3/52 (6%) | 47/52 (90%) |
| Cytotoxic T Lymphocyte-mediated Apoptosis of Target Cells | 2.51E+00 | 1.25E-01 | 3/32 (9%) | 0/32 (0%) | 1/32 (3%) | 28/32 (88%) |
| 3-phosphoinositide Biosynthesis | 2.51E+00 | 5.77E-02 | 4/156 (3%) | 0/156 (0%) | 5/156 (3%) | 147/156 (94%) |
| VEGF Family Ligand-Receptor Interactions | 2.48E+00 | 7.89E-02 | 2/76 (3%) | 0/76 (0%) | 4/76 (5%) | 70/76 (92%) |
| RAR Activation | 2.44E+00 | 5.26E-02 | 5/190 (3%) | 0/190 (0%) | 5/190 (3%) | 180/190 (95%) |
| Thrombin Signaling | 2.44E+00 | 5.26E-02 | 3/190 (2%) | 0/190 (0%) | 7/190 (4%) | 180/190 (95%) |
| Superpathway of Geranylgeranyldiphosphate Biosynthesis I (via Mevalonate) | 2.42E+00 | 1.76E-01 | 2/17 (12%) | 0/17 (0%) | 1/17 (6%) | 14/17 (82%) |
| Oncostatin M Signaling | 2.42E+00 | 1.18E-01 | 2/34 (6%) | 0/34 (0%) | 2/34 (6%) | 30/34 (88%) |
| Thrombopoietin Signaling | 2.41E+00 | 9.09E-02 | 1/55 (2%) | 0/55 (0%) | 4/55 (7%) | 50/55 (91%) |
| Reelin Signaling in Neurons | 2.40E+00 | 7.59E-02 | 4/79 (5%) | 0/79 (0%) | 2/79 (3%) | 73/79 (92%) |
| Relaxin Signaling | 2.36E+00 | 5.93E-02 | 3/135 (2%) | 0/135 (0%) | 5/135 (4%) | 127/135 (94%) |
| Renin-Angiotensin Signaling | 2.31E+00 | 6.42E-02 | 2/109 (2%) | 0/109 (0%) | 5/109 (5%) | 102/109 (94%) |
| Antigen Presentation Pathway | 2.28E+00 | 1.08E-01 | 1/37 (3%) | 0/37 (0%) | 3/37 (8%) | 33/37 (89%) |
| Glutathione Redox Reactions I | 2.28E+00 | 1.58E-01 | 3/19 (16%) | 0/19 (0%) | 0/19 (0%) | 16/19 (84%) |
| Melanocyte Development and Pigmentation Signaling | 2.27E+00 | 7.14E-02 | 2/84 (2%) | 0/84 (0%) | 4/84 (5%) | 78/84 (93%) |
| Inhibition of Matrix Metalloproteases | 2.20E+00 | 1.03E-01 | 3/39 (8%) | 0/39 (0%) | 1/39 (3%) | 35/39 (90%) |
| α-Adrenergic Signaling | 2.20E+00 | 6.90E-02 | 1/87 (1%) | 0/87 (0%) | 5/87 (6%) | 81/87 (93%) |
| Bladder Cancer Signaling | 2.20E+00 | 6.90E-02 | 2/87 (2%) | 0/87 (0%) | 4/87 (5%) | 81/87 (93%) |
| D-myo-inositol-5-phosphate Metabolism | 2.17E+00 | 5.52E-02 | 3/145 (2%) | 0/145 (0%) | 5/145 (3%) | 137/145 (94%) |
| Neuregulin Signaling | 2.17E+00 | 6.82E-02 | 2/88 (2%) | 0/88 (0%) | 4/88 (5%) | 82/88 (93%) |
| IL-6 Signaling | 2.17E+00 | 6.03E-02 | 2/116 (2%) | 0/116 (0%) | 5/116 (4%) | 109/116 (94%) |
| Antiproliferative Role of Somatostatin Receptor 2 | 2.16E+00 | 7.94E-02 | 1/63 (2%) | 0/63 (0%) | 4/63 (6%) | 58/63 (92%) |
| Estrogen-Dependent Breast Cancer Signaling | 2.16E+00 | 7.94E-02 | 1/63 (2%) | 0/63 (0%) | 4/63 (6%) | 58/63 (92%) |
| Glioblastoma Multiforme Signaling | 2.16E+00 | 5.48E-02 | 3/146 (2%) | 0/146 (0%) | 5/146 (3%) | 138/146 (95%) |
| 3-phosphoinositide Degradation | 2.16E+00 | 5.48E-02 | 3/146 (2%) | 0/146 (0%) | 5/146 (3%) | 138/146 (95%) |
| 14-3-3-mediated Signaling | 2.15E+00 | 5.98E-02 | 2/117 (2%) | 0/117 (0%) | 5/117 (4%) | 110/117 (94%) |
| CCR3 Signaling in Eosinophils | 2.15E+00 | 5.98E-02 | 1/117 (1%) | 0/117 (0%) | 6/117 (5%) | 110/117 (94%) |
| Protein Kinase A Signaling | 2.12E+00 | 3.89E-02 | 6/386 (2%) | 0/386 (0%) | 9/386 (2%) | 371/386 (96%) |
| Gαi Signaling | 2.09E+00 | 5.83E-02 | 1/120 (1%) | 0/120 (0%) | 6/120 (5%) | 113/120 (94%) |
| VEGF Signaling | 2.08E+00 | 6.52E-02 | 1/92 (1%) | 0/92 (0%) | 5/92 (5%) | 86/92 (93%) |
| RhoA Signaling | 2.05E+00 | 5.74E-02 | 1/122 (1%) | 0/122 (0%) | 6/122 (5%) | 115/122 (94%) |
| IL-8 Signaling | 2.05E+00 | 4.89E-02 | 2/184 (1%) | 0/184 (0%) | 7/184 (4%) | 175/184 (95%) |
| Erythropoietin Signaling | 2.05E+00 | 7.46E-02 | 1/67 (1%) | 0/67 (0%) | 4/67 (6%) | 62/67 (93%) |
| TCA Cycle II (Eukaryotic) | 2.04E+00 | 1.30E-01 | 0/23 (0%) | 0/23 (0%) | 3/23 (13%) | 20/23 (87%) |
| Macropinocytosis Signaling | 2.02E+00 | 7.35E-02 | 2/68 (3%) | 0/68 (0%) | 3/68 (4%) | 63/68 (93%) |
| Atherosclerosis Signaling | 2.01E+00 | 5.65E-02 | 3/124 (2%) | 0/124 (0%) | 4/124 (3%) | 117/124 (94%) |
| ERK/MAPK Signaling | 2.01E+00 | 4.81E-02 | 2/187 (1%) | 0/187 (0%) | 7/187 (4%) | 178/187 (95%) |
| Protein Ubiquitination Pathway | 2.00E+00 | 4.31E-02 | 3/255 (1%) | 0/255 (0%) | 8/255 (3%) | 244/255 (96%) |
| Molecular Mechanisms of Cancer | 1.97E+00 | 3.84E-02 | 8/365 (2%) | 0/365 (0%) | 6/365 (2%) | 351/365 (96%) |
| Cardiac Hypertrophy Signaling | 1.97E+00 | 4.48E-02 | 2/223 (1%) | 0/223 (0%) | 8/223 (4%) | 213/223 (96%) |
| Caveolar-mediated Endocytosis Signaling | 1.94E+00 | 7.04E-02 | 1/71 (1%) | 0/71 (0%) | 4/71 (6%) | 66/71 (93%) |
| Small Cell Lung Cancer Signaling | 1.94E+00 | 7.04E-02 | 2/71 (3%) | 0/71 (0%) | 3/71 (4%) | 66/71 (93%) |
| Chemokine Signaling | 1.94E+00 | 7.04E-02 | 2/71 (3%) | 0/71 (0%) | 3/71 (4%) | 66/71 (93%) |
| Role of JAK family kinases in IL-6-type Cytokine Signaling | 1.94E+00 | 1.20E-01 | 1/25 (4%) | 0/25 (0%) | 2/25 (8%) | 22/25 (88%) |
| Telomerase Signaling | 1.93E+00 | 6.06E-02 | 2/99 (2%) | 0/99 (0%) | 4/99 (4%) | 93/99 (94%) |
| Role of Macrophages, Fibroblasts and Endothelial Cells in Rheumatoid Arthritis | 1.93E+00 | 4.05E-02 | 5/296 (2%) | 0/296 (0%) | 7/296 (2%) | 284/296 (96%) |
| D-myo-inositol (1,4,5,6)-Tetrakisphosphate Biosynthesis | 1.92E+00 | 5.43E-02 | 3/129 (2%) | 0/129 (0%) | 4/129 (3%) | 122/129 (95%) |
| D-myo-inositol (3,4,5,6)-tetrakisphosphate Biosynthesis | 1.92E+00 | 5.43E-02 | 3/129 (2%) | 0/129 (0%) | 4/129 (3%) | 122/129 (95%) |
| Hematopoiesis from Pluripotent Stem Cells | 1.92E+00 | 8.51E-02 | 4/47 (9%) | 0/47 (0%) | 0/47 (0%) | 43/47 (91%) |
| Nitric Oxide Signaling in the Cardiovascular System | 1.91E+00 | 6.00E-02 | 2/100 (2%) | 0/100 (0%) | 4/100 (4%) | 94/100 (94%) |
| Insulin Receptor Signaling | 1.87E+00 | 5.30E-02 | 2/132 (2%) | 0/132 (0%) | 5/132 (4%) | 125/132 (95%) |
| Adipogenesis pathway | 1.84E+00 | 5.22E-02 | 6/134 (4%) | 0/134 (0%) | 1/134 (1%) | 127/134 (95%) |
| Rac Signaling | 1.83E+00 | 5.77E-02 | 1/104 (1%) | 0/104 (0%) | 5/104 (5%) | 98/104 (94%) |
| Xenobiotic Metabolism Signaling | 1.80E+00 | 4.04E-02 | 7/272 (3%) | 0/272 (0%) | 4/272 (1%) | 261/272 (96%) |
| Superpathway of Cholesterol Biosynthesis | 1.80E+00 | 1.07E-01 | 1/28 (4%) | 0/28 (0%) | 2/28 (7%) | 25/28 (89%) |
| Wnt/β-catenin Signaling | 1.80E+00 | 4.73E-02 | 4/169 (2%) | 0/169 (0%) | 4/169 (2%) | 161/169 (95%) |
| CREB Signaling in Neurons | 1.77E+00 | 4.68E-02 | 2/171 (1%) | 0/171 (0%) | 6/171 (4%) | 163/171 (95%) |
| Endometrial Cancer Signaling | 1.77E+00 | 7.69E-02 | 1/52 (2%) | 0/52 (0%) | 3/52 (6%) | 48/52 (92%) |
| Fc Epsilon RI Signaling | 1.76E+00 | 5.56E-02 | 2/108 (2%) | 0/108 (0%) | 4/108 (4%) | 102/108 (94%) |
| Sphingosine-1-phosphate Signaling | 1.74E+00 | 5.50E-02 | 2/109 (2%) | 0/109 (0%) | 4/109 (4%) | 103/109 (94%) |
| IL-2 Signaling | 1.74E+00 | 7.55E-02 | 2/53 (4%) | 0/53 (0%) | 2/53 (4%) | 49/53 (92%) |
| B Cell Receptor Signaling | 1.73E+00 | 4.60E-02 | 1/174 (1%) | 0/174 (0%) | 7/174 (4%) | 166/174 (95%) |
| Role of Tissue Factor in Cancer | 1.73E+00 | 5.45E-02 | 3/110 (3%) | 0/110 (0%) | 3/110 (3%) | 104/110 (95%) |
| Role of p14/p19ARF in Tumor Suppression | 1.72E+00 | 1.00E-01 | 1/30 (3%) | 0/30 (0%) | 2/30 (7%) | 27/30 (90%) |
| Corticotropin Releasing Hormone Signaling | 1.71E+00 | 5.41E-02 | 1/111 (1%) | 0/111 (0%) | 5/111 (5%) | 105/111 (95%) |
| Androgen Signaling | 1.71E+00 | 5.41E-02 | 1/111 (1%) | 0/111 (0%) | 5/111 (5%) | 105/111 (95%) |
| AMPK Signaling | 1.68E+00 | 4.49E-02 | 2/178 (1%) | 0/178 (0%) | 6/178 (3%) | 170/178 (96%) |
| Epithelial Adherens Junction Signaling | 1.66E+00 | 4.79E-02 | 1/146 (1%) | 0/146 (0%) | 6/146 (4%) | 139/146 (95%) |
| GPCR-Mediated Nutrient Sensing in Enteroendocrine Cells | 1.65E+00 | 5.95E-02 | 1/84 (1%) | 0/84 (0%) | 4/84 (5%) | 79/84 (94%) |
| TR/RXR Activation | 1.63E+00 | 5.88E-02 | 2/85 (2%) | 0/85 (0%) | 3/85 (4%) | 80/85 (94%) |
| ErbB2-ErbB3 Signaling | 1.63E+00 | 7.02E-02 | 1/57 (2%) | 0/57 (0%) | 3/57 (5%) | 53/57 (93%) |
| Myc Mediated Apoptosis Signaling | 1.61E+00 | 6.90E-02 | 1/58 (2%) | 0/58 (0%) | 3/58 (5%) | 54/58 (93%) |
| Oleate Biosynthesis II (Animals) | 1.61E+00 | 1.54E-01 | 1/13 (8%) | 0/13 (0%) | 1/13 (8%) | 11/13 (85%) |
| p38 MAPK Signaling | 1.61E+00 | 5.13E-02 | 1/117 (1%) | 0/117 (0%) | 5/117 (4%) | 111/117 (95%) |
| FAK Signaling | 1.60E+00 | 5.75E-02 | 1/87 (1%) | 0/87 (0%) | 4/87 (5%) | 82/87 (94%) |
| LPS/IL-1 Mediated Inhibition of RXR Function | 1.59E+00 | 4.09E-02 | 7/220 (3%) | 0/220 (0%) | 2/220 (1%) | 211/220 (96%) |
| UVA-Induced MAPK Signaling | 1.58E+00 | 5.68E-02 | 1/88 (1%) | 0/88 (0%) | 4/88 (5%) | 83/88 (94%) |
| p70S6K Signaling | 1.58E+00 | 5.04E-02 | 1/119 (1%) | 0/119 (0%) | 5/119 (4%) | 113/119 (95%) |
| mTOR Signaling | 1.56E+00 | 4.28E-02 | 4/187 (2%) | 0/187 (0%) | 4/187 (2%) | 179/187 (96%) |
| Cell Cycle Regulation by BTG Family Proteins | 1.54E+00 | 8.57E-02 | 0/35 (0%) | 0/35 (0%) | 3/35 (9%) | 32/35 (91%) |
| Regulation of Actin-based Motility by Rho | 1.52E+00 | 5.49E-02 | 0/91 (0%) | 0/91 (0%) | 5/91 (5%) | 86/91 (95%) |
| IL-15 Signaling | 1.49E+00 | 6.35E-02 | 2/63 (3%) | 0/63 (0%) | 2/63 (3%) | 59/63 (94%) |
| ERK5 Signaling | 1.49E+00 | 6.35E-02 | 1/63 (2%) | 0/63 (0%) | 3/63 (5%) | 59/63 (94%) |
| Nicotine Degradation II | 1.49E+00 | 6.35E-02 | 4/63 (6%) | 0/63 (0%) | 0/63 (0%) | 59/63 (94%) |
| Fcγ Receptor-mediated Phagocytosis in Macrophages and Monocytes | 1.49E+00 | 5.38E-02 | 1/93 (1%) | 0/93 (0%) | 4/93 (4%) | 88/93 (95%) |
| Cell Cycle: G1/S Checkpoint Regulation | 1.47E+00 | 6.25E-02 | 2/64 (3%) | 0/64 (0%) | 2/64 (3%) | 60/64 (94%) |
| SAPK/JNK Signaling | 1.47E+00 | 5.32E-02 | 2/94 (2%) | 0/94 (0%) | 3/94 (3%) | 89/94 (95%) |
| PI3K Signaling in B Lymphocytes | 1.46E+00 | 4.72E-02 | 1/127 (1%) | 0/127 (0%) | 5/127 (4%) | 121/127 (95%) |
| Mouse Embryonic Stem Cell Pluripotency | 1.45E+00 | 5.26E-02 | 3/95 (3%) | 0/95 (0%) | 2/95 (2%) | 90/95 (95%) |
| CD40 Signaling | 1.45E+00 | 6.15E-02 | 1/65 (2%) | 0/65 (0%) | 3/65 (5%) | 61/65 (94%) |
| Estrogen Biosynthesis | 1.45E+00 | 7.89E-02 | 2/38 (5%) | 0/38 (0%) | 1/38 (3%) | 35/38 (92%) |
| Cellular Effects of Sildenafil (Viagra) | 1.43E+00 | 4.65E-02 | 2/129 (2%) | 0/129 (0%) | 4/129 (3%) | 123/129 (95%) |
| p53 Signaling | 1.40E+00 | 5.10E-02 | 2/98 (2%) | 0/98 (0%) | 3/98 (3%) | 93/98 (95%) |
| GDNF Family Ligand-Receptor Interactions | 1.39E+00 | 5.88E-02 | 1/68 (1%) | 0/68 (0%) | 3/68 (4%) | 64/68 (94%) |
| Remodeling of Epithelial Adherens Junctions | 1.39E+00 | 5.88E-02 | 0/68 (0%) | 0/68 (0%) | 4/68 (6%) | 64/68 (94%) |
| Cardiac β-adrenergic Signaling | 1.38E+00 | 4.51E-02 | 2/133 (2%) | 0/133 (0%) | 4/133 (3%) | 127/133 (95%) |
| Human Embryonic Stem Cell Pluripotency | 1.36E+00 | 4.48E-02 | 2/134 (1%) | 0/134 (0%) | 4/134 (3%) | 128/134 (96%) |
| Paxillin Signaling | 1.36E+00 | 4.95E-02 | 2/101 (2%) | 0/101 (0%) | 3/101 (3%) | 96/101 (95%) |
| HIF1α Signaling | 1.34E+00 | 4.90E-02 | 2/102 (2%) | 0/102 (0%) | 3/102 (3%) | 97/102 (95%) |
| Mitochondrial Dysfunction | 1.34E+00 | 4.09E-02 | 1/171 (1%) | 0/171 (0%) | 6/171 (4%) | 164/171 (96%) |
| IL-3 Signaling | 1.33E+00 | 5.63E-02 | 1/71 (1%) | 0/71 (0%) | 3/71 (4%) | 67/71 (94%) |
| HGF Signaling | 1.30E+00 | 4.76E-02 | 1/105 (1%) | 0/105 (0%) | 4/105 (4%) | 100/105 (95%) |
