# Supplementary material for: Genomic deletion of GIT2 induces a premature age-related thymic dysfunction and systemic immune system disruption
Source: Aging (Albany NY). 2017 Mar 4;9(3):706–30. doi: 10.18632/aging.101185 (PMC5391227; doi:10.18632/aging.101185)
Supplement: Supplementary file 17 [file aging-09-706-s017.docx]

**Table S16. IPA-based canonical signaling pathway analysis of significantly regulated transcripts differential between GIT2KO MLN compared to WT controls.** For each canonical signaling pathway the –log_10_ p value, enrichment ratio (Ratio), downregulated pathway-populating transcript numbers (Downregulated) and upregulated pathway-populating transcript numbers (Upregulated) are represented.

| **Canonical Signaling Pathway** | **-log(p-value)** | **Ratio** | **Downregulated** | **No change** | **Upregulated** | **No overlap with dataset** |
| --- | --- | --- | --- | --- | --- | --- |
| Prostate Cancer Signaling | 5.06E+00 | 9.76E-02 | 3/82 (4%) | 0/82 (0%) | 5/82 (6%) | 74/82 (90%) |
| Aryl Hydrocarbon Receptor Signaling | 4.96E+00 | 7.14E-02 | 5/140 (4%) | 0/140 (0%) | 5/140 (4%) | 130/140 (93%) |
| Regulation of IL-2 Expression in Activated and Anergic T Lymphocytes | 4.23E+00 | 8.86E-02 | 4/79 (5%) | 0/79 (0%) | 3/79 (4%) | 72/79 (91%) |
| Mitochondrial Dysfunction | 4.22E+00 | 5.85E-02 | 2/171 (1%) | 0/171 (0%) | 8/171 (5%) | 161/171 (94%) |
| Role of NFAT in Regulation of the Immune Response | 4.22E+00 | 5.85E-02 | 6/171 (4%) | 0/171 (0%) | 4/171 (2%) | 161/171 (94%) |
| CD28 Signaling in T Helper Cells | 3.92E+00 | 6.78E-02 | 5/118 (4%) | 0/118 (0%) | 3/118 (3%) | 110/118 (93%) |
| Glucocorticoid Receptor Signaling | 3.72E+00 | 4.36E-02 | 7/275 (3%) | 0/275 (0%) | 5/275 (2%) | 263/275 (96%) |
| PI3K Signaling in B Lymphocytes | 3.70E+00 | 6.30E-02 | 5/127 (4%) | 0/127 (0%) | 3/127 (2%) | 119/127 (94%) |
| Phospholipase C Signaling | 3.68E+00 | 4.64E-02 | 8/237 (3%) | 0/237 (0%) | 3/237 (1%) | 226/237 (95%) |
| T Cell Receptor Signaling | 3.66E+00 | 7.22E-02 | 4/97 (4%) | 0/97 (0%) | 3/97 (3%) | 90/97 (93%) |
| Glioma Signaling | 3.63E+00 | 7.14E-02 | 1/98 (1%) | 0/98 (0%) | 6/98 (6%) | 91/98 (93%) |
| Renal Cell Carcinoma Signaling | 3.58E+00 | 8.45E-02 | 1/71 (1%) | 0/71 (0%) | 5/71 (7%) | 65/71 (92%) |
| B Cell Receptor Signaling | 3.45E+00 | 5.17E-02 | 6/174 (3%) | 0/174 (0%) | 3/174 (2%) | 165/174 (95%) |
| Systemic Lupus Erythematosus Signaling | 3.42E+00 | 4.67E-02 | 5/214 (2%) | 0/214 (0%) | 5/214 (2%) | 204/214 (95%) |
| fMLP Signaling in Neutrophils | 3.38E+00 | 6.48E-02 | 4/108 (4%) | 0/108 (0%) | 3/108 (3%) | 101/108 (94%) |
| iCOS-iCOSL Signaling in T Helper Cells | 3.38E+00 | 6.48E-02 | 4/108 (4%) | 0/108 (0%) | 3/108 (3%) | 101/108 (94%) |
| NRF2-mediated Oxidative Stress Response | 3.34E+00 | 5.00E-02 | 6/180 (3%) | 0/180 (0%) | 3/180 (2%) | 171/180 (95%) |
| Methylglyoxal Degradation I | 3.33E+00 | 6.67E-01 | 1/3 (33%) | 0/3 (0%) | 1/3 (33%) | 1/3 (33%) |
| Clathrin-mediated Endocytosis Signaling | 3.26E+00 | 4.86E-02 | 2/185 (1%) | 0/185 (0%) | 7/185 (4%) | 176/185 (95%) |
| Lymphotoxin β Receptor Signaling | 3.25E+00 | 9.26E-02 | 2/54 (4%) | 0/54 (0%) | 3/54 (6%) | 49/54 (91%) |
| mTOR Signaling | 3.22E+00 | 4.81E-02 | 5/187 (3%) | 0/187 (0%) | 4/187 (2%) | 178/187 (95%) |
| Xenobiotic Metabolism Signaling | 3.18E+00 | 4.04E-02 | 6/272 (2%) | 0/272 (0%) | 5/272 (2%) | 261/272 (96%) |
| PKCθ Signaling in T Lymphocytes | 3.15E+00 | 5.93E-02 | 4/118 (3%) | 0/118 (0%) | 3/118 (3%) | 111/118 (94%) |
| Vitamin-C Transport | 3.10E+00 | 2.00E-01 | 1/15 (7%) | 0/15 (0%) | 2/15 (13%) | 12/15 (80%) |
| PPAR Signaling | 2.95E+00 | 6.45E-02 | 2/93 (2%) | 0/93 (0%) | 4/93 (4%) | 87/93 (94%) |
| Role of Macrophages, Fibroblasts and Endothelial Cells in Rheumatoid Arthritis | 2.88E+00 | 3.72E-02 | 4/296 (1%) | 0/296 (0%) | 7/296 (2%) | 285/296 (96%) |
| Non-Small Cell Lung Cancer Signaling | 2.88E+00 | 7.69E-02 | 1/65 (2%) | 0/65 (0%) | 4/65 (6%) | 60/65 (92%) |
| Acute Phase Response Signaling | 2.88E+00 | 4.73E-02 | 1/169 (1%) | 0/169 (0%) | 7/169 (4%) | 161/169 (95%) |
| Angiopoietin Signaling | 2.85E+00 | 7.58E-02 | 1/66 (2%) | 0/66 (0%) | 4/66 (6%) | 61/66 (92%) |
| Telomerase Signaling | 2.81E+00 | 6.06E-02 | 0/99 (0%) | 0/99 (0%) | 6/99 (6%) | 93/99 (94%) |
| Ephrin Receptor Signaling | 2.80E+00 | 4.60E-02 | 2/174 (1%) | 0/174 (0%) | 6/174 (3%) | 166/174 (95%) |
| GDNF Family Ligand-Receptor Interactions | 2.79E+00 | 7.35E-02 | 1/68 (1%) | 0/68 (0%) | 4/68 (6%) | 63/68 (93%) |
| FcγRIIB Signaling in B Lymphocytes | 2.77E+00 | 9.76E-02 | 1/41 (2%) | 0/41 (0%) | 3/41 (7%) | 37/41 (90%) |
| Role of Osteoblasts, Osteoclasts and Chondrocytes in Rheumatoid Arthritis | 2.74E+00 | 4.11E-02 | 3/219 (1%) | 0/219 (0%) | 6/219 (3%) | 210/219 (96%) |
| AMPK Signaling | 2.73E+00 | 4.49E-02 | 3/178 (2%) | 0/178 (0%) | 5/178 (3%) | 170/178 (96%) |
| Small Cell Lung Cancer Signaling | 2.71E+00 | 7.04E-02 | 2/71 (3%) | 0/71 (0%) | 3/71 (4%) | 66/71 (93%) |
| Pancreatic Adenocarcinoma Signaling | 2.66E+00 | 5.66E-02 | 1/106 (1%) | 0/106 (0%) | 5/106 (5%) | 100/106 (94%) |
| Huntington's Disease Signaling | 2.61E+00 | 3.93E-02 | 3/229 (1%) | 0/229 (0%) | 6/229 (3%) | 220/229 (96%) |
| Oxidative Phosphorylation | 2.60E+00 | 5.50E-02 | 1/109 (1%) | 0/109 (0%) | 5/109 (5%) | 103/109 (94%) |
| Acute Myeloid Leukemia Signaling | 2.50E+00 | 6.33E-02 | 0/79 (0%) | 0/79 (0%) | 5/79 (6%) | 74/79 (94%) |
| TR/RXR Activation | 2.37E+00 | 5.88E-02 | 1/85 (1%) | 0/85 (0%) | 4/85 (5%) | 80/85 (94%) |
| PI3K/AKT Signaling | 2.34E+00 | 4.88E-02 | 2/123 (2%) | 0/123 (0%) | 4/123 (3%) | 117/123 (95%) |
| Neuregulin Signaling | 2.30E+00 | 5.68E-02 | 1/88 (1%) | 0/88 (0%) | 4/88 (5%) | 83/88 (94%) |
| OX40 Signaling Pathway | 2.28E+00 | 5.62E-02 | 4/89 (4%) | 0/89 (0%) | 1/89 (1%) | 84/89 (94%) |
| Glutathione-mediated Detoxification | 2.26E+00 | 1.03E-01 | 3/29 (10%) | 0/29 (0%) | 0/29 (0%) | 26/29 (90%) |
| Nur77 Signaling in T Lymphocytes | 2.25E+00 | 7.02E-02 | 4/57 (7%) | 0/57 (0%) | 0/57 (0%) | 53/57 (93%) |
| Chronic Myeloid Leukemia Signaling | 2.20E+00 | 5.38E-02 | 1/93 (1%) | 0/93 (0%) | 4/93 (4%) | 88/93 (95%) |
| Insulin Receptor Signaling | 2.19E+00 | 4.55E-02 | 2/132 (2%) | 0/132 (0%) | 4/132 (3%) | 126/132 (95%) |
| Molecular Mechanisms of Cancer | 2.19E+00 | 3.01E-02 | 6/365 (2%) | 0/365 (0%) | 5/365 (1%) | 354/365 (97%) |
| Induction of Apoptosis by HIV1 | 2.17E+00 | 6.67E-02 | 3/60 (5%) | 0/60 (0%) | 1/60 (2%) | 56/60 (93%) |
| ErbB4 Signaling | 2.17E+00 | 6.67E-02 | 0/60 (0%) | 0/60 (0%) | 4/60 (7%) | 56/60 (93%) |
| Role of NFAT in Cardiac Hypertrophy | 2.13E+00 | 3.91E-02 | 3/179 (2%) | 0/179 (0%) | 4/179 (2%) | 172/179 (96%) |
| IGF-1 Signaling | 2.13E+00 | 5.15E-02 | 1/97 (1%) | 0/97 (0%) | 4/97 (4%) | 92/97 (95%) |
| Role of JAK1 and JAK3 in γc Cytokine Signaling | 2.12E+00 | 6.45E-02 | 1/62 (2%) | 0/62 (0%) | 3/62 (5%) | 58/62 (94%) |
| Amyotrophic Lateral Sclerosis Signaling | 2.11E+00 | 5.10E-02 | 2/98 (2%) | 0/98 (0%) | 3/98 (3%) | 93/98 (95%) |
| p53 Signaling | 2.11E+00 | 5.10E-02 | 3/98 (3%) | 0/98 (0%) | 2/98 (2%) | 93/98 (95%) |
| Circadian Rhythm Signaling | 2.10E+00 | 9.09E-02 | 3/33 (9%) | 0/33 (0%) | 0/33 (0%) | 30/33 (91%) |
| Antioxidant Action of Vitamin C | 2.09E+00 | 5.05E-02 | 3/99 (3%) | 0/99 (0%) | 2/99 (2%) | 94/99 (95%) |
| Axonal Guidance Signaling | 2.06E+00 | 2.76E-02 | 4/434 (1%) | 0/434 (0%) | 8/434 (2%) | 422/434 (97%) |
| Hypoxia Signaling in the Cardiovascular System | 2.05E+00 | 6.15E-02 | 2/65 (3%) | 0/65 (0%) | 2/65 (3%) | 61/65 (94%) |
| HIF1α Signaling | 2.04E+00 | 4.90E-02 | 1/102 (1%) | 0/102 (0%) | 4/102 (4%) | 97/102 (95%) |
| Erythropoietin Signaling | 2.00E+00 | 5.97E-02 | 1/67 (1%) | 0/67 (0%) | 3/67 (4%) | 63/67 (94%) |
| Neurotrophin/TRK Signaling | 2.00E+00 | 5.97E-02 | 1/67 (1%) | 0/67 (0%) | 3/67 (4%) | 63/67 (94%) |
| Rac Signaling | 2.00E+00 | 4.81E-02 | 3/104 (3%) | 0/104 (0%) | 2/104 (2%) | 99/104 (95%) |
| Macropinocytosis Signaling | 1.98E+00 | 5.88E-02 | 0/68 (0%) | 0/68 (0%) | 4/68 (6%) | 64/68 (94%) |
| NGF Signaling | 1.95E+00 | 4.67E-02 | 2/107 (2%) | 0/107 (0%) | 3/107 (3%) | 102/107 (95%) |
| Fc Epsilon RI Signaling | 1.94E+00 | 4.63E-02 | 1/108 (1%) | 0/108 (0%) | 4/108 (4%) | 103/108 (95%) |
| Aldosterone Signaling in Epithelial Cells | 1.91E+00 | 3.95E-02 | 1/152 (1%) | 0/152 (0%) | 5/152 (3%) | 146/152 (96%) |
| Methylthiopropionate Biosynthesis | 1.90E+00 | 1.00E+00 | 0/1 (0%) | 0/1 (0%) | 1/1 (100%) | 0/1 (0%) |
| Docosahexaenoic Acid (DHA) Signaling | 1.90E+00 | 7.69E-02 | 1/39 (3%) | 0/39 (0%) | 2/39 (5%) | 36/39 (92%) |
| Netrin Signaling | 1.90E+00 | 7.69E-02 | 2/39 (5%) | 0/39 (0%) | 1/39 (3%) | 36/39 (92%) |
| Isoleucine Degradation I | 1.89E+00 | 1.43E-01 | 0/14 (0%) | 0/14 (0%) | 2/14 (14%) | 12/14 (86%) |
| Prolactin Signaling | 1.88E+00 | 5.48E-02 | 1/73 (1%) | 0/73 (0%) | 3/73 (4%) | 69/73 (95%) |
| FLT3 Signaling in Hematopoietic Progenitor Cells | 1.86E+00 | 5.41E-02 | 1/74 (1%) | 0/74 (0%) | 3/74 (4%) | 70/74 (95%) |
| VEGF Family Ligand-Receptor Interactions | 1.82E+00 | 5.26E-02 | 1/76 (1%) | 0/76 (0%) | 3/76 (4%) | 72/76 (95%) |
| IL-6 Signaling | 1.81E+00 | 4.31E-02 | 1/116 (1%) | 0/116 (0%) | 4/116 (3%) | 111/116 (96%) |
| Melanoma Signaling | 1.81E+00 | 7.14E-02 | 0/42 (0%) | 0/42 (0%) | 3/42 (7%) | 39/42 (93%) |
| p38 MAPK Signaling | 1.80E+00 | 4.27E-02 | 3/117 (3%) | 0/117 (0%) | 2/117 (2%) | 112/117 (96%) |
| p70S6K Signaling | 1.77E+00 | 4.20E-02 | 2/119 (2%) | 0/119 (0%) | 3/119 (3%) | 114/119 (96%) |
| P2Y Purigenic Receptor Signaling Pathway | 1.77E+00 | 4.20E-02 | 1/119 (1%) | 0/119 (0%) | 4/119 (3%) | 114/119 (96%) |
| phagosome maturation | 1.76E+00 | 4.17E-02 | 1/120 (1%) | 0/120 (0%) | 4/120 (3%) | 115/120 (96%) |
| Atherosclerosis Signaling | 1.70E+00 | 4.03E-02 | 1/124 (1%) | 0/124 (0%) | 4/124 (3%) | 119/124 (96%) |
| Valine Degradation I | 1.68E+00 | 1.11E-01 | 0/18 (0%) | 0/18 (0%) | 2/18 (11%) | 16/18 (89%) |
| NF-κB Signaling | 1.67E+00 | 3.49E-02 | 2/172 (1%) | 0/172 (0%) | 4/172 (2%) | 166/172 (97%) |
| RhoGDI Signaling | 1.66E+00 | 3.47E-02 | 5/173 (3%) | 0/173 (0%) | 1/173 (1%) | 167/173 (97%) |
| Cardiac Hypertrophy Signaling | 1.65E+00 | 3.14E-02 | 3/223 (1%) | 0/223 (0%) | 4/223 (2%) | 216/223 (97%) |
| ErbB Signaling | 1.64E+00 | 4.65E-02 | 0/86 (0%) | 0/86 (0%) | 4/86 (5%) | 82/86 (95%) |
| Glutathione Redox Reactions I | 1.64E+00 | 1.05E-01 | 2/19 (11%) | 0/19 (0%) | 0/19 (0%) | 17/19 (89%) |
| Hereditary Breast Cancer Signaling | 1.63E+00 | 3.88E-02 | 1/129 (1%) | 0/129 (0%) | 4/129 (3%) | 124/129 (96%) |
| Bladder Cancer Signaling | 1.62E+00 | 4.60E-02 | 1/87 (1%) | 0/87 (0%) | 3/87 (3%) | 83/87 (95%) |
| Dendritic Cell Maturation | 1.62E+00 | 3.39E-02 | 3/177 (2%) | 0/177 (0%) | 3/177 (2%) | 171/177 (97%) |
| Ovarian Cancer Signaling | 1.61E+00 | 3.82E-02 | 1/131 (1%) | 0/131 (0%) | 4/131 (3%) | 126/131 (96%) |
| G Beta Gamma Signaling | 1.61E+00 | 4.55E-02 | 0/88 (0%) | 0/88 (0%) | 4/88 (5%) | 84/88 (95%) |
| RANK Signaling in Osteoclasts | 1.61E+00 | 4.55E-02 | 2/88 (2%) | 0/88 (0%) | 2/88 (2%) | 84/88 (95%) |
| PPARα/RXRα Activation | 1.61E+00 | 3.37E-02 | 3/178 (2%) | 0/178 (0%) | 3/178 (2%) | 172/178 (97%) |
| Epoxysqualene Biosynthesis | 1.60E+00 | 5.00E-01 | 0/2 (0%) | 0/2 (0%) | 1/2 (50%) | 1/2 (50%) |
| PAK Signaling | 1.59E+00 | 4.49E-02 | 0/89 (0%) | 0/89 (0%) | 4/89 (4%) | 85/89 (96%) |
| Apoptosis Signaling | 1.59E+00 | 4.49E-02 | 2/89 (2%) | 0/89 (0%) | 2/89 (2%) | 85/89 (96%) |
| Adipogenesis pathway | 1.57E+00 | 3.73E-02 | 3/134 (2%) | 0/134 (0%) | 2/134 (1%) | 129/134 (96%) |
| Endometrial Cancer Signaling | 1.56E+00 | 5.77E-02 | 0/52 (0%) | 0/52 (0%) | 3/52 (6%) | 49/52 (94%) |
| Hepatic Fibrosis / Hepatic Stellate Cell Activation | 1.56E+00 | 3.28E-02 | 1/183 (1%) | 0/183 (0%) | 5/183 (3%) | 177/183 (97%) |
| IL-8 Signaling | 1.55E+00 | 3.26E-02 | 2/184 (1%) | 0/184 (0%) | 4/184 (2%) | 178/184 (97%) |
| Fcγ Receptor-mediated Phagocytosis in Macrophages and Monocytes | 1.53E+00 | 4.30E-02 | 2/93 (2%) | 0/93 (0%) | 2/93 (2%) | 89/93 (96%) |
| Thrombopoietin Signaling | 1.50E+00 | 5.45E-02 | 1/55 (2%) | 0/55 (0%) | 2/55 (4%) | 52/55 (95%) |
| RAR Activation | 1.49E+00 | 3.16E-02 | 2/190 (1%) | 0/190 (0%) | 4/190 (2%) | 184/190 (97%) |
| Actin Nucleation by ARP-WASP Complex | 1.48E+00 | 5.36E-02 | 1/56 (2%) | 0/56 (0%) | 2/56 (4%) | 53/56 (95%) |
| Wnt/Ca+ pathway | 1.48E+00 | 5.36E-02 | 3/56 (5%) | 0/56 (0%) | 0/56 (0%) | 53/56 (95%) |
| eNOS Signaling | 1.48E+00 | 3.52E-02 | 0/142 (0%) | 0/142 (0%) | 5/142 (4%) | 137/142 (96%) |
| ErbB2-ErbB3 Signaling | 1.46E+00 | 5.26E-02 | 0/57 (0%) | 0/57 (0%) | 3/57 (5%) | 54/57 (95%) |
| Myc Mediated Apoptosis Signaling | 1.44E+00 | 5.17E-02 | 1/58 (2%) | 0/58 (0%) | 2/58 (3%) | 55/58 (95%) |
| Regulation of eIF4 and p70S6K Signaling | 1.43E+00 | 3.42E-02 | 2/146 (1%) | 0/146 (0%) | 3/146 (2%) | 141/146 (97%) |
| Glioblastoma Multiforme Signaling | 1.43E+00 | 3.42E-02 | 1/146 (1%) | 0/146 (0%) | 4/146 (3%) | 141/146 (97%) |
| 5-aminoimidazole Ribonucleotide Biosynthesis I | 1.43E+00 | 3.33E-01 | 1/3 (33%) | 0/3 (0%) | 0/3 (0%) | 2/3 (67%) |
| Oxidized GTP and dGTP Detoxification | 1.43E+00 | 3.33E-01 | 1/3 (33%) | 0/3 (0%) | 0/3 (0%) | 2/3 (67%) |
| Gαq Signaling | 1.42E+00 | 3.40E-02 | 3/147 (2%) | 0/147 (0%) | 2/147 (1%) | 142/147 (97%) |
| Antiproliferative Role of TOB in T Cell Signaling | 1.38E+00 | 7.69E-02 | 0/26 (0%) | 0/26 (0%) | 2/26 (8%) | 24/26 (92%) |
| GM-CSF Signaling | 1.37E+00 | 4.84E-02 | 1/62 (2%) | 0/62 (0%) | 2/62 (3%) | 59/62 (95%) |
| Antiproliferative Role of Somatostatin Receptor 2 | 1.35E+00 | 4.76E-02 | 0/63 (0%) | 0/63 (0%) | 3/63 (5%) | 60/63 (95%) |
| ERK5 Signaling | 1.35E+00 | 4.76E-02 | 2/63 (3%) | 0/63 (0%) | 1/63 (2%) | 60/63 (95%) |
| Estrogen-Dependent Breast Cancer Signaling | 1.35E+00 | 4.76E-02 | 1/63 (2%) | 0/63 (0%) | 2/63 (3%) | 60/63 (95%) |
| Calcium-induced T Lymphocyte Apoptosis | 1.34E+00 | 4.69E-02 | 3/64 (5%) | 0/64 (0%) | 0/64 (0%) | 61/64 (95%) |
| Superpathway of Cholesterol Biosynthesis | 1.32E+00 | 7.14E-02 | 0/28 (0%) | 0/28 (0%) | 2/28 (7%) | 26/28 (93%) |
| Arsenate Detoxification I (Glutaredoxin) | 1.31E+00 | 2.50E-01 | 1/4 (25%) | 0/4 (0%) | 0/4 (0%) | 3/4 (75%) |
| Retinoate Biosynthesis II | 1.31E+00 | 2.50E-01 | 0/4 (0%) | 0/4 (0%) | 1/4 (25%) | 3/4 (75%) |
| 2-ketoglutarate Dehydrogenase Complex | 1.31E+00 | 2.50E-01 | 1/4 (25%) | 0/4 (0%) | 0/4 (0%) | 3/4 (75%) |
| Ascorbate Recycling (Cytosolic) | 1.31E+00 | 2.50E-01 | 1/4 (25%) | 0/4 (0%) | 0/4 (0%) | 3/4 (75%) |
| α-tocopherol Degradation | 1.31E+00 | 2.50E-01 | 1/4 (25%) | 0/4 (0%) | 0/4 (0%) | 3/4 (75%) |
| Natural Killer Cell Signaling | 1.31E+00 | 3.64E-02 | 0/110 (0%) | 0/110 (0%) | 4/110 (4%) | 106/110 (96%) |
