# Supplementary material for: Genomic deletion of GIT2 induces a premature age-related thymic dysfunction and systemic immune system disruption
Source: Aging (Albany NY). 2017 Mar 4;9(3):706–30. doi: 10.18632/aging.101185 (PMC5391227; doi:10.18632/aging.101185)
Supplement: Supplementary file 18 [file aging-09-706-s018.docx]

**Table S17. IPA-based canonical signaling pathway analysis of significantly regulated transcripts differential between GIT2KO spleen compared to WT controls.** For each canonical signaling pathway the –log_10_ p value, enrichment ratio (Ratio), downregulated pathway-populating transcript numbers (Downregulated) and upregulated pathway-populating transcript numbers (Upregulated) are represented.

| **Ingenuity Canonical Pathways** | **-log(p-value)** | **Ratio** | **Downregulated** | **No change** | **Upregulated** | **No overlap with dataset** |
| --- | --- | --- | --- | --- | --- | --- |
| iCOS-iCOSL Signaling in T Helper Cells | 7.25E+00 | 1.57E-01 | 15/108 (14%) | 0/108 (0%) | 2/108 (2%) | 91/108 (84%) |
| T Cell Receptor Signaling | 7.15E+00 | 1.65E-01 | 14/97 (14%) | 0/97 (0%) | 2/97 (2%) | 81/97 (84%) |
| Role of NFAT in Regulation of the Immune Response | 6.86E+00 | 1.23E-01 | 15/171 (9%) | 0/171 (0%) | 6/171 (4%) | 150/171 (88%) |
| CD28 Signaling in T Helper Cells | 6.67E+00 | 1.44E-01 | 13/118 (11%) | 0/118 (0%) | 4/118 (3%) | 101/118 (86%) |
| PKCθ Signaling in T Lymphocytes | 6.67E+00 | 1.44E-01 | 13/118 (11%) | 0/118 (0%) | 4/118 (3%) | 101/118 (86%) |
| Systemic Lupus Erythematosus Signaling | 5.82E+00 | 1.03E-01 | 12/214 (6%) | 0/214 (0%) | 10/214 (5%) | 192/214 (90%) |
| Glucocorticoid Receptor Signaling | 5.54E+00 | 9.09E-02 | 18/275 (7%) | 0/275 (0%) | 7/275 (3%) | 250/275 (91%) |
| Calcium-induced T Lymphocyte Apoptosis | 5.28E+00 | 1.72E-01 | 10/64 (16%) | 0/64 (0%) | 1/64 (2%) | 53/64 (83%) |
| Cdc42 Signaling | 5.16E+00 | 1.08E-01 | 13/167 (8%) | 0/167 (0%) | 5/167 (3%) | 149/167 (89%) |
| Nur77 Signaling in T Lymphocytes | 4.94E+00 | 1.75E-01 | 8/57 (14%) | 0/57 (0%) | 2/57 (4%) | 47/57 (82%) |
| AMPK Signaling | 4.77E+00 | 1.01E-01 | 6/178 (3%) | 0/178 (0%) | 12/178 (7%) | 160/178 (90%) |
| NRF2-mediated Oxidative Stress Response | 4.16E+00 | 9.44E-02 | 6/180 (3%) | 0/180 (0%) | 11/180 (6%) | 163/180 (91%) |
| CTLA4 Signaling in Cytotoxic T Lymphocytes | 3.95E+00 | 1.25E-01 | 8/88 (9%) | 0/88 (0%) | 3/88 (3%) | 77/88 (88%) |
| Mitochondrial Dysfunction | 3.91E+00 | 9.36E-02 | 0/171 (0%) | 0/171 (0%) | 16/171 (9%) | 155/171 (91%) |
| Oxidative Phosphorylation | 3.72E+00 | 1.10E-01 | 0/109 (0%) | 0/109 (0%) | 12/109 (11%) | 97/109 (89%) |
| Regulation of IL-2 Expression in Activated and Anergic T Lymphocytes | 3.69E+00 | 1.27E-01 | 9/79 (11%) | 0/79 (0%) | 1/79 (1%) | 69/79 (87%) |
| Type I Diabetes Mellitus Signaling | 3.68E+00 | 1.09E-01 | 10/110 (9%) | 0/110 (0%) | 2/110 (2%) | 98/110 (89%) |
| Phospholipase C Signaling | 3.63E+00 | 8.02E-02 | 13/237 (5%) | 0/237 (0%) | 6/237 (3%) | 218/237 (92%) |
| Glutathione-mediated Detoxification | 3.58E+00 | 2.07E-01 | 1/29 (3%) | 0/29 (0%) | 5/29 (17%) | 23/29 (79%) |
| Prostate Cancer Signaling | 3.55E+00 | 1.22E-01 | 4/82 (5%) | 0/82 (0%) | 6/82 (7%) | 72/82 (88%) |
| B Cell Receptor Signaling | 3.32E+00 | 8.62E-02 | 9/174 (5%) | 0/174 (0%) | 6/174 (3%) | 159/174 (91%) |
| Xenobiotic Metabolism Signaling | 3.30E+00 | 7.35E-02 | 9/272 (3%) | 0/272 (0%) | 11/272 (4%) | 252/272 (93%) |
| OX40 Signaling Pathway | 3.27E+00 | 1.12E-01 | 8/89 (9%) | 0/89 (0%) | 2/89 (2%) | 79/89 (89%) |
| B Cell Development | 3.26E+00 | 1.82E-01 | 4/33 (12%) | 0/33 (0%) | 2/33 (6%) | 27/33 (82%) |
| Primary Immunodeficiency Signaling | 3.11E+00 | 1.46E-01 | 7/48 (15%) | 0/48 (0%) | 0/48 (0%) | 41/48 (85%) |
| Natural Killer Cell Signaling | 3.10E+00 | 1.00E-01 | 8/110 (7%) | 0/110 (0%) | 3/110 (3%) | 99/110 (90%) |
| Antigen Presentation Pathway | 2.98E+00 | 1.62E-01 | 6/37 (16%) | 0/37 (0%) | 0/37 (0%) | 31/37 (84%) |
| Remodeling of Epithelial Adherens Junctions | 2.85E+00 | 1.18E-01 | 3/68 (4%) | 0/68 (0%) | 5/68 (7%) | 60/68 (88%) |
| Leukocyte Extravasation Signaling | 2.74E+00 | 7.58E-02 | 11/198 (6%) | 0/198 (0%) | 4/198 (2%) | 183/198 (92%) |
| Aryl Hydrocarbon Receptor Signaling | 2.74E+00 | 8.57E-02 | 4/140 (3%) | 0/140 (0%) | 8/140 (6%) | 128/140 (91%) |
| Germ Cell-Sertoli Cell Junction Signaling | 2.72E+00 | 8.12E-02 | 5/160 (3%) | 0/160 (0%) | 8/160 (5%) | 147/160 (92%) |
| Ephrin B Signaling | 2.65E+00 | 1.10E-01 | 4/73 (5%) | 0/73 (0%) | 4/73 (5%) | 65/73 (89%) |
| EIF2 Signaling | 2.61E+00 | 7.61E-02 | 4/184 (2%) | 0/184 (0%) | 10/184 (5%) | 170/184 (92%) |
| IL-4 Signaling | 2.57E+00 | 1.07E-01 | 5/75 (7%) | 0/75 (0%) | 3/75 (4%) | 67/75 (89%) |
| Glutathione Redox Reactions I | 2.56E+00 | 2.11E-01 | 1/19 (5%) | 0/19 (0%) | 3/19 (16%) | 15/19 (79%) |
| Hereditary Breast Cancer Signaling | 2.54E+00 | 8.53E-02 | 5/129 (4%) | 0/129 (0%) | 6/129 (5%) | 118/129 (91%) |
| Fcγ Receptor-mediated Phagocytosis in Macrophages and Monocytes | 2.54E+00 | 9.68E-02 | 5/93 (5%) | 0/93 (0%) | 4/93 (4%) | 84/93 (90%) |
| Methylglyoxal Degradation I | 2.52E+00 | 6.67E-01 | 0/3 (0%) | 0/3 (0%) | 2/3 (67%) | 1/3 (33%) |
| Agranulocyte Adhesion and Diapedesis | 2.51E+00 | 7.41E-02 | 9/189 (5%) | 0/189 (0%) | 5/189 (3%) | 175/189 (93%) |
| PDGF Signaling | 2.50E+00 | 1.04E-01 | 5/77 (6%) | 0/77 (0%) | 3/77 (4%) | 69/77 (90%) |
| Glioma Signaling | 2.38E+00 | 9.18E-02 | 4/98 (4%) | 0/98 (0%) | 5/98 (5%) | 89/98 (91%) |
| Actin Cytoskeleton Signaling | 2.38E+00 | 6.94E-02 | 8/216 (4%) | 0/216 (0%) | 7/216 (3%) | 201/216 (93%) |
| Gap Junction Signaling | 2.38E+00 | 7.74E-02 | 7/155 (5%) | 0/155 (0%) | 5/155 (3%) | 143/155 (92%) |
| 3-phosphoinositide Biosynthesis | 2.36E+00 | 7.69E-02 | 8/156 (5%) | 0/156 (0%) | 4/156 (3%) | 144/156 (92%) |
| Nucleotide Excision Repair Pathway | 2.32E+00 | 1.43E-01 | 4/35 (11%) | 0/35 (0%) | 1/35 (3%) | 30/35 (86%) |
| Sertoli Cell-Sertoli Cell Junction Signaling | 2.32E+00 | 7.30E-02 | 6/178 (3%) | 0/178 (0%) | 7/178 (4%) | 165/178 (93%) |
| α-tocopherol Degradation | 2.23E+00 | 5.00E-01 | 2/4 (50%) | 0/4 (0%) | 0/4 (0%) | 2/4 (50%) |
| Rac Signaling | 2.21E+00 | 8.65E-02 | 5/104 (5%) | 0/104 (0%) | 4/104 (4%) | 95/104 (91%) |
| PI3K/AKT Signaling | 2.21E+00 | 8.13E-02 | 5/123 (4%) | 0/123 (0%) | 5/123 (4%) | 113/123 (92%) |
| CCR5 Signaling in Macrophages | 2.19E+00 | 1.01E-01 | 5/69 (7%) | 0/69 (0%) | 2/69 (3%) | 62/69 (90%) |
| 3-phosphoinositide Degradation | 2.14E+00 | 7.53E-02 | 8/146 (5%) | 0/146 (0%) | 3/146 (2%) | 135/146 (92%) |
| Epithelial Adherens Junction Signaling | 2.14E+00 | 7.53E-02 | 5/146 (3%) | 0/146 (0%) | 6/146 (4%) | 135/146 (92%) |
| T Helper Cell Differentiation | 2.13E+00 | 9.86E-02 | 6/71 (8%) | 0/71 (0%) | 1/71 (1%) | 64/71 (90%) |
| Chemokine Signaling | 2.13E+00 | 9.86E-02 | 4/71 (6%) | 0/71 (0%) | 3/71 (4%) | 64/71 (90%) |
| PI3K Signaling in B Lymphocytes | 2.12E+00 | 7.87E-02 | 6/127 (5%) | 0/127 (0%) | 4/127 (3%) | 117/127 (92%) |
| GNRH Signaling | 2.07E+00 | 7.75E-02 | 5/129 (4%) | 0/129 (0%) | 5/129 (4%) | 119/129 (92%) |
| D-myo-inositol (1,4,5,6)-Tetrakisphosphate Biosynthesis | 2.07E+00 | 7.75E-02 | 7/129 (5%) | 0/129 (0%) | 3/129 (2%) | 119/129 (92%) |
| D-myo-inositol (3,4,5,6)-tetrakisphosphate Biosynthesis | 2.07E+00 | 7.75E-02 | 7/129 (5%) | 0/129 (0%) | 3/129 (2%) | 119/129 (92%) |
| B Cell Activating Factor Signaling | 2.07E+00 | 1.25E-01 | 5/40 (13%) | 0/40 (0%) | 0/40 (0%) | 35/40 (88%) |
| Breast Cancer Regulation by Stathmin1 | 2.07E+00 | 6.81E-02 | 5/191 (3%) | 0/191 (0%) | 8/191 (4%) | 178/191 (93%) |
| Superpathway of Inositol Phosphate Compounds | 2.03E+00 | 6.74E-02 | 9/193 (5%) | 0/193 (0%) | 4/193 (2%) | 180/193 (93%) |
| NF-κB Signaling | 2.03E+00 | 6.98E-02 | 9/172 (5%) | 0/172 (0%) | 3/172 (2%) | 160/172 (93%) |
| Glioma Invasiveness Signaling | 2.02E+00 | 1.05E-01 | 3/57 (5%) | 0/57 (0%) | 3/57 (5%) | 51/57 (89%) |
| FcγRIIB Signaling in B Lymphocytes | 2.02E+00 | 1.22E-01 | 2/41 (5%) | 0/41 (0%) | 3/41 (7%) | 36/41 (88%) |
| RhoGDI Signaling | 2.01E+00 | 6.94E-02 | 8/173 (5%) | 0/173 (0%) | 4/173 (2%) | 161/173 (93%) |
| Ephrin Receptor Signaling | 1.99E+00 | 6.90E-02 | 4/174 (2%) | 0/174 (0%) | 8/174 (5%) | 162/174 (93%) |
| Superpathway of Cholesterol Biosynthesis | 1.94E+00 | 1.43E-01 | 1/28 (4%) | 0/28 (0%) | 3/28 (11%) | 24/28 (86%) |
| Gα12/13 Signaling | 1.89E+00 | 7.69E-02 | 7/117 (6%) | 0/117 (0%) | 2/117 (2%) | 108/117 (92%) |
| TNFR2 Signaling | 1.89E+00 | 1.38E-01 | 4/29 (14%) | 0/29 (0%) | 0/29 (0%) | 25/29 (86%) |
| CDK5 Signaling | 1.85E+00 | 8.08E-02 | 4/99 (4%) | 0/99 (0%) | 4/99 (4%) | 91/99 (92%) |
| Telomerase Signaling | 1.85E+00 | 8.08E-02 | 4/99 (4%) | 0/99 (0%) | 4/99 (4%) | 91/99 (92%) |
| Hepatic Fibrosis / Hepatic Stellate Cell Activation | 1.83E+00 | 6.56E-02 | 7/183 (4%) | 0/183 (0%) | 5/183 (3%) | 171/183 (93%) |
| Integrin Signaling | 1.80E+00 | 6.28E-02 | 4/207 (2%) | 0/207 (0%) | 9/207 (4%) | 194/207 (94%) |
| Clathrin-mediated Endocytosis Signaling | 1.80E+00 | 6.49E-02 | 6/185 (3%) | 0/185 (0%) | 6/185 (3%) | 173/185 (94%) |
| ILK Signaling | 1.80E+00 | 6.49E-02 | 4/185 (2%) | 0/185 (0%) | 8/185 (4%) | 173/185 (94%) |
| Autoimmune Thyroid Disease Signaling | 1.78E+00 | 1.06E-01 | 4/47 (9%) | 0/47 (0%) | 1/47 (2%) | 42/47 (89%) |
| ERK/MAPK Signaling | 1.76E+00 | 6.42E-02 | 7/187 (4%) | 0/187 (0%) | 5/187 (3%) | 175/187 (94%) |
| CD40 Signaling | 1.76E+00 | 9.23E-02 | 4/65 (6%) | 0/65 (0%) | 2/65 (3%) | 59/65 (91%) |
| Non-Small Cell Lung Cancer Signaling | 1.76E+00 | 9.23E-02 | 3/65 (5%) | 0/65 (0%) | 3/65 (5%) | 59/65 (91%) |
| Graft-versus-Host Disease Signaling | 1.74E+00 | 1.04E-01 | 4/48 (8%) | 0/48 (0%) | 1/48 (2%) | 43/48 (90%) |
| D-myo-inositol-5-phosphate Metabolism | 1.74E+00 | 6.90E-02 | 7/145 (5%) | 0/145 (0%) | 3/145 (2%) | 135/145 (93%) |
| RAR Activation | 1.71E+00 | 6.32E-02 | 7/190 (4%) | 0/190 (0%) | 5/190 (3%) | 178/190 (94%) |
| Acute Phase Response Signaling | 1.70E+00 | 6.51E-02 | 5/169 (3%) | 0/169 (0%) | 6/169 (4%) | 158/169 (93%) |
| Macropinocytosis Signaling | 1.67E+00 | 8.82E-02 | 3/68 (4%) | 0/68 (0%) | 3/68 (4%) | 62/68 (91%) |
| Fc Epsilon RI Signaling | 1.65E+00 | 7.41E-02 | 5/108 (5%) | 0/108 (0%) | 3/108 (3%) | 100/108 (93%) |
| fMLP Signaling in Neutrophils | 1.65E+00 | 7.41E-02 | 2/108 (2%) | 0/108 (0%) | 6/108 (6%) | 100/108 (93%) |
| Altered T Cell and B Cell Signaling in Rheumatoid Arthritis | 1.65E+00 | 7.95E-02 | 5/88 (6%) | 0/88 (0%) | 2/88 (2%) | 81/88 (92%) |
| RANK Signaling in Osteoclasts | 1.65E+00 | 7.95E-02 | 5/88 (6%) | 0/88 (0%) | 2/88 (2%) | 81/88 (92%) |
| Renin-Angiotensin Signaling | 1.63E+00 | 7.34E-02 | 5/109 (5%) | 0/109 (0%) | 3/109 (3%) | 101/109 (93%) |
| Endometrial Cancer Signaling | 1.61E+00 | 9.62E-02 | 2/52 (4%) | 0/52 (0%) | 3/52 (6%) | 47/52 (90%) |
| Ovarian Cancer Signaling | 1.61E+00 | 6.87E-02 | 5/131 (4%) | 0/131 (0%) | 4/131 (3%) | 122/131 (93%) |
| Small Cell Lung Cancer Signaling | 1.59E+00 | 8.45E-02 | 4/71 (6%) | 0/71 (0%) | 2/71 (3%) | 65/71 (92%) |
| Molecular Mechanisms of Cancer | 1.58E+00 | 5.21E-02 | 13/365 (4%) | 0/365 (0%) | 6/365 (2%) | 346/365 (95%) |
| IL-1 Signaling | 1.58E+00 | 7.69E-02 | 4/91 (4%) | 0/91 (0%) | 3/91 (3%) | 84/91 (92%) |
| Cardiac β-adrenergic Signaling | 1.57E+00 | 6.77E-02 | 4/133 (3%) | 0/133 (0%) | 5/133 (4%) | 124/133 (93%) |
| Dendritic Cell Maturation | 1.57E+00 | 6.21E-02 | 7/177 (4%) | 0/177 (0%) | 4/177 (2%) | 166/177 (94%) |
| Granulocyte Adhesion and Diapedesis | 1.57E+00 | 6.21E-02 | 7/177 (4%) | 0/177 (0%) | 4/177 (2%) | 166/177 (94%) |
| IL-17 Signaling | 1.56E+00 | 8.33E-02 | 3/72 (4%) | 0/72 (0%) | 3/72 (4%) | 66/72 (92%) |
| Lymphotoxin β Receptor Signaling | 1.54E+00 | 9.26E-02 | 4/54 (7%) | 0/54 (0%) | 1/54 (2%) | 49/54 (91%) |
| FLT3 Signaling in Hematopoietic Progenitor Cells | 1.51E+00 | 8.11E-02 | 3/74 (4%) | 0/74 (0%) | 3/74 (4%) | 68/74 (92%) |
| Toll-like Receptor Signaling | 1.51E+00 | 8.11E-02 | 5/74 (7%) | 0/74 (0%) | 1/74 (1%) | 68/74 (92%) |
| SAPK/JNK Signaling | 1.51E+00 | 7.45E-02 | 3/94 (3%) | 0/94 (0%) | 4/94 (4%) | 87/94 (93%) |
| Methylthiopropionate Biosynthesis | 1.50E+00 | 1.00E+00 | 0/1 (0%) | 0/1 (0%) | 1/1 (100%) | 0/1 (0%) |
| Asparagine Biosynthesis I | 1.50E+00 | 1.00E+00 | 0/1 (0%) | 0/1 (0%) | 1/1 (100%) | 0/1 (0%) |
| Adenine and Adenosine Salvage VI | 1.50E+00 | 1.00E+00 | 0/1 (0%) | 0/1 (0%) | 1/1 (100%) | 0/1 (0%) |
| April Mediated Signaling | 1.49E+00 | 1.05E-01 | 4/38 (11%) | 0/38 (0%) | 0/38 (0%) | 34/38 (89%) |
| HER-2 Signaling in Breast Cancer | 1.46E+00 | 7.89E-02 | 3/76 (4%) | 0/76 (0%) | 3/76 (4%) | 70/76 (92%) |
| IGF-1 Signaling | 1.45E+00 | 7.22E-02 | 4/97 (4%) | 0/97 (0%) | 3/97 (3%) | 90/97 (93%) |
| p70S6K Signaling | 1.43E+00 | 6.72E-02 | 2/119 (2%) | 0/119 (0%) | 6/119 (5%) | 111/119 (93%) |
| P2Y Purigenic Receptor Signaling Pathway | 1.43E+00 | 6.72E-02 | 2/119 (2%) | 0/119 (0%) | 6/119 (5%) | 111/119 (93%) |
| Neuroprotective Role of THOP1 in Alzheimer's Disease | 1.42E+00 | 1.00E-01 | 3/40 (8%) | 0/40 (0%) | 1/40 (3%) | 36/40 (90%) |
| Signaling by Rho Family GTPases | 1.42E+00 | 5.56E-02 | 6/234 (3%) | 0/234 (0%) | 7/234 (3%) | 221/234 (94%) |
| Dopamine Receptor Signaling | 1.41E+00 | 7.69E-02 | 2/78 (3%) | 0/78 (0%) | 4/78 (5%) | 72/78 (92%) |
| ATM Signaling | 1.40E+00 | 8.47E-02 | 3/59 (5%) | 0/59 (0%) | 2/59 (3%) | 54/59 (92%) |
| Reelin Signaling in Neurons | 1.39E+00 | 7.59E-02 | 3/79 (4%) | 0/79 (0%) | 3/79 (4%) | 73/79 (92%) |
| Acute Myeloid Leukemia Signaling | 1.39E+00 | 7.59E-02 | 2/79 (3%) | 0/79 (0%) | 4/79 (5%) | 73/79 (92%) |
| RhoA Signaling | 1.38E+00 | 6.56E-02 | 4/122 (3%) | 0/122 (0%) | 4/122 (3%) | 114/122 (93%) |
| Ceramide Signaling | 1.37E+00 | 7.50E-02 | 2/80 (3%) | 0/80 (0%) | 4/80 (5%) | 74/80 (93%) |
| Paxillin Signaling | 1.37E+00 | 6.93E-02 | 4/101 (4%) | 0/101 (0%) | 3/101 (3%) | 94/101 (93%) |
| Melanoma Signaling | 1.35E+00 | 9.52E-02 | 2/42 (5%) | 0/42 (0%) | 2/42 (5%) | 38/42 (90%) |
| UVC-Induced MAPK Signaling | 1.35E+00 | 9.52E-02 | 3/42 (7%) | 0/42 (0%) | 1/42 (2%) | 38/42 (90%) |
| Wnt/β-catenin Signaling | 1.34E+00 | 5.92E-02 | 7/169 (4%) | 0/169 (0%) | 3/169 (2%) | 159/169 (94%) |
| GM-CSF Signaling | 1.32E+00 | 8.06E-02 | 3/62 (5%) | 0/62 (0%) | 2/62 (3%) | 57/62 (92%) |
| CREB Signaling in Neurons | 1.32E+00 | 5.85E-02 | 4/171 (2%) | 0/171 (0%) | 6/171 (4%) | 161/171 (94%) |
| IL-15 Signaling | 1.30E+00 | 7.94E-02 | 3/63 (5%) | 0/63 (0%) | 2/63 (3%) | 58/63 (92%) |
| Estrogen-Dependent Breast Cancer Signaling | 1.30E+00 | 7.94E-02 | 2/63 (3%) | 0/63 (0%) | 3/63 (5%) | 58/63 (92%) |
