# Supplementary material for: Genomic deletion of GIT2 induces a premature age-related thymic dysfunction and systemic immune system disruption
Source: Aging (Albany NY). 2017 Mar 4;9(3):706–30. doi: 10.18632/aging.101185 (PMC5391227; doi:10.18632/aging.101185)
Supplement: Supplementary file 19 [file aging-09-706-s019.docx]

**Table S18. Venn diagram analysis of significantly populated Ingenuity Pathway Analysis canonical signaling pathways from GIT2KO versus WT control ILN, MLN, spleen and thymus transcriptomic data.** For each significantly-populated signaling pathway the Z score (calculated by subtracting the number of downregulated transcripts populating the specific pathway from the number of upregulated transcripts populating the specific pathway. A Z score of 0 was nominally considered to be a positive value.

| **Canonical Signaling Pathway** | **ILN Z score** | **MLN Z score** | **Spleen Z score** | **Thymus Z score** |
| --- | --- | --- | --- | --- |
| Mitochondrial Dysfunction | 5 | 6 | 16 | 24 |
| Ephrin Receptor Signaling | 7 | 4 | 4 | 10 |
| AMPK Signaling | 4 | 2 | 6 | 5 |
| P2Y Purigenic Receptor Signaling Pathway | 4 | 3 | 4 | 4 |
| p70S6K Signaling | 4 | 1 | 4 | 4 |
| Acute Phase Response Signaling | 2 | 6 | 1 | 2 |
| Clathrin-mediated Endocytosis Signaling | 5 | 5 | 0 | 0 |
| Telomerase Signaling | 2 | 6 | 0 | 2 |
| Superpathway of Cholesterol Biosynthesis | 1 | 2 | 2 | 3 |
| PI3K/AKT Signaling | 4 | 2 | 0 | 1 |
| Prostate Cancer Signaling | 3 | 2 | 2 | 0 |
| Estrogen-Dependent Breast Cancer Signaling | 3 | 1 | 1 | 2 |
| FcγRIIB Signaling in B Lymphocytes | 3 | 2 | 1 | 0 |
|  |  |  |  |  |
| PKCθ Signaling in T Lymphocytes | -1 | -1 | -9 | -4 |
| CD28 Signaling in T Helper Cells | -2 | -2 | -9 | -4 |
| Molecular Mechanisms of Cancer | -2 | -1 | -7 | -8 |
| iCOS-iCOSL Signaling in T Helper Cells | -3 | -1 | -13 | -6 |
|  |  |  |  |  |
| Dendritic Cell Maturation | 8 | 0 | -3 | -6 |
| Systemic Lupus Erythematosus Signaling | 6 | 0 | -2 | 1 |
| fMLP Signaling in Neutrophils | 6 | -1 | 4 | 3 |
| RhoGDI Signaling | 5 | -4 | -4 | -2 |
| Hereditary Breast Cancer Signaling | 4 | 3 | 1 | -1 |
| Rac Signaling | 4 | -1 | -1 | -1 |
| PI3K Signaling in B Lymphocytes | 4 | -2 | -2 | -2 |
| NF-κB Signaling | 3 | 2 | -6 | 1 |
| Ovarian Cancer Signaling | 3 | 3 | -1 | 0 |
| Melanoma Signaling | 3 | 3 | 0 | -1 |
| Fcγ Receptor-mediated Phagocytosis in Macrophages and Monocytes | 3 | 0 | -1 | -2 |
| Role of NFAT in Regulation of the Immune Response | 2 | -2 | -9 | -4 |
| Glucocorticoid Receptor Signaling | 2 | -2 | -11 | -2 |
| IGF-1 Signaling | 2 | 3 | -1 | 2 |
| GM-CSF Signaling | 2 | 1 | -1 | -2 |
| Non-Small Cell Lung Cancer Signaling | 2 | 3 | 0 | -2 |
| Endometrial Cancer Signaling | 2 | 3 | 1 | -1 |
| Fc Epsilon RI Signaling | 2 | 3 | -2 | -1 |
| Glioma Signaling | 1 | 5 | 1 | -1 |
| Macropinocytosis Signaling | 1 | 4 | 0 | -1 |
| Small Cell Lung Cancer Signaling | 1 | 1 | -2 | -4 |
| Natural Killer Cell Signaling | 0 | 4 | -5 | -2 |
| RAR Activation | 0 | 2 | -2 | -4 |
| Aryl Hydrocarbon Receptor Signaling | -1 | 0 | 4 | 2 |
| Glutathione-mediated Detoxification | -2 | -3 | 4 | 4 |
| NRF2-mediated Oxidative Stress Response | -3 | -3 | 5 | 2 |
| Glutathione Redox Reactions I | -3 | -2 | 2 | 4 |
| Xenobiotic Metabolism Signaling | -3 | -1 | 2 | 3 |
|  |  |  |  |  |
| Signaling by Rho Family GTPases | 7 |  | 1 | 1 |
| Integrin Signaling | 6 |  | 5 | 4 |
| Epithelial Adherens Junction Signaling | 5 |  | 1 | 0 |
| CREB Signaling in Neurons | 4 |  | 2 | 3 |
| Breast Cancer Regulation by Stathmin1 | 3 |  | 3 | 3 |
| Cardiac β-adrenergic Signaling | 2 |  | 1 | 3 |
|  |  |  |  |  |
| Leukocyte Extravasation Signaling | -4 |  | -7 | -6 |
|  |  |  |  |  |
| PDGF Signaling | 7 |  | -2 | 1 |
| Actin Cytoskeleton Signaling | 7 |  | -1 | -1 |
| T Helper Cell Differentiation | 5 |  | -5 | -6 |
| IL-4 Signaling | 5 |  | -2 | -1 |
| Gap Junction Signaling | 5 |  | -2 | -1 |
| RhoA Signaling | 5 |  | 0 | -2 |
| ERK/MAPK Signaling | 5 |  | -2 | 5 |
| Autoimmune Thyroid Disease Signaling | 4 |  | -3 | -2 |
| Graft-versus-Host Disease Signaling | 4 |  | -3 | -2 |
| Remodeling of Epithelial Adherens Junctions | 4 |  | 2 | -1 |
| Type I Diabetes Mellitus Signaling | 3 |  | -8 | -6 |
| Superpathway of Inositol Phosphate Compounds | 3 |  | -5 | -3 |
| Renin-Angiotensin Signaling | 3 |  | -2 | -2 |
| Antigen Presentation Pathway | 2 |  | -6 | -3 |
| CD40 Signaling | 2 |  | -2 | -2 |
| 3-phosphoinositide Biosynthesis | 1 |  | -4 | -3 |
| SAPK/JNK Signaling | 1 |  | 1 | -2 |
| Paxillin Signaling | 1 |  | -1 | -3 |
| IL-15 Signaling | 0 |  | -1 | -1 |
| Glioma Invasiveness Signaling | -1 |  | 0 | -1 |
| HER-2 Signaling in Breast Cancer | -1 |  | 0 | -4 |
| CTLA4 Signaling in Cytotoxic T Lymphocytes | -7 |  | -5 | 0 |
|  |  |  |  |  |
| PPARα/RXRα Activation | 7 | 0 |  | 3 |
| G Beta Gamma Signaling | 7 | 4 |  | 7 |
| eNOS Signaling | 6 | 5 |  | 2 |
| Cardiac Hypertrophy Signaling | 6 | 1 |  | 3 |
| Renal Cell Carcinoma Signaling | 4 | 4 |  | 1 |
| Role of NFAT in Cardiac Hypertrophy | 3 | 1 |  | 0 |
| Antiproliferative Role of Somatostatin Receptor 2 | 3 | 3 |  | 2 |
| Insulin Receptor Signaling | 3 | 2 |  | 3 |
| Neuregulin Signaling | 2 | 3 |  | 1 |
| Myc Mediated Apoptosis Signaling | 2 | 1 |  | 1 |
| TR/RXR Activation | 1 | 3 |  | 0 |
|  |  |  |  |  |
| Pancreatic Adenocarcinoma Signaling | 5 | 4 |  | -4 |
| IL-8 Signaling | 5 | 2 |  | -1 |
| Prolactin Signaling | 4 | 2 |  | -2 |
| Role of JAK1 and JAK3 in γc Cytokine Signaling | 3 | 2 |  | -1 |
| Thrombopoietin Signaling | 3 | 1 |  | -1 |
| Chronic Myeloid Leukemia Signaling | 2 | 3 |  | -1 |
| Aldosterone Signaling in Epithelial Cells | 2 | 4 |  | -1 |
| ErbB4 Signaling | 2 | 4 |  | -1 |
| Glioblastoma Multiforme Signaling | 2 | 3 |  | -3 |
| ErbB2-ErbB3 Signaling | 2 | 3 |  | -1 |
| GDNF Family Ligand-Receptor Interactions | 2 | 3 |  | -2 |
| p53 Signaling | 1 | -1 |  | -4 |
| mTOR Signaling | 0 | -1 |  | 0 |
|  |  |  |  |  |
| Acute Myeloid Leukemia Signaling |  | 5 | 2 | 0 |
| Oxidative Phosphorylation |  | 4 | 12 | 19 |
| FLT3 Signaling in Hematopoietic Progenitor Cells |  | 2 | 0 | 0 |
| Methylglyoxal Degradation I |  | 0 | 2 | 0 |
|  |  |  |  |  |
| α-tocopherol Degradation |  | -1 | -2 | -2 |
|  |  |  |  |  |
| Lymphotoxin β Receptor Signaling |  | 1 | -3 | -4 |
|  |  |  |  |  |
| Nur77 Signaling in T Lymphocytes | -1 | -4 | -6 |  |
| Calcium-induced T Lymphocyte Apoptosis | -2 | -3 | -9 |  |
| Regulation of IL-2 Expression in Activated and Anergic T Lymphocytes | -2 | -1 | -8 |  |
| Phospholipase C Signaling | -4 | -5 | -7 |  |
| T Cell Receptor Signaling | -7 | -1 | -12 |  |
|  |  |  |  |  |
| B Cell Receptor Signaling | 6 | -3 | -3 |  |
| Hepatic Fibrosis / Hepatic Stellate Cell Activation | 3 | 4 | -2 |  |
| OX40 Signaling Pathway | 2 | -3 | -6 |  |
|  |  |  |  |  |
| Virus Entry via Endocytic Pathways | 5 |  |  | 0 |
| Colorectal Cancer Metastasis Signaling | 5 |  |  | 1 |
| CCR3 Signaling in Eosinophils | 5 |  |  | 1 |
| Gαi Signaling | 5 |  |  | 5 |
| α-Adrenergic Signaling | 4 |  |  | 3 |
| Corticotropin Releasing Hormone Signaling | 4 |  |  | 1 |
| Androgen Signaling | 4 |  |  | 1 |
| 14-3-3-mediated Signaling | 3 |  |  | 0 |
| Protein Kinase A Signaling | 3 |  |  | 1 |
| TCA Cycle II (Eukaryotic) | 3 |  |  | 7 |
| FAK Signaling | 3 |  |  | 0 |
| Cell Cycle Regulation by BTG Family Proteins | 3 |  |  | 2 |
| PTEN Signaling | 2 |  |  | 1 |
| Leptin Signaling in Obesity | 2 |  |  | 0 |
| Relaxin Signaling | 2 |  |  | 1 |
| Melanocyte Development and Pigmentation Signaling | 2 |  |  | 2 |
| CXCR4 Signaling | 1 |  |  | 2 |
|  |  |  |  |  |
| Mouse Embryonic Stem Cell Pluripotency | -1 |  |  | -2 |
|  |  |  |  |  |
| Protein Ubiquitination Pathway | 5 |  |  | -3 |
| Tec Kinase Signaling | 4 |  |  | -1 |
| Thrombin Signaling | 4 |  |  | -1 |
| Caveolar-mediated Endocytosis Signaling | 3 |  |  | -1 |
| UVA-Induced MAPK Signaling | 3 |  |  | -2 |
| JAK/Stat Signaling | 2 |  |  | -2 |
| Nitric Oxide Signaling in the Cardiovascular System | 2 |  |  | -2 |
| IL-3 Signaling | 2 |  |  | -1 |
| IL-9 Signaling | 1 |  |  | -2 |
| CNTF Signaling | 1 |  |  | -2 |
| Role of JAK family kinases in IL-6-type Cytokine Signaling | 1 |  |  | -3 |
| Role of p14/p19ARF in Tumor Suppression | 1 |  |  | -2 |
| Cell Cycle: G1/S Checkpoint Regulation | 0 |  |  | -3 |
| Superpathway of Geranylgeranyldiphosphate Biosynthesis I (via Mevalonate) | -1 |  |  | 1 |
|  |  |  |  |  |
| Huntington's Disease Signaling |  | 3 |  | 4 |
| Neurotrophin/TRK Signaling |  | 2 |  | 1 |
| Docosahexaenoic Acid (DHA) Signaling |  | 1 |  | 1 |
| Regulation of eIF4 and p70S6K Signaling |  | 1 |  | 4 |
| Hypoxia Signaling in the Cardiovascular System |  | 0 |  | 1 |
|  |  |  |  |  |
| Gαq Signaling |  | -1 |  | -1 |
|  |  |  |  |  |
| Angiopoietin Signaling |  | 3 |  | -1 |
| phagosome maturation |  | 3 |  | -2 |
|  |  |  |  |  |
| Ephrin B Signaling | 3 |  | 0 |  |
|  |  |  |  |  |
| Agranulocyte Adhesion and Diapedesis | -1 |  | -4 |  |
| Granulocyte Adhesion and Diapedesis | -2 |  | -3 |  |
| Primary Immunodeficiency Signaling | -3 |  | -7 |  |
|  |  |  |  |  |
| Altered T Cell and B Cell Signaling in Rheumatoid Arthritis | 8 |  | -3 |  |
| Cdc42 Signaling | 5 |  | -8 |  |
| B Cell Development | 4 |  | -2 |  |
| D-myo-inositol-5-phosphate Metabolism | 2 |  | -4 |  |
| 3-phosphoinositide Degradation | 2 |  | -5 |  |
| CCR5 Signaling in Macrophages | 1 |  | -3 |  |
| Chemokine Signaling | 1 |  | -1 |  |
| D-myo-inositol (1,4,5,6)-Tetrakisphosphate Biosynthesis | 1 |  | -4 |  |
| D-myo-inositol (3,4,5,6)-tetrakisphosphate Biosynthesis | 1 |  | -4 |  |
| Wnt/β-catenin Signaling | 0 |  | -4 |  |
| Reelin Signaling in Neurons | -2 |  | 0 |  |
|  |  |  |  |  |
| RANK Signaling in Osteoclasts |  | 0 | -3 |  |
|  |  |  |  |  |
| Axonal Guidance Signaling | 10 | 4 |  |  |
| IL-6 Signaling | 3 | 3 |  |  |
| Erythropoietin Signaling | 3 | 2 |  |  |
| VEGF Family Ligand-Receptor Interactions | 2 | 2 |  |  |
| Bladder Cancer Signaling | 2 | 2 |  |  |
| Role of Macrophages, Fibroblasts and Endothelial Cells in Rheumatoid Arthritis | 2 | 3 |  |  |
| Atherosclerosis Signaling | 1 | 3 |  |  |
| HIF1α Signaling | 1 | 3 |  |  |
|  |  |  |  |  |
| Adipogenesis pathway | -5 | -1 |  |  |
|  |  |  |  |  |
| p38 MAPK Signaling | 4 | -1 |  |  |
| ERK5 Signaling | 2 | -1 |  |  |
|  |  |  |  |  |
| ILK Signaling |  |  | 4 | 0 |
| CDK5 Signaling |  |  | 0 | 3 |
|  |  |  |  |  |
| ATM Signaling |  |  | -1 | -3 |
| Nucleotide Excision Repair Pathway |  |  | -3 | -2 |
| TNFR2 Signaling |  |  | -4 | -1 |
|  |  |  |  |  |
| Germ Cell-Sertoli Cell Junction Signaling |  |  | 3 | -2 |
| IL-17 Signaling |  |  | 0 | -1 |
| IL-1 Signaling |  |  | -1 | 2 |
|  |  |  |  |  |
| Allograft Rejection Signaling | 7 |  |  |  |
| Regulation of Actin-based Motility by Rho | 5 |  |  |  |
| VEGF Signaling | 4 |  |  |  |
| G Protein Signaling Mediated by Tubby | 3 |  |  |  |
| GPCR-Mediated Nutrient Sensing in Enteroendocrine Cells | 3 |  |  |  |
| HGF Signaling | 3 |  |  |  |
| Growth Hormone Signaling | 2 |  |  |  |
| NF-κB Activation by Viruses | 2 |  |  |  |
| Sphingosine-1-phosphate Signaling | 2 |  |  |  |
| Cellular Effects of Sildenafil (Viagra) | 2 |  |  |  |
| Human Embryonic Stem Cell Pluripotency | 2 |  |  |  |
| Communication between Innate and Adaptive Immune Cells | 1 |  |  |  |
| Geranylgeranyldiphosphate Biosynthesis | 0 |  |  |  |
| STAT3 Pathway | 0 |  |  |  |
| Oncostatin M Signaling | 0 |  |  |  |
| IL-2 Signaling | 0 |  |  |  |
| Role of Tissue Factor in Cancer | 0 |  |  |  |
| Oleate Biosynthesis II (Animals) | 0 |  |  |  |
|  |  |  |  |  |
| Estrogen Biosynthesis | -1 |  |  |  |
| Cytotoxic T Lymphocyte-mediated Apoptosis of Target Cells | -2 |  |  |  |
| Inhibition of Matrix Metalloproteases | -2 |  |  |  |
| Hematopoiesis from Pluripotent Stem Cells | -4 |  |  |  |
| Nicotine Degradation II | -4 |  |  |  |
| LPS/IL-1 Mediated Inhibition of RXR Function | -5 |  |  |  |
|  |  |  |  |  |
| ErbB Signaling |  | 4 |  |  |
| PAK Signaling |  | 4 |  |  |
| Role of Osteoblasts, Osteoclasts and Chondrocytes in Rheumatoid Arthritis |  | 3 |  |  |
| PPAR Signaling |  | 2 |  |  |
| Isoleucine Degradation I |  | 2 |  |  |
| Valine Degradation I |  | 2 |  |  |
| Antiproliferative Role of TOB in T Cell Signaling |  | 2 |  |  |
| Vitamin-C Transport |  | 1 |  |  |
| Amyotrophic Lateral Sclerosis Signaling |  | 1 |  |  |
| NGF Signaling |  | 1 |  |  |
| Methylthiopropionate Biosynthesis |  | 1 |  |  |
| Epoxysqualene Biosynthesis |  | 1 |  |  |
| Actin Nucleation by ARP-WASP Complex |  | 1 |  |  |
| Retinoate Biosynthesis II |  | 1 |  |  |
| Apoptosis Signaling |  | 0 |  |  |
|  |  |  |  |  |
| Antioxidant Action of Vitamin C |  | -1 |  |  |
| Netrin Signaling |  | -1 |  |  |
| 5-aminoimidazole Ribonucleotide Biosynthesis I |  | -1 |  |  |
| Oxidized GTP and dGTP Detoxification |  | -1 |  |  |
| Arsenate Detoxification I (Glutaredoxin) |  | -1 |  |  |
| 2-ketoglutarate Dehydrogenase Complex |  | -1 |  |  |
| Ascorbate Recycling (Cytosolic) |  | -1 |  |  |
| Induction of Apoptosis by HIV1 |  | -2 |  |  |
| Circadian Rhythm Signaling |  | -3 |  |  |
| Wnt/Ca+ pathway |  | -3 |  |  |
|  |  |  |  |  |
| EIF2 Signaling |  |  | 6 |  |
| Dopamine Receptor Signaling |  |  | 2 |  |
| Ceramide Signaling |  |  | 2 |  |
| Sertoli Cell-Sertoli Cell Junction Signaling |  |  | 1 |  |
| GNRH Signaling |  |  | 0 |  |
|  |  |  |  |  |
| Neuroprotective Role of THOP1 in Alzheimer's Disease |  |  | -2 |  |
| UVC-Induced MAPK Signaling |  |  | -2 |  |
| Toll-like Receptor Signaling |  |  | -4 |  |
| April Mediated Signaling |  |  | -4 |  |
| B Cell Activating Factor Signaling |  |  | -5 |  |
| Gα12/13 Signaling |  |  | -5 |  |
|  |  |  |  |  |
| Fatty Acid β-oxidation I |  |  |  | 5 |
| PEDF Signaling |  |  |  | 3 |
| Glycolysis I |  |  |  | 3 |
| Gluconeogenesis I |  |  |  | 3 |
| Cell Cycle Control of Chromosomal Replication |  |  |  | 3 |
| Triacylglycerol Degradation |  |  |  | 2 |
| Pyrimidine Ribonucleotides Interconversion |  |  |  | 2 |
| Pyrimidine Ribonucleotides De Novo Biosynthesis |  |  |  | 2 |
| Acetyl-CoA Biosynthesis I (Pyruvate Dehydrogenase Complex) |  |  |  | 2 |
| Ketolysis |  |  |  | 2 |
| Glutaryl-CoA Degradation |  |  |  | 2 |
| Cholesterol Biosynthesis I |  |  |  | 2 |
| Cholesterol Biosynthesis II (via 24,25-dihydrolanosterol) |  |  |  | 2 |
| Cholesterol Biosynthesis III (via Desmosterol) |  |  |  | 2 |
| Unfolded protein response |  |  |  | 1 |
| Dopamine-DARPP32 Feedback in cAMP Signaling |  |  |  | 1 |
| UVB-Induced MAPK Signaling |  |  |  | 0 |
| Acetate Conversion to Acetyl-CoA |  |  |  | 0 |
| autophagy |  |  |  | 0 |
| Ketogenesis |  |  |  | 0 |
| Mineralocorticoid Biosynthesis |  |  |  | 0 |
| Glucocorticoid Biosynthesis |  |  |  | 0 |
| Mevalonate Pathway I |  |  |  | 0 |
|  |  |  |  |  |
| MSP-RON Signaling Pathway |  |  |  | -2 |
| Ephrin A Signaling |  |  |  | -2 |
| Production of Nitric Oxide and Reactive Oxygen Species in Macrophages |  |  |  | -3 |
| Interferon Signaling |  |  |  | -3 |
| Endothelin-1 Signaling |  |  |  | -3 |
| IL-12 Signaling and Production in Macrophages |  |  |  | -3 |
| GADD45 Signaling |  |  |  | -3 |
| D-myo-inositol (1,4,5)-Trisphosphate Biosynthesis |  |  |  | -3 |
| HMGB1 Signaling |  |  |  | -3 |
| EGF Signaling |  |  |  | -4 |
| Estrogen Receptor Signaling |  |  |  | -4 |
| iNOS Signaling |  |  |  | -4 |
