# Supplementary material for: Genomic deletion of GIT2 induces a premature age-related thymic dysfunction and systemic immune system disruption
Source: Aging (Albany NY). 2017 Mar 4;9(3):706–30. doi: 10.18632/aging.101185 (PMC5391227; doi:10.18632/aging.101185)
Supplement: Supplementary file 20 [file aging-09-706-s020.docx]

**Table S19. Enrichr-based Reactome-2015 analysis of SASP (senescence-associated secretory phenotype) factors affected by GIT2 deletion.** The seventeen common proteins observed between multiple GIT2KO immune tissues and a canonical SASP pathway list were used as the input data for Enrichr pathway analysis. For each resultant enriched pathway term (p<0.05) the adjusted P value of pathway enrichment as well as the combined pathway ranking score (Combined Score) is reported.

| **Reactome Pathway Term** | **Adjusted P-value** | **Combined Score** |
| --- | --- | --- |
| Senescence-Associated Secretory Phenotype (SASP) | 2.64303E-40 | 190.8340781 |
| Cellular Senescence | 1.22248E-34 | 165.0596459 |
| Cellular responses to stress | 2.25296E-31 | 157.5123137 |
| Oxidative Stress Induced Senescence | 3.52578E-18 | 80.8695264 |
| Meiotic recombination | 5.88849E-18 | 81.23548846 |
| RNA Polymerase I Promoter Opening | 1.20861E-17 | 82.21421574 |
| DNA methylation | 1.86022E-17 | 80.65118327 |
| SIRT1 negatively regulates rRNA Expression | 3.63777E-17 | 78.71884925 |
| RNA Polymerase I Promoter Clearance | 7.66779E-17 | 73.61262688 |
| RNA Polymerase I Transcription | 8.62751E-17 | 73.40305898 |
| PRC2 methylates histones and DNA | 8.62751E-17 | 70.14913128 |
| Condensation of Prophase Chromosomes | 9.82184E-17 | 70.91594222 |
| Meiosis | 1.73396E-16 | 67.52787745 |
| Amyloids | 5.58805E-16 | 65.54975835 |
| RNA Polymerase I Chain Elongation | 1.16115E-15 | 65.44503575 |
| DNA Damage/Telomere Stress Induced Senescence | 1.3814E-15 | 65.6792304 |
| formation of the beta-catenin:TCF transactivating complex | 1.3814E-15 | 64.23036255 |
| RNA Polymerase I, RNA Polymerase III, and Mitochondrial Transcription | 1.79754E-15 | 65.4157013 |
| Packaging Of Telomere Ends | 3.42244E-15 | 61.91529165 |
| Transcriptional regulation by small RNAs | 6.4834E-15 | 60.68867448 |
| NoRC negatively regulates rRNA expression | 6.94371E-15 | 59.34605355 |
| Negative epigenetic regulation of rRNA expression | 9.33145E-15 | 58.65191546 |
| M Phase | 3.71451E-14 | 65.71592163 |
| Epigenetic regulation of gene expression | 4.68722E-14 | 55.51722535 |
| Regulatory RNA pathways | 8.96703E-14 | 54.11259572 |
| RNF mutants show enhanced WNT signaling and proliferation | 1.20832E-13 | 60.13484407 |
| misspliced LRP5 mutants have enhanced beta-catenin-dependent signaling | 1.20832E-13 | 60.08583748 |
| XAV939 inhibits tankyrase, stabilizing AXIN | 1.20832E-13 | 59.56278788 |
| TCF dependent signaling in response to WNT | 1.20832E-13 | 59.51233898 |
| Deposition of new CENPA-containing nucleosomes at the centromere | 1.20832E-13 | 53.4118865 |
| Nucleosome assembly | 1.20832E-13 | 52.53464761 |
| Mitotic Prophase | 1.20832E-13 | 52.29176326 |
| Meiotic synapsis | 1.95875E-13 | 48.12179658 |
| Transcription | 1.98781E-13 | 55.27836698 |
| Telomere Maintenance | 2.69423E-13 | 47.51043622 |
| Signaling by WNT in cancer | 2.91466E-13 | 57.65895902 |
| Chromosome Maintenance | 3.81064E-12 | 44.51450125 |
| Signaling by Wnt | 4.03175E-12 | 54.26191903 |
| Chromatin organization | 2.18643E-11 | 45.0846727 |
| Chromatin modifying enzymes | 2.18643E-11 | 44.63088297 |
| HDACs deacetylate histones | 9.80815E-09 | 28.77551398 |
| HATs acetylate histones | 1.65505E-07 | 25.61976092 |
| RMTs methylate histone arginines | 3.94868E-07 | 21.14250356 |
| Regulation of APC/C activators between G1/S and early anaphase | 0.000265909 | 13.93221646 |
| Regulation of mitotic cell cycle | 0.000318471 | 13.85661115 |
| APC/C-mediated degradation of cell cycle proteins | 0.000318471 | 13.68038988 |
| Cell Cycle Checkpoints | 0.000888979 | 12.06749144 |
| APC/C:Cdc20 mediated degradation of Cyclin B | 0.000960727 | 7.888501988 |
| APC-Cdc20 mediated degradation of Nek2A | 0.001132693 | 7.984879243 |
| Mitotic G1-G1/S phases | 0.001244239 | 11.35248573 |
| Oncogene Induced Senescence | 0.002123955 | 8.124372888 |
| G1 Phase | 0.002888092 | 8.147931325 |
| Cyclin D associated events in G1 | 0.002888092 | 8.14771478 |
| CDK-mediated phosphorylation and removal of Cdc6 | 0.00490987 | 7.708763472 |
| p53-Dependent G1/S DNA damage checkpoint | 0.006039029 | 8.11811198 |
| p53-Dependent G1 DNA Damage Response | 0.006039029 | 8.111544343 |
| SCF(Skp2)-mediated degradation of p27/p21 | 0.006039029 | 7.651761176 |
| G1/S DNA Damage Checkpoints | 0.006460701 | 8.148989324 |
| Autodegradation of Cdh1 by Cdh1:APC/C | 0.006460701 | 7.55074562 |
| APC/C:Cdc20 mediated degradation of Securin | 0.007221544 | 7.531049018 |
| Cyclin E associated events during G1/S transition | 0.007324996 | 7.493390228 |
| Cyclin A:Cdk2-associated events at S phase entry | 0.007428355 | 7.443249143 |
| Switching of origins to a post-replicative state | 0.007723816 | 7.257824699 |
| Orc1 removal from chromatin | 0.007723816 | 7.233949115 |
| Cdc20:Phospho-APC/C mediated degradation of Cyclin A | 0.007723816 | 7.103591152 |
| APC/C:Cdh1 mediated degradation of Cdc20 and other APC/C:Cdh1 targeted proteins in late mitosis/early G1 | 0.007723816 | 7.093402263 |
| APC:Cdc20 mediated degradation of cell cycle proteins prior to satisfation of the cell cycle checkpoint | 0.007723816 | 7.09005603 |
| Signalling by NGF | 0.007919652 | 8.273746379 |
| Removal of licensing factors from origins | 0.007919652 | 7.141052055 |
| APC/C:Cdc20 mediated degradation of mitotic proteins | 0.007919652 | 7.013212283 |
| Activation of APC/C and APC/C:Cdc20 mediated degradation of mitotic proteins | 0.007919652 | 6.906733983 |
| Regulation of DNA replication | 0.008125415 | 6.90968882 |
| TRAF6 Mediated Induction of proinflammatory cytokines | 0.008125415 | 6.223023549 |
| M/G1 Transition | 0.009431818 | 6.960619433 |
| DNA Replication Pre-Initiation | 0.009431818 | 6.793561166 |
| Toll Like Receptor 10 (TLR10) Cascade | 0.009734443 | 6.168661369 |
| MyD88 cascade initiated on plasma membrane | 0.009734443 | 6.130581173 |
| Toll Like Receptor 5 (TLR5) Cascade | 0.009734443 | 6.07060336 |
| TRAF6 mediated induction of NFkB and MAP kinases upon TLR7/8 or 9 activation | 0.009835151 | 6.118085419 |
| MyD88 dependent cascade initiated on endosome | 0.010036312 | 6.279410288 |
| Toll Like Receptor 7/8 (TLR7/8) Cascade | 0.010036312 | 6.190598079 |
| Toll Like Receptor 9 (TLR9) Cascade | 0.010819462 | 6.211600807 |
| MyD88:Mal cascade initiated on plasma membrane | 0.010987355 | 6.076306892 |
| Toll Like Receptor TLR6:TLR2 Cascade | 0.010987355 | 6.076005258 |
| Toll Like Receptor TLR1:TLR2 Cascade | 0.010987355 | 6.033130282 |
| Toll Like Receptor 2 (TLR2) Cascade | 0.010987355 | 5.949941548 |
| Synthesis of DNA | 0.011314372 | 6.140261138 |
| MyD88-independent cascade | 0.011383928 | 5.884967291 |
| Toll Like Receptor 3 (TLR3) Cascade | 0.011383928 | 5.817047537 |
| TRIF-mediated TLR3/TLR4 signaling | 0.011383928 | 5.734023146 |
| DNA Replication | 0.0126344 | 5.838620303 |
| G1/S Transition | 0.013445261 | 5.661882913 |
| Activated TLR4 signalling | 0.014109487 | 5.496278887 |
| G2/M Transition | 0.014109487 | 5.257888825 |
| DNA Repair | 0.014109487 | 5.161634985 |
| Mitotic G2-G2/M phases | 0.014109487 | 5.116708518 |
| G2 Phase | 0.014109487 | -24.68851554 |
| ATM mediated response to DNA double-strand break | 0.014109487 | -29.02158283 |
| ATM mediated phosphorylation of repair proteins | 0.014109487 | -30.27827371 |
| Toll Like Receptor 4 (TLR4) Cascade | 0.015658118 | 5.350942341 |
| S Phase | 0.015749382 | 5.446452631 |
| Factors involved in megakaryocyte development and platelet production | 0.015840653 | 4.772392536 |
| RSK activation | 0.016109495 | -18.75927659 |
| CREB phosphorylation | 0.018606253 | -18.86469898 |
| Toll-Like Receptors Cascades | 0.019709676 | 4.859318794 |
| Downregulation of ERBB4 signaling | 0.020659831 | -10.1759073 |
| Recruitment of repair and signaling proteins to double-strand breaks | 0.020659831 | -19.12282159 |
| Separation of Sister Chromatids | 0.024421496 | 4.522956102 |
| Regulation of the Fanconi anemia pathway | 0.024874269 | -7.30501912 |
| IRAK2 mediated activation of TAK1 complex upon TLR7/8 or 9 stimulation | 0.024874269 | -7.821697969 |
| IRAK2 mediated activation of TAK1 complex | 0.024874269 | -7.932466255 |
| Mitotic Anaphase | 0.026171848 | 4.321681136 |
| Mitotic Metaphase and Anaphase | 0.026171848 | 4.319828479 |
| Synthesis And Processing Of GAG, GAGPOL Polyproteins | 0.026171848 | -7.093989854 |
| Membrane binding and targetting of GAG proteins | 0.026171848 | -7.457295797 |
| Interleukin-6 signaling | 0.026171848 | -9.545496983 |
| Signaling by FGFR in disease | 0.027045179 | 4.39539217 |
| Downregulation of ERBB2:ERBB3 signaling | 0.029628558 | -5.658302366 |
| Assembly Of The HIV Virion | 0.029628558 | -6.033067023 |
| NF-kB is activated and signals survival | 0.029628558 | -6.12257565 |
| p75NTR recruits signalling complexes | 0.029628558 | -6.155611046 |
| Receptor-ligand binding initiates the second proteolytic cleavage of Notch receptor | 0.031633496 | -5.162632982 |
| NRIF signals cell death from the nucleus | 0.032545653 | -4.636618168 |
| Regulation of innate immune responses to cytosolic DNA | 0.032545653 | -4.855680648 |
| Constitutive Signaling by NOTCH1 HD Domain Mutants | 0.032545653 | -4.856021289 |
| Glycogen synthesis | 0.032545653 | -4.933876813 |
| Association of licensing factors with the pre-replicative complex | 0.032545653 | -4.965623536 |
| NGF signalling via TRKA from the plasma membrane | 0.033031849 | 3.864564285 |
| Antigen processing: Ubiquitination & Proteasome degradation | 0.03327298 | 3.361364904 |
| Spry regulation of FGF signaling | 0.03327298 | -3.936417602 |
| TGF-beta receptor signaling in EMT (epithelial to mesenchymal transition) | 0.03327298 | -4.499413612 |
| TRAF6 mediated induction of TAK1 complex | 0.03327298 | -4.50370214 |
| p75NTR signals via NF-kB | 0.03327298 | -4.57933044 |
| Phosphorylation of the APC/C | 0.034165422 | -6.208278374 |
| Conversion from APC/C:Cdc20 to APC/C:Cdh1 in late anaphase | 0.034165422 | -6.678175461 |
| Homologous recombination repair of replication-independent double-strand breaks | 0.034165422 | -6.715786519 |
| Homologous Recombination Repair | 0.034165422 | -6.972211036 |
| Activation of IRF3/IRF7 mediated by TBK1/IKK epsilon | 0.035134869 | -3.64728077 |
| Oxygen-dependent proline hydroxylation of Hypoxia-inducible Factor Alpha | 0.035134869 | -4.390514307 |
| Inhibition of the proteolytic activity of APC/C required for the onset of anaphase by mitotic spindle checkpoint components | 0.035134869 | -6.230674242 |
| Inactivation of APC/C via direct inhibition of the APC/C complex | 0.035134869 | -6.318137999 |
| Constitutive Signaling by Ligand-Responsive EGFR Cancer Variants | 0.036299663 | -3.533137141 |
| Signaling by FGFR1 fusion mutants | 0.036299663 | -5.782210092 |
| Mitotic Spindle Checkpoint | 0.036299663 | -5.840859052 |
| NOTCH2 Activation and Transmission of Signal to the Nucleus | 0.039270512 | -3.203648736 |
| regulation of FZD by ubiquitination | 0.039270512 | -3.287224547 |
| ERK/MAPK targets | 0.039270512 | -4.361718072 |
| Fanconi Anemia pathway | 0.040301631 | -3.338595987 |
| Cyclin A/B1 associated events during G2/M transition | 0.040301631 | -3.9952774 |
| POU5F1 (OCT4), SOX2, NANOG activate genes related to proliferation | 0.040301631 | -4.506377393 |
| Class I MHC mediated antigen processing & presentation | 0.041364249 | 2.807836436 |
| Downregulation of SMAD2/3:SMAD4 transcriptional activity | 0.041364249 | -3.254184565 |
| Double-Strand Break Repair | 0.041364249 | -4.728071094 |
| IKK complex recruitment mediated by RIP1 | 0.042240696 | -3.071275191 |
| Nuclear Events (kinase and transcription factor activation) | 0.042240696 | -3.638413686 |
| Growth hormone receptor signaling | 0.042240696 | -3.951041475 |
| Budding and maturation of HIV virion | 0.042883585 | -3.031237613 |
| Cellular response to hypoxia | 0.042883585 | -3.073163835 |
| Regulation of Hypoxia-inducible Factor (HIF) by oxygen | 0.042883585 | -3.195329858 |
| G0 and Early G1 | 0.042883585 | -3.919196077 |
| Signaling by Leptin | 0.04376062 | -2.708951196 |
| EGFR downregulation | 0.04376062 | -2.814381177 |
| Downregulation of TGF-beta receptor signaling | 0.04376062 | -2.969579094 |
| CREB phosphorylation through the activation of Ras | 0.044874974 | -3.482128152 |
| Recycling pathway of L1 | 0.044874974 | -3.843867671 |
| Endosomal Sorting Complex Required For Transport (ESCRT) | 0.046238212 | -2.789582108 |
| SMAD2/SMAD3:SMAD4 heterotrimer regulates transcription | 0.047300585 | -2.491702719 |
| PKMTs methylate histone lysines | 0.047300585 | -3.533753781 |
| Activated NOTCH1 Transmits Signal to the Nucleus | 0.04777463 | -2.469819499 |
| Signaling by FGFR1 mutants | 0.04777463 | -2.655352578 |
| MAPK targets/ Nuclear events mediated by MAP kinases | 0.04777463 | -2.833350844 |
| Activation of the pre-replicative complex | 0.04777463 | -3.017641622 |
